# Supplementary material for: Genetically stable kill-switch using “demon and angel” expression construct of essential genes
Source: Front Bioeng Biotechnol. 2024 Feb 28;12:1365870. doi: 10.3389/fbioe.2024.1365870 (PMC10933106; doi:10.3389/fbioe.2024.1365870)
Supplement: Supplementary file 1 [file DataSheet1.PDF]

Supplementary Information

Genetically stable kill-switch using "demon and angel" expression construct of essential genes

Yusuke Kato[1]* and Hirotada Mori[2#]

1. Institute of Agrobiological Sciences, National Agriculture and Food Research Organization (NARO), Tsukuba, Ibaraki, Japan

2. Laboratory of Systems Microbiology, Data Science Center, Nara Institute of Science and Technology, Ikoma, Nara, Japan

*Correspondence.

kato@affrc.go.jp

#Present address.

Innovation laboratory of Systems Microbiology and Synthetic Biology, Institute of Animal Science, Guangdong Academy of Agricultural Sciences. Guangzhou, Guangdong , China.

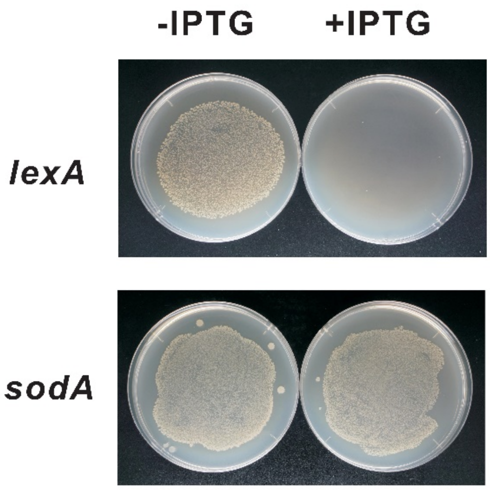

Figure S1. Toxic overexpression of endogenous genes. We tested the *E. coli* BL21-AI strain with an ASKA-plasmid containing an IPTG-inducible overexpression construct of the indicated gene. Overnight cultures were diluted 10^3-fold and inoculated onto LB-agar plates with or without 5 mM IPTG. *lexA* and *sodA* showed the toxic and non-toxic overexpression phenotype, respectively.

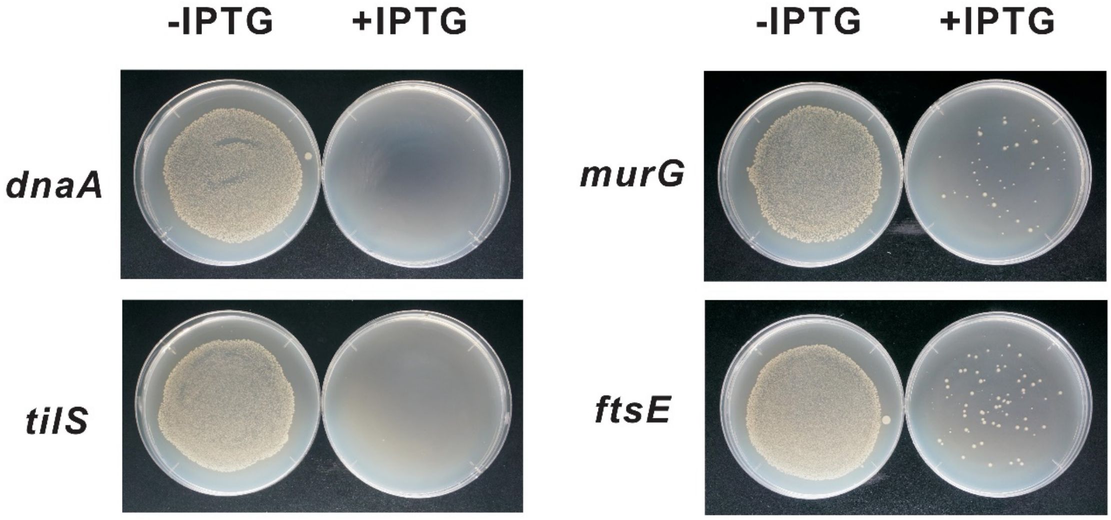

Figure S2. Various toxicity severity of essential genes. Overnight cultures were diluted 10^3-fold and inoculated onto LB-agar plates with or without 5 mM IPTG.

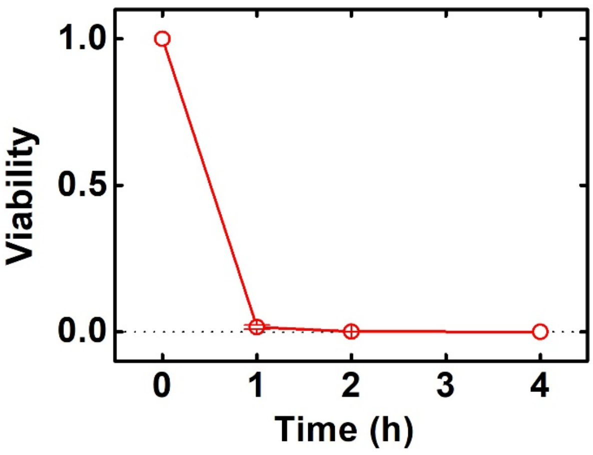

Figure S3. Mode of action for *tyrS* toxic-overexpression. Viability of BL21-AI [tyrS-; ASKA-(tyrS)] after addition of IPTG (final 5 mM) was determined. Data points represent the mean ± s.d. of three biological replicates.

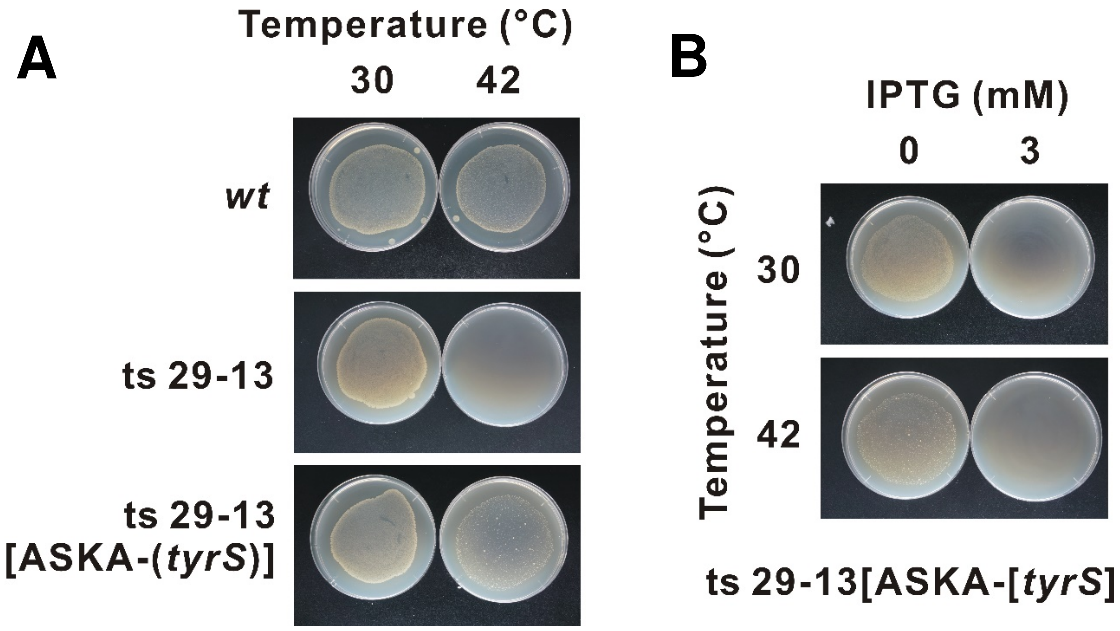

Figure S4. Evaluation of the "demon and angel" *tyrS* expression construct in W3110 ts 29-13. W3110 and its derivative strains were evaluated. (A) Maintenance of host survival and growth in the OFF state. W3110 ts 29-13 can grow at 30°C but not at 42°C, due to a temperature-sensitive mutation in *tyrS*. The ASKA- plasmid containing a conditional *tyrS* overexpression construct is carried in W3110 ts 29-13[ASKA-(tyrS)]. Since this experiment was performed in the absence of the inducer, IPTG, the overexpression of *tyrS* was not induced. Meanwhile, leakage expression of *tyrS* was expected. (B) Conditional host killing. Overexpression of *tyrS* encoded on the ASKA- plasmid is induced by 3 mM IPTG.

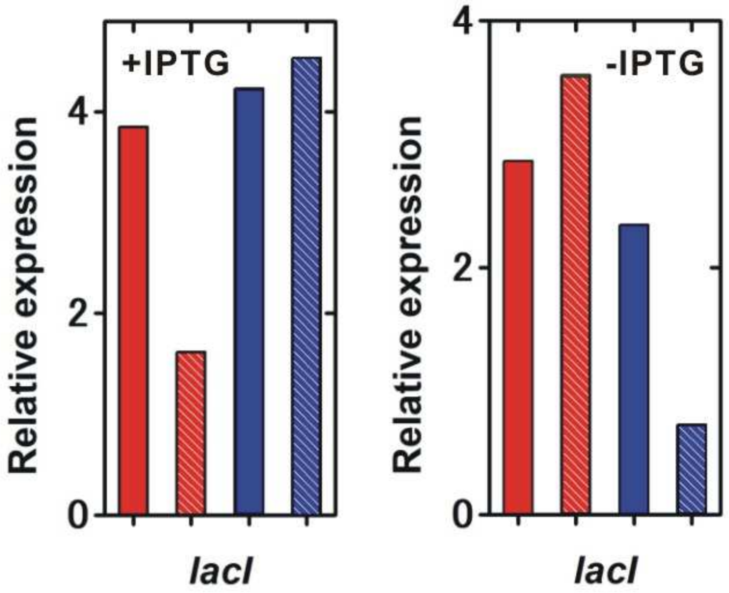

Figure S5. Relative expression of *lacI*. Comprehensive analysis of gene expression was performed using RNA-seq. Red, red-striped, blue and blue-striped columns indicate P0S, P31S, P14R and P30R, respectively. The expression level was normalized by that of a constitutively expressed gene, *gltA*, citrate synthase.

Table S1. Growth inhibition and GFP expression in overexpression of ASKA+ plasmids. Total 4269 clones were tested. Results are listed in alphabetical order by gene name. The intensity of GFP expression was evaluated on the following three levels： gg, strong; g, weak; o, not detected. Growth inhibition was evaluated on the following three levels: tt, no growth; t, slower growth; o, normal growth.

Gene	Feature	Product	Location	Function	Process	GFP fluorescence	IPTG growth
aaeA	CDS	p-hydroxybenzoic acid efflux system component	Periplasmic	COG1566;Multidrug resistance efflux pump			
aaeB	CDS	p-hydroxybenzoic acid efflux system component	Integral Membrane Protein	COG1289;Predicted membrane protein		o	tt
aaeR	CDS	predicted DNA-binding transcriptional regulator, efflux system	Cytoplasmic	COG0583;Transcriptional regulator	GO:0006350 transcription	o	tt
aaeX	CDS	membrane protein of efflux system	Integral Membrane Protein				
aas	CDS	fused 2-acylglycerophospho-ethanolamine acyl transferase -!- acyl-acyl carrier protein synthetase	Integral Membrane Protein	COG0204;1-acyl-sn-glycerol-3-phosphate acyltransferase -!- COG0318;Acyl-CoA synthetases (AMP-forming)/AMP-acid ligases II	GO:0006464 protein modification	o	tt
aat	CDS	leucyl/phenylalanyl-tRNA-protein transferase	Cytoplasmic	COG2360;Leu/Phe-tRNA-protein transferase	GO:0006508 proteolysis and peptidolysis	gg	tt
abgA	CDS	predicted peptidase, aminobenzoyl-glutamate utilization protein	Cytoplasmic		GO:0016052 carbohydrate catabolism		
abgB	CDS	predicted peptidase, aminobenzoyl-glutamate utilization protein	Cytoplasmic	COG1473;Metal-dependent amidase/aminoacylase/carboxypeptidase	GO:0016052 carbohydrate catabolism	gg	t
abgR	CDS	predicted DNA-binding transcriptional regulator	Cytoplasmic	COG0583;Transcriptional regulator	GO:0016052 carbohydrate catabolism -!- GO:0006350	gg	tt
abgT	CDS	predicted cryptic aminobenzoyl-glutamate transporter	Integral Membrane Protein	COG2978;Putative p-aminobenzoyl-glutamate transporter			
abrB	CDS	predicted regulator	Integral Membrane Protein				
accA	CDS	acetylCoA carboxylase, carboxytransferase, alpha subunit	Cytoplasmic	COG0825;Acetyl-CoA carboxylase alpha subunit		gg	o
accB	CDS	acetyl CoA carboxylase, BCCP subunit	Cytoplasmic	COG0511;Biotin carboxyl carrier protein	GO:0042966 biotin carboxyl carrier protein biosynthesis	o	tt
accC	CDS	acetyl-CoA carboxylase, biotin carboxylase subunit	Cytoplasmic	COG0439;Biotin carboxylase		o	tt
accD	CDS	acetylCoA carboxylase, beta (carboxyltranferase) subunit	Cytoplasmic	COG0777;Acetyl-CoA carboxylase beta subunit		gg	t
aceA	CDS	isocitrate lyase	Cytoplasmic	COG2224;Isocitrate lyase	GO:0006097 glyoxylate cycle	o	tt
aceB	CDS	malate synthase A	Cytoplasmic	COG2225;Malate synthase	GO:0006097 glyoxylate cycle	gg	t
aceE	CDS	pyruvate dehydrogenase, decarboxylase component E1, thiamin-binding	Cytoplasmic	COG2609;Pyruvate dehydrogenase complex, dehydrogenase (E1) component	GO:0016052 carbohydrate catabolism -!- GO:0006086 acetyl-CoA biosynthesis from pyruvate -!- GO:0006096 glycolysis -!- GO:0009436 glyoxylate catabolism -!- GO:0009061 anaerobic respiration	gg	tt
aceF	CDS	pyruvate dehydrogenase, dihydrolipoyltransacetylase component E2	Cytoplasmic	COG0508;Pyruvate/2-oxoglutarate dehydrogenase complex, dihydrolipoamide acyltransferase (E2) component, and related enzymes	GO:0016052 carbohydrate catabolism -!- GO:0006086 acetyl-CoA biosynthesis from pyruvate -!- GO:0006096 glycolysis -!- GO:0009436 glyoxylate catabolism -!- GO:0009061 anaerobic respiration	o	tt
aceK	CDS	isocitrate dehydrogenase kinase/phosphatase	Cytoplasmic	COG4579;Isocitrate dehydrogenase kinase/phosphatase	GO:0006097 glyoxylate cycle -!- GO:0006464 protein modification	o	t
ackA	CDS	acetate kinase A and propionate kinase 2	Cytoplasmic	COG0282;Acetate kinase	GO:0016052 carbohydrate catabolism -!- GO:0009063 amino acid catabolism -!- GO:0042867 pyruvate catabolism -!- GO:0045733 acetate catabolism	gg	t
acnA	CDS	aconitate hydratase 1	Cytoplasmic	COG1048;Aconitase A	GO:0006099 tricarboxylic acid cycle -!- GO:0009061 anaerobic respiration	g	tt
acnB	CDS	bifunctional aconitate hydratase 2 -!- 2-methylisocitrate dehydratase	Cytoplasmic	COG1049;Aconitase B	GO:0006099 tricarboxylic acid cycle -!- GO:0009060 aerobic respiration -!- GO:0009061 anaerobic respiration	gg	o
acpP	CDS	acyl carrier protein (ACP)	Cytoplasmic	COG0236;Acyl carrier protein	GO:0042967 acyl-carrier protein biosynthesis	gg	t
acpS	CDS	holo-[acyl-carrier-protein] synthase 1	Cytoplasmic	COG0736;Phosphopantetheinyl transferase (holo-ACP synthase)		gg	t
acpT	CDS	holo-(acyl carrier protein) synthase 2	Cytoplasmic	COG2091;Phosphopantetheinyl transferase		o	t
acrA	CDS	multidrug efflux system	Inner Membrane Lipoprotein	COG0845;Membrane-fusion protein	GO:0042493 response to drug	o	tt
acrB	CDS	multidrug efflux system protein	Integral Membrane Protein	COG0841;Cation/multidrug efflux pump	GO:0042493 response to drug	o	tt
acrD	CDS	aminoglycoside/multidrug efflux system	Integral Membrane Protein	COG0841;Cation/multidrug efflux pump	GO:0042493 response to drug	o	tt
acrE	CDS	cytoplasmic membrane lipoprotein	Inner Membrane Lipoprotein	COG0845;Membrane-fusion protein		o	tt
acrF	CDS	multidrug efflux system protein	Integral Membrane Protein	COG0841;Cation/multidrug efflux pump	GO:0042493 response to drug	o	o
acrR	CDS	DNA-binding transcriptional regulator	Cytoplasmic	COG1309;Transcriptional regulator	GO:0006350 transcription -!- GO:0042493 response to drug	gg	tt
acs	CDS	bifunctional acetyl-CoA synthetase -!- propionyl-CoA synthetase	Cytoplasmic	COG0365;Acyl-coenzyme A synthetases/AMP-(fatty) acid ligases	GO:0016052 carbohydrate catabolism -!- GO:0045733 acetate	gg	t
actP	CDS	acetate transporter	Integral Membrane Protein	COG4147;Predicted symporter		o	tt
ada	CDS	fused DNA-binding transcriptional dual regulator -!- O6-methylguanine-DNA methyltransferase	Cytoplasmic	COG2169;Adenosine deaminase -!- COG0350;Methylated DNA-protein cysteine methyltransferase	GO:0006281 DNA repair -!- GO:0006350 transcription	o	tt
add	CDS	adenosine deaminase	Cytoplasmic	COG1816;Adenosine deaminase	GO:0015949 nucleobase, nucleoside and nucleotide interconversion	gg	o
ade	CDS	cryptic adenine deaminase	Cytoplasmic	COG1001;Adenine deaminase	GO:0008152 metabolism	g	o
adhE	CDS	fused acetaldehyde-CoA dehydrogenase -!- iron-dependent alcohol dehydrogenase -!- pyruvate-formate lyase deactivase	Cytoplasmic	COG1012;NAD-dependent aldehyde dehydrogenases -!- COG1454;Alcohol dehydrogenase, class IV	GO:0006113 fermentation	gg	t
adhP	CDS	alcohol dehydrogenase, 1-propanol preferring	Cytoplasmic		GO:0009061 anaerobic respiration	gg	t
adiA	CDS	biodegradative arginine decarboxylase	Cytoplasmic	COG1982;Arginine/lysine/ornithine decarboxylases	GO:0009063 amino acid catabolism		
adiC	CDS	arginine:agmatin	Integral Membrane Protein	COG0531;Amino acid transporters		o	tt
adiY	CDS	DNA-binding transcriptional activator	Cytoplasmic	COG2207;AraC-type DNA-binding domain-containing proteins	GO:0006350 transcription	gg	t
adk	CDS	adenylate kinase	Cytoplasmic	COG0563;Adenylate kinase and related kinases	GO:0015949 nucleobase, nucleoside and nucleotide interconversion -!- GO:0009152 purine ribonucleotide biosynthesis	gg	o
aegA	CDS	fused predicted FeS binding subunit -!- predicted NAD/FAD-binding subunit of oxidoreductase	Cytoplasmic	COG1142;Fe-S-cluster-containing hydrogenase components 2 -!- COG0493;NADPH-dependent glutamate synthase beta chain and related oxidoreductases		gg	t
aer	CDS	fused signal transducer for aerotaxis sensory component -!- methyl accepting chemotaxis component	Membrane Anchored	COG2202;FOG: PAS/PAC domain -!- COG0840;Methyl-accepting chemotaxis protein	GO:0016052 carbohydrate catabolism -!- GO:0042330 taxis	o	tt
aes	CDS	acetyl esterase	Cytoplasmic	COG0657;Esterase/lipase	GO:0016052 carbohydrate	o	t
afuB	CDS	CP4-6 prophage; predicted ferric trasnporter subunit -!- membrane component of ABC superfamily	Integral Membrane Protein	COG1178;ABC-type Fe3+ transport system, permease component		o	tt
afuC	CDS	CP4-6 prophage; predicted ferric transporter subunit -!- ATP-binding component of ABC superfamily	Cytoplasmic	COG3842;ABC-type spermidine/putrescine transport systems, ATPase components		gg	tt
agaA	CDS	predicted truncated N-acetylgalactosamine-6-phosphate deacetylase	Cytoplasmic				
agaB	CDS	N-acetylgalactosamine-specific enzyme IIB component of PTS	Cytoplasmic	COG3444;Phosphotransferase system, mannose/fructose/N-acetylgalactosamine-specific component IIB		gg	t
agaC	CDS	N-acetylgalactosamine-specific enzyme IIC component of PTS	Integral Membrane Protein	COG3715;Phosphotransferase system, mannose/fructose/N-acetylgalactosamine-specific component IIC		o	tt
agaD	CDS	N-acetylgalactosamine-specific enzyme IID component of PTS	Integral Membrane Protein	COG3716;Phosphotransferase system, mannose/fructose/N-acetylgalactosamine-specific component IID	GO:0046349 amino sugar biosynthesis	o	tt
agaI	CDS	galactosamine-6-phosphate isomerase	Cytoplasmic	COG0363;6-phosphogluconolactonase/Glucosamine-6-ph osphate isomerase/deaminase		gg	t
agaR	CDS	DNA-binding transcriptional dual regulator	Cytoplasmic	COG1349;Transcriptional regulators of sugar metabolism	GO:0046349 amino sugar biosynthesis -!- GO:0006350 transcription	g	tt
agaS	CDS	tagatose-6-phosphate ketose/aldose isomerase	Cytoplasmic	COG2222;Predicted phosphosugar isomerases		gg	o
agaV	CDS	N-acetylgalactosamine-specific enzyme IIB component of PTS	Cytoplasmic		GO:0046349 amino sugar biosynthesis	gg	o
agaW	CDS	N-acetylgalactosamine-specific enzyme IIC component of PTS, fragment (pseudogene)	Integral Membrane Protein	COG3715;Phosphotransferase system, mannose/fructose/N-acetylgalactosamine-specific component IIC	GO:0046349 amino sugar biosynthesis	o	tt
agp	CDS	glucose-1-phosphatase/inositol phosphatase	Periplasmic		GO:0006006 glucose metabolism	o	tt
ahpC	CDS	alkyl hydroperoxide reductase, C22 subunit	Cytoplasmic	COG0450;Peroxiredoxin	GO:0006805 xenobiotic metabolism	gg	t
ahpF	CDS	alkyl hydroperoxide reductase, F52a subunit, FAD/NAD(P)-binding	Cytoplasmic	COG3634;Alkyl hydroperoxide reductase, large subunit	GO:0006805 xenobiotic metabolism	gg	tt
aidB	CDS	isovaleryl CoA dehydrogenase	Cytoplasmic		GO:0006950 response to stress		
ais	CDS	conserved protein	Membrane Anchored			g	tt
alaS	CDS	alanyl-tRNA synthetase	Cytoplasmic	COG0013;Alanyl-tRNA synthetase	GO:0006418 amino acid activation	gg	tt
alaT	tRNA	tRNA-Ala(UGC) (Alanine tRNA 1B)					
alaU	tRNA	tRNA-Ala(UGC) (Alanine tRNA 1B)					
alaV	tRNA	tRNA-Ala(UGC) (Alanine tRNA 1B)					
alaW	tRNA	tRNA-Ala(GGC), (Alanine tRNA 2)					
alaX	tRNA	tRNA-Ala(GGC) (Alanine tRNA 2)					
aldA	CDS	aldehyde dehydrogenase A, NAD-linked	Cytoplasmic	COG1012;NAD-dependent aldehyde dehydrogenases	GO:0016052 carbohydrate catabolism -!- GO:0009438 methylglyoxal metabolism -!- GO:0006113 fermentation -!- GO:0009441 glycolate metabolism	o	t
aldB	CDS	aldehyde dehydrogenase B	Cytoplasmic		GO:0016052 carbohydrate catabolism -!- GO:0009438 methylglyoxal metabolism	g	o
alkA	CDS	3-methyl-adenine DNA glycosylase II	Cytoplasmic	COG0122;3-methyladenine DNA glycosylase/8-oxoguanine DNA	GO:0006281 DNA repair	g	o
alkB	CDS	oxidative demethylase of N1-methyladenine or N3-methylcytosine DNA lesions	Cytoplasmic	COG3145;Alkylated DNA repair protein	GO:0006281 DNA repair	gg	o
allA	CDS	ureidoglycolate hydrolase	Cytoplasmic	COG3194;Ureidoglycolate hydrolase	GO:0006807 nitrogen metabolism -!- GO:0009442 allantoin assimilation	gg	t
allB	CDS	allantoinase	Cytoplasmic	COG0044;Dihydroorotase and related cyclic amidohydrolases	GO:0006807 nitrogen metabolism -!- GO:0009442 allantoin assimilation	gg	t

allC	CDS	allantoate amidohydrolase	Cytoplasmic	COG0624;Acetylornithine deacetylase/Succinyl-diaminopimelate desuccinylase and related deacylases	GO:0006807 nitrogen metabolism -!- GO:0009442 allantoin assimilation	o	t
allD	CDS	ureidoglycolate dehydrogenase	Cytoplasmic	COG2055;Malate/L-lactate dehydrogenases	GO:0006807 nitrogen metabolism -!- GO:0009442 allantoin assimilation	gg	t
allR	CDS	DNA-binding transcriptional regulator	Cytoplasmic	COG1414;Transcriptional regulator	GO:0006350 transcription	gg	tt
alpA	CDS	CP4-57 prophage; DNA-binding transcriptional activator	Cytoplasmic	COG3311;Predicted transcriptional regulator	GO:0006350 transcription	o	tt
alr	CDS	alanine racemase 1, PLP-binding, biosynthetic	Cytoplasmic	COG0787;Alanine racemase	GO:0006523 alanine biosynthesis	gg	o
alsA	CDS	fused D-allose transporter subunits -!- ATP-binding components of ABC superfamily	Cytoplasmic	COG1129;ABC-type sugar transport system, ATPase component	GO:0016052 carbohydrate catabolism	g	o
alsB	CDS	D-allose transporter subunit -!- periplasmic-binding component of ABC superfamily	Periplasmic	COG1879;ABC-type sugar transport system, periplasmic component	GO:0016052 carbohydrate catabolism	o	tt
alsC	CDS	D-allose transporter subunit -!- membrane component of ABC superfamily	Integral Membrane Protein	COG1172;Ribose/xylose/arabinose/galactoside ABC-type transport systems, permease components	GO:0016052 carbohydrate catabolism	o	tt
alsE	CDS	allulose-6-phosphate 3-epimerase	Cytoplasmic	COG0036;Pentose-5-phosphate-3-epimerase		gg	o
alsK	CDS	D-allose kinase	Cytoplasmic	COG1940;Transcriptional regulator/sugar kinase	GO:0006355 regulation of transcription, DNA-dependent		
alx	CDS	predicted inner membrane protein, part of terminus	Integral Membrane Protein		GO:0006950 response to stress		
amiA	CDS	N-acetylmuramoyl-l-alanine amidase I	Periplasmic	COG0860;N-acetylmuramoyl-L-alanine amidase	GO:0009252 peptidoglycan biosynthesis	gg	t
amiB	CDS	N-acetylmuramoyl-l-alanine amidase II	Periplasmic	COG0860;N-acetylmuramoyl-L-alanine amidase	GO:0009252 peptidoglycan biosynthesis	o	tt
amiC	CDS	N-acetylmuramoyl-L-alanine amidase	Periplasmic		GO:0009252 peptidoglycan biosynthesis		
amn	CDS	AMP nucleosidase	Cytoplasmic	COG0775;Nucleoside phosphorylase	GO:0015949 nucleobase, nucleoside and nucleotide interconversion	gg	o
ampC	CDS	beta-lactamase/D-alanine carboxypeptidase	Periplasmic	COG1680;Beta-lactamase class C and other penicillin binding proteins	GO:0009252 peptidoglycan biosynthesis -!- GO:0042493 response to drug	o	tt
ampD	CDS	N-acetyl-anhydromuranmyl-L-alanine amidase	Cytoplasmic	COG3023;Negative regulator of beta-lactamase expression	GO:0000270 peptidoglycan metabolism	gg	o
ampE	CDS	predicted inner membrane protein	Integral Membrane Protein	COG3725;Membrane protein required for beta-lactamase induction	GO:0042493 response to drug	o	tt
ampG	CDS	muropeptide transporter	Integral Membrane Protein	COG0477;Permeases of the major facilitator superfamily	GO:0000270 peptidoglycan metabolism	o	tt
ampH	CDS	beta-lactamase/D-alanine carboxypeptidase	Periplasmic	COG1680;Beta-lactamase class C and other penicillin binding proteins	GO:0042493 response to drug		
amtB	CDS	ammonium transporter	Integral Membrane Protein	COG0004;Ammonia permease		o	tt
amyA	CDS	cytoplasmic alpha-amylase	Cytoplasmic	COG0366;Glycosidases	GO:0016052 carbohydrate catabolism -!- GO:0000272 polysaccharide catabolism	o	tt
ansA	CDS	cytoplasmic L-asparaginase I	Cytoplasmic	COG0252;L-asparaginase/archaeal Glu-tRNAGln amidotransferase subunit D	GO:0009063 amino acid catabolism	gg	t
ansB	CDS	periplasmic L-asparaginase II	Periplasmic	COG0252;L-asparaginase/archaeal Glu-tRNAGln amidotransferase subunit D	GO:0009063 amino acid catabolism	g	tt
ansP	CDS	L-asparagine transporter	Integral Membrane Protein		GO:0006529 asparagine		
apaG	CDS	protein associated with Co2+ and Mg2+ efflux	Cytoplasmic	COG2967;Uncharacterized protein affecting Mg2+/Co2+ transport		gg	tt
apaH	CDS	diadenosine tetraphosphatase	Cytoplasmic	COG0639;Diadenosine tetraphosphatase and related serine/threonine protein phosphatases	GO:0015949 nucleobase, nucleoside and nucleotide interconversion	gg	tt
aphA	CDS	acid phosphatase/phosphotransferase, class B, non-specific	Periplasmic	COG3700;Acid phosphatase (class B)	GO:0015949 nucleobase, nucleoside and nucleotide interconversion	o	tt
appA	CDS	phosphoanhydride phosphorylase	Periplasmic		GO:0006793 phosphorus metabolism -!- GO:0042594 response to	o	tt
appB	CDS	cytochrome bd-II oxidase, subunit II	Integral Membrane Protein	COG1294;Cytochrome bd-type quinol oxidase, subunit 2		o	tt
appC	CDS	cytochrome bd-II oxidase, subunit I	Integral Membrane Protein	COG1271;Cytochrome bd-type quinol oxidase, subunit 1		o	tt
appY	CDS	DLP12 prophage; DNA-binding transcriptional activator	Cytoplasmic	COG2207;AraC-type DNA-binding domain-containing proteins	GO:0006350 transcription -!- GO:0042594 response to starvation	o	tt
apt	CDS	adenine phosphoribosyltransferase	Cytoplasmic	COG0503;Adenine/guanine phosphoribosyltransferases and related PRPP-binding proteins	GO:0015949 nucleobase, nucleoside and nucleotide interconversion	gg	o
aqpZ	CDS	aquaporin	Integral Membrane Protein	COG0580;Glycerol uptake facilitator and related permeases (Major Intrinsic Protein Family)	GO:0006970 response to osmotic stress	o	tt
araA	CDS	L-arabinose isomerase	Cytoplasmic	COG2160;L-arabinose isomerase	GO:0016052 carbohydrate	o	tt
araB	CDS	L-ribulokinase	Cytoplasmic	COG1069;Ribulose kinase	GO:0016052 carbohydrate	gg	o
araC	CDS	DNA-binding transcriptional dual regulator	Cytoplasmic	COG2207;AraC-type DNA-binding domain-containing proteins	GO:0016052 carbohydrate catabolism -!- GO:0006350	gg	t
araD	CDS	L-ribulose-5-phosphate 4-epimerase	Cytoplasmic	COG0235;Ribulose-5-phosphate 4-epimerase and related epimerases and aldolases	GO:0016052 carbohydrate catabolism	gg	o
araE	CDS	arabinose transporter	Integral Membrane Protein	COG0477;Permeases of the major facilitator superfamily	GO:0016052 carbohydrate	o	tt
araF	CDS	L-arabinose transporter subunit -!- periplasmic-binding component of ABC superfamily	Periplasmic	COG1879;ABC-type sugar transport system, periplasmic component	GO:0016052 carbohydrate catabolism	o	tt
araG	CDS	fused L-arabinose transporter subunits -!- ATP-binding components of ABC superfamily	Cytoplasmic	COG1129;ABC-type sugar transport system, ATPase component	GO:0016052 carbohydrate catabolism	gg	o
araH	CDS	fused L-arabinose transporter subunits -!- membrane components of ABC superfamily	Integral Membrane Protein		GO:0016052 carbohydrate catabolism	o	tt
araJ	CDS	predicted transporter	Integral Membrane Protein		GO:0016052 carbohydrate	o	tt
arcA	CDS	DNA-binding response regulator in two-component regulatory system with ArcB or CpxA	Cytoplasmic	COG0745;Response regulators consisting of a CheY-like receiver domain and a winged-helix DNA-binding domain	GO:0006350 transcription	o	tt
arcB	CDS	hybrid sensory histidine kinase in two-component regulatory system with ArcA	Integral Membrane Protein	COG0642;Signal transduction histidine kinase -!- COG0784;FOG: CheY-like receiver	GO:0009060 aerobic respiration -!- GO:0006464 protein modification		
argA	CDS	fused acetylglutamate kinase homolog (inactive) -!- amino acid N-acetyltransferase	Cytoplasmic	COG0548;Acetylglutamate kinase -!- COG1246;N-acetylglutamate synthase and related acetyltransferases	GO:0006526 arginine biosynthesis	gg	tt
argB	CDS	acetylglutamate kinase	Cytoplasmic	COG0548;Acetylglutamate kinase	GO:0006526 arginine biosynthesis		
argC	CDS	N-acetyl-gamma-glutamylphosphate reductase, NAD(P)-binding	Cytoplasmic	COG0002;Acetylglutamate semialdehyde dehydrogenase	GO:0006526 arginine biosynthesis	gg	t
argD	CDS	bifunctional acetylornithine aminotransferase -!- succinyldiaminopimelate aminotransferase	Cytoplasmic	COG4992;Ornithine/acetylornithine aminotransferase	GO:0006526 arginine biosynthesis -!- GO:0009089 lysine biosynthesis via diaminopimelate	gg	tt
argE	CDS	acetylornithine deacetylase	Cytoplasmic	COG0624;Acetylornithine deacetylase/Succinyl-diaminopimelate desuccinylase and related deacylases	GO:0006526 arginine biosynthesis	o	tt
argF	CDS	CP4-6 prophage; ornithine carbamoyltransferase 2, chain F	Cytoplasmic	COG0078;Ornithine carbamoyltransferase	GO:0006526 arginine biosynthesis	gg	tt
argG	CDS	argininosuccinate synthetase	Cytoplasmic	COG0137;Argininosuccinate synthase	GO:0006526 arginine biosynthesis	gg	t
argH	CDS	argininosuccinate lyase	Cytoplasmic	COG0165;Argininosuccinate lyase	GO:0006526 arginine biosynthesis	o	tt
argI	CDS	ornithine carbamoyltransferase 1	Cytoplasmic	COG0078;Ornithine carbamoyltransferase	GO:0006526 arginine biosynthesis	gg	o
argK	CDS	membrane ATPase/protein kinase	Cytoplasmic	COG1703;Putative periplasmic protein kinase ArgK and related GTPases of G3E family		gg	t
argO	CDS	arginine transporter	Integral Membrane Protein	COG1279;Lysine efflux permease		gg	tt
argP	CDS	DNA-binding transcriptional activator, replication initiation inhibitor	Cytoplasmic	COG0583;Transcriptional regulator	GO:0006261 DNA dependent DNA replication -!- GO:0006350 transcription	gg	t
argQ	tRNA	tRNA-Arg(ACG) (Arginine tRNA2)					
argR	CDS	DNA-binding transcriptional dual regulator, L-arginine-binding	Cytoplasmic	COG1438;Arginine repressor	GO:0006526 arginine biosynthesis -!- GO:0006350 transcription	o	tt
argS	CDS	arginyl-tRNA synthetase	Cytoplasmic	COG0018;Arginyl-tRNA synthetase	GO:0006418 amino acid activation	o	tt
argT	CDS	lysine/arginine/ornithine transporter subunit -!- periplasmic-binding component of ABC superfamily	Periplasmic	COG0834;ABC-type amino acid transport/signal transduction systems, periplasmic component/domain	GO:0009089 lysine biosynthesis via diaminopimelate -!- GO:0006526 arginine biosynthesis	o	tt
argU	tRNA	tRNA-Arg(UCU) (Arginine tRNA4)					
argV	tRNA	tRNA-Arg(ACG) (Arginine tRNA2)					
argW	tRNA	tRNA-Arg(CCU) (Arginine tRNA5)					
argX	tRNA	tRNA-Arg(CCG) (Arginine tRNA3)					
argY	tRNA	tRNA-Arg(ACG) (Arginine tRNA2)					
argZ	tRNA	tRNA-Arg(ACG) (Arginine tRNA2)					
arnT	CDS	4-amino-4-deoxy-L-arabinose transferase	Integral Membrane Protein	COG1807;4-amino-4-deoxy-L-arabinose transferase and related glycosyltransferases of PMT family	GO:0006950 response to stress	g	tt
aroA	CDS	5-enolpyruvylshikimate-3-phosphate synthetase	Cytoplasmic	COG0128;5-enolpyruvylshikimate-3-phosphate synthase	GO:0009423 chorismate biosynthesis	gg	o
aroB	CDS	3-dehydroquinate synthase	Cytoplasmic	COG0337;3-dehydroquinate synthetase	GO:0009423 chorismate biosynthesis	gg	t
aroC	CDS	chorismate synthase	Cytoplasmic	COG0082;Chorismate synthase	GO:0009423 chorismate biosynthesis	gg	tt
aroD	CDS	3-dehydroquinate dehydratase	Cytoplasmic	COG0710;3-dehydroquinate dehydratase	GO:0009423 chorismate biosynthesis	gg	o
aroE	CDS	dehydroshikimate reductase, NAD(P)-binding	Cytoplasmic	COG0169;Shikimate 5-dehydrogenase	GO:0009423 chorismate biosynthesis	gg	t
aroF	CDS	3-deoxy-D-arabinoheptulosonate-7-phosphate synthase, tyrosine-repressible	Cytoplasmic	COG0722;3-deoxy-D-arabino-heptulosonate 7-phosphate (DAHP) synthase	GO:0009423 chorismate biosynthesis	gg	t
aroG	CDS	3-deoxy-D-arabino-heptulosonate-7-phosphate synthase, phenylalanine repressible	Cytoplasmic	COG0722;3-deoxy-D-arabino-heptulosonate 7-phosphate (DAHP) synthase	GO:0009094 L-phenylalanine biosynthesis	gg	t
aroH	CDS	3-deoxy-D-arabino-heptulosonate-7-phosphate synthase, tryptophan repressible	Cytoplasmic	COG0722;3-deoxy-D-arabino-heptulosonate 7-phosphate (DAHP) synthase	GO:0000162 tryptophan biosynthesis	gg	t
aroK	CDS	shikimate kinase I	Cytoplasmic		GO:0009423 chorismate biosynthesis		
aroL	CDS	shikimate kinase II	Cytoplasmic	COG0703;Shikimate kinase	GO:0009423 chorismate biosynthesis	gg	o
aroM	CDS	conserved protein	Cytoplasmic			o	tt
aroP	CDS	aromatic amino acid transporter	Integral Membrane Protein	COG1113;Gamma-aminobutyrate permease and related permeases	GO:0009094 L-phenylalanine biosynthesis -!- GO:0006571 tyrosine biosynthesis	gg	tt
arpA	CDS	regulator of acetyl CoA synthetase	Cytoplasmic	COG0666;FOG: Ankyrin repeat		gg	o
arpB	CDS	predicted protein, N-ter fragment (pseudogene)	Cytoplasmic				
arpB	ancestral	predicted protein (pseudogene)	Cytoplasmic				
arpB	CDS	predicted protein, C-ter fragment (pseudogene)	Cytoplasmic			g	o
arsB	CDS	arsenite/antimonite transporter	Integral Membrane Protein		GO:0006805 xenobiotic metabolism	o	tt
arsC	CDS	arsenate reductase	Cytoplasmic	COG1393;Arsenate reductase and related proteins, glutaredoxin family	GO:0042493 response to drug	gg	o
arsR	CDS	DNA-binding transcriptional regulator	Cytoplasmic	COG0640;Predicted transcriptional regulators	GO:0006350 transcription -!- GO:0042493 response to drug	o	tt
artI	CDS	arginine transporter subunit -!- periplasmic-binding component of ABC superfamily	Periplasmic	COG0834;ABC-type amino acid transport/signal transduction systems, periplasmic component/domain	GO:0006542 glutamine biosynthesis	o	tt

artJ	CDS	arginine transporter subunit -!- periplasmic-binding component of ABC superfamily	Periplasmic	COG0834;ABC-type amino acid transport/signal transduction systems, periplasmic component/domain	GO:0006526 arginine biosynthesis	o	tt
artM	CDS	arginine transporter subunit -!- membrane component of ABC superfamily	Integral Membrane Protein	COG4160;ABC-type arginine/histidine transport system, permease component	GO:0006526 arginine biosynthesis -!- GO:0009063 amino acid catabolism	o	tt
artP	CDS	arginine transporter subunit -!- ATP-binding component of ABC superfamily	Cytoplasmic	COG4161;ABC-type arginine transport system, ATPase component	GO:0006526 arginine biosynthesis	o	t
artQ	CDS	arginine transporter subunit -!-membrane component of ABC superfamily	Integral Membrane Protein	COG4215;ABC-type arginine transport system, permease component	GO:0006526 arginine biosynthesis	o	tt
ascB	CDS	cryptic 6-phospho-beta-glucosidase	Cytoplasmic	COG2723;Beta-glucosidase/6-phospho-beta-glucosidas e/beta-galactosidase	GO:0016052 carbohydrate catabolism	gg	t
ascF	CDS	fused cellobiose/arbutin/salicin-specific enzyme IIBC component of PTS	Integral Membrane Protein	COG1264;Phosphotransferase system IIB components -!- COG1263;Phosphotransferase system IIC components, glucose/maltose/N-acetylglucosamine-specific	GO:0016052 carbohydrate catabolism		
ascG	CDS	DNA-binding transcriptional regulator	Cytoplasmic	COG1609;Transcriptional regulators	GO:0016052 carbohydrate catabolism -!- GO:0006350		
asd	CDS	aspartate-semialdehyde dehydrogenase, NAD(P)-binding	Cytoplasmic	COG0136;Aspartate-semialdehyde dehydrogenase	GO:0009089 lysine biosynthesis via diaminopimelate -!- GO:0009088 threonine biosynthesis -!- GO:0009086 methionine biosynthesis -!- GO:0009090 homoserine biosynthesis	g	t
aslA	CDS	acrylsulfatase-like enzyme	Periplasmic	COG3119;Arylsulfatase A and related enzymes	GO:0006790 sulfur metabolism	o	tt
aslB	CDS	predicted regulator of arylsulfatase activity	Cytoplasmic		GO:0006790 sulfur metabolism -!- GO:0006457 protein folding		
asmA	CDS	predicted assembly protein	Membrane Anchored	COG2982;Uncharacterized protein involved in outer membrane biogenesis	GO:0009103 lipopolysaccharide biosynthesis	o	tt
asnA	CDS	asparagine synthetase A	Cytoplasmic	COG2502;Asparagine synthetase A	GO:0009063 amino acid catabolism -!- GO:0006529 asparagine biosynthesis	gg	o
asnB	CDS	asparagine synthetase B	Cytoplasmic	COG0367;Asparagine synthase (glutamine-hydrolyzing)	GO:0009063 amino acid catabolism -!- GO:0006529 asparagine biosynthesis	gg	t
asnC	CDS	DNA-binding transcriptional dual regulator	Cytoplasmic	COG1522;Transcriptional regulators	GO:0006529 asparagine biosynthesis -!- GO:0006350	gg	t
asnS	CDS	asparaginyl tRNA synthetase	Cytoplasmic	COG0017;Aspartyl/asparaginyl-tRNA synthetases	GO:0006418 amino acid activation	gg	t
asnT	tRNA	tRNA-Asn(GUU) (Asparagine tRNA)					
asnU	tRNA	tRNA-Asn(GUU) (Asparagine tRNA)					
asnV	tRNA	tRNA-Asn(GUU) (Asparagine tRNA)					
asnW	tRNA	tRNA-Asn(GUU) (Asparagine tRNA)					
aspA	CDS	aspartate ammonia-lyase	Cytoplasmic		GO:0006520 amino acid metabolism -!- GO:0006529 asparagine biosynthesis -!- GO:0009089 lysine biosynthesis via diaminopimelate	o	tt
aspC	CDS	aspartate aminotransferase, PLP-dependent	Cytoplasmic	COG1448;Aspartate/tyrosine/aromatic aminotransferase	GO:0009063 amino acid catabolism -!- GO:0006532 aspartate	gg	o
aspS	CDS	aspartyl-tRNA synthetase	Cytoplasmic	COG0173;Aspartyl-tRNA synthetase	GO:0006418 amino acid activation	gg	t
aspT	tRNA	tRNA-Asp(GUC) (Aspartate tRNA1)					
aspU	tRNA	tRNA-Asp(GUC) (Aspartate tRNA1)					
aspV	tRNA	tRNA-Asp(GUC) (Aspartate tRNA1)					
asr	CDS	acid shock-inducible periplasmic protein	Periplasmic		GO:0009268 response to pH		
astA	CDS	arginine succinyltransferase	Cytoplasmic	COG3138;Arginine/ornithine N-succinyltransferase beta subunit	GO:0009063 amino acid catabolism	gg	t
astB	CDS	succinylarginine dihydrolase	Cytoplasmic	COG3724;Succinylarginine dihydrolase	GO:0009063 amino acid catabolism	o	t
astC	CDS	succinylornithine transaminase, PLP-dependent	Cytoplasmic	COG4992;Ornithine/acetylornithine aminotransferase	GO:0009063 amino acid catabolism	gg	o
astD	CDS	succinylglutamic semialdehyde dehydrogenase	Cytoplasmic	COG1012;NAD-dependent aldehyde dehydrogenases	GO:0009063 amino acid catabolism		
astE	CDS	succinylglutamate desuccinylase	Cytoplasmic	COG2988;Succinylglutamate desuccinylase	GO:0009063 amino acid catabolism	gg	o
atoA	CDS	acetyl-CoA:acetoacetyl-CoA transferase, beta	Cytoplasmic	COG2057;Acyl CoA:acetate/3-ketoacid CoA transferase, beta subunit	GO:0019395 fatty acid oxidation	gg	o
atoB	CDS	acetyl-CoA acetyltransferase	Cytoplasmic	COG0183;Acetyl-CoA acetyltransferase	GO:0019395 fatty acid oxidation	o	tt
atoC	CDS	fused response regulator of ato opeon, in two-component system with AtoS	Cytoplasmic	COG2204;Response regulator containing CheY-like receiver, AAA-type ATPase, and DNA-binding domains	GO:0019395 fatty acid oxidation -!- GO:0006596 polyamine biosynthesis -!- GO:0006350 transcription	gg	t
atoD	CDS	acetyl-CoA:acetoacetyl-CoA transferase, alpha	Cytoplasmic	COG1788;Acyl CoA:acetate/3-ketoacid CoA transferase, alpha subunit	GO:0019395 fatty acid oxidation	gg	t
atoE	CDS	short chain fatty acid transporter	Integral Membrane Protein	COG2031;Short chain fatty acids transporter	GO:0019395 fatty acid oxidation	o	tt
atoS	CDS	sensory histidine kinase in two-component regulatory system with AtoC	Integral Membrane Protein	COG1012;NAD-dependent aldehyde dehydrogenases -!- COG0642;Signal transduction histidine kinase	GO:0019395 fatty acid oxidation -!- GO:0006464 protein modification	o	tt
atpA	CDS	F1 sector of membrane-bound ATP synthase, alpha subunit	Cytoplasmic	COG0056;F0F1-type ATP synthase, alpha subunit	GO:0015986 ATP synthesis coupled proton transport	gg	o
atpB	CDS	F0 sector of membrane-bound ATP synthase, subunit a	Integral Membrane Protein	COG0356;F0F1-type ATP synthase, subunit a	GO:0015986 ATP synthesis coupled proton transport -!- GO:0006457 protein folding	o	tt
atpC	CDS	F1 sector of membrane-bound ATP synthase, epsilon subunit	Cytoplasmic	COG0355;F0F1-type ATP synthase, epsilon subunit (mitochondrial delta subunit)	GO:0015986 ATP synthesis coupled proton transport	gg	t
atpD	CDS	F1 sector of membrane-bound ATP synthase, beta subunit	Cytoplasmic	COG0055;F0F1-type ATP synthase, beta subunit	GO:0015986 ATP synthesis coupled proton transport	g	t
atpE	CDS	F0 sector of membrane-bound ATP synthase, subunit c	Integral Membrane Protein	COG0636;F0F1-type ATP synthase, subunit c/Archaeal/vacuolar-type H+-ATPase, subunit K	GO:0015986 ATP synthesis coupled proton transport	o	tt
atpF	CDS	F0 sector of membrane-bound ATP synthase, subunit b	Membrane Anchored	COG0711;F0F1-type ATP synthase, subunit b	GO:0015986 ATP synthesis coupled proton transport	o	tt
atpG	CDS	F1 sector of membrane-bound ATP synthase, gamma subunit	Cytoplasmic	COG0224;F0F1-type ATP synthase, gamma subunit	GO:0015986 ATP synthesis coupled proton transport	gg	tt
atpH	CDS	F1 sector of membrane-bound ATP synthase, delta subunit	Cytoplasmic	COG0712;F0F1-type ATP synthase, delta subunit (mitochondrial oligomycin sensitivity protein)	GO:0015986 ATP synthesis coupled proton transport	o	o
atpI	CDS	ATP synthase, membrane-bound accesory subunit	Integral Membrane Protein	COG3312;F0F1-type ATP synthase, subunit I	GO:0015986 ATP synthesis coupled proton transport		
avtA	CDS	valine-pyruvate aminotransferase	Cytoplasmic		GO:0006523 alanine biosynthesis		
azoR	CDS	NADH-azoreductase, FMN-dependent	Cytoplasmic	COG1182;Acyl carrier protein phosphodiesterase		gg	o
bacA	CDS	undecaprenyl pyrophosphate phosphatase	Integral Membrane Protein	COG1968;Uncharacterized bacitracin resistance protein	GO:0042493 response to drug	o	tt
baeR	CDS	DNA-binding response regulator in two-component regulatory system with BaeS	Cytoplasmic	COG0745;Response regulators consisting of a CheY-like receiver domain and a winged-helix DNA-binding domain	GO:0006350 transcription	gg	o
baeS	CDS	sensory histidine kinase in two-component regulatory system with BaeR	Integral Membrane Protein	COG0642;Signal transduction histidine kinase	GO:0006464 protein modification	o	tt
barA	CDS	hybrid sensory histidine kinase, in two-component regulatory system with UvrY	Integral Membrane Protein	COG0642;Signal transduction histidine kinase -!- COG4999;Uncharacterized domain of BarA-like signal transduction histidine kinases -!- COG2201;Chemotaxis response regulator containing a CheY-like receiver domain and a methylesterase domain -!-	GO:0006464 protein modification	o	tt
basR	CDS	DNA-binding response regulator in two-component regulatory system with BasS	Cytoplasmic	COG0745;Response regulators consisting of a CheY-like receiver domain and a winged-helix DNA-binding domain	GO:0006350 transcription	o	tt
basS	CDS	sensory histidine kinase in two-component regulatory system with BasR	Integral Membrane Protein	COG0642;Signal transduction histidine kinase	GO:0006464 protein modification	o	tt
bax	CDS	conserved protein	Periplasmic				
bcp	CDS	thiol peroxidase, thioredoxin-dependent	Cytoplasmic	COG1225;Peroxiredoxin	GO:0006805 xenobiotic metabolism	gg	t
bcr	CDS	bicyclomycin/multidrug efflux system	Integral Membrane Protein	COG0477;Permeases of the major facilitator superfamily	GO:0042493 response to drug		
bcsA	CDS	cellulose synthase, catalytic subunit	Integral Membrane Protein				
bcsB	CDS	regulator of cellulose synthase, cyclic di-GMP binding	Integral Membrane Protein			o	tt
bcsC	CDS	cellulose synthase subunit	Periplasmic				
bcsE	CDS	conserved protein	Cytoplasmic			gg	t
bcsF	CDS	predicted protein	Membrane Anchored				
bcsG	CDS	predicted inner membrane protein	Integral Membrane Protein			o	tt
bcsZ	CDS	endo-1,4-D-glucanase	Outer Membrane Lipoprotein	COG3405;Endoglucanase Y	GO:0016052 carbohydrate catabolism -!- GO:0000272 polysaccharide catabolism	o	tt
bdm	CDS	biofilm-dependent modulation protein	Cytoplasmic				
betA	CDS	choline dehydrogenase, a flavoprotein	Cytoplasmic	COG2303;Choline dehydrogenase and related flavoproteins	GO:0006578 betaine biosynthesis -!- GO:0006970 response to osmotic stress	gg	t
betB	CDS	betaine aldehyde dehydrogenase, NAD-dependent	Cytoplasmic	COG1012;NAD-dependent aldehyde dehydrogenases	GO:0006578 betaine biosynthesis -!- GO:0006970 response to osmotic stress	gg	t
betI	CDS	DNA-binding transcriptional repressor	Cytoplasmic		GO:0006350 transcription -!- GO:0006970 response to osmotic stress	o	o
betT	CDS	choline transporter of high affinity	Integral Membrane Protein	COG1292;Choline-glycine betaine transporter	GO:0006578 betaine biosynthesis	o	tt
bfd	CDS	bacterioferritin-associated ferredoxin	Cytoplasmic	COG2906;Bacterioferritin-associated ferredoxin	GO:0006826 iron ion transport	gg	t
bfr	CDS	bacterioferritin, iron storage and detoxification protein	Cytoplasmic	COG2193;Bacterioferritin (cytochrome b1)	GO:0006826 iron ion transport	gg	tt
bglA	CDS	6-phospho-beta-glucosidase A	Cytoplasmic	COG2723;Beta-glucosidase/6-phospho-beta-glucosidas e/beta-galactosidase	GO:0016052 carbohydrate catabolism	gg	o
bglB	CDS	cryptic phospho-beta-glucosidase B	Cytoplasmic	COG2723;Beta-glucosidase/6-phospho-beta-glucosidas e/beta-galactosidase	GO:0016052 carbohydrate catabolism	gg	t
bglF	CDS	fused beta-glucoside-specific PTS enzyme IIABC components	Integral Membrane Protein	COG1264;Phosphotransferase system IIB components -!- COG1263;Phosphotransferase system IIC components, glucose/maltose/N-acetylglucosamine-specific -!- COG2190;Phosphotransferase system IIA components	GO:0016052 carbohydrate catabolism -!- GO:0006464 protein modification	o	tt
bglG	CDS	transcriptional antiterminator of the bgl operon	Cytoplasmic	COG3711;Transcriptional antiterminator	GO:0016052 carbohydrate catabolism -!- GO:0006350	gg	t
bglH	CDS	carbohydrate-specific outer membrane porin, cryptic	Outer Membrane B-barrel protein	COG4580;Maltoporin (phage lambda and maltose receptor)		o	tt
bglJ	CDS	DNA-binding transcriptional regulator	Cytoplasmic	COG2197;Response regulator containing a CheY-like receiver domain and an HTH DNA-binding domain	GO:0016052 carbohydrate catabolism -!- GO:0006350		
bglX	CDS	beta-D-glucoside glucohydrolase, periplasmic	Periplasmic	COG1472;Beta-glucosidase-related glycosidases	GO:0016052 carbohydrate	g	tt
bioA	CDS	7,8-diaminopelargonic acid synthase, PLP-	Cytoplasmic	COG0161;Adenosylmethionine-8-amino-7-oxononanoate aminotransferase	GO:0009102 biotin biosynthesis	gg	t
bioB	CDS	biotin synthase	Cytoplasmic	COG0502;Biotin synthase and related enzymes	GO:0009102 biotin biosynthesis	gg	t
bioC	CDS	predicted methltransferase, enzyme of biotin	Cytoplasmic	COG0500;SAM-dependent methyltransferases	GO:0009102 biotin biosynthesis	g	t

bioD	CDS	dethiobiotin synthetase	Cytoplasmic	COG0132;Dethiobiotin synthetase	GO:0009102 biotin biosynthesis	g	t
bioF	CDS	8-amino-7-oxononanoate synthase	Cytoplasmic	COG0156;7-keto-8-aminopelargonate synthetase and related enzymes	GO:0009102 biotin biosynthesis	g	t
bioH	CDS	carboxylesterase of pimeloyl-CoA synthesis	Cytoplasmic	COG0596;Predicted hydrolases or acyltransferases (alpha/beta hydrolase superfamily)	GO:0009102 biotin biosynthesis	gg	tt
bipA	CDS	GTP-binding protein	Cytoplasmic				
birA	CDS	bifunctional biotin-[acetylCoA carboxylase] holoenzyme synthetase -!- DNA-binding transcriptional repressor, bio-5'-AMP-binding	Cytoplasmic	COG1654;Biotin operon repressor -!- COG0340;Biotin-(acetyl-CoA carboxylase) ligase	GO:0009102 biotin biosynthesis -!- GO:0006350 transcription	gg	t
bisC	CDS	biotin sulfoxide reductase	Cytoplasmic	COG0243;Anaerobic dehydrogenases, typically selenocysteine-containing	GO:0009102 biotin biosynthesis		
blc	CDS	outer membrane lipoprotein (lipocalin)	Outer Membrane Lipoprotein	COG3040;Bacterial lipocalin		o	tt
blr	CDS	beta-lactam resistance membrane protein	Integral Membrane Protein				
bolA	CDS	regulator of penicillin binding proteins and beta lactamase transcription (morphogene)	Cytoplasmic		GO:0006970 response to osmotic stress		
borD	CDS	DLP12 prophage; predicted lipoprotein	Outer Membrane Lipoprotein			o	tt
brnQ	CDS	predicted branched chain amino acid transporter (LIV-II)	Integral Membrane Protein	COG1114;Branched-chain amino acid permeases	GO:0009098 leucine biosynthesis -!- GO:0009063 amino acid catabolism	o	tt
btuB	CDS	vitamin B12/cobalamin outer membrane transporter	Outer Membrane B-barrel protein	COG4206;Outer membrane cobalamin receptor protein			
btuC	CDS	vitamin B12 transporter subunit -!- membrane component of ABC superfamily	Integral Membrane Protein	COG4139;ABC-type cobalamin transport system, permease component	GO:0009236 vitamin B12 biosynthesis	o	tt
btuD	CDS	vitamin B12 transporter subunit -!- ATP-binding component of ABC superfamily	Cytoplasmic	COG4138;ABC-type cobalamin transport system, ATPase component	GO:0009236 vitamin B12 biosynthesis	g	t
btuE	CDS	predicted glutathione peroxidase	Cytoplasmic	COG0386;Glutathione peroxidase	GO:0009236 vitamin B12	gg	t
btuF	CDS	vitamin B12 transporter subunit -!- periplasmic-binding component of ABC superfamily	Periplasmic	COG0614;ABC-type Fe3+-hydroxamate transport system, periplasmic component		o	tt
btuR	CDS	cob(I)alamin adenolsyltransferase/cobinamide ATP-dependent adenolsyltransferase	Cytoplasmic	COG2109;ATP:corrinoid adenosyltransferase	GO:0009236 vitamin B12 biosynthesis	gg	t
cadA	CDS	lysine decarboxylase 1	Cytoplasmic	COG1982;Arginine/lysine/ornithine decarboxylases	GO:0009063 amino acid catabolism -!- GO:0009089 lysine biosynthesis via diaminopimelate	g	t
cadB	CDS	predicted lysine/cadaverine transporter	Integral Membrane Protein	COG0531;Amino acid transporters	GO:0009089 lysine biosynthesis via diaminopimelate	o	tt
cadC	CDS	DNA-binding transcriptional activator	Membrane Anchored	COG3710;DNA-binding winged-HTH domains	GO:0009063 amino acid catabolism -!- GO:0006350 transcription	o	tt
caiA	CDS	crotonobetaine reductase subunit II, FAD-binding	Cytoplasmic	COG1960;Acyl-CoA dehydrogenases	GO:0006097 glyoxylate cycle -!- GO:0009310 amine catabolism -!- GO:0042413 carnitine catabolism	gg	t
caiB	CDS	crotonobetainyl CoA:carnitine CoA transferase	Cytoplasmic	COG1804;Predicted acyl-CoA transferases/carnitine dehydratase	GO:0042413 carnitine catabolism -!- GO:0009310 amine catabolism	gg	o
caiC	CDS	predicted crotonobetaine CoA ligase:carnitine CoA ligase	Cytoplasmic		GO:0009310 amine catabolism	gg	o
caiD	CDS	crotonobetainyl CoA hydratase	Cytoplasmic	COG1024;Enoyl-CoA hydratase/carnithine racemase	GO:0009310 amine catabolism -!- GO:0042413 carnitine catabolism	o	tt
caiE	CDS	predicted acyl transferase	Cytoplasmic	COG0663;Carbonic anhydrases/acetyltransferases, isoleucine patch superfamily	GO:0009310 amine catabolism -!- GO:0042413 carnitine catabolism		
caiF	CDS	DNA-binding transcriptional activator	Cytoplasmic		GO:0009310 amine catabolism -!- GO:0006350 transcription	gg	o
caiT	CDS	predicted transporter	Integral Membrane Protein	COG1292;Choline-glycine betaine transporter	GO:0016052 carbohydrate	o	tt
can	CDS	carbonic anhydrase	Cytoplasmic	COG0288;Carbonic anhydrase		o	t
carA	CDS	carbamoyl phosphate synthetase small subunit, glutamine amidotransferase	Cytoplasmic	COG0505;Carbamoylphosphate synthase small subunit	GO:0006526 arginine biosynthesis -!- GO:0006221 pyrimidine nucleotide biosynthesis	gg	o
carB	CDS	carbamoyl-phosphate synthase large subunit	Cytoplasmic	COG0458;Carbamoylphosphate synthase large subunit (split gene in MJ)	GO:0006526 arginine biosynthesis -!- GO:0006221 pyrimidine nucleotide biosynthesis	o	tt
cbl	CDS	DNA-binding transcriptional activator	Cytoplasmic	COG0583;Transcriptional regulator	GO:0019344 cysteine biosynthesis -!- GO:0006790 sulfur metabolism -!- GO:0006350 transcription	gg	t
cbpA	CDS	curved DNA-binding protein, DnaJ homologue that functions as a co-chaperone of DnaK	Cytoplasmic	COG2214;DnaJ-class molecular chaperone	GO:0006457 protein folding	gg	t
cbpM	CDS	modulator of CbpA co-chaperone	Cytoplasmic			gg	o
cbrA	CDS	predicted oxidoreductase, FAD/NAD(P)-binding domain	Cytoplasmic	COG0644;Dehydrogenases (flavoproteins)			
cbrC	CDS	conserved protein	Cytoplasmic	COG3196;Uncharacterized protein conserved in bacteria		gg	tt
cca	CDS	fused tRNA nucleotidyl transferase -!- 2'3'-cyclic phosphodiesterase/2'nucleotidase/phosphatase	Cytoplasmic	COG0617;tRNA nucleotidyltransferase/poly(A) polymerase	GO:0009451 RNA modification	g	t
cchA	CDS	predicted carboxysome structural protein, ethanolamine utilization protein	Cytoplasmic	COG4577;Carbon dioxide concentrating mechanism/carboxysome shell protein	GO:0006805 xenobiotic metabolism	gg	o
cchB	CDS	predicted carboxysome structural protein, ethanolamine utilization protein	Cytoplasmic	COG4576;Carbon dioxide concentrating mechanism/carboxysome shell protein	GO:0006805 xenobiotic metabolism	gg	o
ccmA	CDS	heme exporter subunit -!- ATP-binding component of ABC superfamily	Cytoplasmic	COG4133;ABC-type transport system involved in cytochrome c biogenesis, ATPase component	GO:0017004 cytochrome biogenesis -!- GO:0006457 protein folding		
ccmB	CDS	heme exporter subunit -!- membrane component of ABC superfamily	Integral Membrane Protein	COG2386;ABC-type transport system involved in cytochrome c biogenesis, permease component	GO:0017004 cytochrome biogenesis -!- GO:0006457 protein folding	o	tt
ccmC	CDS	heme exporter subunit -!- membrane component of ABC superfamily	Integral Membrane Protein	COG0755;ABC-type transport system involved in cytochrome c biogenesis, permease component	GO:0017004 cytochrome biogenesis -!- GO:0006457 protein folding	o	tt
ccmD	CDS	cytochrome c biogenesis protein	Membrane Anchored	COG3114;Heme exporter protein D	GO:0017004 cytochrome biogenesis -!- GO:0006457 protein folding	o	tt
ccmE	CDS	periplasmic heme chaperone	Periplasmic	COG2332;Cytochrome c-type biogenesis protein CcmE	GO:0017004 cytochrome biogenesis -!- GO:0006457 protein folding	o	tt
ccmF	CDS	heme lyase, CcmF subunit	Integral Membrane Protein	COG1138;Cytochrome c biogenesis factor	GO:0017004 cytochrome biogenesis	o	tt
ccmG	CDS	periplasmic thioredoxin of cytochrome c-type biogenesis	Membrane Anchored	COG0526;Thiol-disulfide isomerase and thioredoxins	GO:0017004 cytochrome biogenesis -!- GO:0006457 protein folding	o	tt
ccmH	CDS	heme lyase, CcmH subunit	Integral Membrane Protein	COG3088;Uncharacterized protein involved in biosynthesis of c-type cytochromes -!- COG4235;Cytochrome c biogenesis factor	GO:0017004 cytochrome biogenesis -!- GO:0006457 protein folding	o	tt
cdaR	CDS	DNA-binding transcriptional regulator	Cytoplasmic				
cdd	CDS	cytidine/deoxycytidine deaminase	Cytoplasmic	COG0295;Cytidine deaminase	GO:0015949 nucleobase, nucleoside and nucleotide interconversion	g	t
cdh	CDS	CDP-diacylglycerol phosphotidylhydrolase	Membrane Anchored	COG2134;CDP-diacylglycerol pyrophosphatase		o	tt
cdsA	CDS	CDP-diglyceride synthase	Integral Membrane Protein	COG0575;CDP-diglyceride synthetase	GO:0008654 phospholipid biosynthesis		
cedA	CDS	cell division modulator	Cytoplasmic			gg	o
cfa	CDS	cyclopropane fatty acyl phospholipid synthase (unsaturated-phospholipid methyltransferase)	Cytoplasmic	COG2230;Cyclopropane fatty acid synthase and related methyltransferases		gg	t
chaA	CDS	calcium/sodium:proton antiporter	Integral Membrane Protein	COG0387;Ca2+/H+ antiporter		o	tt
chaB	CDS	predicted cation regulator	Cytoplasmic	COG4572;Putative cation transport regulator	GO:0006350 transcription	gg	o
chaC	CDS	regulatory protein for cation transport	Cytoplasmic	COG3703;Uncharacterized protein involved in cation transport	GO:0006350 transcription	gg	o
chbA	CDS	N,N'-diacetylchitobiose-specific enzyme IIA component of PTS	Cytoplasmic	COG1447;Phosphotransferase system cellobiose-specific component IIA	GO:0016052 carbohydrate catabolism	o	o
chbB	CDS	N,N'-diacetylchitobiose-specific enzyme IIB component of PTS	Periplasmic	COG1440;Phosphotransferase system cellobiose-specific component IIB	GO:0016052 carbohydrate catabolism	gg	o
chbC	CDS	N,N'-diacetylchitobiose-specific enzyme IIC component of PTS	Integral Membrane Protein	COG1455;Phosphotransferase system cellobiose-specific component IIC	GO:0016052 carbohydrate catabolism	gg	tt
chbF	CDS	cryptic phospho-beta-glucosidase, NAD(P)-binding	Cytoplasmic	COG1486;Alpha-galactosidases/6-phospho-beta-glucos idases, family 4 of glycosyl hydrolases	GO:0016052 carbohydrate catabolism -!- GO:0009435 nicotinamide adenine dinucleotide	gg	t
chbG	CDS	conserved protein	Cytoplasmic	COG3394;Uncharacterized protein conserved in bacteria		o	tt
chbR	CDS	DNA-binding transcriptional dual regulator	Cytoplasmic	COG2207;AraC-type DNA-binding domain-containing proteins	GO:0016052 carbohydrate catabolism -!- GO:0006350	o	tt
cheA	CDS	fused chemotactic sensory histidine kinase (soluble) in two-component regulatory system with CheB and CheY	Cytoplasmic	COG0643;Chemotaxis protein histidine kinase and related kinases	GO:0006464 protein modification -!- GO:0042330 taxis	gg	tt
cheB	CDS	fused chemotaxis regulator -!- protein-glutamate methylesterase, in two-component regulatory system with CheA	Cytoplasmic	COG2201;Chemotaxis response regulator containing a CheY-like receiver domain and a methylesterase domain	GO:0006464 protein modification -!- GO:0042330 taxis	gg	t
cheR	CDS	chemotaxis regulator	Cytoplasmic	COG1352;Methylase of chemotaxis methyl-accepting proteins	GO:0006464 protein modification -!- GO:0042330 taxis	g	t
cheW	CDS	purine-binding chemotaxis protein	Cytoplasmic	COG0835;Chemotaxis signal transduction protein	GO:0042330 taxis	gg	t
cheY	CDS	chemotaxis regulator transmitting signal to flagellar motor component	Cytoplasmic	COG0784;FOG: CheY-like receiver	GO:0042330 taxis	gg	o
cheZ	CDS	chemotaxis regulator	Cytoplasmic	COG3143;Chemotaxis protein	GO:0006464 protein modification -!- GO:0042330 taxis	gg	tt
chiA	CDS	periplasmic endochitinase	Periplasmic		GO:0009057 macromolecule catabolism	g	tt
chpA	CDS	toxin of the ChpA-ChpR toxin-antitoxin system, endoribonuclease	Cytoplasmic	COG2337;Growth inhibitor		o	tt
chpB	CDS	toxin of the ChpB-ChpS toxin-antitoxin system	Cytoplasmic	COG2337;Growth inhibitor		o	tt
chpR	CDS	antitoxin of the ChpA-ChpR toxin-antitoxin system	Cytoplasmic	COG2336;Growth regulator		gg	tt
chpS	CDS	antitoxin of the ChpB-ChpS toxin-antitoxin system	Cytoplasmic	COG2336;Growth regulator			
cirA	CDS	ferric iron-catecholate outer membrane transporter	Outer Membrane B-barrel protein	COG4771;Outer membrane receptor for ferrienterochelin and colicins		g	t
citA	CDS	sensory histidine kinase in two-component regulatory system with citB	Integral Membrane Protein	COG3290;Signal transduction histidine kinase regulating citrate/malate metabolism	GO:0006464 protein modification	o	tt
citB	CDS	DNA-binding response regulator in two-component regulatory system with citA	Cytoplasmic	COG4565;Response regulator of citrate/malate metabolism	GO:0006350 transcription	o	tt
citC	CDS	citrate lyase synthetase	Cytoplasmic		GO:0006464 protein modification	gg	t
citD	CDS	citrate lyase, acyl carrier (gamma) subunit	Cytoplasmic	COG3052;Citrate lyase, gamma subunit	GO:0042967 acyl-carrier protein biosynthesis	gg	t
citE	CDS	citrate lyase, citryl-ACP lyase (beta) subunit	Cytoplasmic			g	t
citF	CDS	citrate lyase, citrate-ACP transferase (alpha) subunit	Cytoplasmic	COG3051;Citrate lyase, alpha subunit			

citG	CDS	triphosphoribosyl-dephospho-CoA transferase	Cytoplasmic	COG1767;Triphosphoribosyl-dephospho-CoA synthetase	GO:0006412 protein biosynthesis	g	t
citT	CDS	citrate:succinate antiporter	Integral Membrane Protein	COG0471;Di- and tricarboxylate transporters	GO:0016052 carbohydrate	o	tt
citX	CDS	apo-citrate lyase phosphoribosyl-dephospho-CoA transferase	Cytoplasmic	COG3697;Phosphoribosyl-dephospho-CoA transferase (holo-ACP synthetase)	GO:0006464 protein modification	gg	t
clcA	CDS	chloride channel, voltage-gated	Integral Membrane Protein	COG0038;Chloride channel protein EriC			
clcB	CDS	predicted voltage-gated chloride channel	Integral Membrane Protein	COG0038;Chloride channel protein EriC			
cld	CDS	regulator of length of O-antigen component of lipopolysaccharide chains	Integral Membrane Protein		GO:0009243 O antigen biosynthesis		
clpA	CDS	ATPase and specificity subunit of ClpA-ClpP ATP-dependent serine protease, chaperone activity	Cytoplasmic	COG0542;ATPases with chaperone activity, ATP-binding subunit		gg	tt
clpB	CDS	protein disaggregation chaperone	Cytoplasmic	COG0542;ATPases with chaperone activity, ATP-binding subunit	GO:0006457 protein folding	gg	tt
clpP	CDS	proteolytic subunit of ClpA-ClpP and ClpX-ClpP ATP-dependent serine proteases	Cytoplasmic	COG0740;Protease subunit of ATP-dependent Clp proteases	GO:0009266 response to temperature	gg	o
clpS	CDS	regulatory protein for ClpA substrate specificity	Cytoplasmic	COG2127;Uncharacterized conserved protein		gg	o
clpX	CDS	ATPase and specificity subunit of ClpX-ClpP ATP-dependent serine protease	Cytoplasmic	COG1219;ATP-dependent protease Clp, ATPase subunit	GO:0006457 protein folding	o	tt
cls	CDS	cardiolipin synthase 1	Integral Membrane Protein	COG1502;Phosphatidylserine/phosphatidylglycerophos phate/cardiolipin synthases and related enzymes	GO:0008654 phospholipid biosynthesis -!- GO:0042493 response to drug	o	tt
cmk	CDS	cytidylate kinase	Cytoplasmic	COG0283;Cytidylate kinase	GO:0015949 nucleobase, nucleoside and nucleotide interconversion	gg	o
cmr	CDS	multidrug efflux system protein	Integral Membrane Protein	COG0477;Permeases of the major facilitator superfamily	GO:0042493 response to drug	o	tt
cmtA	CDS	predicted fused mannitol-specific enzyme IIBC component of PTS	Integral Membrane Protein	COG2213;Phosphotransferase system, mannitol-specific IIBC component	GO:0016052 carbohydrate catabolism	o	tt
cmtB	CDS	predicted mannitol-specific enzyme IIA component of PTS	Cytoplasmic	COG1762;Phosphotransferase system mannitol/fructose-specific IIA domain (Ntr-type)	GO:0016052 carbohydrate catabolism	gg	o
coaA	CDS	pantothenate kinase	Cytoplasmic	COG1072;Panthothenate kinase	GO:0015937 coenzyme A biosynthesis	gg	tt
coaD	CDS	pantetheine-phosphate adenylyltransferase	Cytoplasmic	COG0669;Phosphopantetheine adenylyltransferase		gg	o
coaE	CDS	dephospho-CoA kinase	Periplasmic	COG0237;Dephospho-CoA kinase		gg	t
cobB	CDS	deacetylase of acetyl-CoA synthetase, NAD-dependent	Cytoplasmic	COG0846;NAD-dependent protein deacetylases, SIR2 family	GO:0006355 regulation of transcription, DNA-dependent	o	tt
cobC	CDS	predicted alpha-ribazole-5'-P phosphatase	Cytoplasmic	COG0406;Fructose-2,6-bisphosphatase	GO:0009236 vitamin B12	gg	t
cobS	CDS	cobalamin 5'-phosphate synthase	Integral Membrane Protein	COG0368;Cobalamin-5-phosphate synthase	GO:0009236 vitamin B12	o	tt
cobT	CDS	nicotinate-nucleotide dimethylbenzimidazole-P phophoribosyl transferase	Cytoplasmic	COG2038;NaMN:DMB phosphoribosyltransferase	GO:0009236 vitamin B12 biosynthesis	gg	o
cobU	CDS	bifunctional cobinamide kinase -!- cobinamide phosphate guanylyltransferase	Cytoplasmic	COG2087;Adenosyl cobinamide kinase/adenosyl cobinamide phosphate guanylyltransferase	GO:0009236 vitamin B12 biosynthesis	g	t
codA	CDS	cytosine deaminase	Cytoplasmic	COG0402;Cytosine deaminase and related metal-dependent hydrolases	GO:0015949 nucleobase, nucleoside and nucleotide interconversion	o	tt
codB	CDS	cytosine transporter	Integral Membrane Protein	COG1457;Purine-cytosine permease and related proteins	GO:0015949 nucleobase, nucleoside and nucleotide interconversion	o	tt
cof	CDS	thiamin pyrimidine pyrophosphate hydrolase	Cytoplasmic	COG0561;Predicted hydrolases of the HAD superfamily		gg	o
copA	CDS	copper transporter	Integral Membrane Protein	COG2217;Cation transport ATPase		o	tt
corA	CDS	magnesium/nickel/cobalt transporter	Integral Membrane Protein	COG0598;Mg2+ and Co2+ transporters		o	tt
cpdA	CDS	cyclic 3',5'-adenosine monophosphate phosphodiesterase	Cytoplasmic	COG1409;Predicted phosphohydrolases	GO:0016052 carbohydrate catabolism	gg	t
cpdB	CDS	2':3'-cyclic-nucleotide 2'-phosphodiesterase	Periplasmic	COG0737;5'-nucleotidase/2',3'-cyclic phosphodiesterase and related esterases	GO:0015949 nucleobase, nucleoside and nucleotide interconversion	o	tt
cpsB	CDS	mannose-1-phosphate guanyltransferase	Cytoplasmic	COG0836;Mannose-1-phosphate guanylyltransferase -!- COG0662;Mannose-6-phosphate isomerase	GO:0009242 colanic acid biosynthesis -!- GO:0016052 carbohydrate catabolism -!- GO:0009226 nucleotide-sugar biosynthesis -!- GO:0009269	gg	t
cpsG	CDS	phosphomannomutase	Cytoplasmic	COG1109;Phosphomannomutase	GO:0009226 nucleotide-sugar biosynthesis -!- GO:0009242 colanic acid biosynthesis -!- GO:0009269 response to dessication	gg	t
cpxA	CDS	sensory histidine kinase in two-component regulatory system with CpxR	Integral Membrane Protein	COG0642;Signal transduction histidine kinase	GO:0006464 protein modification -!- GO:0006805 xenobiotic metabolism	o	tt
cpxP	CDS	periplasmic protein combats stress	Periplasmic		GO:0006350 transcription -!- GO:0006805 xenobiotic metabolism		
cpxR	CDS	DNA-binding response regulator in two-component regulatory system with CpxA	Cytoplasmic	COG0745;Response regulators consisting of a CheY-like receiver domain and a winged-helix DNA-binding domain	GO:0006350 transcription -!- GO:0006805 xenobiotic metabolism	o	tt
crcA	CDS	palmitoyl transferase for Lipid A	Outer Membrane B-barrel protein			o	t
crcB	CDS	predicted inner membrane protein associated with chromosome condensation	Integral Membrane Protein	COG0239;Integral membrane protein possibly involved in chromosome condensation		o	tt
creA	CDS	conserved protein	Periplasmic	COG3045;Uncharacterized protein conserved in bacteria		o	tt
creB	CDS	DNA-binding response regulator in two-component regulatory system with CreC	Cytoplasmic	COG0745;Response regulators consisting of a CheY-like receiver domain and a winged-helix DNA-binding domain	GO:0006350 transcription	o	t
creC	CDS	sensory histidine kinase in two-component regulatory system with CreB or PhoB, regulator of the CreBC regulon	Integral Membrane Protein	COG0642;Signal transduction histidine kinase	GO:0006464 protein modification	o	tt
creD	CDS	inner membrane protein	Integral Membrane Protein	COG4452;Inner membrane protein involved in colicin E2 resistance		o	tt
crl	CDS	DNA-binding transcriptional regulator	Cytoplasmic		GO:0006350 transcription	gg	o
crp	CDS	DNA-binding transcriptional dual regulator	Cytoplasmic	COG0664;cAMP-binding proteins - catabolite gene activator and regulatory subunit of cAMP-dependent protein kinases	GO:0006350 transcription		
crr	CDS	glucose-specific enzyme IIA component of PTS	Cytoplasmic	COG2190;Phosphotransferase system IIA components	GO:0016052 carbohydrate	gg	o
csdA	CDS	cysteine sulfinate desulfinase	Cytoplasmic	COG0520;Selenocysteine lyase	GO:0006790 sulfur metabolism -!- GO:0006520 amino acid metabolism	gg	tt
csgA	CDS	cryptic curlin major subunit	Extracellular		GO:0009101 glycoprotein biosynthesis	o	tt
csgB	CDS	curlin nucleator protein, minor subunit in curli complex	Extracellular		GO:0009101 glycoprotein biosynthesis	o	tt
csgC	CDS	predicted curli production protein	Periplasmic			o	tt
csgD	CDS	DNA-binding transcriptional activator in two-component regulatory system	Cytoplasmic	COG2771;DNA-binding HTH domain-containing proteins	GO:0006350 transcription	o	tt
csgE	CDS	predicted transport protein	Periplasmic			o	tt
csgF	CDS	predicted transport protein	Periplasmic			o	tt
csgG	CDS	outer membrane lipoprotein	Outer Membrane Lipoprotein	COG1462;Uncharacterized protein involved in formation of curli polymers		o	tt
csiE	CDS	stationary phase inducible protein	Cytoplasmic	COG3711;Transcriptional antiterminator			
csiR	CDS	DNA-binding transcriptional dual regulator	Cytoplasmic		GO:0006350 transcription	gg	t
cspA	CDS	major cold shock protein	Cytoplasmic	COG1278;Cold shock proteins	GO:0006350 transcription -!- GO:0009266 response to	gg	tt
cspB	CDS	Qin prophage; cold shock protein	Cytoplasmic	COG1278;Cold shock proteins		gg	t
cspC	CDS	stress protein, member of the CspA-family	Cytoplasmic	COG1278;Cold shock proteins	GO:0006350 transcription -!- GO:0009266 response to	gg	t
cspD	CDS	cold shock protein homolog	Cytoplasmic	COG1278;Cold shock proteins		gg	tt
cspE	CDS	DNA-binding transcriptional repressor	Cytoplasmic	COG1278;Cold shock proteins	GO:0006350 transcription -!- GO:0009266 response to	gg	tt
cspF	CDS	Qin prophage; cold shock protein	Cytoplasmic	COG1278;Cold shock proteins		o	t
cspG	CDS	DNA-binding transcriptional regulator	Cytoplasmic	COG1278;Cold shock proteins	GO:0009266 response to	o	tt
cspH	CDS	stress protein, member of the CspA-family	Cytoplasmic	COG1278;Cold shock proteins	GO:0009266 response to		
cspI	CDS	Qin prophage; cold shock protein	Cytoplasmic	COG1278;Cold shock proteins		gg	tt
csrA	CDS	pleiotropic regulatory protein for carbon source metabolism	Cytoplasmic	COG1551;Carbon storage regulator (could also regulate swarming and quorum sensing)	GO:0016052 carbohydrate catabolism -!- GO:0006096 glycolysis -!- GO:0006094 gluconeogenesis -!- GO:0006401 RNA catabolism -!- GO:0009386 translational attenuation -!- GO:0042330 taxis	gg	o
csrB	misc_RNA	regulatory sRNA					
csrC	misc_RNA	regulatory sRNA					
cstA	CDS	carbon starvation protein	Integral Membrane Protein	COG1966;Carbon starvation protein, predicted membrane protein	GO:0042594 response to starvation	o	tt
cueO	CDS	multicopper oxidase (laccase)	Periplasmic	COG2132;Putative multicopper oxidases		gg	tt
cueR	CDS	DNA-binding transcriptional activator	Cytoplasmic	COG0789;Predicted transcriptional regulators	GO:0006350 transcription	gg	t
cusA	CDS	copper/silver efflux system, membrane component	Integral Membrane Protein	COG3696;Putative silver efflux pump		o	tt
cusB	CDS	copper/silver efflux system, membrane fusion protein	Membrane Anchored	COG0845;Membrane-fusion protein	GO:0042493 response to drug	o	tt
cusC	CDS	copper/silver efflux system, outer membrane component	Outer Membrane Lipoprotein	COG1538;Outer membrane protein		o	tt
cusF	CDS	periplasmic copper-binding protein	Periplasmic			o	tt
cusR	CDS	DNA-binding response regulator in two-component regulatory system with CusS	Cytoplasmic	COG0745;Response regulators consisting of a CheY-like receiver domain and a winged-helix DNA-binding domain	GO:0006350 transcription	gg	tt
cusS	CDS	sensory histidine kinase in two-component regulatory system with CusR, senses copper ions	Integral Membrane Protein	COG0642;Signal transduction histidine kinase	GO:0006464 protein modification		
cutA	CDS	copper binding protein, copper sensitivity	Cytoplasmic	COG1324;Uncharacterized protein involved in tolerance to divalent cations	GO:0017004 cytochrome biogenesis	gg	o
cutC	CDS	copper homeostasis protein	Cytoplasmic		GO:0006805 xenobiotic metabolism	gg	t
cvpA	CDS	membrane protein required for colicin V production	Integral Membrane Protein	COG1286;Uncharacterized membrane protein, required for colicin V production		o	tt
cvrA	CDS	predicted cation/proton antiporter	Integral Membrane Protein	COG3263;NhaP-type Na+/H+ and K+/H+ antiporters with a unique C-terminal domain			
cyaA	CDS	adenylate cyclase	Cytoplasmic	COG3072;Adenylate cyclase		o	tt
cyaY	CDS	frataxin, iron-binding and oxidizing protein	Cytoplasmic	COG1965;Protein implicated in iron transport, frataxin homolog		gg	o
cybB	CDS	cytochrome b561	Integral Membrane Protein	COG3038;Cytochrome B561	GO:0017004 cytochrome biogenesis		
cybC	CDS	cytochrome b562, truncated (pseudogene)	Cytoplasmic		GO:0017004 cytochrome biogenesis	gg	o

cycA	CDS	D-alanine/D-serine/glycine transporter	Integral Membrane Protein	COG1113;Gamma-aminobutyrate permease and related permeases	GO:0009063 amino acid catabolism -!- GO:0009252 peptidoglycan biosynthesis -!- GO:0006545 glycine biosynthesis -!- GO:0006523 alanine biosynthesis -!- GO:0006164 purine nucleotide biosynthesis -!- GO:0009257 10-formyltetrahydrofolate biosynthesis -!-	o	tt
cydA	CDS	cytochrome d terminal oxidase, subunit I	Integral Membrane Protein	COG1271;Cytochrome bd-type quinol oxidase, subunit 1		o	tt
cydB	CDS	cytochrome d terminal oxidase, subunit II	Integral Membrane Protein	COG1294;Cytochrome bd-type quinol oxidase, subunit 2		o	tt
cydC	CDS	fused cysteine transporter subunits -!- membrane component and ATP-binding component of ABC superfamily	Integral Membrane Protein	COG4987;ABC-type transport system involved in cytochrome bd biosynthesis, fused ATPase and permease components		o	tt
cydD	CDS	fused cysteine transporter subunits -!- membrane component and ATP-binding component of ABC superfamily	Integral Membrane Protein	COG4988;ABC-type transport system involved in cytochrome bd biosynthesis, ATPase and permease components		o	tt
cynR	CDS	DNA-binding transcriptional dual regulator	Cytoplasmic	COG0583;Transcriptional regulator	GO:0006350 transcription -!- GO:0006805 xenobiotic metabolism		
cynS	CDS	cyanate aminohydrolase	Cytoplasmic	COG1513;Cyanate lyase	GO:0009440 cyanate catabolism -!- GO:0006807 nitrogen metabolism -!- GO:0006805 xenobiotic metabolism	gg	t
cynT	CDS	carbonic anhydrase	Cytoplasmic	COG0288;Carbonic anhydrase	GO:0009440 cyanate catabolism -!- GO:0006805 xenobiotic metabolism	gg	t
cynX	CDS	predicted cyanate transporter	Integral Membrane Protein	COG2807;Cyanate permease	GO:0009440 cyanate catabolism -!- GO:0006805 xenobiotic metabolism	o	tt
cyoA	CDS	cytochrome o ubiquinol oxidase subunit II	Integral Membrane Protein	COG1622;Heme/copper-type cytochrome/quinol oxidases, subunit 2	GO:0009060 aerobic respiration	o	tt
cyoB	CDS	cytochrome o ubiquinol oxidase subunit I	Integral Membrane Protein	COG0843;Heme/copper-type cytochrome/quinol oxidases, subunit 1	GO:0009060 aerobic respiration	o	tt
cyoC	CDS	cytochrome o ubiquinol oxidase subunit III	Integral Membrane Protein	COG1845;Heme/copper-type cytochrome/quinol oxidase, subunit 3	GO:0009060 aerobic respiration	o	tt
cyoD	CDS	cytochrome o ubiquinol oxidase subunit IV	Integral Membrane Protein	COG3125;Heme/copper-type cytochrome/quinol oxidase, subunit 4	GO:0009060 aerobic respiration	o	tt
cyoE	CDS	protoheme IX farnesyltransferase	Integral Membrane Protein	COG0109;Polyprenyltransferase (cytochrome oxidase assembly factor)	GO:0009060 aerobic respiration	o	tt
cysA	CDS	sulfate/thiosulfate transporter subunit -!- ATP-binding component of ABC superfamily	Cytoplasmic	COG1118;ABC-type sulfate/molybdate transport systems, ATPase component	GO:0006790 sulfur metabolism -!- GO:0042493 response to drug	gg	t
cysB	CDS	DNA-binding transcriptional dual regulator, O-acetyl-L-serine-binding	Cytoplasmic	COG0583;Transcriptional regulator	GO:0019344 cysteine biosynthesis -!- GO:0006350 transcription	gg	tt
cysC	CDS	adenosine 5'-phosphosulfate kinase	Cytoplasmic	COG0529;Adenylylsulfate kinase and related kinases	GO:0006790 sulfur metabolism	gg	t
cysD	CDS	sulfate adenylyltransferase, subunit 2	Cytoplasmic	COG0175;3'-phosphoadenosine 5'-phosphosulfate sulfotransferase (PAPS reductase)/FAD synthetase and related enzymes	GO:0006790 sulfur metabolism	gg	t
cysE	CDS	serine acetyltransferase	Cytoplasmic	COG1045;Serine acetyltransferase	GO:0019344 cysteine biosynthesis	gg	tt
cysG	CDS	fused siroheme synthase 1,3-dimethyluroporphyriongen III dehydrogenase/siroheme ferrochelatase -!-	Cytoplasmic	COG1648;Siroheme synthase (precorrin-2 oxidase/ferrochelatase domain) -!- COG0007;Uroporphyrinogen-III methylase		o	tt
cysH	CDS	3'-phosphoadenosine 5'-phosphosulfate reductase	Cytoplasmic	COG0175;3'-phosphoadenosine 5'-phosphosulfate sulfotransferase (PAPS reductase)/FAD synthetase and related enzymes	GO:0006790 sulfur metabolism	gg	t
cysI	CDS	sulfite reductase, beta subunit, NAD(P)-binding	Cytoplasmic	COG0155;Sulfite reductase, beta subunit (hemoprotein)	GO:0006790 sulfur metabolism	o	tt
cysJ	CDS	sulfite reductase, alpha subunit, flavoprotein	Cytoplasmic	COG0369;Sulfite reductase, alpha subunit (flavoprotein)	GO:0006790 sulfur metabolism	o	tt
cysK	CDS	cysteine synthase A, O-acetylserine sulfhydrolase A subunit	Cytoplasmic	COG0031;Cysteine synthase	GO:0019344 cysteine biosynthesis	gg	t
cysM	CDS	cysteine synthase B (O-acetylserine sulfhydrolase B)	Cytoplasmic	COG0031;Cysteine synthase	GO:0019344 cysteine biosynthesis	gg	t
cysN	CDS	sulfate adenylyltransferase, subunit 1	Cytoplasmic	COG2895;GTPases - Sulfate adenylate transferase subunit 1	GO:0006790 sulfur metabolism	gg	tt
cysP	CDS	thiosulfate transporter subunit -!- periplasmic-binding component of ABC superfamily	Periplasmic	COG4150;ABC-type sulfate transport system, periplasmic component	GO:0006790 sulfur metabolism	o	tt
cysQ	CDS	PAPS (adenosine 3'-phosphate 5'-phosphosulfate) 3'(2'),5'-bisphosphate nucleotidase	Cytoplasmic	COG1218;3'-Phosphoadenosine 5'-phosphosulfate (PAPS) 3'-phosphatase	GO:0006790 sulfur metabolism	gg	tt
cysS	CDS	cysteinyl-tRNA synthetase	Cytoplasmic	COG0215;Cysteinyl-tRNA synthetase	GO:0006418 amino acid activation	gg	tt
cysT	tRNA	tRNA-Cys(GCA) (Cysteine tRNA)					
cysU	CDS	sulfate/thiosulfate transporter subunit -!- membrane component of ABC superfamily	Integral Membrane Protein	COG0555;ABC-type sulfate transport system, permease component	GO:0006790 sulfur metabolism	o	tt
cysW	CDS	sulfate/thiosulfate transporter subunit -!- membrane component of ABC superfamily	Integral Membrane Protein		GO:0006790 sulfur metabolism	gg	t
cysZ	CDS	predicted inner membrane protein	Integral Membrane Protein	COG2981;Uncharacterized protein involved in cysteine biosynthesis	GO:0006790 sulfur metabolism	g	tt
cytR	CDS	DNA-binding transcriptional dual regulator	Cytoplasmic	COG1609;Transcriptional regulators	GO:0015949 nucleobase, nucleoside and nucleotide interconversion -!- GO:0006350 transcription	o	tt
dacA	CDS	D-alanyl-D-alanine carboxypeptidase (penicillin-binding protein 5)	Periplasmic	COG1686;D-alanyl-D-alanine carboxypeptidase	GO:0042493 response to drug	o	tt
dacB	CDS	D-alanyl-D-alanine carboxypeptidase	Periplasmic	COG2027;D-alanyl-D-alanine carboxypeptidase (penicillin-binding protein 4)	GO:0009252 peptidoglycan biosynthesis -!- GO:0042493 response to drug	o	tt
dacC	CDS	D-alanyl-D-alanine carboxypeptidase (penicillin-binding protein 6a)	Periplasmic	COG1686;D-alanyl-D-alanine carboxypeptidase	GO:0042493 response to drug	o	tt
dacD	CDS	D-alanyl-D-alanine carboxypeptidase (penicillin-binding protein 6b)	Periplasmic	COG1686;D-alanyl-D-alanine carboxypeptidase			
dadA	CDS	D-amino acid dehydrogenase	Cytoplasmic	COG0665;Glycine/D-amino acid oxidases (deaminating)	GO:0009063 amino acid catabolism	o	tt
dadX	CDS	alanine racemase 2, PLP-binding	Cytoplasmic	COG0787;Alanine racemase	GO:0009063 amino acid catabolism -!- GO:0006523 alanine biosynthesis	o	tt
dam	CDS	DNA adenine methylase	Cytoplasmic	COG0338;Site-specific DNA methylase	GO:0006306 DNA methylation	o	tt
damX	CDS	predicted protein	Membrane Anchored	COG3266;Uncharacterized protein conserved in bacteria		o	tt
dapA	CDS	dihydrodipicolinate synthase	Cytoplasmic	COG0329;Dihydrodipicolinate synthase/N-acetylneuraminate lyase	GO:0009089 lysine biosynthesis via diaminopimelate	gg	o
dapB	CDS	dihydrodipicolinate reductase	Cytoplasmic	COG0289;Dihydrodipicolinate reductase	GO:0009089 lysine biosynthesis via diaminopimelate	gg	o
dapD	CDS	2,3,4,5-tetrahydropyridine-2-carboxylate N-succinyltransferase	Cytoplasmic	COG2171;Tetrahydrodipicolinate N-succinyltransferase	GO:0009089 lysine biosynthesis via diaminopimelate	gg	tt
dapE	CDS	N-succinyl-diaminopimelate deacylase	Cytoplasmic	COG0624;Acetylornithine deacetylase/Succinyl-diaminopimelate desuccinylase and related deacylases	GO:0009089 lysine biosynthesis via diaminopimelate	g	tt
dapF	CDS	diaminopimelate epimerase	Cytoplasmic	COG0253;Diaminopimelate epimerase	GO:0009089 lysine biosynthesis via diaminopimelate		
dbpA	CDS	ATP-dependent RNA helicase specific for 23S rRNA	Cytoplasmic	COG0513;Superfamily II DNA and RNA helicases	GO:0009451 RNA modification	gg	t
dcd	CDS	2'-deoxycytidine 5'-triphosphate deaminase	Cytoplasmic	COG0717;Deoxycytidine deaminase	GO:0015949 nucleobase, nucleoside and nucleotide interconversion	gg	o
dcm	CDS	DNA cytosine methylase	Cytoplasmic	COG0270;Site-specific DNA methylase	GO:0006306 DNA methylation	gg	tt
dcp	CDS	dipeptidyl carboxypeptidase II	Cytoplasmic	COG0339;Zn-dependent oligopeptidases		gg	t
dcrB	CDS	periplasmic protein	Inner Membrane Lipoprotein				
dctA	CDS	C4-dicarboxylic acid, orotate and citrate transporter	Integral Membrane Protein	COG1301;Na+/H+-dicarboxylate symporters	GO:0016052 carbohydrate	o	tt
dcuA	CDS	C4-dicarboxylate antiporter	Integral Membrane Protein	COG2704;Anaerobic C4-dicarboxylate transporter	GO:0016052 carbohydrate		
dcuB	CDS	C4-dicarboxylate antiporter	Integral Membrane Protein	COG2704;Anaerobic C4-dicarboxylate transporter	GO:0016052 carbohydrate	o	tt
dcuC	CDS	anaerobic C4-dicarboxylate transport	Integral Membrane Protein	COG3069;C4-dicarboxylate transporter	GO:0016052 carbohydrate		
dcuC	CDS	anaerobic C4-dicarboxylate transport	Integral Membrane Protein			o	tt
dcuC	CDS	anaerobic C4-dicarboxylate transport	Integral Membrane Protein			o	tt
dcuD	CDS	predicted transporter	Integral Membrane Protein	COG3069;C4-dicarboxylate transporter		o	tt
dcuR	CDS	DNA-binding response regulator in two-component regulatory system with DcuS	Cytoplasmic	COG4565;Response regulator of citrate/malate metabolism	GO:0009061 anaerobic respiration -!- GO:0006350 transcription	o	o
dcuS	CDS	sensory histidine kinase in two-component regulatory system with DcuR, regulator of anaerobic fumarate respiration	Integral Membrane Protein	COG3290;Signal transduction histidine kinase regulating citrate/malate metabolism	GO:0009061 anaerobic respiration -!- GO:0006464 protein modification	o	tt
ddg	CDS	palmitoleoyl-acyl carrier protein (ACP)-dependent acyltransferase	Membrane Anchored		GO:0009245 lipid A biosynthesis -!- GO:0009266 response to	gg	tt
ddlA	CDS	D-alanine-D-alanine ligase A	Cytoplasmic	COG1181;D-alanine-D-alanine ligase and related ATP-grasp enzymes	GO:0009252 peptidoglycan biosynthesis	gg	tt
ddlB	CDS	D-Alanine:D-alanine ligase	Cytoplasmic	COG1181;D-alanine-D-alanine ligase and related ATP-grasp enzymes	GO:0009252 peptidoglycan biosynthesis	gg	o
ddpA	CDS	D-Ala-D-Ala transporter subunit -!- periplasmic-binding component of ABC superfamily	Periplasmic	COG0747;ABC-type dipeptide transport system, periplasmic component			
ddpB	CDS	D-Ala-D-Ala transporter subunit -!- membrane component of ABC superfamily	Integral Membrane Protein	COG0601;ABC-type dipeptide/oligopeptide/nickel transport systems, permease components		o	tt
ddpC	CDS	D-Ala-D-Ala transporter subunit -!- membrane component of ABC superfamily	Integral Membrane Protein	COG1173;ABC-type dipeptide/oligopeptide/nickel transport systems, permease components		o	tt
ddpD	CDS	D-Ala-D-Ala transporter subunit -!- ATP-binding component of ABC superfamily	Cytoplasmic	COG0444;ABC-type dipeptide/oligopeptide/nickel transport system, ATPase component		gg	o
ddpF	CDS	D-Ala-D-Ala transporter subunit -!- ATP-binding component of ABC superfamily	Cytoplasmic	COG1124;ABC-type dipeptide/oligopeptide/nickel transport system, ATPase component		g	t
ddpX	CDS	D-Ala-D-Ala dipeptidase, Zn-dependent	Cytoplasmic	COG2173;D-alanyl-D-alanine dipeptidase	GO:0006970 response to osmotic stress	gg	t
deaD	CDS	ATP-dependent RNA helicase	Cytoplasmic	COG0513;Superfamily II DNA and RNA helicases	GO:0009451 RNA modification		
dedA	CDS	conserved inner membrane protein	Integral Membrane Protein	COG0586;Uncharacterized membrane-associated protein		o	tt
dedD	CDS	conserved protein	Membrane Anchored	COG3147;Uncharacterized protein conserved in bacteria			
def	CDS	peptide deformylase	Cytoplasmic	COG0242;N-formylmethionyl-tRNA deformylase	GO:0006464 protein modification	gg	t
degP	CDS	serine endoprotease (protease Do), membrane-associated	Periplasmic	COG0265;Trypsin-like serine proteases, typically periplasmic, contain C-terminal PDZ domain	GO:0009266 response to temperature	o	tt
degQ	CDS	serine endoprotease, periplasmic	Membrane Anchored	COG0265;Trypsin-like serine proteases, typically periplasmic, contain C-terminal PDZ domain		o	tt
degS	CDS	serine endoprotease, periplasmic	Membrane Anchored	COG0265;Trypsin-like serine proteases, typically periplasmic, contain C-terminal PDZ domain	GO:0006508 proteolysis and peptidolysis	o	tt
deoA	CDS	thymidine phosphorylase	Cytoplasmic	COG0213;Thymidine phosphorylase	GO:0015949 nucleobase, nucleoside and nucleotide interconversion	gg	t
deoB	CDS	phosphopentomutase	Cytoplasmic	COG1015;Phosphopentomutase	GO:0015949 nucleobase, nucleoside and nucleotide interconversion	gg	t
deoC	CDS	2-deoxyribose-5-phosphate aldolase, NAD(P)-linked	Cytoplasmic	COG0274;Deoxyribose-phosphate aldolase	GO:0015949 nucleobase, nucleoside and nucleotide interconversion	gg	o

deoD	CDS	purine-nucleoside phosphorylase	Cytoplasmic	COG0813;Purine-nucleoside phosphorylase	GO:0015949 nucleobase, nucleoside and nucleotide interconversion	g	o
deoR	CDS	DNA-binding transcriptional repressor	Cytoplasmic	COG1349;Transcriptional regulators of sugar metabolism	GO:0015949 nucleobase, nucleoside and nucleotide interconversion -!- GO:0006350 transcription	gg	t
der	CDS	predicted GTP-binding protein	Cytoplasmic	COG1160;Predicted GTPases			
dfp	CDS	fused 4'-phosphopantothenoylcysteine decarboxylase -!- phosphopantothenoylcysteine synthetase, FMN-binding	Cytoplasmic		GO:0015937 coenzyme A biosynthesis -!- GO:0006261 DNA dependent DNA replication		
dgkA	CDS	diacylglycerol kinase	Integral Membrane Protein	COG0818;Diacylglycerol kinase	GO:0008654 phospholipid biosynthesis	o	tt
dgoA	CDS	2-oxo-3-deoxygalactonate 6-phosphate aldolase	Cytoplasmic		GO:0016052 carbohydrate		
dgoD	CDS	galactonate dehydratase	Cytoplasmic		GO:0016052 carbohydrate		
dgoK	CDS	2-oxo-3-deoxygalactonate kinase	Cytoplasmic	COG3734;2-keto-3-deoxy-galactonokinase	GO:0016052 carbohydrate	g	t
dgoR	CDS	predicted DNA-binding transcriptional regulator	Cytoplasmic		GO:0016052 carbohydrate catabolism -!- GO:0006350		
dgoT	CDS	D-galactonate transporter	Integral Membrane Protein		GO:0016052 carbohydrate		
dgsA	CDS	DNA-binding transcriptional repressor	Cytoplasmic	COG1940;Transcriptional regulator/sugar kinase	GO:0000271 polysaccharide biosynthesis -!- GO:0006350 transcription	o	tt
dgt	CDS	deoxyguanosine triphosphate triphosphohydrolase	Cytoplasmic	COG0232;dGTP triphosphohydrolase	GO:0015949 nucleobase, nucleoside and nucleotide interconversion	gg	t
dhaH	CDS	fused predicted dihydroxyacetone-specific PTS enzyme HPr component -!- EI component	Cytoplasmic	COG3412;Uncharacterized protein conserved in bacteria -!- COG1080;Phosphoenolpyruvate-protein kinase (PTS system EI component in bacteria)			
dhaK	CDS	dihydroxyacetone kinase, N-terminal domain	Cytoplasmic				
dhaL	CDS	dihydroxyacetone kinase, C-terminal domain	Cytoplasmic	COG2376;Dihydroxyacetone kinase			
dhaR	CDS	predicted DNA-binding transcriptional regulator, dihydroxyacetone	Cytoplasmic	COG3284;Transcriptional activator of acetoin/glycerol metabolism	GO:0006350 transcription		
dicA	CDS	Qin prophage; predicted regulator for DicB	Cytoplasmic	COG1396;Predicted transcriptional regulators		o	tt
dicB	CDS	Qin prophage; cell division inhibition protein	Cytoplasmic			o	tt
dicC	CDS	Qin prophage; DNA-binding transcriptional regulator	Cytoplasmic			gg	o
dicF	misc_RNA	Qin prophage; DicF antisense RNA					
dinB	CDS	DNA polymerase IV	Cytoplasmic	COG0389;Nucleotidyltransferase/DNA polymerase involved in DNA repair	GO:0006261 DNA dependent DNA replication -!- GO:0009432 SOS response	o	tt
dinD	CDS	DNA-damage-inducible protein	Cytoplasmic		GO:0006281 DNA repair	gg	o
dinF	CDS	DNA-damage-inducible SOS response protein	Integral Membrane Protein	COG0534;Na+-driven multidrug efflux pump	GO:0006281 DNA repair -!- GO:0009314 response to radiation -!- GO:0009432 SOS response	o	tt
dinG	CDS	ATP-dependent DNA helicase	Cytoplasmic	COG1199;Rad3-related DNA helicases	GO:0006281 DNA repair -!- GO:0009432 SOS response	o	tt
dinI	CDS	DNA damage-inducible protein I	Cytoplasmic		GO:0006281 DNA repair -!- GO:0009432 SOS response	g	o
dinJ	CDS	predicted antitoxin of YafQ-DinJ toxin-antitoxin	Cytoplasmic	COG3077;DNA-damage-inducible protein J	GO:0006281 DNA repair	gg	t
dipZ	CDS	fused thiol:disulfide interchange protein	Integral Membrane Protein	COG4232;Thiol:disulfide interchange protein	GO:0006790 sulfur metabolism -!- GO:0017004 cytochrome biogenesis		
djlA	CDS	DnaJ-like protein, membrane anchored	Membrane Anchored	COG1076;DnaJ-domain-containing proteins 1	GO:0006457 protein folding	o	tt
djlB	CDS	predicted chaperone	Membrane Anchored		GO:0006457 protein folding	o	tt
djlC	CDS	Hsc56 co-chaperone of HscC	Cytoplasmic			o	tt
dkgA	CDS	2,5-diketo-D-gluconate reductase A	Cytoplasmic		GO:0016052 carbohydrate		
dkgB	CDS	2,5-diketo-D-gluconate reductase B	Cytoplasmic	COG0656;Aldo/keto reductases, related to diketogulonate reductase	GO:0016052 carbohydrate	gg	o
dksA	CDS	transcriptional regulator of rRNA transcription, DnaK suppressor protein	Cytoplasmic	COG1734;DnaK suppressor protein	GO:0006457 protein folding	gg	o
dld	CDS	D-lactate dehydrogenase, FAD-binding, NADH independent	Cytoplasmic	COG0277;FAD/FMN-containing dehydrogenases	GO:0009060 aerobic respiration	gg	tt
dmsA	CDS	dimethyl sulfoxide reductase, anaerobic, subunit A	Periplasmic		GO:0009061 anaerobic respiration		
dmsB	CDS	dimethyl sulfoxide reductase, anaerobic, subunit B	Cytoplasmic	COG0437;Fe-S-cluster-containing hydrogenase components 1	GO:0009061 anaerobic respiration	gg	t
dmsC	CDS	dimethyl sulfoxide reductase, anaerobic, subunit C	Integral Membrane Protein	COG3302;DMSO reductase anchor subunit	GO:0009061 anaerobic respiration	o	tt
dmsD	CDS	twin-argninine leader-binding protein for DmsA and TorA	Cytoplasmic	COG3381;Uncharacterized component of anaerobic dehydrogenases			
dnaA	CDS	chromosomal replication initiator protein DnaA, DNA-binding transcriptional dual regulator	Cytoplasmic	COG0593;ATPase involved in DNA replication initiation	GO:0006261 DNA dependent DNA replication	o	tt
dnaB	CDS	replicative DNA helicase	Cytoplasmic	COG0305;Replicative DNA helicase	GO:0006261 DNA dependent DNA replication	o	tt
dnaC	CDS	DNA biosynthesis protein	Cytoplasmic	COG1484;DNA replication protein	GO:0006261 DNA dependent DNA replication	o	tt
dnaE	CDS	DNA polymerase III alpha subunit	Cytoplasmic	COG0587;DNA polymerase III, alpha subunit	GO:0006261 DNA dependent DNA replication	o	tt
dnaG	CDS	DNA primase	Cytoplasmic	COG0358;DNA primase (bacterial type)	GO:0006261 DNA dependent DNA replication	gg	t
dnaJ	CDS	chaperone Hsp40, co-chaperone with DnaK	Cytoplasmic	COG0484;DnaJ-class molecular chaperone with C-terminal Zn finger domain	GO:0006457 protein folding	o	tt
dnaK	CDS	chaperone Hsp70, co-chaperone with DnaJ	Cytoplasmic	COG0443;Molecular chaperone	GO:0006457 protein folding -!- GO:0006970 response to osmotic stress	gg	t
dnaN	CDS	DNA polymerase III, beta subunit	Cytoplasmic	COG0592;DNA polymerase sliding clamp subunit (PCNA homolog)	GO:0006261 DNA dependent DNA replication	o	t
dnaQ	CDS	DNA polymerase III epsilon subunit	Cytoplasmic	COG0847;DNA polymerase III, epsilon subunit and related 3'-5' exonucleases	GO:0006261 DNA dependent DNA replication -!- GO:0006457 protein folding	gg	t
dnaT	CDS	DNA biosynthesis protein (primosomal protein I)	Cytoplasmic		GO:0006261 DNA dependent DNA replication	o	t
dnaX	CDS	DNA polymerase III/DNA elongation factor III, tau and gamma subunits	Cytoplasmic	COG2812;DNA polymerase III, gamma/tau subunits	GO:0006261 DNA dependent DNA replication	o	tt
dos	CDS	cAMP phosphodiesterase, heme-regulated	Cytoplasmic	COG2202;FOG: PAS/PAC domain -!- COG2199;FOG: GGDEF domain -!- COG2200;FOG: EAL domain		o	tt
dppA	CDS	dipeptide transporter -!- periplasmic-binding component of ABC superfamily	Periplasmic	COG0747;ABC-type dipeptide transport system, periplasmic component	GO:0009063 amino acid catabolism	o	tt
dppB	CDS	dipeptide transporter-!- membrane component of ABC superfamily	Integral Membrane Protein	COG0601;ABC-type dipeptide/oligopeptide/nickel transport systems, permease components		o	o
dppC	CDS	dipeptide transporter -!- membrane component of ABC superfamily	Integral Membrane Protein	COG1173;ABC-type dipeptide/oligopeptide/nickel transport systems, permease components		o	tt
dppD	CDS	dipeptide transporter -!- ATP-binding component of ABC superfamily	Cytoplasmic	COG0444;ABC-type dipeptide/oligopeptide/nickel transport system, ATPase component	GO:0009063 amino acid catabolism	g	t
dppF	CDS	dipeptide transporter -!- ATP-binding component of ABC superfamily	Cytoplasmic	COG4608;ABC-type oligopeptide transport system, ATPase component	GO:0009063 amino acid catabolism	o	tt
dps	CDS	Fe-binding and storage protein	Cytoplasmic	COG0783;DNA-binding ferritin-like protein (oxidative damage protectant)	GO:0042594 response to starvation	gg	t
dsbA	CDS	periplasmic protein disulfide isomerase I	Periplasmic	COG0526;Thiol-disulfide isomerase and thioredoxins	GO:0006457 protein folding -!- GO:0006950 response to stress	o	tt
dsbB	CDS	oxidoreductase that catalyzes reoxidation of DsbA protein disulfide isomerase I	Integral Membrane Protein		GO:0009296 flagella biogenesis -!- GO:0006457 protein folding -!- GO:0042330 taxis		
dsbC	CDS	protein disulfide isomerase II	Periplasmic	COG1651;Protein-disulfide isomerase	GO:0006457 protein folding	gg	tt
dsbG	CDS	periplasmic disulfide isomerase/thiol-disulphide oxidase	Periplasmic	COG1651;Protein-disulfide isomerase	GO:0006457 protein folding	o	tt
dsdA	CDS	D-serine ammonia-lyase	Cytoplasmic	COG3048;D-serine dehydratase	GO:0009063 amino acid catabolism	gg	t
dsdC	CDS	DNA-binding transcriptional dual regulator	Cytoplasmic	COG0583;Transcriptional regulator	GO:0009063 amino acid catabolism -!- GO:0006350 transcription	gg	t
dsdX	CDS	predicted transporter	Integral Membrane Protein	COG2610;H+/gluconate symporter and related permeases		g	tt
dsrA	misc_RNA	regulatory, anti-sense RNA					
dsrB	CDS	predicted protein	Cytoplasmic			gg	o
dtd	CDS	D-Tyr-tRNA(Tyr) deacylase	Cytoplasmic	COG1490;D-Tyr-tRNAtyr deacylase	GO:0009451 RNA modification -!- GO:0006805 xenobiotic metabolism	o	t
dusB	CDS	tRNA-dihydrouridine synthase B	Cytoplasmic	COG0042;tRNA-dihydrouridine synthase		g	t
dusC	CDS	tRNA-dihydrouridine synthase C	Cytoplasmic	COG0042;tRNA-dihydrouridine synthase		gg	t
dut	CDS	deoxyuridinetriphosphatase	Cytoplasmic	COG0756;dUTPase	GO:0015949 nucleobase, nucleoside and nucleotide interconversion	gg	o
dxr	CDS	1-deoxy-D-xylulose 5-phosphate reductoisomerase	Cytoplasmic	COG0743;1-deoxy-D-xylulose 5-phosphate reductoisomerase		gg	t
dxs	CDS	1-deoxyxylulose-5-phosphate synthase, thiamine-requiring, FAD-requiring	Cytoplasmic	COG1154;Deoxyxylulose-5-phosphate synthase	GO:0008615 pyridoxine biosynthesis -!- GO:0009228 thiamin biosynthesis	o	tt
eaeH	CDS	attaching and effacing protein, pathogenesis factor	Membrane Anchored			o	tt
eamA	CDS	cysteine and O-acetyl-L-serine efflux system	Integral Membrane Protein				
ebgA	CDS	cryptic beta-D-galactosidase, alpha subunit	Cytoplasmic		GO:0016052 carbohydrate		
ebgC	CDS	cryptic beta-D-galactosidase, beta subunit	Cytoplasmic	COG2731;Beta-galactosidase, beta subunit	GO:0016052 carbohydrate	o	t
ebgR	CDS	DNA-binding transcriptional repressor	Cytoplasmic	COG1609;Transcriptional regulators	GO:0016052 carbohydrate catabolism -!- GO:0006350	gg	tt
ecnA	CDS	entericidin A membrane lipoprotein, antidote entericidin B	Outer Membrane Lipoprotein				
ecnB	CDS	entericidin B membrane lipoprotein	Outer Membrane Lipoprotein			o	o
eco	CDS	ecotin, a serine protease inhibitor	Periplasmic	COG4574;Serine protease inhibitor ecotin		o	tt
ecpD	CDS	predicted periplasmic pilin chaperone	Periplasmic	COG3121;P pilus assembly protein, chaperone PapD	GO:0006457 protein folding	o	tt
eda	CDS	multifunctional 2-keto-3-deoxygluconate 6-phosphate aldolase -!- 2-keto-4-hydroxyglutarate aldolase -!- oxaloacetate decarboxylase	Cytoplasmic	COG0800;2-keto-3-deoxy-6-phosphogluconate aldolase	GO:0016052 carbohydrate catabolism -!- GO:0009255 Entner-Doudoroff pathway -!- GO:0009436 glyoxylate catabolism	gg	t
edd	CDS	6-phosphogluconate dehydratase	Cytoplasmic	COG0129;Dihydroxyacid dehydratase/phosphogluconate dehydratase	GO:0009255 Entner-Doudoroff pathway	gg	o

efp	CDS	Elongation factor EF-P	Cytoplasmic	COG0231;Translation elongation factor P (EF-P)/translation initiation factor 5A (eIF-5A)	GO:0006412 protein biosynthesis	gg	o
ego	CDS	fused AI2 transporter subunits -!- ATP-binding components of ABC superfamily	Cytoplasmic	COG1129;ABC-type sugar transport system, ATPase component		gg	t
elaA	CDS	predicted acyltransferase with acyl-CoA N-acyltransferase domain	Cytoplasmic	COG2153;Predicted acyltransferase		gg	tt
elaB	CDS	conserved protein	Membrane Anchored	COG4575;Uncharacterized conserved protein		g	o
elaC	CDS	binuclear zinc phosphodiesterase	Cytoplasmic			gg	t
elaD	CDS	predicted enzyme	Cytoplasmic				
elbA	CDS	predicted protein	Cytoplasmic			o	o
elbB	CDS	isoprenoid biosynthesis protein with amidotransferase-like domain	Cytoplasmic	COG3155;Uncharacterized protein involved in an early stage of isoprenoid biosynthesis		gg	t
emrA	CDS	multidrug efflux system	Membrane Anchored	COG1566;Multidrug resistance efflux pump	GO:0042493 response to drug	o	tt
emrB	CDS	multidrug efflux system protein	Integral Membrane Protein	COG0477;Permeases of the major facilitator superfamily	GO:0042493 response to drug	g	tt
emrD	CDS	multidrug efflux system protein	Integral Membrane Protein	COG0477;Permeases of the major facilitator superfamily	GO:0042493 response to drug		
emrE	CDS	DLP12 prophage; multidrug resistance protein	Integral Membrane Protein	COG2076;Membrane transporters of cations and cationic drugs	GO:0042493 response to drug	o	tt
emrK	CDS	EmrKY-TolC multidrug resistance efflux pump, membrane fusion protein component	Periplasmic	COG1566;Multidrug resistance efflux pump	GO:0042493 response to drug	gg	tt
emrY	CDS	predicted multidrug efflux system	Integral Membrane Protein	COG0477;Permeases of the major facilitator superfamily	GO:0042493 response to drug	o	tt
emtA	CDS	lytic murein endotransglycosylase E	Outer Membrane Lipoprotein	COG0741;Soluble lytic murein transglycosylase and related regulatory proteins (some contain LysM/invasin domains)	GO:0009252 peptidoglycan biosynthesis		
endA	CDS	DNA-specific endonuclease I	Periplasmic	COG2356;Endonuclease I	GO:0006308 DNA catabolism -!- GO:0006308 DNA catabolism	gg	tt
eno	CDS	enolase	Cytoplasmic	COG0148;Enolase	GO:0006096 glycolysis -!- GO:0009061 anaerobic respiration -!- GO:0006094 gluconeogenesis	gg	o
entA	CDS	2,3-dihydro-2,3-dihydroxybenzoate dehydrogenase	Cytoplasmic	COG1028;Dehydrogenases with different specificities (related to short-chain alcohol dehydrogenases)	GO:0009239 enterobactin biosynthesis	gg	t
entB	CDS	isochorismatase	Cytoplasmic	COG1535;Isochorismate hydrolase -!- COG3433;Aryl carrier domain	GO:0009239 enterobactin biosynthesis -!- GO:0019184 nonribosomal peptide biosynthesis	gg	tt
entC	CDS	isochorismate synthase 1	Cytoplasmic	COG1169;Isochorismate synthase	GO:0009239 enterobactin biosynthesis	gg	t
entD	CDS	phosphopantetheinyltransferase component of enterobactin synthase multienzyme complex	Membrane Anchored	COG2977;Phosphopantetheinyl transferase component of siderophore synthetase	GO:0009239 enterobactin biosynthesis		
entE	CDS	2,3-dihydroxybenzoate-AMP ligase component of enterobactin synthase multienzyme complex	Cytoplasmic	COG1021;Peptide arylation enzymes	GO:0009239 enterobactin biosynthesis -!- GO:0019184 nonribosomal peptide biosynthesis	gg	t
entF	CDS	enterobactin synthase multienzyme complex component, ATP-dependent	Cytoplasmic	COG1020;Non-ribosomal peptide synthetase modules and related proteins -!- COG3319;Thioesterase domains of type I polyketide synthases or non-ribosomal peptide synthetases	GO:0009239 enterobactin biosynthesis -!- GO:0015937 coenzyme A biosynthesis -!- GO:0019184 nonribosomal peptide biosynthesis -!- GO:0006826 iron ion transport	o	tt
envC	CDS	protease with a role in cell division	Periplasmic				
envR	CDS	DNA-binding transcriptional regulator	Cytoplasmic	COG1309;Transcriptional regulator	GO:0006350 transcription -!- GO:0042493 response to drug	gg	t
envY	CDS	DNA-binding transcriptional activator	Cytoplasmic	COG2207;AraC-type DNA-binding domain-containing proteins	GO:0006350 transcription	gg	t
envZ	CDS	sensory histidine kinase in two-component regulatory system with OmpR	Integral Membrane Protein	COG0642;Signal transduction histidine kinase	GO:0006464 protein modification	o	tt
epd	CDS	D-erythrose 4-phosphate dehydrogenase	Cytoplasmic	COG0057;Glyceraldehyde-3-phosphate dehydrogenase/erythrose-4-phosphate dehydrogenase	GO:0008615 pyridoxine biosynthesis -!- GO:0006094 gluconeogenesis	g	o
eptA	CDS	predicted metal dependent hydrolase	Integral Membrane Protein	COG2194;Predicted membrane-associated, metal-dependent hydrolase			
eptB	CDS	predicted metal dependent hydrolase	Integral Membrane Protein	COG2194;Predicted membrane-associated, metal-dependent hydrolase			
era	CDS	membrane-associated, 16S rRNA-binding GTPase	Cytoplasmic	COG1159;GTPase	GO:0007049 cell cycle	gg	t
erfK	CDS	conserved protein with NAD(P)-binding Rossmann-fold domain	Periplasmic	COG1376;Uncharacterized protein conserved in bacteria		o	tt
essD	CDS	DLP12 prophage; predicted phage lysis protein	Integral Membrane Protein			o	tt
essQ	CDS	Qin prophage; predicted S lysis protein	Integral Membrane Protein				
etp	CDS	phosphotyrosine-protein phosphatase	Cytoplasmic		GO:0006464 protein modification		
eutA	CDS	reactivating factor for ethanolamine ammonia lyase	Cytoplasmic	COG4819;Ethanolamine utilization protein, possible chaperonin protecting lyase from inhibition		gg	tt
eutB	CDS	ethanolamine ammonia-lyase, large subunit, heavy chain	Cytoplasmic		GO:0009310 amine catabolism	g	tt
eutC	CDS	ethanolamine ammonia-lyase, small subunit (light chain)	Cytoplasmic	COG4302;Ethanolamine ammonia-lyase, small subunit	GO:0009310 amine catabolism	gg	t
eutE	CDS	predicted aldehyde dehydrogenase, ethanolamine utilization protein	Cytoplasmic	COG1012;NAD-dependent aldehyde dehydrogenases	GO:0009310 amine catabolism	g	t
eutG	CDS	predicted alcohol dehydrogenase in ethanolamine utilization	Cytoplasmic		GO:0009310 amine catabolism	gg	tt
eutH	CDS	predicted inner membrane protein	Integral Membrane Protein	COG3192;Ethanolamine utilization protein	GO:0009310 amine catabolism	o	tt
eutI	CDS	predicted phosphotransacetylase subunit	Cytoplasmic	COG0280;Phosphotransacetylase	GO:0009310 amine catabolism	gg	t
eutJ	CDS	predicted chaperonin, ethanolamine utilization protein	Cytoplasmic	COG4820;Ethanolamine utilization protein, possible chaperonin	GO:0009310 amine catabolism -!- GO:0006457 protein folding	gg	t
eutL	CDS	predicted carboxysome structural protein with predicted role in ethanolamine utilization	Cytoplasmic	COG4816;Ethanolamine utilization protein	GO:0009310 amine catabolism	gg	t
eutP	CDS	conserved protein with nucleoside triphosphate hydrolase domain	Cytoplasmic	COG4917;Ethanolamine utilization protein	GO:0009310 amine catabolism	gg	t
eutQ	CDS	conserved protein	Cytoplasmic	COG4766;Ethanolamine utilization protein	GO:0009310 amine catabolism	gg	t
eutT	CDS	predicted cobalamin adenosyltransferase in ethanolamine utilization	Cytoplasmic	COG4812;Ethanolamine utilization cobalamin adenosyltransferase	GO:0009310 amine catabolism	g	tt
evgA	CDS	DNA-binding response regulator in two-component regulatory system with EvgS	Cytoplasmic	COG2197;Response regulator containing a CheY-like receiver domain and an HTH DNA-binding domain	GO:0006350 transcription	gg	o
evgS	CDS	hybrid sensory histidine kinase in two-component regulatory system with EvgA	Integral Membrane Protein	COG0834;ABC-type amino acid transport/signal transduction systems, periplasmic component/domain -!- COG0642;Signal transduction histidine kinase -!- COG0784;FOG: CheY-like receiver	GO:0006464 protein modification	o	tt
exbB	CDS	membrane spanning protein in TonB-ExbB-ExbD complex	Integral Membrane Protein	COG0811;Biopolymer transport proteins	GO:0006826 iron ion transport	o	tt
exbD	CDS	membrane spanning protein in TonB-ExbB-ExbD complex	Membrane Anchored	COG0848;Biopolymer transport protein	GO:0006826 iron ion transport	o	tt
exo	CDS	exonuclease IX (5'-3' exonuclease)	Cytoplasmic		GO:0006308 DNA catabolism -!- GO:0006308 DNA catabolism		
exoX	CDS	DNA exonuclease X	Cytoplasmic	COG0847;DNA polymerase III, epsilon subunit and related 3'-5' exonucleases	GO:0006308 DNA catabolism -!- GO:0006281 DNA repair -!- GO:0006308 DNA catabolism	g	t
exuR	CDS	DNA-binding transcriptional repressor	Cytoplasmic	COG2186;Transcriptional regulators	GO:0016052 carbohydrate catabolism -!- GO:0006350	o	tt
exuT	CDS	hexuronate transporter	Integral Membrane Protein	COG0477;Permeases of the major facilitator superfamily	GO:0016052 carbohydrate	o	tt
fabA	CDS	beta-hydroxydecanoyl thioester dehydrase	Cytoplasmic	COG0764;3-hydroxymyristoyl/3-hydroxydecanoyl-(acyl carrier protein) dehydratases		gg	t
fabB	CDS	3-oxoacyl-[acyl-carrier-protein] synthase I	Cytoplasmic	COG0304;3-oxoacyl-(acyl-carrier-protein) synthase		gg	tt
fabD	CDS	malonyl-CoA-[acyl-carrier-protein] transacylase	Cytoplasmic	COG0331;(acyl-carrier-protein) S-malonyltransferase		gg	o
fabF	CDS	3-oxoacyl-[acyl-carrier-protein] synthase II	Cytoplasmic	COG0304;3-oxoacyl-(acyl-carrier-protein) synthase		g	t
fabG	CDS	3-oxoacyl-[acyl-carrier-protein] reductase	Cytoplasmic	COG1028;Dehydrogenases with different specificities (related to short-chain alcohol dehydrogenases)		gg	o
fabH	CDS	3-oxoacyl-[acyl-carrier-protein] synthase III	Cytoplasmic	COG0332;3-oxoacyl-[acyl-carrier-protein] synthase III		o	tt
fabI	CDS	enoyl-[acyl-carrier-protein] reductase, NADH-dependent	Cytoplasmic	COG0623;Enoyl-[acyl-carrier-protein] reductase (NADH)		gg	o
fabR	CDS	DNA-binding transcriptional repressor	Cytoplasmic	COG1309;Transcriptional regulator		gg	t
fabZ	CDS	(3R)-hydroxymyristol acyl carrier protein dehydratase	Cytoplasmic	COG0764;3-hydroxymyristoyl/3-hydroxydecanoyl-(acyl carrier protein) dehydratases		gg	tt
fadA	CDS	3-ketoacyl-CoA thiolase (thiolase I)	Cytoplasmic		GO:0019395 fatty acid oxidation		
fadB	CDS	fused 3-hydroxybutyryl-CoA epimerase/delta(3)-cis-delta(2)-trans-enoyl-CoA isomerase/enoyl-CoA hydratase -!- 3-hydroxyacyl-CoA dehydrogenase	Cytoplasmic	COG1024;Enoyl-CoA hydratase/carnithine racemase -!- COG1250;3-hydroxyacyl-CoA dehydrogenase	GO:0019395 fatty acid oxidation	o	tt
fadD	CDS	acyl-CoA synthetase (long-chain-fatty-acid--CoA ligase)	Cytoplasmic	COG0318;Acyl-CoA synthetases (AMP-forming)/AMP-acid ligases II	GO:0019395 fatty acid oxidation -!- GO:0008654 phospholipid biosynthesis	o	o
fadE	CDS	acyl coenzyme A dehydrogenase	Integral Membrane Protein	COG1960;Acyl-CoA dehydrogenases	GO:0019395 fatty acid oxidation		
fadH	CDS	2,4-dienoyl-CoA reductase, NADH and FMN-linked	Cytoplasmic	COG1902;NADH:flavin oxidoreductases, Old Yellow Enzyme family -!- COG0446;Uncharacterized NAD(FAD)-dependent dehydrogenases	GO:0019395 fatty acid oxidation	o	t
fadL	CDS	long-chain fatty acid outer membrane transporter	Outer Membrane B-barrel protein	COG2067;Long-chain fatty acid transport protein	GO:0019395 fatty acid oxidation	o	tt
fadR	CDS	DNA-binding transcriptional dual regulator	Cytoplasmic	COG2186;Transcriptional regulators	GO:0019395 fatty acid oxidation -!- GO:0006350 transcription	gg	t
fbaA	CDS	fructose-bisphosphate aldolase, class II	Cytoplasmic	COG0191;Fructose/tagatose bisphosphate aldolase	GO:0016052 carbohydrate	gg	o
fbaB	CDS	fructose-bisphosphate aldolase class I	Cytoplasmic		GO:0006096 glycolysis		
fbp	CDS	fructose-1,6-bisphosphatase I	Cytoplasmic	COG0158;Fructose-1,6-bisphosphatase	GO:0006094 gluconeogenesis	gg	t
fcl	CDS	bifunctional GDP-fucose synthetase: GDP-4-dehydro-6-deoxy-D-mannose epimerase and GDP-4-dehydro-6-L-deoxygalactose reductase	Cytoplasmic	COG0451;Nucleoside-diphosphate-sugar epimerases	GO:0009242 colanic acid biosynthesis -!- GO:0009269 response to dessication	gg	t
fdhD	CDS	formate dehydrogenase formation protein	Cytoplasmic	COG1526;Uncharacterized protein required for formate dehydrogenase activity	GO:0009061 anaerobic respiration -!- GO:0006457 protein folding	g	t
fdhE	CDS	formate dehydrogenase formation protein	Cytoplasmic	COG3058;Uncharacterized protein involved in formate dehydrogenase formation	GO:0009061 anaerobic respiration -!- GO:0006457 protein folding	gg	o
fdhF	CDS	formate dehydrogenase-H, selenopolypeptide	Cytoplasmic	COG0243;Anaerobic dehydrogenases, typically selenocysteine-containing	GO:0006113 fermentation	o	t
fdnG	CDS	formate dehydrogenase-N, alpha subunit, nitrate-inducible	Periplasmic	COG0243;Anaerobic dehydrogenases, typically selenocysteine-containing	GO:0009061 anaerobic respiration	o	o
fdnH	CDS	formate dehydrogenase-N, Fe-S (beta) subunit, nitrate-inducible	Membrane Anchored	COG0437;Fe-S-cluster-containing hydrogenase components 1	GO:0009061 anaerobic respiration	o	tt
fdnI	CDS	formate dehydrogenase-N, cytochrome B556 (gamma) subunit, nitrate-inducible	Integral Membrane Protein	COG2864;Cytochrome b subunit of formate dehydrogenase	GO:0009061 anaerobic respiration -!- GO:0017004 cytochrome biogenesis	o	tt

fdoG	CDS	formate dehydrogenase-O, large subunit	Periplasmic	COG0243;Anaerobic dehydrogenases, typically selenocysteine-containing	GO:0009061 anaerobic respiration	o	o
fdoH	CDS	formate dehydrogenase-O, Fe-S subunit	Membrane Anchored	COG0437;Fe-S-cluster-containing hydrogenase components 1	GO:0009061 anaerobic respiration	o	tt
fdoI	CDS	formate dehydrogenase-O, cytochrome b556 subunit	Integral Membrane Protein	COG2864;Cytochrome b subunit of formate dehydrogenase	GO:0009061 anaerobic respiration -!- GO:0017004 cytochrome biogenesis	o	tt
fdrA	CDS	predicted acyl-CoA synthetase with NAD(P)-binding Rossmann-fold domain	Integral Membrane Protein	COG0074;Succinyl-CoA synthetase, alpha subunit		gg	t
fdx	CDS	[2Fe-2S] ferredoxin	Cytoplasmic	COG0633;Ferredoxin		gg	tt
feaB	CDS	phenylacetaldehyde dehydrogenase	Cytoplasmic		GO:0009310 amine catabolism -!- GO:0009435 nicotinamide adenine dinucleotide biosynthesis	o	tt
feaR	CDS	DNA-binding transcriptional regulator	Cytoplasmic	COG2207;AraC-type DNA-binding domain-containing proteins	GO:0009063 amino acid catabolism -!- GO:0009310 amine catabolism -!- GO:0006350 transcription	gg	t
fecA	CDS	KpLE2 phage-like element; ferric citrate outer membrane transporter	Outer Membrane B-barrel protein	COG4772;Outer membrane receptor for Fe3+-dicitrate	GO:0006826 iron ion transport	o	tt
fecB	CDS	KpLE2 phage-like element; iron-dicitrate transporter subunit -!- periplasmic-binding component of ABC superfamily	Periplasmic	COG4594;ABC-type Fe3+-citrate transport system, periplasmic component	GO:0006826 iron ion transport	o	tt
fecC	CDS	KpLE2 phage-like element; iron-dicitrate transporter subunit -!- membrane component of ABC superfamily	Integral Membrane Protein	COG0609;ABC-type Fe3+-siderophore transport system, permease component	GO:0006826 iron ion transport	o	tt
fecD	CDS	KpLE2 phage-like element; iron-dicitrate transporter subunit -!- membrane component of ABC superfamily	Integral Membrane Protein	COG0609;ABC-type Fe3+-siderophore transport system, permease component		o	tt
fecE	CDS	KpLE2 phage-like element; iron-dicitrate transporter subunit -!- ATP-binding component of ABC superfamily	Cytoplasmic	COG1120;ABC-type cobalamin/Fe3+-siderophores transport systems, ATPase components	GO:0006826 iron ion transport	g	t
fecI	CDS	KpLE2 phage-like element; RNA polymerase, sigma 19 factor	Cytoplasmic	COG1595;DNA-directed RNA polymerase specialized sigma subunit, sigma24 homolog	GO:0006350 transcription	gg	t
fecR	CDS	KpLE2 phage-like element; transmembrane signal transducer for ferric citrate transport	Integral Membrane Protein	COG3712;Fe2+-dicitrate sensor, membrane component	GO:0006350 transcription -!- GO:0006826 iron ion transport	o	t
feoA	CDS	ferrous iron transporter, protein A	Cytoplasmic	COG1918;Fe2+ transport system protein A	GO:0006826 iron ion transport	gg	o
feoB	CDS	fused ferrous iron transporter, protein B	Integral Membrane Protein	COG0370;Fe2+ transport system protein B	GO:0006826 iron ion transport	o	tt
fepA	CDS	iron-enterobactin outer membrane transporter	Outer Membrane B-barrel protein	COG4771;Outer membrane receptor for ferrienterochelin and colicins	GO:0006826 iron ion transport		
fepB	CDS	iron-enterobactin transporter subunit -!- periplasmic-binding component of ABC superfamily	Periplasmic	COG4592;ABC-type Fe2+-enterobactin transport system, periplasmic component	GO:0006826 iron ion transport	o	tt
fepC	CDS	iron-enterobactin transporter subunit -!- ATP-binding component of ABC superfamily	Cytoplasmic	COG1120;ABC-type cobalamin/Fe3+-siderophores transport systems, ATPase components	GO:0006826 iron ion transport	gg	t
fepD	CDS	iron-enterobactin transporter subunit -!- membrane component of ABC superfamily	Integral Membrane Protein	COG0609;ABC-type Fe3+-siderophore transport system, permease component	GO:0006826 iron ion transport	o	tt
fepE	CDS	regulator of length of O-antigen component of lipopolysaccharide chains	Integral Membrane Protein	COG3765;Chain length determinant protein	GO:0006826 iron ion transport	o	tt
fepG	CDS	iron-enterobactin transporter subunit -!- membrane component of ABC superfamily	Integral Membrane Protein	COG4779;ABC-type enterobactin transport system, permease component	GO:0006826 iron ion transport	o	tt
fes	CDS	enterobactin/ferric enterobactin esterase	Cytoplasmic	COG2382;Enterochelin esterase and related enzymes	GO:0006826 iron ion transport	gg	t
ffh	CDS	Signal Recognition Particle (SRP) component with 4.5S RNA (ffs)	Cytoplasmic	COG0541;Signal recognition particle GTPase			
ffs	misc_RNA	4.5S RNA					
fhiA	CDS	flagellar system protein, promoterless fragment (pseudogene)	Integral Membrane Protein	COG1298;Flagellar biosynthesis pathway, component FlhA			
fhlA	CDS	DNA-binding transcriptional activator	Cytoplasmic	COG3604;Transcriptional regulator containing GAF, AAA-type ATPase, and DNA binding domains	GO:0006113 fermentation -!- GO:0006350 transcription	gg	t
fhuA	CDS	ferrichrome outer membrane transporter	Outer Membrane B-barrel protein	COG1629;Outer membrane receptor proteins, mostly Fe transport		o	tt
fhuB	CDS	fused subunits of iron-hydroxamate transporter -!- membrane components of ABC superfamily transporter	Integral Membrane Protein	COG0609;ABC-type Fe3+-siderophore transport system, permease component		o	tt
fhuC	CDS	iron-hydroxamate transporter subunit -!- ATP-binding component of ABC superfamily	Cytoplasmic	COG1120;ABC-type cobalamin/Fe3+-siderophores transport systems, ATPase components		gg	o
fhuD	CDS	iron-hydroxamate transporter subunit -!- periplasmic-binding component of ABC superfamily	Periplasmic	COG0614;ABC-type Fe3+-hydroxamate transport system, periplasmic component	GO:0006826 iron ion transport	gg	t
fhuE	CDS	ferric-rhodotorulic acid outer membrane transporter	Outer Membrane B-barrel protein	COG4773;Outer membrane receptor for ferric coprogen and ferric-rhodotorulic acid	GO:0006826 iron ion transport	o	tt
fhuF	CDS	ferric iron reductase involved in ferric hydroximate transport	Cytoplasmic	COG4114;Uncharacterized Fe-S protein	GO:0006457 protein folding	o	tt
fic	CDS	stationary-phase protein, cell division	Cytoplasmic	COG2184;Protein involved in cell division		gg	tt
fieF	CDS	zinc transporter	Integral Membrane Protein	COG0053;Predicted Co/Zn/Cd cation transporters		o	tt
fimA	CDS	major type 1 subunit fimbrin (pilin)	Periplasmic	COG3539;P pilus assembly protein, pilin FimA	GO:0009101 glycoprotein biosynthesis	o	tt
fimB	CDS	tyrosine recombinase/inversion of on/off regulator of fimA	Cytoplasmic	COG0582;Integrase	GO:0009297 fimbrial biogenesis -!- GO:0006310 DNA recombination	o	tt
fimC	CDS	chaperone, periplasmic	Periplasmic		GO:0006457 protein folding	o	o
fimD	CDS	outer membrane usher protein, type 1 fimbrial synthesis	Outer Membrane B-barrel protein	COG3188;P pilus assembly protein, porin PapC			
fimE	CDS	tyrosine recombinase/inversion of on/off regulator of fimA	Cytoplasmic	COG0582;Integrase	GO:0009297 fimbrial biogenesis -!- GO:0006310 DNA recombination	o	t
fimF	CDS	minor component of type 1 fimbriae	Periplasmic	COG3539;P pilus assembly protein, pilin FimA	GO:0009101 glycoprotein biosynthesis	o	o
fimG	CDS	minor component of type 1 fimbriae	Periplasmic	COG3539;P pilus assembly protein, pilin FimA	GO:0009101 glycoprotein biosynthesis	o	t
fimH	CDS	minor component of type 1 fimbriae	Membrane Anchored		GO:0009101 glycoprotein biosynthesis	o	tt
fimI	CDS	fimbrial protein involved in type 1 pilus biosynthesis	Periplasmic	COG3539;P pilus assembly protein, pilin FimA	GO:0009101 glycoprotein biosynthesis		
fimZ	CDS	predicted DNA-binding transcriptional regulator	Cytoplasmic	COG2197;Response regulator containing a CheY-like receiver domain and an HTH DNA-binding domain	GO:0006350 transcription		
fis	CDS	global DNA-binding transcriptional dual regulator	Cytoplasmic	COG2901;Factor for inversion stimulation Fis, transcriptional activator	GO:0006310 DNA recombination	o	tt
fiu	CDS	predicted iron outer membrane transporter	Outer Membrane B-barrel protein	COG4774;Outer membrane receptor for monomeric catechols	GO:0006826 iron ion transport	o	tt
fixA	CDS	predicted electron transfer flavoprotein subunit, ETFP adenine nucleotide-binding domain	Cytoplasmic	COG2086;Electron transfer flavoprotein, beta subunit	GO:0016052 carbohydrate catabolism	gg	tt
fixB	CDS	predicted electron transfer flavoprotein, NAD/FAD-binding domain and ETFP adenine nucleotide-binding domain-like	Cytoplasmic	COG2025;Electron transfer flavoprotein, alpha subunit	GO:0016052 carbohydrate catabolism	gg	o
fixC	CDS	predicted oxidoreductase, FAD/NAD(P)-binding domain	Periplasmic	COG0644;Dehydrogenases (flavoproteins)	GO:0042413 carnitine catabolism	gg	tt
fixX	CDS	predicted 4Fe-4S ferredoxin-type protein	Cytoplasmic	COG2440;Ferredoxin-like protein	GO:0042413 carnitine catabolism	g	o
fklB	CDS	FKBP-type peptidyl-prolyl cis-trans isomerase (rotamase)	Cytoplasmic		GO:0006457 protein folding		
fkpA	CDS	FKBP-type peptidyl-prolyl cis-trans isomerase (rotamase)	Periplasmic	COG0545;FKBP-type peptidyl-prolyl cis-trans isomerases 1	GO:0006457 protein folding	o	tt
fkpB	CDS	FKBP-type peptidyl-prolyl cis-trans isomerase (rotamase)	Cytoplasmic	COG1047;FKBP-type peptidyl-prolyl cis-trans isomerases 2	GO:0006457 protein folding	gg	o
fldA	CDS	flavodoxin 1	Cytoplasmic	COG0716;Flavodoxins		gg	tt
fldB	CDS	flavodoxin 2	Cytoplasmic	COG0716;Flavodoxins	GO:0009060 aerobic respiration	gg	tt
flgA	CDS	assembly protein for flagellar basal-body periplasmic P ring	Periplasmic	COG1261;Flagellar basal body P-ring biosynthesis protein	GO:0009296 flagella biogenesis -!- GO:0042330 taxis	g	tt
flgB	CDS	flagellar component of cell-proximal portion of basal-body rod	Periplasmic	COG1815;Flagellar basal body protein	GO:0009296 flagella biogenesis -!- GO:0042330 taxis	gg	t
flgC	CDS	flagellar component of cell-proximal portion of basal-body rod	Periplasmic	COG1558;Flagellar basal body rod protein	GO:0009296 flagella biogenesis -!- GO:0042330 taxis	gg	o
flgD	CDS	flagellar hook assembly protein	Periplasmic	COG1843;Flagellar hook capping protein	GO:0009296 flagella biogenesis -!- GO:0042330 taxis	gg	o
flgE	CDS	flagellar hook protein	Cytoplasmic	COG1749;Flagellar hook protein FlgE	GO:0009296 flagella biogenesis -!- GO:0042330 taxis	gg	t
flgF	CDS	flagellar component of cell-proximal portion of basal-body rod	Periplasmic	COG4787;Flagellar basal body rod protein	GO:0009296 flagella biogenesis -!- GO:0042330 taxis	gg	o
flgG	CDS	flagellar component of cell-distal portion of basal-body rod	Periplasmic	COG4786;Flagellar basal body rod protein	GO:0009296 flagella biogenesis -!- GO:0042330 taxis	gg	o
flgH	CDS	flagellar protein of basal-body outer-membrane L ring	Outer Membrane Lipoprotein	COG2063;Flagellar basal body L-ring protein	GO:0009296 flagella biogenesis -!- GO:0042330 taxis		
flgI	CDS	predicted flagellar basal body protein	Periplasmic		GO:0009296 flagella biogenesis -!- GO:0042330 taxis	o	tt
flgJ	CDS	muramidase	Cytoplasmic	COG3951;Rod binding protein -!- COG1705;Muramidase (flagellum-specific)	GO:0009296 flagella biogenesis -!- GO:0042330 taxis	gg	t
flgK	CDS	flagellar hook-filament junction protein 1	Cytoplasmic	COG1256;Flagellar hook-associated protein	GO:0009296 flagella biogenesis -!- GO:0006457 protein folding -!- GO:0042330 taxis	gg	tt
flgL	CDS	flagellar hook-filament junction protein	Cytoplasmic	COG1344;Flagellin and related hook-associated proteins	GO:0009296 flagella biogenesis -!- GO:0006457 protein folding -!- GO:0042330 taxis	gg	t
flgM	CDS	anti-sigma factor for FliA (sigma 28)	Cytoplasmic	COG2747;Negative regulator of flagellin synthesis (anti-sigma28 factor)	GO:0006350 transcription -!- GO:0042330 taxis	gg	o
flgN	CDS	export chaperone for FlgK and FlgL	Cytoplasmic	COG3418;Flagellar biosynthesis/type III secretory pathway chaperone	GO:0009296 flagella biogenesis -!- GO:0006457 protein folding -!- GO:0042330 taxis	gg	o
flhA	CDS	predicted flagellar export pore protein	Integral Membrane Protein	COG1298;Flagellar biosynthesis pathway, component FlhA	GO:0009296 flagella biogenesis -!- GO:0042330 taxis	o	tt
flhB	CDS	predicted flagellar export pore protein	Integral Membrane Protein	COG1377;Flagellar biosynthesis pathway, component FlhB		o	tt
flhC	CDS	DNA-binding transcriptional regulator with FlhD	Cytoplasmic		GO:0009296 flagella biogenesis -!- GO:0006350 transcription -!- GO:0042330 taxis	gg	t

flhD	CDS	DNA-binding transcriptional dual regulator with FlhC	Cytoplasmic		GO:0009296 flagella biogenesis -!- GO:0006350 transcription -!- GO:0042330 taxis	gg	o
flhE	CDS	flagellar protein	Periplasmic		GO:0009296 flagella biogenesis -!- GO:0042330 taxis	o	tt
fliA	CDS	RNA polymerase, sigma 28 (sigma F) factor	Cytoplasmic		GO:0006350 transcription -!- GO:0042330 taxis	gg	tt
fliC	CDS	flagellar filament structural protein (flagellin)	Cytoplasmic	COG1344;Flagellin and related hook-associated proteins	GO:0009296 flagella biogenesis -!- GO:0042330 taxis	gg	tt
fliD	CDS	flagellar filament capping protein	Cytoplasmic	COG1345;Flagellar capping protein	GO:0009296 flagella biogenesis -!- GO:0042330 taxis	gg	t
fliE	CDS	flagellar basal-body component	Cytoplasmic	COG1677;Flagellar hook-basal body protein	GO:0009296 flagella biogenesis -!- GO:0042330 taxis	gg	o
fliF	CDS	flagellar basal-body MS-ring and collar protein	Integral Membrane Protein	COG1766;Flagellar biosynthesis/type III secretory pathway lipoprotein	GO:0009296 flagella biogenesis -!- GO:0042330 taxis	o	tt
fliG	CDS	flagellar motor switching and energizing component	Cytoplasmic	COG1536;Flagellar motor switch protein	GO:0009296 flagella biogenesis -!- GO:0042330 taxis	gg	t
fliH	CDS	flagellar biosynthesis protein	Cytoplasmic	COG1317;Flagellar biosynthesis/type III secretory pathway protein	GO:0009296 flagella biogenesis -!- GO:0042330 taxis	gg	tt
fliI	CDS	flagellum-specific ATP synthase	Cytoplasmic	COG1157;Flagellar biosynthesis/type III secretory pathway ATPase	GO:0015986 ATP synthesis coupled proton transport	gg	t
fliJ	CDS	flagellar protein	Cytoplasmic	COG2882;Flagellar biosynthesis chaperone	GO:0009296 flagella biogenesis -!- GO:0042330 taxis	gg	o
fliK	CDS	flagellar hook-length control protein	Cytoplasmic	COG3144;Flagellar hook-length control protein	GO:0009296 flagella biogenesis -!- GO:0042330 taxis	gg	o
fliL	CDS	flagellar biosynthesis protein	Outer Membrane Lipoprotein	COG1580;Flagellar basal body-associated protein	GO:0009296 flagella biogenesis -!- GO:0042330 taxis	o	tt
fliM	CDS	flagellar motor switching and energizing component	Cytoplasmic	COG1868;Flagellar motor switch protein	GO:0009296 flagella biogenesis -!- GO:0042330 taxis	gg	o
fliN	CDS	flagellar motor switching and energizing component	Cytoplasmic	COG1886;Flagellar motor switch/type III secretory pathway protein	GO:0009296 flagella biogenesis -!- GO:0042330 taxis	gg	o
fliO	CDS	flagellar biosynthesis protein	Membrane Anchored		GO:0009296 flagella biogenesis -!- GO:0042330 taxis		
fliP	CDS	flagellar biosynthesis protein	Integral Membrane Protein	COG1338;Flagellar biosynthesis pathway, component FliP	GO:0009296 flagella biogenesis -!- GO:0042330 taxis	o	tt
fliQ	CDS	flagellar biosynthesis protein	Integral Membrane Protein	COG1987;Flagellar biosynthesis pathway, component FliQ	GO:0009101 glycoprotein biosynthesis -!- GO:0009296 flagella biogenesis	o	tt
fliR	CDS	flagellar export pore protein	Integral Membrane Protein	COG1684;Flagellar biosynthesis pathway, component FliR		o	tt
fliS	CDS	flagellar protein potentiates polymerization	Cytoplasmic	COG1516;Flagellin-specific chaperone FliS	GO:0009296 flagella biogenesis -!- GO:0042330 taxis	gg	o
fliT	CDS	predicted chaperone	Cytoplasmic		GO:0009296 flagella biogenesis -!- GO:0006457 protein folding -!- GO:0042330 taxis	gg	o
fliY	CDS	cystine transporter subunit -!- periplasmic-binding component of ABC superfamily	Periplasmic	COG0834;ABC-type amino acid transport/signal transduction systems, periplasmic component/domain	GO:0019344 cysteine biosynthesis -!- GO:0042883 L-cysteine transport	o	tt
fliZ	CDS	predicted regulator of FliA activity	Cytoplasmic			gg	tt
flk	CDS	predicted flagella assembly protein	Membrane Anchored			o	tt
flu	CDS	CP4-44 prophage; antigen 43 (Ag43) phase-variable biofilm formation autotransporter	Outer Membrane B-barrel protein			o	tt
flxA	CDS	Qin prophage; predicted protein	Cytoplasmic			gg	o
fmt	CDS	10-formyltetrahydrofolate:L-methionyl-tRNA(fMet) N-formyltransferase	Cytoplasmic	COG0223;Methionyl-tRNA formyltransferase	GO:0009451 RNA modification	o	tt
fnr	CDS	DNA-binding transcriptional dual regulator, global regulator of anaerobic growth	Cytoplasmic	COG0664;cAMP-binding proteins - catabolite gene activator and regulatory subunit of cAMP-dependent protein kinases	GO:0009061 anaerobic respiration -!- GO:0006350 transcription	gg	t
focA	CDS	formate transporter	Integral Membrane Protein	COG2116;Formate/nitrite family of transporters	GO:0016052 carbohydrate	o	t
focB	CDS	predicted formate transporter	Integral Membrane Protein	COG2116;Formate/nitrite family of transporters		o	tt
folA	CDS	dihydrofolate reductase	Cytoplasmic	COG0262;Dihydrofolate reductase	GO:0046656 folic acid biosynthesis -!- GO:0009257 10-formyltetrahydrofolate biosynthesis	gg	tt
folB	CDS	bifunctioal dihydroneopterin aldolase -!- dihydroneopterin triphosphate 2'-epimerase	Cytoplasmic	COG1539;Dihydroneopterin aldolase	GO:0046656 folic acid biosynthesis	g	t
folC	CDS	bifunctional folylpolyglutamate synthase -!- dihydrofolate synthase	Cytoplasmic	COG0285;Folylpolyglutamate synthase	GO:0046656 folic acid biosynthesis -!- GO:0009257 10-formyltetrahydrofolate biosynthesis	gg	o
folD	CDS	bifunctional 5,10-methylene-tetrahydrofolate dehydrogenase -!- 5,10-methylene-tetrahydrofolate cyclohydrolase	Cytoplasmic	COG0190;5,10-methylene-tetrahydrofolate dehydrogenase/Methenyl tetrahydrofolate cyclohydrolase	GO:0046656 folic acid biosynthesis -!- GO:0009257 10-formyltetrahydrofolate biosynthesis	gg	t
folE	CDS	GTP cyclohydrolase I	Cytoplasmic	COG0302;GTP cyclohydrolase I	GO:0046656 folic acid biosynthesis	gg	o
folK	CDS	2-amino-4-hydroxy-6-hydroxymethyldihyropteridine pyrophosphokinase	Cytoplasmic	COG0801;7,8-dihydro-6-hydroxymethylpterin-pyrophos phokinase	GO:0046656 folic acid biosynthesis	gg	o
folM	CDS	dihydrofolate reductase isozyme	Cytoplasmic	COG1028;Dehydrogenases with different specificities (related to short-chain alcohol dehydrogenases)		gg	t
folP	CDS	7,8-dihydropteroate synthase	Cytoplasmic		GO:0046656 folic acid biosynthesis	gg	t
folX	CDS	D-erythro-7,8-dihydroneopterin triphosphate 2'-epimerase and dihydroneopterin aldolase	Cytoplasmic	COG1539;Dihydroneopterin aldolase		g	t
fpr	CDS	ferredoxin-NADP reductase	Cytoplasmic	COG1018;Flavodoxin reductases (ferredoxin-NADPH reductases) family 1	GO:0006805 xenobiotic metabolism	gg	o
frc	CDS	formyl-CoA transferase, NAD(P)-binding	Cytoplasmic	COG1804;Predicted acyl-CoA transferases/carnitine dehydratase		gg	t
frdA	CDS	fumarate reductase (anaerobic) catalytic and NAD/flavoprotein subunit	Cytoplasmic	COG1053;Succinate dehydrogenase/fumarate reductase, flavoprotein subunit	GO:0006113 fermentation -!- GO:0009061 anaerobic respiration	g	t
frdB	CDS	fumarate reductase (anaerobic), Fe-S subunit	Cytoplasmic	COG0479;Succinate dehydrogenase/fumarate reductase, Fe-S protein subunit	GO:0006113 fermentation -!- GO:0009061 anaerobic respiration	g	tt
frdC	CDS	fumarate reductase (anaerobic), membrane anchor subunit	Integral Membrane Protein	COG3029;Fumarate reductase subunit C	GO:0006113 fermentation -!- GO:0009061 anaerobic respiration	o	tt
frdD	CDS	fumarate reductase (anaerobic), membrane anchor subunit	Integral Membrane Protein	COG3080;Fumarate reductase subunit D	GO:0006113 fermentation -!- GO:0009061 anaerobic respiration	o	tt
fre	CDS	flavin reductase	Cytoplasmic	COG0543;2-polyprenylphenol hydroxylase and related flavodoxin oxidoreductases		gg	o
frlA	CDS	predicted fructoselysine transporter	Integral Membrane Protein	COG0531;Amino acid transporters		o	tt
frlB	CDS	fructoselysine-6-P-deglycase	Cytoplasmic	COG2222;Predicted phosphosugar isomerases			
frlC	CDS	predicted isomerase	Cytoplasmic				
frlD	CDS	fructoselysine 6-kinase	Cytoplasmic	COG0524;Sugar kinases, ribokinase family		o	tt
frlR	CDS	predicted DNA-binding transcriptional regulator	Cytoplasmic		GO:0006350 transcription		
frmA	CDS	alcohol dehydrogenase class III/glutathione-dependent formaldehyde dehydrogenase	Cytoplasmic	COG1062;Zn-dependent alcohol dehydrogenases, class III	GO:0006113 fermentation	gg	t
frmB	CDS	predicted esterase	Cytoplasmic	COG0627;Predicted esterase		gg	t
frmR	CDS	regulator protein that represses frmRAB operon	Cytoplasmic	COG1937;Uncharacterized protein conserved in bacteria		gg	t
frr	CDS	ribosome recycling factor	Cytoplasmic	COG0233;Ribosome recycling factor	GO:0006412 protein biosynthesis -!- GO:0009386 translational attenuation	o	tt
frsA	CDS	hydrolase, binds to enzyme IIA(Glc)	Cytoplasmic	COG1073;Hydrolases of the alpha/beta superfamily		gg	tt
fruA	CDS	fused fructose-specific PTS enzyme IIB'BC components	Integral Membrane Protein	COG1445;Phosphotransferase system fructose-specific component IIB -!- COG1299;Phosphotransferase system, fructose-specific IIC component	GO:0016052 carbohydrate catabolism	gg	tt
fruB	CDS	fused fructose-specific PTS enzyme IIA component -!- HPr component	Cytoplasmic	COG1925;Phosphotransferase system, HPr-related proteins	GO:0016052 carbohydrate catabolism	gg	t
fruK	CDS	fructose-1-phosphate kinase	Cytoplasmic	COG1105;Fructose-1-phosphate kinase and related fructose-6-phosphate kinase (PfkB)	GO:0006096 glycolysis	gg	t
fruR	CDS	DNA-binding transcriptional dual regulator	Cytoplasmic	COG1609;Transcriptional regulators	GO:0006096 glycolysis -!- GO:0006350 transcription	gg	tt
frvA	CDS	predicted enzyme IIA component of PTS	Cytoplasmic	COG1762;Phosphotransferase system mannitol/fructose-specific IIA domain (Ntr-type)	GO:0016052 carbohydrate catabolism	g	t
frvB	CDS	fused predicted PTS enzyme IIBC components	Integral Membrane Protein	COG1445;Phosphotransferase system fructose-specific component IIB -!- COG1299;Phosphotransferase system, fructose-specific IIC component			
frvR	CDS	predicted regulator	Cytoplasmic	COG3711;Transcriptional antiterminator -!- COG1762;Phosphotransferase system mannitol/fructose-specific IIA domain (Ntr-type)	GO:0006350 transcription	o	t
frvX	CDS	predicted endo-1,4-beta-glucanase	Cytoplasmic	COG1363;Cellulase M and related proteins	GO:0000272 polysaccharide catabolism	o	tt
frwB	CDS	predicted enzyme IIB component of PTS	Periplasmic	COG1445;Phosphotransferase system fructose-specific component IIB	GO:0016052 carbohydrate	gg	t
frwC	CDS	predicted enzyme IIC component of PTS	Integral Membrane Protein	COG1299;Phosphotransferase system, fructose-specific IIC component		o	tt
frwD	CDS	predicted enzyme IIB component of PTS	Periplasmic	COG1445;Phosphotransferase system fructose-specific component IIB	GO:0016052 carbohydrate	o	tt
fsaA	CDS	fructose-6-phosphate aldolase 1	Cytoplasmic				
fsaB	CDS	fructose-6-phosphate aldolase 2	Cytoplasmic	COG0176;Transaldolase		gg	o
fsr	CDS	predicted fosmidomycin efflux system	Integral Membrane Protein	COG0477;Permeases of the major facilitator superfamily	GO:0042493 response to drug	o	tt
ftn	CDS	ferritin iron storage protein (cytoplasmic)	Cytoplasmic	COG1528;Ferritin-like protein	GO:0006826 iron ion transport	o	o
ftsA	CDS	ATP-binding cell division protein involved in recruitment of FtsK to Z ring	Cytoplasmic	COG0849;Actin-like ATPase involved in cell division		o	tt
ftsB	CDS	cell division protein	Membrane Anchored	COG2919;Septum formation initiator		o	tt
ftsE	CDS	predicted transporter subunit -!- ATP-binding component of ABC superfamily	Cytoplasmic	COG2884;Predicted ATPase involved in cell division		o	tt
ftsH	CDS	protease, ATP-dependent zinc-metallo	Integral Membrane Protein	COG0465;ATP-dependent Zn proteases		o	tt
ftsI	CDS	transpeptidase involved in septal peptidoglycan synthesis (penicillin-binding protein 3)	Membrane Anchored	COG0768;Cell division protein FtsI/penicillin-binding protein 2	GO:0009252 peptidoglycan biosynthesis -!- GO:0042493 response to drug	o	tt
ftsK	CDS	DNA-binding membrane protein required for chromosome resolution and partitioning	Integral Membrane Protein	COG1674;DNA segregation ATPase FtsK/SpoIIIE and related proteins	GO:0009432 SOS response	o	tt
ftsL	CDS	membrane bound cell division protein at septum containing leucine zipper motif	Membrane Anchored	COG3116;Cell division protein		o	tt
ftsN	CDS	essential cell division protein	Membrane Anchored	COG3087;Cell division protein		o	tt
ftsQ	CDS	membrane anchored protein involved in growth of wall at septum	Membrane Anchored	COG1589;Cell division septal protein		o	tt
ftsW	CDS	integral membrane protein involved in stabilising FstZ ring during cell division	Integral Membrane Protein	COG0772;Bacterial cell division membrane protein		o	tt

ftsX	CDS	predicted transporter subunit -!- membrane component of ABC superfamily	Integral Membrane Protein	COG2177;Cell division protein		o	tt
ftsY	CDS	fused Signal Recognition Particle (SRP) receptor	Cytoplasmic	COG0552;Signal recognition particle GTPase		o	tt
ftsZ	CDS	GTP-binding tubulin-like cell division protein	Cytoplasmic	COG0206;Cell division GTPase		gg	tt
fucA	CDS	L-fuculose-1-phosphate aldolase	Cytoplasmic	COG0235;Ribulose-5-phosphate 4-epimerase and related epimerases and aldolases	GO:0016052 carbohydrate catabolism	gg	t
fucI	CDS	L-fucose isomerase	Cytoplasmic	COG2407;L-fucose isomerase and related proteins	GO:0016052 carbohydrate	gg	o
fucK	CDS	L-fuculokinase	Cytoplasmic	COG1070;Sugar (pentulose and hexulose) kinases	GO:0016052 carbohydrate	g	tt
fucO	CDS	L-1,2-propanediol oxidoreductase	Cytoplasmic	COG1454;Alcohol dehydrogenase, class IV	GO:0016052 carbohydrate catabolism -!- GO:0009441 glycolate	gg	tt
fucP	CDS	L-fucose transporter	Integral Membrane Protein	COG0738;Fucose permease	GO:0016052 carbohydrate	g	tt
fucR	CDS	DNA-binding transcriptional activator	Cytoplasmic	COG1349;Transcriptional regulators of sugar metabolism	GO:0016052 carbohydrate catabolism -!- GO:0006350	gg	t
fucU	CDS	L-fucose mutarotase	Cytoplasmic	COG4154;Fucose dissimilation pathway protein FucU		gg	o
fumA	CDS	aerobic Class I fumarate hydratase (fumarase A)	Cytoplasmic	COG1951;Tartrate dehydratase alpha subunit/Fumarate hydratase class I, N-terminal domain -!- COG1838;Tartrate dehydratase beta subunit/Fumarate hydratase class I, C-terminal domain	GO:0006099 tricarboxylic acid cycle	gg	tt
fumB	CDS	anaerobic class I fumarate hydratase (fumarase B)	Cytoplasmic	COG1951;Tartrate dehydratase alpha subunit/Fumarate hydratase class I, N-terminal domain -!- COG1838;Tartrate dehydratase beta subunit/Fumarate hydratase class I, C-terminal domain	GO:0006099 tricarboxylic acid cycle -!- GO:0006113 fermentation -!- GO:0009061 anaerobic respiration	gg	o
fumC	CDS	fumarate hydratase (fumarase C),aerobic Class II	Cytoplasmic	COG0114;Fumarase	GO:0006099 tricarboxylic acid cycle -!- GO:0006113 fermentation -!- GO:0009061 anaerobic respiration	gg	t
fur	CDS	DNA-binding transcriptional dual regulator	Cytoplasmic	COG0735;Fe2+/Zn2+ uptake regulation proteins	GO:0006350 transcription	o	tt
fusA	CDS	protein chain elongation factor EF-G	Cytoplasmic	COG0480;Translation elongation factors (GTPases)	GO:0006412 protein biosynthesis	gg	tt
fxsA	CDS	inner membrane protein	Integral Membrane Protein	COG3030;Protein affecting phage T7 exclusion by the F plasmid		o	tt
gabD	CDS	succinate-semialdehyde dehydrogenase I, NADP-dependent	Cytoplasmic	COG1012;NAD-dependent aldehyde dehydrogenases	GO:0009450 aminobutyrate catabolism -!- GO:0009447 putrescine catabolism	gg	t
gabP	CDS	gamma-aminobutyrate transporter	Integral Membrane Protein	COG1113;Gamma-aminobutyrate permease and related permeases	GO:0016052 carbohydrate	o	tt
gabT	CDS	4-aminobutyrate aminotransferase, PLP-dependent	Cytoplasmic	COG0160;4-aminobutyrate aminotransferase and related aminotransferases	GO:0009450 aminobutyrate catabolism -!- GO:0009447 putrescine catabolism	gg	t
gadA	CDS	glutamate decarboxylase A, PLP-dependent	Cytoplasmic	COG0076;Glutamate decarboxylase and related PLP-dependent proteins	GO:0009063 amino acid catabolism -!- GO:0006520 amino acid metabolism	gg	o
gadB	CDS	glutamate decarboxylase B, PLP-dependent	Cytoplasmic	COG0076;Glutamate decarboxylase and related PLP-dependent proteins	GO:0009063 amino acid catabolism -!- GO:0006520 amino acid metabolism	gg	t
gadC	CDS	predicted glutamate:gamma-aminobutyric acid antiporter	Integral Membrane Protein	COG0531;Amino acid transporters		gg	tt
gadE	CDS	DNA-binding transcriptional activator	Cytoplasmic	COG2771;DNA-binding HTH domain-containing proteins		g	t
gadW	CDS	DNA-binding transcriptional activator	Cytoplasmic	COG2207;AraC-type DNA-binding domain-containing proteins	GO:0006350 transcription	gg	t
gadX	CDS	DNA-binding transcriptional dual regulator	Cytoplasmic	COG2207;AraC-type DNA-binding domain-containing proteins	GO:0006350 transcription	o	tt
gadY	misc_RNA	small RNA					
galE	CDS	UDP-galactose-4-epimerase	Cytoplasmic		GO:0006012 galactose metabolism -!- GO:0009242 colanic acid biosynthesis -!- GO:0016052 carbohydrate catabolism -!- GO:0009435 nicotinamide adenine dinucleotide biosynthesis -!- GO:0009269 response to dessication	gg	o
galF	CDS	predicted subunit with GalU	Cytoplasmic	COG1210;UDP-glucose pyrophosphorylase	GO:0009226 nucleotide-sugar biosynthesis	gg	t
galK	CDS	galactokinase	Cytoplasmic	COG0153;Galactokinase	GO:0006012 galactose metabolism -!- GO:0009242 colanic acid biosynthesis -!- GO:0016052 carbohydrate catabolism -!- GO:0009269 response to dessication	gg	t
galM	CDS	galactose-1-epimerase (mutarotase)	Cytoplasmic	COG2017;Galactose mutarotase and related enzymes	GO:0016052 carbohydrate	gg	t
galP	CDS	D-galactose transporter	Integral Membrane Protein	COG0477;Permeases of the major facilitator superfamily	GO:0016052 carbohydrate	o	tt
galR	CDS	DNA-binding transcriptional repressor	Cytoplasmic	COG1609;Transcriptional regulators	GO:0016052 carbohydrate catabolism -!- GO:0006350	gg	tt
galS	CDS	DNA-binding transcriptional repressor	Cytoplasmic	COG1609;Transcriptional regulators	GO:0016052 carbohydrate catabolism -!- GO:0006350	gg	t
galT	CDS	galactose-1-phosphate uridylyltransferase	Cytoplasmic	COG1085;Galactose-1-phosphate uridylyltransferase	GO:0006012 galactose metabolism -!- GO:0009242 colanic acid biosynthesis -!- GO:0016052 carbohydrate catabolism -!- GO:0009269 response to dessication	gg	t
galU	CDS	glucose-1-phosphate uridylyltransferase	Cytoplasmic	COG1210;UDP-glucose pyrophosphorylase	GO:0006012 galactose metabolism -!- GO:0006006 glucose metabolism -!- GO:0009242 colanic acid biosynthesis -!- GO:0016052 carbohydrate catabolism -!- GO:0009269 response to dessication	gg	t
gapA	CDS	glyceraldehyde-3-phosphate dehydrogenase A	Cytoplasmic	COG0057;Glyceraldehyde-3-phosphate dehydrogenase/erythrose-4-phosphate dehydrogenase	GO:0006096 glycolysis -!- GO:0008615 pyridoxine biosynthesis -!- GO:0006094 gluconeogenesis	gg	t
gapC	CDS	glyceraldehyde-3-phosphate dehydrogenase C, N-ter fragment (pseudogene)	Cytoplasmic			gg	t
gapC	ancestral	glyceraldehyde-3-phosphate dehydrogenase C (pseudogene)	Cytoplasmic				
gapC	CDS	glyceraldehyde-3-phosphate dehydrogenase C, C-ter fragment (pseudogene)	Cytoplasmic				
garD	CDS	(D)-galactarate dehydrogenase	Cytoplasmic	COG2721;Altronate dehydratase	GO:0016052 carbohydrate	gg	t
garK	CDS	glycerate kinase I	Cytoplasmic		GO:0016052 carbohydrate	gg	tt
garL	CDS	alpha-dehydro-beta-deoxy-D-glucarate aldolase	Cytoplasmic	COG3836;2,4-dihydroxyhept-2-ene-1,7-dioic acid aldolase	GO:0016052 carbohydrate	gg	t
garP	CDS	predicted (D)-galactarate transporter	Integral Membrane Protein	COG0477;Permeases of the major facilitator superfamily	GO:0016052 carbohydrate	g	tt
garR	CDS	tartronate semialdehyde reductase	Cytoplasmic	COG2084;3-hydroxyisobutyrate dehydrogenase and related beta-hydroxyacid dehydrogenases	GO:0016052 carbohydrate catabolism -!- GO:0009441 glycolate		
gatA	CDS	galactitol-specific enzyme IIA component of PTS	Cytoplasmic	COG1762;Phosphotransferase system mannitol/fructose-specific IIA domain (Ntr-type)	GO:0016052 carbohydrate catabolism		
gatA	CDS	galactitol-specific enzyme IIA component of PTS	Cytoplasmic			o	o
gatA	CDS	galactitol-specific enzyme IIA component of PTS	Cytoplasmic			gg	t
gatB	CDS	galactitol-specific enzyme IIB component of PTS	Cytoplasmic	COG3414;Phosphotransferase system, galactitol-specific IIB component	GO:0016052 carbohydrate	gg	o
gatC	CDS	galactitol-specific enzyme IIC component of PTS	Integral Membrane Protein	COG3775;Phosphotransferase system, galactitol-specific IIC component	GO:0016052 carbohydrate	o	tt
gatD	CDS	galactitol-1-phosphate dehydrogenase, Zn-dependent and NAD(P)-binding	Cytoplasmic	COG1063;Threonine dehydrogenase and related Zn-dependent dehydrogenases	GO:0016052 carbohydrate catabolism	gg	t
gatR	CDS	DNA-binding transcriptional regulator, N-ter fragment (pseudogene)	Cytoplasmic				
gatR	ancestral	DNA-binding transcriptional regulator (pseudogene)	Cytoplasmic				
gatR	CDS	DNA-binding transcriptional regulator, C-ter fragment (pseudogene)	Cytoplasmic			o	tt
gatY	CDS	D-tagatose 1,6-bisphosphate aldolase 2, catalytic subunit	Cytoplasmic	COG0191;Fructose/tagatose bisphosphate aldolase	GO:0016052 carbohydrate catabolism		
gatZ	CDS	D-tagatose 1,6-bisphosphate aldolase 2, subunit	Cytoplasmic	COG4573;Predicted tagatose 6-phosphate kinase	GO:0016052 carbohydrate	g	tt
gcd	CDS	glucose dehydrogenase	Integral Membrane Protein	COG4993;Glucose dehydrogenase	GO:0006006 glucose metabolism -!- GO:0016052 carbohydrate	o	tt
gcl	CDS	glyoxylate carboligase	Cytoplasmic	COG3960;Glyoxylate carboligase	GO:0009441 glycolate metabolism -!- GO:0009442 allantoin assimilation	o	o
gcvA	CDS	DNA-binding transcriptional dual regulator	Cytoplasmic	COG0583;Transcriptional regulator	GO:0009063 amino acid catabolism -!- GO:0009257 10-formyltetrahydrofolate biosynthesis -!- GO:0006350 transcription	o	tt
gcvB	misc_RNA	regulatory sRNA					
gcvH	CDS	glycine cleavage complex lipoylprotein	Cytoplasmic	COG0509;Glycine cleavage system H protein (lipoate-binding)	GO:0009063 amino acid catabolism -!- GO:0009257 10-formyltetrahydrofolate biosynthesis	gg	t
gcvP	CDS	glycine decarboxylase, PLP-dependent, subunit (protein P) of glycine cleavage complex	Cytoplasmic	COG0403;Glycine cleavage system protein P (pyridoxal-binding), N-terminal domain -!- COG1003;Glycine cleavage system protein P (pyridoxal-binding), C-terminal domain	GO:0009063 amino acid catabolism -!- GO:0009257 10-formyltetrahydrofolate biosynthesis	gg	tt
gcvR	CDS	DNA-binding transcriptional repressor, regulatory protein accessory to GcvA	Cytoplasmic		GO:0009063 amino acid catabolism -!- GO:0009257 10-formyltetrahydrofolate biosynthesis -!- GO:0006350 transcription	o	t
gcvT	CDS	aminomethyltransferase, tetrahydrofolate-dependent, subunit (T protein) of glycine cleavage complex	Cytoplasmic	COG0404;Glycine cleavage system T protein (aminomethyltransferase)	GO:0009063 amino acid catabolism -!- GO:0009257 10-formyltetrahydrofolate biosynthesis	gg	o
gdhA	CDS	glutamate dehydrogenase, NADP-specific	Cytoplasmic	COG0334;Glutamate dehydrogenase/leucine dehydrogenase	GO:0006537 glutamate biosynthesis	gg	t
ggt	CDS	gamma-glutamyltranspeptidase	Periplasmic	COG0405;Gamma-glutamyltransferase		o	tt
gidA	CDS	glucose-inhibited cell-division protein	Cytoplasmic	COG0445;NAD/FAD-utilizing enzyme apparently involved in cell division		gg	t
gidB	CDS	methyltransferase, glucose-inhibited cell-division protein	Cytoplasmic	COG0357;Predicted S-adenosylmethionine-dependent methyltransferase involved in bacterial cell division		gg	o
glcB	CDS	malate synthase G	Cytoplasmic	COG2225;Malate synthase	GO:0016052 carbohydrate catabolism -!- GO:0009436 glyoxylate	gg	t
glcC	CDS	DNA-binding transcriptional dual regulator, glycolate-binding	Cytoplasmic	COG2186;Transcriptional regulators	GO:0016052 carbohydrate catabolism -!- GO:0006350	gg	t
glcD	CDS	glycolate oxidase subunit, FAD-linked	Cytoplasmic	COG0277;FAD/FMN-containing dehydrogenases	GO:0016052 carbohydrate	g	tt
glcE	CDS	glycolate oxidase FAD binding subunit	Cytoplasmic				

glcF	CDS	glycolate oxidase iron-sulfur subunit	Cytoplasmic				
glcG	CDS	conserved protein	Cytoplasmic	COG3193;Uncharacterized protein, possibly involved in utilization of glycolate and propanediol		gg	o
gldA	CDS	glycerol dehydrogenase, NAD	Cytoplasmic		GO:0006071 glycerol metabolism		
glf	CDS	UDP-galactopyranose mutase, FAD/NAD(P)-binding	Cytoplasmic	COG0562;UDP-galactopyranose mutase	GO:0009243 O antigen biosynthesis	gg	o
glgA	CDS	glycogen synthase	Cytoplasmic	COG0297;Glycogen synthase	GO:0000271 polysaccharide biosynthesis	g	t
glgB	CDS	1,4-alpha-glucan branching enzyme	Cytoplasmic	COG0296;1,4-alpha-glucan branching enzyme	GO:0000271 polysaccharide biosynthesis	o	tt
glgC	CDS	glucose-1-phosphate adenylyltransferase	Cytoplasmic	COG0448;ADP-glucose pyrophosphorylase	GO:0000271 polysaccharide biosynthesis	o	tt
glgP	CDS	glycogen phosphorylase	Cytoplasmic	COG0058;Glucan phosphorylase	GO:0016052 carbohydrate catabolism -!- GO:0000271 polysaccharide biosynthesis	o	tt
glgS	CDS	predicted glycogen synthesis protein	Cytoplasmic		GO:0000271 polysaccharide biosynthesis	gg	o
glgX	CDS	glycogen debranching enzyme	Cytoplasmic	COG1523;Type II secretory pathway, pullulanase PulA and related glycosidases	GO:0016052 carbohydrate catabolism -!- GO:0000271 polysaccharide biosynthesis	o	tt
glk	CDS	glucokinase	Cytoplasmic	COG0837;Glucokinase	GO:0016052 carbohydrate	gg	o
glmM	CDS	phosphoglucosamine mutase	Cytoplasmic	COG1109;Phosphomannomutase	GO:0009243 O antigen biosynthesis -!- GO:0009246 enterobacterial common antigen biosynthesis -!- GO:0006006 glucose metabolism -!- GO:0009252 peptidoglycan biosynthesis	gg	t
glmS	CDS	L-glutamine:D-fructose-6-phosphate aminotransferase	Cytoplasmic	COG0449;Glucosamine 6-phosphate synthetase, contains amidotransferase and phosphosugar isomerase domains	GO:0009243 O antigen biosynthesis -!- GO:0009252 peptidoglycan biosynthesis -!- GO:0046349 amino sugar biosynthesis	gg	o
glmU	CDS	fused N-acetyl glucosamine-1-phosphate uridyltransferase -!- glucosamine-1-phosphate acetyl transferase	Cytoplasmic	COG1207;N-acetylglucosamine-1-phosphate uridyltransferase (contains nucleotidyltransferase and I-patch acetyltransferase domains)	GO:0009243 O antigen biosynthesis -!- GO:0009252 peptidoglycan biosynthesis -!- GO:0009226 nucleotide-sugar biosynthesis -!- GO:0009246 enterobacterial common antigen biosynthesis -!- GO:0009310 amine catabolism	gg	tt
glnA	CDS	glutamine synthetase	Cytoplasmic	COG0174;Glutamine synthetase	GO:0006542 glutamine biosynthesis -!- GO:0006807 nitrogen metabolism	g	t
glnB	CDS	regulatory protein P-II for glutamine synthetase	Cytoplasmic	COG0347;Nitrogen regulatory protein PII	GO:0006542 glutamine biosynthesis -!- GO:0006350 transcription	o	tt
glnD	CDS	uridylyltransferase	Cytoplasmic	COG2844;UTP:GlnB (protein PII) uridylyltransferase	GO:0006807 nitrogen metabolism -!- GO:0006542 glutamine biosynthesis -!- GO:0006464 protein modification	gg	tt
glnE	CDS	fused deadenylyltransferase -!- adenylyltransferase for glutamine synthetase	Cytoplasmic	COG1391;Glutamine synthetase adenylyltransferase	GO:0006807 nitrogen metabolism -!- GO:0006464 protein modification	o	t
glnG	CDS	fused DNA-binding response regulator in two-component regulatory system with GlnL, nitrogen regulator I (NRI)	Cytoplasmic	COG2204;Response regulator containing CheY-like receiver, AAA-type ATPase, and DNA-binding domains	GO:0006542 glutamine biosynthesis -!- GO:0006350 transcription	o	t
glnH	CDS	glutamine transporter subunit -!- periplasmic binding component of ABC superfamily	Periplasmic	COG0834;ABC-type amino acid transport/signal transduction systems, periplasmic component/domain	GO:0006542 glutamine biosynthesis	o	tt
glnK	CDS	nitrogen assimilation regulatory protein for GlnL, GlnE, and AmtB	Cytoplasmic	COG0347;Nitrogen regulatory protein PII	GO:0006807 nitrogen metabolism	gg	tt
glnL	CDS	sensory kinase in two-component regulatory system with GlnG	Cytoplasmic	COG3852;Signal transduction histidine kinase, nitrogen specific	GO:0006542 glutamine biosynthesis -!- GO:0006464 protein modification	o	o
glnP	CDS	glutamine transporter subunit -!- membrane component of ABC superfamily	Integral Membrane Protein	COG0765;ABC-type amino acid transport system, permease component	GO:0006542 glutamine biosynthesis	o	tt
glnQ	CDS	glutamine transporter subunit -!- ATP-binding component of ABC superfamily	Cytoplasmic	COG1126;ABC-type polar amino acid transport system, ATPase component	GO:0006542 glutamine biosynthesis	g	t
glnS	CDS	glutamyl-tRNA synthetase	Cytoplasmic	COG0008;Glutamyl- and glutaminyl-tRNA synthetases	GO:0006418 amino acid activation	gg	tt
glnU	tRNA	tRNA-Gln(UUG) (Glutamine tRNA1)					
glnV	tRNA	tRNA-Gln(CUG) (Glutamine tRNA2)					
glnW	tRNA	tRNA-Gln(UUG) (Glutamine tRNA1)					
glnX	tRNA	tRNA-Gln(CUG) (Glutamine tRNA2)					
gloA	CDS	glyoxalase I, Ni-dependent	Cytoplasmic	COG0346;Lactoylglutathione lyase and related lyases	GO:0009438 methylglyoxal metabolism	gg	o
gloB	CDS	predicted hydroxyacylglutathione hydrolase	Cytoplasmic	COG0491;Zn-dependent hydrolases, including glyoxylases	GO:0009438 methylglyoxal metabolism	gg	o
glpA	CDS	sn-glycerol-3-phosphate dehydrogenase (anaerobic), large subunit, FAD/NAD(P)-binding	Cytoplasmic	COG0578;Glycerol-3-phosphate dehydrogenase	GO:0009061 anaerobic respiration -!- GO:0006071 glycerol metabolism	gg	t
glpB	CDS	sn-glycerol-3-phosphate dehydrogenase (anaerobic), membrane anchor subunit	Membrane Associated	COG3075;Anaerobic glycerol-3-phosphate dehydrogenase	GO:0009061 anaerobic respiration -!- GO:0006071 glycerol metabolism	gg	t
glpC	CDS	sn-glycerol-3-phosphate dehydrogenase (anaerobic), small subunit	Cytoplasmic	COG0247;Fe-S oxidoreductase	GO:0009061 anaerobic respiration -!- GO:0006071 glycerol metabolism	g	t
glpD	CDS	sn-glycerol-3-phosphate dehydrogenase, aerobic, FAD/NAD(P)-binding	Cytoplasmic	COG0578;Glycerol-3-phosphate dehydrogenase	GO:0009060 aerobic respiration -!- GO:0006071 glycerol metabolism	o	tt
glpE	CDS	thiosulfate:cyanide sulfurtransferase (rhodanese)	Cytoplasmic	COG0607;Rhodanese-related sulfurtransferase	GO:0009061 anaerobic respiration	gg	tt
glpF	CDS	glycerol facilitator	Integral Membrane Protein	COG0580;Glycerol uptake facilitator and related permeases (Major Intrinsic Protein Family)	GO:0006071 glycerol metabolism	o	tt
glpG	CDS	predicted intramembrane serine protease	Integral Membrane Protein				
glpK	CDS	glycerol kinase	Cytoplasmic	COG0554;Glycerol kinase	GO:0006071 glycerol metabolism	o	tt
glpQ	CDS	periplasmic glycerophosphodiester	Periplasmic	COG0584;Glycerophosphoryl diester phosphodiesterase	GO:0006071 glycerol metabolism	o	tt
glpR	CDS	DNA-binding transcriptional repressor	Cytoplasmic	COG1349;Transcriptional regulators of sugar metabolism	GO:0009061 anaerobic respiration -!- GO:0006350 transcription	o	tt
glpT	CDS	sn-glycerol-3-phosphate transporter	Integral Membrane Protein	COG2271;Sugar phosphate permease	GO:0009060 aerobic respiration -!- GO:0009061 anaerobic respiration -!- GO:0006071 glycerol metabolism	o	tt
glpX	CDS	fructose 1,6-bisphosphatase II	Cytoplasmic	COG1494;Fructose-1,6-bisphosphatase/sedoheptulose 1,7-bisphosphatase and related proteins	GO:0008652 amino acid biosynthesis	gg	t
gltA	CDS	citrate synthase	Cytoplasmic	COG0372;Citrate synthase	GO:0006099 tricarboxylic acid cycle -!- GO:0009061 anaerobic respiration	gg	t
gltB	CDS	glutamate synthase, large subunit	Cytoplasmic	COG0067;Glutamate synthase domain 1 -!- COG0069;Glutamate synthase domain 2 -!- COG0070;Glutamate synthase domain 3	GO:0006537 glutamate biosynthesis -!- GO:0006807 nitrogen metabolism	g	o
gltD	CDS	glutamate synthase, 4Fe-4S protein, small subunit	Cytoplasmic	COG0493;NADPH-dependent glutamate synthase beta chain and related oxidoreductases	GO:0006537 glutamate biosynthesis -!- GO:0006807 nitrogen metabolism	g	t
gltF	CDS	periplasmic protein	Periplasmic		GO:0006537 glutamate biosynthesis -!- GO:0006807 nitrogen metabolism -!- GO:0006355 regulation of transcription, DNA-dependent	o	tt
gltI	CDS	glutamate and aspartate transporter subunit -!- periplasmic-binding component of ABC superfamily	Periplasmic	COG0834;ABC-type amino acid transport/signal transduction systems, periplasmic component/domain	GO:0006537 glutamate biosynthesis		
gltJ	CDS	glutamate and aspartate transporter subunit -!- membrane component of ABC superfamily	Integral Membrane Protein	COG0765;ABC-type amino acid transport system, permease component	GO:0006537 glutamate biosynthesis -!- GO:0006532 aspartate	o	tt
gltK	CDS	glutamate and aspartate transporter subunit -!- membrane component of ABC superfamily	Integral Membrane Protein	COG0765;ABC-type amino acid transport system, permease component	GO:0006537 glutamate biosynthesis -!- GO:0006532 aspartate	o	tt
gltL	CDS	glutamate and aspartate transporter subunit -!- ATP-binding component of ABC superfamily	Cytoplasmic	COG1126;ABC-type polar amino acid transport system, ATPase component	GO:0006537 glutamate biosynthesis -!- GO:0006532 aspartate	g	t
gltP	CDS	glutamate/aspartate:proton symporter	Integral Membrane Protein	COG1301;Na+/H+-dicarboxylate symporters	GO:0006537 glutamate biosynthesis -!- GO:0006532 aspartate	o	tt
gltS	CDS	glutamate transporter	Integral Membrane Protein	COG0786;Na+/glutamate symporter	GO:0006537 glutamate biosynthesis -!- GO:0006532 aspartate	o	tt
gltT	tRNA	tRNA-Glu(UUC) (Glutamate tRNA2)					
gltU	tRNA	tRNA-Glu(UUC) (Glutamate tRNA2)					
gltV	tRNA	tRNA-Glu(UUC) (Glutamate tRNA2)					
gltW	tRNA	tRNA-Glu(UUC) (Glutamate tRNA2)					
gltX	CDS	glutamyl-tRNA synthetase	Cytoplasmic	COG0008;Glutamyl- and glutaminyl-tRNA synthetases	GO:0006418 amino acid activation	gg	tt
glvB	CDS	arbutin specific enzyme IIB component of PTS	Membrane Anchored			o	tt
glvC	CDS	arbutin sp ecific enzyme IIC component of PTS	Integral Membrane Protein			o	tt
glvG	CDS	predicted 6-phospho-beta-glucosidase (pseudogene)	Membrane Anchored	COG1486;Alpha-galactosidases/6-phospho-beta-glucos idases, family 4 of glycosyl hydrolases	GO:0016052 carbohydrate catabolism -!- GO:0009435 nicotinamide adenine dinucleotide	gg	t
glxK	CDS	glycerate kinase II	Cytoplasmic	COG1929;Glycerate kinase	GO:0016052 carbohydrate catabolism -!- GO:0009441 glycolate metabolism -!- GO:0009442 allantoin	g	tt
glxR	CDS	tartronate semialdehyde reductase, NADH-dependent	Cytoplasmic	COG2084;3-hydroxyisobutyrate dehydrogenase and related beta-hydroxyacid dehydrogenases	GO:0016052 carbohydrate catabolism -!- GO:0009441 glycolate metabolism -!- GO:0009442 allantoin	gg	t
glyA	CDS	serine hydroxymethyltransferase	Cytoplasmic	COG0112;Glycine/serine hydroxymethyltransferase	GO:0009063 amino acid catabolism -!- GO:0006545 glycine biosynthesis -!- GO:0046656 folic acid biosynthesis -!- GO:0009257 10-formyltetrahydrofolate biosynthesis	gg	t
glyQ	CDS	glycine tRNA synthetase, alpha subunit	Cytoplasmic	COG0752;Glycyl-tRNA synthetase, alpha subunit	GO:0006418 amino acid activation	g	t
glyS	CDS	glycine tRNA synthetase, beta subunit	Cytoplasmic	COG0751;Glycyl-tRNA synthetase, beta subunit	GO:0006418 amino acid activation	o	tt
glyT	tRNA	tRNA-Gly(UCC) (Glycine tRNA2)					
glyU	tRNA	tRNA-Gly(CCC) (Glycine tRNA1)					
glyV	tRNA	tRNA-Gly(GCC) (Glycine tRNA3)					
glyW	tRNA	tRNA-Gly(GCC) (Glycine tRNA3)					
glyX	tRNA	tRNA-Gly(GCC) (Glycine tRNA3)					
glyY	tRNA	tRNA-Gly(GCC) (Glycine tRNA3)					

gmd	CDS	GDP-D-mannose dehydratase, NAD(P)-binding	Cytoplasmic	COG1089;GDP-D-mannose dehydratase	GO:0009242 colanic acid biosynthesis -!- GO:0009435 nicotinamide adenine dinucleotide biosynthesis -!- GO:0009269	gg	t
gmhB	CDS	D,D-heptose 1,7-bisphosphate phosphatase	Cytoplasmic	COG0241;Histidinol phosphatase and related phosphatases		gg	tt
gmk	CDS	guanylate kinase	Cytoplasmic	COG0194;Guanylate kinase	GO:0015949 nucleobase, nucleoside and nucleotide interconversion -!- GO:0009152 purine ribonucleotide biosynthesis	gg	t
gmr	CDS	modulator of Rnase II stability	Cytoplasmic	COG2202;FOG: PAS/PAC domain -!- COG2200;FOG: EAL domain		o	t
gnd	CDS	gluconate-6-phosphate dehydrogenase, decarboxylating	Cytoplasmic	COG0362;6-phosphogluconate dehydrogenase	GO:0016052 carbohydrate catabolism -!- GO:0009051 pentose-phosphate shunt, oxidative branch -!- GO:0009052 pentose-phosphate shunt, non-oxidative branch	gg	o
gnsA	CDS	predicted regulator of phosphatidylethanolamine synthesis	Cytoplasmic			gg	t
gnsB	CDS	Qin prophage; predicted protein	Cytoplasmic				
gntK	CDS	gluconate kinase 2 -!- gluconate transport, GNT I system	Cytoplasmic	COG3265;Gluconate kinase	GO:0006006 glucose metabolism	gg	o
gntP	CDS	fructuronate transporter	Integral Membrane Protein	COG2610;H+/gluconate symporter and related permeases		o	tt
gntR	CDS	DNA-binding transcriptional repressor	Cytoplasmic		GO:0009255 Entner-Doudoroff pathway -!- GO:0006350 transcription		
gntT	CDS	gluconate transporter, high-affinity GNT I system	Integral Membrane Protein				
gntU	CDS	gluconate transporter, low affinity GNT 1 system	Integral Membrane Protein				
gntX	CDS	gluconate periplasmic binding protein with phosphoribosyltransferase domain, GNT I system	Cytoplasmic	COG1040;Predicted amidophosphoribosyltransferases			
gntY	CDS	predicted gluconate transport associated protein	Cytoplasmic	COG0316;Uncharacterized conserved protein -!- COG0694;Thioredoxin-like proteins and domains		gg	t
gor	CDS	glutathione oxidoreductase	Cytoplasmic	COG1249;Pyruvate/2-oxoglutarate dehydrogenase complex, dihydrolipoamide dehydrogenase (E3) component, and related enzymes		gg	t
gph	CDS	phosphoglycolate phosphatase	Cytoplasmic	COG0546;Predicted phosphatases	GO:0016052 carbohydrate	gg	tt
gpmA	CDS	phosphoglyceromutase 1	Cytoplasmic	COG0588;Phosphoglycerate mutase 1	GO:0006096 glycolysis -!- GO:0006094 gluconeogenesis	gg	t
gpmI	CDS	phosphoglycero mutase III, cofactor-independent	Cytoplasmic	COG0696;Phosphoglyceromutase	GO:0016052 carbohydrate	gg	tt
gpp	CDS	guanosine pentaphosphatase/exopolyphosphatase	Cytoplasmic		GO:0015949 nucleobase, nucleoside and nucleotide interconversion -!- GO:0006793 phosphorus metabolism -!- GO:0042594 response to		
gpsA	CDS	glycerol-3-phosphate dehydrogenase (NAD+)	Cytoplasmic	COG0240;Glycerol-3-phosphate dehydrogenase	GO:0006071 glycerol metabolism -!- GO:0006793 phosphorus metabolism	gg	t
gpt	CDS	guanine-hypoxanthine phosphoribosyltransferase	Cytoplasmic	COG0503;Adenine/guanine phosphoribosyltransferases and related PRPP-binding proteins	GO:0015949 nucleobase, nucleoside and nucleotide interconversion	gg	tt
greA	CDS	transcription elongation factor	Cytoplasmic		GO:0006350 transcription -!- GO:0006355 regulation of transcription, DNA-dependent	gg	tt
greB	CDS	transcription elongation factor	Cytoplasmic		GO:0006350 transcription -!- GO:0006355 regulation of transcription, DNA-dependent	gg	tt
groL	CDS	Cpn60 chaperonin GroEL, large subunit of GroESL	Cytoplasmic	COG0459;Chaperonin GroEL (HSP60 family)	GO:0006457 protein folding	o	tt
groS	CDS	Cpn10 chaperonin GroES, small subunit of GroESL	Cytoplasmic	COG0234;Co-chaperonin GroES (HSP10)	GO:0006457 protein folding	g	t
grpE	CDS	heat shock protein	Cytoplasmic	COG0576;Molecular chaperone GrpE (heat shock protein)	GO:0006457 protein folding	o	tt
grxA	CDS	glutaredoxin 1, redox coenzyme for ribonucleotide reductase (RNR1a)	Cytoplasmic	COG0695;Glutaredoxin and related proteins		g	o
grxB	CDS	glutaredoxin 2 (Grx2)	Cytoplasmic	COG2999;Glutaredoxin 2		gg	o
grxC	CDS	glutaredoxin 3	Cytoplasmic	COG0695;Glutaredoxin and related proteins		gg	o
gshA	CDS	gamma-glutamate-cysteine ligase	Cytoplasmic	COG2918;Gamma-glutamylcysteine synthetase	GO:0006750 glutathione biosynthesis	gg	tt
gshB	CDS	glutathione synthetase	Cytoplasmic	COG0189;Glutathione synthase/Ribosomal protein S6 modification enzyme (glutaminyl transferase)	GO:0006750 glutathione biosynthesis	gg	o
gsk	CDS	inosine/guanosine kinase	Cytoplasmic	COG0524;Sugar kinases, ribokinase family	GO:0015949 nucleobase, nucleoside and nucleotide interconversion	o	tt
gspA	CDS	general secretory pathway component, cryptic	Membrane Anchored			o	tt
gspC	CDS	general secretory pathway component, cryptic	Membrane Anchored	COG3031;Type II secretory pathway, component PulC		o	tt
gspD	CDS	general secretory pathway component, cryptic	Periplasmic				
gspE	CDS	general secretory pathway component, cryptic	Cytoplasmic	COG2804;Type II secretory pathway, ATPase PulE/Tfp pilus assembly pathway, ATPase PilB		o	t
gspF	CDS	general secretory pathway component, cryptic	Integral Membrane Protein	COG1459;Type II secretory pathway, component PulF		o	tt
gspG	CDS	pseudopilin, cryptic, general secretion pathway	Membrane Anchored	COG2165;Type II secretory pathway, pseudopilin PulG		o	tt
gspH	CDS	predicted general secretory pathway component, cryptic	Membrane Anchored	COG2165;Type II secretory pathway, pseudopilin PulG		o	tt
gspI	CDS	general secretory pathway component, cryptic	Periplasmic				
gspJ	CDS	predicted general secretory pathway component, cryptic	Periplasmic	COG4795;Type II secretory pathway, component PulJ		o	tt
gspK	CDS	general secretory pathway component, cryptic	Periplasmic	COG3156;Type II secretory pathway, component PulK		o	tt
gspL	CDS	general secretory pathway component, cryptic	Cytoplasmic	COG3297;Type II secretory pathway, component PulL			
gspM	CDS	general secretory pathway component, cryptic	Membrane Anchored				
gspO	CDS	bifunctional prepilin leader peptidase -!- methylase	Integral Membrane Protein	COG1989;Type II secretory pathway, prepilin signal peptidase PulO and related peptidases		o	tt
gss	CDS	fused glutathionylspermidine amidase -!- glutathionylspermidine synthetase	Membrane Anchored	COG0754;Glutathionylspermidine synthase	GO:0006596 polyamine biosynthesis	gg	t
gst	CDS	glutathionine S-transferase	Cytoplasmic	COG0625;Glutathione S-transferase		gg	t
guaA	CDS	GMP synthetase (glutamine aminotransferase)	Cytoplasmic	COG0518;GMP synthase - Glutamine amidotransferase domain -!- COG0519;GMP synthase, PP-ATPase domain/subunit	GO:0006164 purine nucleotide biosynthesis -!- GO:0015949 nucleobase, nucleoside and nucleotide interconversion	gg	t
guaB	CDS	IMP dehydrogenase	Cytoplasmic	COG0516;IMP dehydrogenase/GMP reductase -!- COG2524;Predicted transcriptional regulator, contains C-terminal CBS domains -!- COG0516;IMP dehydrogenase/GMP reductase	GO:0006164 purine nucleotide biosynthesis -!- GO:0015949 nucleobase, nucleoside and nucleotide interconversion		
guaC	CDS	GMP reductase	Cytoplasmic	COG0516;IMP dehydrogenase/GMP reductase	GO:0009152 purine ribonucleotide biosynthesis -!- GO:0015949 nucleobase, nucleoside and nucleotide interconversion	gg	o
guaD	CDS	guanine deaminase	Cytoplasmic	COG0402;Cytosine deaminase and related metal-dependent hydrolases			
gudD	CDS	(D)-glucarate dehydratase 1	Cytoplasmic	COG4948;L-alanine-DL-glutamate epimerase and related enzymes of enolase superfamily	GO:0016052 carbohydrate catabolism	gg	t
gudP	CDS	predicted D-glucarate transporter	Integral Membrane Protein	COG0477;Permeases of the major facilitator superfamily	GO:0016052 carbohydrate	g	tt
gudX	CDS	predicted glucarate dehydratase	Cytoplasmic	COG4948;L-alanine-DL-glutamate epimerase and related enzymes of enolase superfamily	GO:0016052 carbohydrate catabolism	gg	o
gutM	CDS	DNA-binding transcriptional activator	Periplasmic	COG4578;Glucitol operon activator	GO:0016052 carbohydrate	o	tt
gutQ	CDS	predicted phosphosugar-binding protein	Cytoplasmic	COG0794;Predicted sugar phosphate isomerase involved in capsule formation -!- COG0517;FOG: CBS domain			
gyrA	CDS	DNA gyrase (type II topoisomerase), subunit A	Cytoplasmic	COG0188;Type IIA topoisomerase (DNA gyrase/topo II, topoisomerase IV), A subunit	GO:0006261 DNA dependent DNA replication -!- GO:0006350 transcription	gg	tt
gyrB	CDS	DNA gyrase, subunit B	Cytoplasmic		GO:0006261 DNA dependent DNA replication -!- GO:0006350 transcription		
hcaB	CDS	2,3-dihydroxy-2,3-dihydrophenylpropionate dehydrogenase	Cytoplasmic	COG1028;Dehydrogenases with different specificities (related to short-chain alcohol dehydrogenases)	GO:0016052 carbohydrate catabolism	gg	t
hcaC	CDS	3-phenylpropionate dioxygenase, predicted ferredoxin subunit	Cytoplasmic	COG2146;Ferredoxin subunits of nitrite reductase and ring-hydroxylating dioxygenases	GO:0016052 carbohydrate catabolism	gg	t
hcaD	CDS	phenylpropionate dioxygenase, ferredoxin reductase subunit	Periplasmic	COG0446;Uncharacterized NAD(FAD)-dependent dehydrogenases	GO:0016052 carbohydrate catabolism	gg	t
hcaE	CDS	3-phenylpropionate dioxygenase, large (alpha) subunit	Cytoplasmic	COG4638;Phenylpropionate dioxygenase and related ring-hydroxylating dioxygenases, large terminal subunit	GO:0016052 carbohydrate catabolism	gg	t
hcaF	CDS	3-phenylpropionate dioxygenase, small (beta) subunit	Cytoplasmic		GO:0016052 carbohydrate	o	t
hcaR	CDS	DNA-binding transcriptional regulator	Cytoplasmic	COG0583;Transcriptional regulator	GO:0016052 carbohydrate catabolism -!- GO:0006350	g	t
hcaT	CDS	predicted 3-phenylpropionic transporter	Integral Membrane Protein	COG0477;Permeases of the major facilitator superfamily	GO:0042889 3-phenylpropionic acid transport	o	tt
hchA	CDS	Hsp31 molecular chaperone	Cytoplasmic	COG0693;Putative intracellular protease/amidase		gg	o
hcp	CDS	hybrid-cluster [4Fe-2S-2O] protein in anaerobic terminal reductases	Cytoplasmic			gg	t
hcr	CDS	HCP oxidoreductase, NADH-dependent	Cytoplasmic	COG1018;Flavodoxin reductases (ferredoxin-NADPH reductases) family 1			
hda	CDS	ATPase regulatory factor involved in DnaA inactivation	Cytoplasmic	COG0593;ATPase involved in DNA replication initiation	GO:0006261 DNA dependent DNA replication		
hdeA	CDS	stress response protein acid-resistance protein	Periplasmic			o	tt
hdeB	CDS	acid-resistance protein	Periplasmic				
hdeD	CDS	acid-resistance membrane protein	Integral Membrane Protein	COG3247;Uncharacterized conserved protein		o	tt
hdfR	CDS	DNA-binding transcriptional regulator	Cytoplasmic		GO:0006350 transcription		
hdhA	CDS	7alpha-hydroxysteroid dehydrogenase, NAD-dependent	Cytoplasmic	COG1028;Dehydrogenases with different specificities (related to short-chain alcohol dehydrogenases)		gg	t
helD	CDS	DNA helicase IV	Cytoplasmic	COG0210;Superfamily I DNA and RNA helicases	GO:0006261 DNA dependent DNA replication	gg	tt
hemA	CDS	glutamyl tRNA reductase	Cytoplasmic	COG0373;Glutamyl-tRNA reductase	GO:0042493 response to drug	o	tt
hemB	CDS	porphobilinogen synthase	Cytoplasmic			gg	t
hemC	CDS	hydroxymethylbilane synthase	Cytoplasmic				
hemD	CDS	uroporphyrinogen III synthase	Cytoplasmic	COG1587;Uroporphyrinogen-III synthase		o	t
hemE	CDS	uroporphyrinogen decarboxylase	Cytoplasmic	COG0407;Uroporphyrinogen-III decarboxylase		gg	t
hemF	CDS	coproporphyrinogen III oxidase	Cytoplasmic	COG0408;Coproporphyrinogen III oxidase		gg	o

hemG	CDS	protoporphyrin oxidase, flavoprotein	Cytoplasmic	COG4635;Flavodoxin		o	tt
hemH	CDS	ferrochelatase	Cytoplasmic	COG0276;Protoheme ferro-lyase (ferrochelatase)		o	tt
hemL	CDS	glutamate-1-semialdehyde aminotransferase (aminomutase)	Cytoplasmic	COG0001;Glutamate-1-semialdehyde aminotransferase		gg	t
hemN	CDS	coproporphyrinogen III oxidase, SAM and NAD(P)H dependent, oxygen-independent	Cytoplasmic	COG0635;Coproporphyrinogen III oxidase and related Fe-S oxidoreductases		gg	tt
hemX	CDS	uroporphyrinogen III methylase	Membrane Anchored	COG2959;Uncharacterized enzyme of heme biosynthesis		o	tt
hemY	CDS	predicted protoheme IX synthesis protein	Integral Membrane Protein	COG3071;Uncharacterized enzyme of heme biosynthesis		o	tt
hepA	CDS	RNA polymerase-associated helicase protein (ATPase and RNA polymerase recycling factor)	Cytoplasmic	COG0553;Superfamily II DNA/RNA helicases, SNF2 family	GO:0009451 RNA modification	o	tt
hflC	CDS	modulator for HflB protease specific for phage lambda cII repressor	Membrane Anchored	COG0330;Membrane protease subunits, stomatin/prohibitin homologs		o	tt
hflD	CDS	predicted lysogenization regulator	Cytoplasmic	COG2915;Uncharacterized protein involved in purine metabolism			
hflK	CDS	modulator for HflB protease specific for phage lambda cII repressor	Membrane Anchored	COG0330;Membrane protease subunits, stomatin/prohibitin homologs		o	tt
hflX	CDS	predicted GTPase	Cytoplasmic	COG2262;GTPases		g	o
hfq	CDS	HF-I, host factor for RNA phage Q beta replication	Cytoplasmic	COG1923;Uncharacterized host factor I protein	GO:0009386 translational attenuation	g	t
hha	CDS	modulator of gene expression, with H-NS	Cytoplasmic		GO:0006350 transcription	o	tt
hinT	CDS	purine nucleoside phosphoramidase	Cytoplasmic		GO:0006412 protein biosynthesis	gg	o
hipA	CDS	regulator with hipB	Cytoplasmic	COG3550;Uncharacterized protein related to capsule biosynthesis enzymes	GO:0009252 peptidoglycan biosynthesis -!- GO:0006259 DNA metabolism		
hipB	CDS	DNA-binding transcriptional regulator	Cytoplasmic	COG1396;Predicted transcriptional regulators	GO:0009252 peptidoglycan biosynthesis -!- GO:0006259 DNA metabolism	gg	tt
hisA	CDS	N-(5'-phospho-L-ribosyl-formimino)-5-amino-1-(5'-phosphoribosyl)-4-imidazolecarboxamide isomerase	Cytoplasmic	COG0106;Phosphoribosylformimino-5-aminoimidazole carboxamide ribonucleotide (ProFAR) isomerase	GO:0000105 histidine biosynthesis	gg	t
hisB	CDS	fused histidinol-phosphatase -!- imidazoleglycerol-phosphate dehydratase	Cytoplasmic	COG0241;Histidinol phosphatase and related phosphatases -!- COG0131;Imidazoleglycerol-phosphate dehydratase	GO:0000105 histidine biosynthesis	gg	t
hisC	CDS	histidinol-phosphate aminotransferase	Cytoplasmic	COG0079;Histidinol-phosphate/aromatic aminotransferase and cobyric acid decarboxylase	GO:0000105 histidine biosynthesis	o	o
hisD	CDS	bifunctional histidinal dehydrogenase -!- histidinol dehydrogenase	Cytoplasmic	COG0141;Histidinol dehydrogenase	GO:0000105 histidine biosynthesis	gg	t
hisF	CDS	imidazole glycerol phosphate synthase, catalytic subunit with HisF	Cytoplasmic	COG0107;Imidazoleglycerol-phosphate synthase	GO:0000105 histidine biosynthesis	gg	o
hisG	CDS	ATP phosphoribosyltransferase	Cytoplasmic	COG0040;ATP phosphoribosyltransferase	GO:0000105 histidine biosynthesis	gg	o
hisH	CDS	imidazole glycerol phosphate synthase, glutamine amidotransferase subunit	Cytoplasmic	COG0118;Glutamine amidotransferase	GO:0000105 histidine biosynthesis	gg	t
hisI	CDS	fused phosphoribosyl-AMP cyclohydrolase -!- phosphoribosyl-ATP pyrophosphatase	Cytoplasmic	COG0139;Phosphoribosyl-AMP cyclohydrolase -!- COG0140;Phosphoribosyl-ATP pyrophosphohydrolase	GO:0000105 histidine biosynthesis	gg	t
hisJ	CDS	histidine/lysine/arginine/ornithine transporter subunit -!- periplasmic-binding component of ABC superfamily	Periplasmic	COG0834;ABC-type amino acid transport/signal transduction systems, periplasmic component/domain	GO:0000105 histidine biosynthesis	o	tt
hisL	CDS	his operon leader peptide	Cytoplasmic		GO:0000105 histidine biosynthesis	gg	o
hisM	CDS	histidine/lysine/arginine/ornithine transporter subunit -!- membrane component of ABC superfamily	Integral Membrane Protein	COG4160;ABC-type arginine/histidine transport system, permease component	GO:0009089 lysine biosynthesis via diaminopimelate -!- GO:0006526 arginine biosynthesis	o	tt
hisP	CDS	histidine/lysine/arginine/ornithine transporter subunit -!- ATP-binding component of ABC superfamily	Cytoplasmic	COG4598;ABC-type histidine transport system, ATPase component	GO:0009089 lysine biosynthesis via diaminopimelate -!- GO:0006526 arginine biosynthesis -!- GO:0000105 histidine biosynthesis -!- GO:0009063 amino acid catabolism	gg	t
hisQ	CDS	histidine/lysine/arginine/ornithine transporter subunit -!- membrane component of ABC superfamily	Integral Membrane Protein	COG4215;ABC-type arginine transport system, permease component	GO:0009089 lysine biosynthesis via diaminopimelate -!- GO:0006526 arginine biosynthesis	o	tt
hisR	tRNA	tRNA-His(GUG) (Histidine tRNA)					
hisS	CDS	histidyl tRNA synthetase	Cytoplasmic	COG0124;Histidyl-tRNA synthetase	GO:0006418 amino acid activation	gg	o
hlpA	CDS	periplasmic chaperone	Periplasmic	COG2825;Outer membrane protein	GO:0006457 protein folding	o	tt
hlyE	CDS	hemolysin E	Extracellular				
hmp	CDS	fused nitric oxide dioxygenase -!- dihydropteridine reductase 2	Cytoplasmic	COG1017;Hemoglobin-like flavoprotein -!- COG1018;Flavodoxin reductases (ferredoxin-NADPH reductases) family 1		gg	o
hns	CDS	global DNA-binding transcriptional dual regulator H-NS	Cytoplasmic	COG2916;DNA-binding protein H-NS	GO:0006350 transcription -!- GO:0006355 regulation of transcription, DNA-dependent -!- GO:0042330 taxis	o	tt
hofB	CDS	conserved protein with nucleoside triphosphate hydrolase domain	Cytoplasmic	COG2804;Type II secretory pathway, ATPase PulE/Tfp pilus assembly pathway, ATPase PilB		gg	t
hofC	CDS	assembly protein in type IV pilin biogenesis, transmembrane protein	Integral Membrane Protein	COG1459;Type II secretory pathway, component PulF		o	tt
hofQ	CDS	predicted fimbrial transporter	Periplasmic	COG4796;Type II secretory pathway, component HofQ		o	tt
hokA	CDS	toxic polypeptide, small	Membrane Anchored			gg	t
hokB	CDS	toxic polypeptide, small	Membrane Anchored				
hokC	CDS	toxic membrane protein, small	Periplasmic				
hokD	CDS	Qin prophage; small toxic polypeptide	Periplasmic			o	tt
hokE	CDS	toxic polypeptide, small	Membrane Anchored				
holA	CDS	DNA polymerase III, delta subunit	Cytoplasmic	COG1466;DNA polymerase III, delta subunit	GO:0006261 DNA dependent DNA replication	gg	tt
holB	CDS	DNA polymerase III, delta prime subunit	Cytoplasmic	COG0470;ATPase involved in DNA replication	GO:0006261 DNA dependent DNA replication	o	tt
holC	CDS	DNA polymerase III, chi subunit	Cytoplasmic	COG2927;DNA polymerase III, chi subunit	GO:0006261 DNA dependent DNA replication	o	tt
holD	CDS	DNA polymerase III, psi subunit	Cytoplasmic	COG3050;DNA polymerase III, psi subunit	GO:0006261 DNA dependent DNA replication	gg	o
holE	CDS	DNA polymerase III, theta subunit	Cytoplasmic		GO:0006261 DNA dependent DNA replication	gg	o
hpt	CDS	hypoxanthine phosphoribosyltransferase	Cytoplasmic		GO:0015949 nucleobase, nucleoside and nucleotide interconversion		
hrpA	CDS	ATP-dependent helicase	Cytoplasmic		GO:0006261 DNA dependent DNA replication		
hrpB	CDS	predicted ATP-dependent helicase	Cytoplasmic	COG1643;HrpA-like helicases	GO:0006261 DNA dependent DNA replication	gg	t
hscA	CDS	DnaK-like molecular chaperone specific for IscU	Cytoplasmic	COG0443;Molecular chaperone	GO:0006457 protein folding	gg	tt
hscB	CDS	DnaJ-like molecular chaperone specific for IscU	Cytoplasmic	COG1076;DnaJ-domain-containing proteins 1	GO:0006457 protein folding	gg	tt
hscC	CDS	Hsp70 family chaperone Hsc62, binds to RpoD and inhibits transcription	Cytoplasmic	COG0443;Molecular chaperone	GO:0006457 protein folding	o	tt
hsdM	CDS	DNA methylase M	Cytoplasmic	COG0286;Type I restriction-modification system methyltransferase subunit	GO:0006306 DNA methylation	gg	t
hsdR	CDS	endonuclease R	Cytoplasmic	COG4096;Type I site-specific restriction-modification system, R (restriction) subunit and related helicases	GO:0006308 DNA catabolism -!- GO:0006308 DNA catabolism	o	tt
hsdS	CDS	specificity determinant for hsdM and hsdR	Cytoplasmic	COG0732;Restriction endonuclease S subunits	GO:0006306 DNA methylation	gg	o
hslJ	CDS	heat-inducible protein	Outer Membrane Lipoprotein	COG3187;Heat shock protein	GO:0009266 response to temperature	o	tt
hslO	CDS	heat shock protein Hsp33	Cytoplasmic	COG1281;Disulfide bond chaperones of the HSP33 family	GO:0006457 protein folding		
hslR	CDS	ribosome-associated heat shock protein Hsp15	Cytoplasmic	COG1188;Ribosome-associated heat shock protein implicated in the recycling of the 50S subunit (S4 paralog)	GO:0009266 response to temperature	gg	tt
hslU	CDS	molecular chaperone and ATPase component of HslUV protease	Cytoplasmic	COG1220;ATP-dependent protease HslVU (ClpYQ), ATPase subunit	GO:0006508 proteolysis and peptidolysis -!- GO:0006457 protein folding -!- GO:0009266 response to temperature	g	tt
hslV	CDS	peptidase component of the HslUV protease	Cytoplasmic	COG5405;ATP-dependent protease HslVU (ClpYQ), peptidase subunit	GO:0006508 proteolysis and peptidolysis -!- GO:0006457 protein folding	g	t
hsrA	CDS	predicted multidrug or homocysteine efflux system	Integral Membrane Protein	COG0477;Permeases of the major facilitator superfamily		o	tt
htgA	CDS	predicted protein	Cytoplasmic		GO:0006457 protein folding -!- GO:0006350 transcription -!- GO:0009266 response to		
htpG	CDS	molecular chaperone HSP90 family	Cytoplasmic	COG0326;Molecular chaperone, HSP90 family	GO:0006457 protein folding	gg	t
htpX	CDS	predicted endopeptidase	Integral Membrane Protein	COG0501;Zn-dependent protease with chaperone function	GO:0009266 response to	g	tt
htrC	CDS	heat shock protein	Cytoplasmic		GO:0009266 response to	o	tt
htrE	CDS	predicted outer membrane usher protein	Outer Membrane B-barrel protein	COG3188;P pilus assembly protein, porin PapC		o	tt
htrG	CDS	predicted signal transduction protein (SH3 domain)	Membrane Anchored	COG3103;SH3 domain protein		o	tt
htrL	CDS	predicted protein	Cytoplasmic		GO:0009103 lipopolysaccharide biosynthesis		
hupA	CDS	HU, DNA-binding transcriptional regulator, alpha subunit	Cytoplasmic	COG0776;Bacterial nucleoid DNA-binding protein		gg	o
hupB	CDS	HU, DNA-binding transcriptional regulator, beta subunit	Cytoplasmic	COG0776;Bacterial nucleoid DNA-binding protein		gg	t
hyaA	CDS	hydrogenase 1, small subunit	Integral Membrane Protein	COG1740;Ni,Fe-hydrogenase I small subunit	GO:0009060 aerobic respiration -!- GO:0009061 anaerobic respiration	gg	o
hyaB	CDS	hydrogenase 1, large subunit	Periplasmic	COG0374;Ni,Fe-hydrogenase I large subunit	GO:0009060 aerobic respiration -!- GO:0009061 anaerobic respiration	gg	t
hyaC	CDS	hydrogenase 1, b-type cytochrome subunit	Integral Membrane Protein	COG1969;Ni,Fe-hydrogenase I cytochrome b subunit	GO:0009060 aerobic respiration -!- GO:0009061 anaerobic respiration -!- GO:0017004 cytochrome biogenesis	o	tt
hyaD	CDS	protein involved in processing of HyaA and HyaB proteins	Cytoplasmic	COG0680;Ni,Fe-hydrogenase maturation factor	GO:0009060 aerobic respiration -!- GO:0009061 anaerobic respiration -!- GO:0006457 protein folding	gg	o
hyaE	CDS	protein involved in processing of HyaA and HyaB proteins	Cytoplasmic		GO:0009060 aerobic respiration -!- GO:0009061 anaerobic respiration -!- GO:0006457 protein folding	gg	o
hyaF	CDS	protein involved in nickel incorporation into hydrogenase-1 proteins	Cytoplasmic		GO:0009060 aerobic respiration -!- GO:0009061 anaerobic respiration -!- GO:0006457 protein folding	gg	tt

hybA	CDS	hydrogenase 2 4Fe-4S ferredoxin-type component	Periplasmic	COG0437;Fe-S-cluster-containing hydrogenase components 1		gg	t
hybB	CDS	predicted hydrogenase 2 cytochrome b type component	Integral Membrane Protein		GO:0009061 anaerobic respiration -!- GO:0017004 cytochrome biogenesis		
hybC	CDS	hydrogenase 2, large subunit	Periplasmic	COG0374;Ni,Fe-hydrogenase I large subunit	GO:0009061 anaerobic respiration	gg	t
hybD	CDS	predicted maturation element for hydrogenase 2	Cytoplasmic	COG0680;Ni,Fe-hydrogenase maturation factor	GO:0009061 anaerobic respiration	gg	tt
hybE	CDS	hydrogenase 2-specific chaperone	Cytoplasmic			gg	t
hybF	CDS	protein involved with the maturation of hydrogenases 1 and 2	Cytoplasmic	COG0375;Zn finger protein HypA/HybF (possibly regulating hydrogenase expression)	GO:0009061 anaerobic respiration -!- GO:0006457 protein folding		
hybG	CDS	hydrogenase 2 accessory protein	Cytoplasmic	COG0298;Hydrogenase maturation factor	GO:0009061 anaerobic respiration -!- GO:0006457 protein folding	gg	t
hybO	CDS	hydrogenase 2, small subunit	Membrane Anchored	COG1740;Ni,Fe-hydrogenase I small subunit	GO:0009061 anaerobic respiration -!- GO:0006457 protein folding	gg	t
hycA	CDS	regulator of the transcriptional regulator FhlA	Cytoplasmic		GO:0006113 fermentation -!- GO:0006350 transcription	gg	o
hycB	CDS	hydrogenase 3, Fe-S subunit	Cytoplasmic	COG1142;Fe-S-cluster-containing hydrogenase components 2	GO:0006113 fermentation -!- GO:0009061 anaerobic respiration	gg	t
hycC	CDS	hydrogenase 3, membrane subunit	Integral Membrane Protein	COG0651;Formate hydrogenlyase subunit 3/Multisubunit Na+/H+ antiporter, MnhD subunit	GO:0006113 fermentation	o	tt
hycD	CDS	hydrogenase 3, membrane subunit	Integral Membrane Protein	COG0650;Formate hydrogenlyase subunit 4	GO:0006113 fermentation	o	tt
hycE	CDS	hydrogenase 3, large subunit	Cytoplasmic	COG3262;Ni,Fe-hydrogenase III component G -!- COG3261;Ni,Fe-hydrogenase III large subunit	GO:0006113 fermentation -!- GO:0009061 anaerobic respiration	gg	t
hycF	CDS	formate hydrogenlyase complex iron-sulfur protein	Cytoplasmic	COG1143;Formate hydrogenlyase subunit 6/NADH:ubiquinone oxidoreductase 23 kD subunit (chain I)	GO:0006113 fermentation -!- GO:0009061 anaerobic respiration	gg	tt
hycG	CDS	hydrogenase 3 and formate hydrogenase complex, HycG subunit	Membrane Anchored	COG3260;Ni,Fe-hydrogenase III small subunit	GO:0006113 fermentation -!- GO:0009061 anaerobic respiration	o	t
hycH	CDS	protein required for maturation of hydrogenase 3	Cytoplasmic		GO:0006113 fermentation	gg	t
hycI	CDS	protease involved in processing C-terminal end of HycE	Cytoplasmic	COG0680;Ni,Fe-hydrogenase maturation factor	GO:0006457 protein folding	gg	t
hydN	CDS	formate dehydrogenase-H, [4Fe-4S] ferredoxin	Cytoplasmic	COG1142;Fe-S-cluster-containing hydrogenase components 2	GO:0009061 anaerobic respiration	g	t
hyfA	CDS	hydrogenase 4, 4Fe-4S subunit	Cytoplasmic		GO:0009061 anaerobic respiration	gg	t
hyfB	CDS	hydrogenase 4, membrane subunit	Integral Membrane Protein	COG0651;Formate hydrogenlyase subunit 3/Multisubunit Na+/H+ antiporter, MnhD subunit	GO:0009061 anaerobic respiration	o	tt
hyfC	CDS	hydrogenase 4, membrane subunit	Integral Membrane Protein		GO:0009061 anaerobic respiration	gg	tt
hyfD	CDS	hydrogenase 4, membrane subunit	Integral Membrane Protein	COG1009;NADH:ubiquinone oxidoreductase subunit 5 (chain L)/Multisubunit Na+/H+ antiporter, MnhA subunit	GO:0009061 anaerobic respiration	o	tt
hyfE	CDS	hydrogenase 4, membrane subunit	Integral Membrane Protein	COG4237;Hydrogenase 4 membrane component (E)	GO:0009061 anaerobic respiration	o	tt
hyfF	CDS	hydrogenase 4, membrane subunit	Integral Membrane Protein	COG0651;Formate hydrogenlyase subunit 3/Multisubunit Na+/H+ antiporter, MnhD subunit	GO:0009061 anaerobic respiration	g	tt
hyfG	CDS	hydrogenase 4, subunit	Cytoplasmic	COG3262;Ni,Fe-hydrogenase III component G -!- COG3261;Ni,Fe-hydrogenase III large subunit	GO:0009061 anaerobic respiration	g	t
hyfH	CDS	hydrogenase 4, Fe-S subunit	Cytoplasmic	COG1143;Formate hydrogenlyase subunit 6/NADH:ubiquinone oxidoreductase 23 kD subunit (chain I)	GO:0009061 anaerobic respiration	gg	t
hyfI	CDS	hydrogenase 4, Fe-S subunit	Cytoplasmic	COG3260;Ni,Fe-hydrogenase III small subunit	GO:0009061 anaerobic respiration		
hyfJ	CDS	predicted processing element hydrogenase 4	Cytoplasmic		GO:0006412 protein biosynthesis	g	t
hyfR	CDS	DNA-binding transcriptional activator, formate sensing	Cytoplasmic		GO:0009061 anaerobic respiration -!- GO:0006350 transcription	gg	t
hyi	CDS	hydroxypyruvate isomerase	Cytoplasmic	COG3622;Hydroxypyruvate isomerase		gg	o
hypA	CDS	protein involved in nickel insertion into hydrogenases 3	Cytoplasmic	COG0375;Zn finger protein HypA/HybF (possibly regulating hydrogenase expression)	GO:0009061 anaerobic respiration	g	o
hypB	CDS	GTP hydrolase involved in nickel liganding into hydrogenases	Cytoplasmic	COG0378;Ni2+-binding GTPase involved in regulation of expression and maturation of urease and hydrogenase	GO:0006113 fermentation -!- GO:0006457 protein folding	gg	t
hypC	CDS	protein required for maturation of hydrogenases 1 and 3	Cytoplasmic	COG0298;Hydrogenase maturation factor	GO:0009061 anaerobic respiration -!- GO:0006464 protein modification	gg	tt
hypD	CDS	protein required for maturation of hydrogenases	Cytoplasmic	COG0409;Hydrogenase maturation factor	GO:0009061 anaerobic respiration -!- GO:0006464 protein modification	gg	t
hypE	CDS	carbamoyl phosphate phosphatase, hydrogenase 3 maturation protein	Cytoplasmic	COG0309;Hydrogenase maturation factor	GO:0009061 anaerobic respiration -!- GO:0006457 protein folding	gg	t
hypF	CDS	carbamoyl phosphate phosphatase and maturation protein for [NiFe] hydrogenases	Cytoplasmic	COG0068;Hydrogenase maturation factor	GO:0009061 anaerobic respiration -!- GO:0006457 protein folding		
hyuA	CDS	D-stereospecific phenylhydantoinase	Cytoplasmic			gg	t
iaaA	CDS	L-asparaginase	Cytoplasmic	COG1446;Asparaginase	GO:0009063 amino acid catabolism	gg	t
iadA	CDS	isoaspartyl dipeptidase	Cytoplasmic			g	t
iap	CDS	aminopeptidase in alkaline phosphatase isozyme conversion	Periplasmic	COG2234;Predicted aminopeptidases	GO:0006464 protein modification	o	tt
ibpA	CDS	heat shock chaperone	Cytoplasmic	COG0071;Molecular chaperone (small heat shock protein)	GO:0009266 response to	g	o
ibpB	CDS	heat shock chaperone	Cytoplasmic	COG0071;Molecular chaperone (small heat shock protein)	GO:0009266 response to	g	o
icd	CDS	e14 prophage; isocitrate dehydrogenase, specific for NADP+	Cytoplasmic	COG0538;Isocitrate dehydrogenases	GO:0006099 tricarboxylic acid cycle -!- GO:0009061 anaerobic respiration	gg	o
icdC	CDS	conserved protein (pseudogene)	Cytoplasmic				
iclR	CDS	DNA-binding transcriptional repressor	Cytoplasmic	COG1414;Transcriptional regulator	GO:0006097 glyoxylate cycle -!- GO:0006350 transcription	o	tt
idi	CDS	isopentenyl diphosphate isomerase	Cytoplasmic	COG1443;Isopentenyldiphosphate isomerase		gg	o
idnD	CDS	L-idonate 5-dehydrogenase, NAD-binding	Cytoplasmic	COG1063;Threonine dehydrogenase and related Zn-dependent dehydrogenases	GO:0016052 carbohydrate catabolism	o	tt
idnK	CDS	D-gluconate kinase, thermosensitive	Cytoplasmic	COG3265;Gluconate kinase	GO:0016052 carbohydrate	gg	o
idnO	CDS	5-keto-D-gluconate-5-reductase	Cytoplasmic	COG1028;Dehydrogenases with different specificities (related to short-chain alcohol dehydrogenases)	GO:0016052 carbohydrate catabolism	o	o
idnR	CDS	DNA-binding transcriptional repressor, 5-gluconate-binding	Cytoplasmic	COG1609;Transcriptional regulators	GO:0016052 carbohydrate catabolism -!- GO:0006350	o	o
idnT	CDS	L-idonate and D-gluconate transporter	Integral Membrane Protein	COG2610;H+/gluconate symporter and related permeases		o	tt
ihfA	CDS	integration host factor (IHF), DNA-binding protein, alpha subunit	Cytoplasmic	COG0776;Bacterial nucleoid DNA-binding protein	GO:0006310 DNA recombination	gg	o
ihfB	CDS	integration host factor (IHF), DNA-binding protein, beta subunit	Cytoplasmic	COG0776;Bacterial nucleoid DNA-binding protein	GO:0006310 DNA recombination	gg	o
ileS	CDS	isoleucyl-tRNA synthetase	Cytoplasmic	COG0060;Isoleucyl-tRNA synthetase	GO:0006418 amino acid activation	o	tt
ileT	tRNA	tRNA-Ile(GAU) (Isoleucine tRNA1)					
ileU	tRNA	tRNA-Ile(GAU) (Isoleucine tRNA1)					
ileV	tRNA	tRNA-Ile(GAU) (Isoleucine tRNA1)					
ileX	tRNA	tRNA-Ile(CAU) (Isoleucine tRNA2)					
ileY	tRNA	tRNA-Ile(CAU) (Isoleucine tRNA2 variant)					
ilvA	CDS	threonine deaminase	Cytoplasmic	COG1171;Threonine dehydratase		g	t
ilvB	CDS	acetolactate synthase I, large subunit	Cytoplasmic	COG0028;Thiamine pyrophosphate-requiring enzymes [acetolactate synthase, pyruvate dehydrogenase (cytochrome), glyoxylate carboligase, phosphonopyruvate decarboxylase]		gg	t
ilvC	CDS	ketol-acid reductoisomerase, NAD(P)-binding	Cytoplasmic	COG0059;Ketol-acid reductoisomerase		o	tt
ilvD	CDS	dihydroxyacid dehydratase	Cytoplasmic				
ilvE	CDS	branched-chain amino-acid aminotransferase	Cytoplasmic		GO:0006523 alanine biosynthesis -!- GO:0009098 leucine biosynthesis		
ilvG	CDS	acetolactate synthase II, large subunit, N-ter fragment (pseudogene)	Cytoplasmic			g	t
ilvG	ancestral	large subunit of acetolactate synthase II	Cytoplasmic				
ilvG	CDS	acetolactate synthase II, large subunit, C-ter fragment (pseudogene)	Cytoplasmic			g	o
ilvH	CDS	acetolactate synthase III, thiamin-dependent, small subunit	Cytoplasmic	COG0440;Acetolactate synthase, small (regulatory) subunit		gg	o
ilvI	CDS	acetolactate synthase III, large subunit	Cytoplasmic			gg	t
ilvL	CDS	ilv operon leader peptide	Cytoplasmic			o	t
ilvM	CDS	acetolactate synthase II, small subunit	Cytoplasmic	COG3978;Acetolactate synthase (isozyme II), small (regulatory) subunit		gg	t
ilvN	CDS	acetolactate synthase I, small subunit	Cytoplasmic	COG0440;Acetolactate synthase, small (regulatory) subunit		g	o
ilvY	CDS	DNA-binding transcriptional dual regulator	Cytoplasmic	COG0583;Transcriptional regulator	GO:0006350 transcription	o	tt
imp	CDS	exported protein required for envelope biosynthesis and integrity	Outer Membrane B-barrel protein	COG1452;Organic solvent tolerance protein OstA		o	tt
inaA	CDS	conserved protein	Cytoplasmic			gg	o
infA	CDS	translation initiation factor IF-1	Cytoplasmic	COG0361;Translation initiation factor 1 (IF-1)	GO:0006412 protein biosynthesis	gg	o
infB	CDS	fused protein chain initiation factor 2, IF2	Cytoplasmic	COG0532;Translation initiation factor 2 (IF-2; GTPase)	GO:0006412 protein biosynthesis	o	tt
infC	CDS	protein chain initiation factor IF-3	Cytoplasmic	COG0290;Translation initiation factor 3 (IF-3)	GO:0006412 protein biosynthesis		
insA	CDS	IS1 element protein	Cytoplasmic				
insA	CDS	IS1 repressor protein InsA	Cytoplasmic	COG3677;Transposase and inactivated derivatives			
insA	CDS	CP4-6 prophage; IS1 repressor protein InsA	Cytoplasmic	COG3677;Transposase and inactivated derivatives			
insA	CDS	CP4-6 prophage; IS1 repressor protein InsA	Cytoplasmic	COG3677;Transposase and inactivated derivatives			
insA	CDS	IS1 repressor protein InsA	Cytoplasmic				
insA	CDS	IS1 repressor protein InsA	Cytoplasmic	COG3677;Transposase and inactivated derivatives			
insA	CDS	IS1 repressor protein InsA	Cytoplasmic	COG3677;Transposase and inactivated derivatives			
insA	CDS	KpLE2 phage-like element; IS1 repressor protein	Cytoplasmic				
insB	CDS	IS1 element protein	Cytoplasmic				
insB	CDS	IS1 transposase InsAB'	Cytoplasmic	COG1662;Transposase and inactivated derivatives, IS1 family			
insB	CDS	CP4-6 prophage; IS1 transposase InsAB'	Cytoplasmic	COG1662;Transposase and inactivated derivatives, IS1 family			
insB	CDS	CP4-6 prophage; IS1 transposase InsAB'	Cytoplasmic	COG1662;Transposase and inactivated derivatives, IS1 family			
insB	CDS	IS1 transposase InsAB'	Cytoplasmic	COG1662;Transposase and inactivated derivatives, IS1 family			
insB	CDS	IS1 transposase InsAB'	Cytoplasmic	COG1662;Transposase and inactivated derivatives, IS1 family			
insB	CDS	IS1 transposase InsAB'	Cytoplasmic				
insB	CDS	IS1 transposase InsAB', N-ter fragment	Cytoplasmic				
insB	ancestral	IS1 transposase InsAB' (pseudogene)	Cytoplasmic				
insB	CDS	IS1 transposase InsAB', C-ter fragment	Cytoplasmic				
insC	CDS	IS2 element protein	Cytoplasmic				
insC	CDS	IS2 element protein	Cytoplasmic				
insC	CDS	IS2 element protein	Cytoplasmic				
insC	CDS	IS2 element protein	Cytoplasmic				
insC	CDS	IS2 insertion element repressor InsA	Cytoplasmic	COG2963;Transposase and inactivated derivatives			
insC	CDS	IS2 insertion element repressor InsA	Cytoplasmic	COG2963;Transposase and inactivated derivatives			

insC	CDS	CP4-44 prophage; IS2 insertion element repressor InsA	Cytoplasmic				
insC	CDS	IS2 insertion element repressor InsA	Cytoplasmic	COG2963;Transposase and inactivated derivatives			
insC	CDS	IS2 insertion element repressor InsA	Cytoplasmic	COG2963;Transposase and inactivated derivatives			
insC	CDS	KpLE2 phage-like element; IS2 insertion element repressor InsA	Cytoplasmic	COG2963;Transposase and inactivated derivatives			
insD	CDS	IS2 element protein	Cytoplasmic				
insD	CDS	IS2 element protein	Cytoplasmic				
insD	CDS	IS2 element protein	Cytoplasmic				
insD	CDS	IS2 element protein	Cytoplasmic				
insD	CDS	IS2 insertion element transposase InsAB'	Cytoplasmic	COG2801;Transposase and inactivated derivatives			
insD	CDS	IS2 insertion element transposase InsAB'	Cytoplasmic	COG2801;Transposase and inactivated derivatives			
insD	CDS	Qin prophage; predicted transposase	Cytoplasmic	COG2801;Transposase and inactivated derivatives			
insD	CDS	CP4-44 prophage; IS2 insertion element transposase InsAB'	Cytoplasmic	COG2801;Transposase and inactivated derivatives			
insD	CDS	IS2 insertion element transposase InsAB'	Cytoplasmic	COG2801;Transposase and inactivated derivatives			
insD	CDS	IS2 insertion element transposase InsAB'	Cytoplasmic	COG2801;Transposase and inactivated derivatives			
insD	CDS	KpLE2 phage-like element; IS2 insertion element transposase InsAB'	Cytoplasmic	COG2801;Transposase and inactivated derivatives			
insE	CDS	IS3 element protein	Cytoplasmic	COG2963;Transposase and inactivated derivatives			
insE	CDS	IS3 element protein	Cytoplasmic	COG2963;Transposase and inactivated derivatives			
insE	CDS	DLP12 prophage; IS3 element protein InsE	Cytoplasmic	COG2963;Transposase and inactivated derivatives			
insE	CDS	IS3 element protein	Cytoplasmic	COG2963;Transposase and inactivated derivatives			
insE	CDS	IS3 element protein InsE	Cytoplasmic	COG2963;Transposase and inactivated derivatives			
insF	CDS	IS3 element protein InsF	Cytoplasmic	COG2801;Transposase and inactivated derivatives			
insF	CDS	IS3 element protein InsF	Cytoplasmic	COG2801;Transposase and inactivated derivatives			
insF	CDS	DLP12 prophage; IS3 element protein InsF	Cytoplasmic	COG2801;Transposase and inactivated derivatives			
insF	CDS	IS3 element protein InsF	Cytoplasmic	COG2801;Transposase and inactivated derivatives			
insF	CDS	IS3 element protein InsF	Cytoplasmic	COG2801;Transposase and inactivated derivatives			
insG	CDS	KpLE2 phage-like element; IS4 predicted	Cytoplasmic	COG3385;FOG: Transposase and inactivated derivatives			
insH	CDS	IS5 element protein	Cytoplasmic				
insH	CDS	IS5 element protein	Cytoplasmic				
insH	CDS	IS5 element protein	Cytoplasmic				
insH	CDS	IS5 element protein	Cytoplasmic				
insH	CDS	IS5 element protein	Cytoplasmic				
insH	CDS	IS5 element protein	Cytoplasmic				
insH	CDS	IS5 element protein	Cytoplasmic				
insH	CDS	CP4-6 prophage; IS5 transposase and trans-	Cytoplasmic	COG3039;Transposase and inactivated derivatives, IS5 family			
insH	CDS	DLP12 prophage; IS5 transposase and trans-	Cytoplasmic	COG3039;Transposase and inactivated derivatives, IS5 family			
insH	CDS	IS5 transposase and trans-activator	Cytoplasmic	COG3039;Transposase and inactivated derivatives, IS5 family			
insH	CDS	IS5 transposase and trans-activator	Cytoplasmic	COG3039;Transposase and inactivated derivatives, IS5 family			
insH	CDS	Rac prophage; IS5 transposase and trans-activator	Cytoplasmic	COG3039;Transposase and inactivated derivatives, IS5 family			
insH	CDS	CP4-44 prophage; IS5 transposase and trans-	Cytoplasmic	COG3039;Transposase and inactivated derivatives, IS5 family			
insH	CDS	IS5 transposase and trans-activator	Cytoplasmic	COG3039;Transposase and inactivated derivatives, IS5 family			
insH	CDS	IS5 transposase and trans-activator	Cytoplasmic	COG3039;Transposase and inactivated derivatives, IS5 family			
insH	CDS	IS5 transposase and trans-activator	Cytoplasmic	COG3039;Transposase and inactivated derivatives, IS5 family			
insH	CDS	IS5 transposase and trans-activator	Cytoplasmic	COG3039;Transposase and inactivated derivatives, IS5 family			
insH	CDS	IS5 transposase and trans-activator	Cytoplasmic	COG3039;Transposase and inactivated derivatives, IS5 family			
insI	CDS	CP4-6 prophage; IS30 transposase	Cytoplasmic	COG2826;Transposase and inactivated derivatives, IS30 family			
insI	CDS	IS30 transposase	Cytoplasmic	COG2826;Transposase and inactivated derivatives, IS30 family			
insI	CDS	KpLE2 phage-like element; IS30 transposase	Cytoplasmic	COG2826;Transposase and inactivated derivatives, IS30 family			
insJ	CDS	IS150 protein InsA. Couples formation of linear intermediate to transposition.	Cytoplasmic	COG2963;Transposase and inactivated derivatives			
insK	CDS	IS150 conserved protein InsB	Cytoplasmic	COG2801;Transposase and inactivated derivatives			
insL	CDS	IS186/IS421 transposase	Cytoplasmic	COG3385;FOG: Transposase and inactivated derivatives			
insL	CDS	IS186/IS421 transposase	Cytoplasmic	COG3385;FOG: Transposase and inactivated derivatives			
insL	CDS	predicted transposase	Cytoplasmic	COG3385;FOG: Transposase and inactivated derivatives			
insM	CDS	KpLE2 phage-like element; predicted transposase fragment (pseudogene)	Cytoplasmic				
insN	CDS	CP4-6 prophage; partial regulator of insertion element IS911A	Cytoplasmic	COG2963;Transposase and inactivated derivatives			
insN	CDS	KpLE2 phage-like element; partial regulator of insertion element IS911B. Interrupted by insertion of IS30 element.	Cytoplasmic				
insO	CDS	CP4-6 prophage; partial transposase of insertion element IS911A, interrupted by insertion of IS30 element.	Cytoplasmic	COG2801;Transposase and inactivated derivatives		o	tt
insO	CDS	KpLE2 phage-like element; partial transposase (pseudogene)	Cytoplasmic	COG2801;Transposase and inactivated derivatives			
intA	CDS	CP4-57 prophage; integrase	Cytoplasmic	COG0582;Integrase		o	tt
intB	CDS	KpLE2 phage-like element; predicted integrase	Cytoplasmic	COG0582;Integrase		o	tt
intD	CDS	DLP12 prophage; predicted integrase	Cytoplasmic	COG0582;Integrase		o	tt
intE	CDS	e14 prophage; predicted integrase	Cytoplasmic	COG0582;Integrase		o	tt
intF	CDS	CP4-6 prophage; predicted phage integrase	Cytoplasmic	COG0582;Integrase		o	tt
intG	CDS	predicted defective phage integrase (pseudogene)	Cytoplasmic	COG0582;Integrase			
intQ	CDS	Qin prophage; predicted defective integrase	Cytoplasmic	COG0582;Integrase		gg	o
intR	CDS	Rac prophage; integrase	Cytoplasmic	COG0582;Integrase		o	tt
intS	CDS	CPS-53 (KpLE1) prophage; predicted prophage CPS-53 integrase	Cytoplasmic	COG0582;Integrase		g	tt
intZ	CDS	CPZ-55 prophage; predicted integrase	Cytoplasmic	COG0582;Integrase			
iscA	CDS	FeS cluster assembly protein	Cytoplasmic	COG0316;Uncharacterized conserved protein	GO:0006457 protein folding	gg	o
iscR	CDS	DNA-binding transcriptional activator	Cytoplasmic	COG1959;Predicted transcriptional regulator	GO:0006412 protein biosynthesis	gg	o
iscS	CDS	cysteine desulfurase (tRNA sulfurtransferase), PLP-dependent	Cytoplasmic		GO:0009451 RNA modification	gg	t
iscU	CDS	scaffold protein	Cytoplasmic	COG0822;NifU homolog involved in Fe-S cluster formation	GO:0006412 protein biosynthesis	gg	t
ispA	CDS	geranyltranstransferase	Cytoplasmic	COG0142;Geranylgeranyl pyrophosphate synthase		gg	t
ispB	CDS	octaprenyl diphosphate synthase	Cytoplasmic	COG0142;Geranylgeranyl pyrophosphate synthase		gg	tt
ispD	CDS	4-diphosphocytidyl-2C-methyl-D-erythritol synthase	Cytoplasmic	COG1211;4-diphosphocytidyl-2-methyl-D-erithritol synthase		gg	t
ispE	CDS	4-diphosphocytidyl-2-C-methylerythritol kinase	Cytoplasmic	COG1947;4-diphosphocytidyl-2C-methyl-D-erythritol 2-phosphate synthase		gg	o
ispF	CDS	2C-methyl-D-erythritol 2,4-cyclodiphosphate synthase	Cytoplasmic	COG0245;2C-methyl-D-erythritol 2,4-cyclodiphosphate synthase		gg	tt
ispG	CDS	1-hydroxy-2-methyl-2-(E)-butenyl 4-diphosphate synthase	Cytoplasmic	COG0821;Enzyme involved in the deoxyxylulose pathway of isoprenoid biosynthesis		gg	t
ispH	CDS	1-hydroxy-2-methyl-2-(E)-butenyl 4-diphosphate reductase, 4Fe-4S protein	Cytoplasmic	COG0761;Penicillin tolerance protein	GO:0042493 response to drug	g	tt
ispU	CDS	undecaprenyl pyrophosphate synthase	Cytoplasmic	COG0020;Undecaprenyl pyrophosphate synthase		gg	tt
isrA	misc_RNA	small RNA					
isrB	misc_RNA	small RNA					
isrC	misc_RNA	small RNA					
ivbL	CDS	ilvB operon leader peptide	Cytoplasmic			gg	o
ivy	CDS	inhibitor of vertebrate C-lysozyme	Periplasmic			gg	tt
katE	CDS	hydroperoxidase HPII(III) (catalase)	Cytoplasmic		GO:0006805 xenobiotic metabolism	gg	o
katG	CDS	catalase/hydroperoxidase HPI(I)	Cytoplasmic	COG0376;Catalase (peroxidase I)	GO:0006805 xenobiotic metabolism	g	tt
kbaY	CDS	tagatose 6-phosphate aldolase 1, kbaY subunit	Cytoplasmic	COG0191;Fructose/tagatose bisphosphate aldolase	GO:0016052 carbohydrate	gg	t
kbaZ	CDS	tagatose 6-phosphate aldolase 1, kbaZ subunit	Cytoplasmic	COG4573;Predicted tagatose 6-phosphate kinase	GO:0016052 carbohydrate	gg	t
kbl	CDS	glycine C-acetyltransferase	Cytoplasmic	COG0156;7-keto-8-aminopelargonate synthetase and related enzymes	GO:0009063 amino acid catabolism	gg	t
kch	CDS	voltage-gated potassium channel	Integral Membrane Protein	COG1226;Kef-type K+ transport systems, predicted NAD-binding		gg	tt
kdgK	CDS	ketodeoxygluconokinase	Cytoplasmic	COG0524;Sugar kinases, ribokinase family	GO:0016052 carbohydrate		
kdgR	CDS	predicted DNA-binding transcriptional regulator	Cytoplasmic	COG1414;Transcriptional regulator	GO:0006350 transcription	g	tt
kdgT	CDS	2-keto-3-deoxy-D-gluconate transporter	Integral Membrane Protein		GO:0016052 carbohydrate		
kdpA	CDS	potassium translocating ATPase, subunit A	Integral Membrane Protein	COG2060;K+-transporting ATPase, A chain		o	tt
kdpB	CDS	potassium translocating ATPase, subunit B	Integral Membrane Protein	COG2216;High-affinity K+ transport system, ATPase chain B		o	tt
kdpC	CDS	potassium translocating ATPase, subunit C	Membrane Anchored	COG2156;K+-transporting ATPase, c chain		o	tt
kdpD	CDS	fused sensory histidine kinase in two-component regulatory system with KdpE	Integral Membrane Protein	COG2205;Osmosensitive K+ channel histidine kinase	GO:0006464 protein modification	o	tt
kdpE	CDS	DNA-binding response regulator in two-component regulatory system with KdpD	Cytoplasmic	COG0745;Response regulators consisting of a CheY-like receiver domain and a winged-helix DNA-binding domain	GO:0006350 transcription		
kdpF	CDS	potassium ion accessory transporter subunit	Membrane Anchored			o	tt
kdsA	CDS	3-deoxy-D-manno-octulosonate 8-phosphate synthase	Cytoplasmic	COG2877;3-deoxy-D-manno-octulosonic acid (KDO) 8-phosphate synthase	GO:0009244 lipopolysaccharide core region biosynthesis -!- GO:0009248 K antigen biosynthesis	gg	t
kdsB	CDS	3-deoxy-manno-octulosonate cytidylyltransferase	Cytoplasmic	COG1212;CMP-2-keto-3-deoxyoctulosonic acid synthetase	GO:0009244 lipopolysaccharide core region biosynthesis -!- GO:0009245 lipid A biosynthesis -!- GO:0009248 K antigen biosynthesis	gg	t
kdsC	CDS	3-deoxy-D-manno-octulosonate 8-phosphate phosphatase	Periplasmic	COG1778;Low specificity phosphatase (HAD superfamily)		o	tt
kdsD	CDS	D-arabinose 5-phosphate isomerase	Cytoplasmic	COG0794;Predicted sugar phosphate isomerase involved in capsule formation -!- COG0517;FOG: CBS domain		gg	o
kdtA	CDS	3-deoxy-D-manno-octulosonic-acid transferase (KDO transferase)	Cytoplasmic	COG1519;3-deoxy-D-manno-octulosonic-acid transferase	GO:0009245 lipid A biosynthesis -!- GO:0009244 lipopolysaccharide core region biosynthesis -!- GO:0009248 K antigen biosynthesis	o	tt
kduD	CDS	2-deoxy-D-gluconate 3-dehydrogenase	Cytoplasmic	COG1028;Dehydrogenases with different specificities (related to short-chain alcohol dehydrogenases)		gg	t
kduI	CDS	predicted 5-keto 4-deoxyuronate isomerase	Cytoplasmic	COG3717;5-keto 4-deoxyuronate isomerase	GO:0016052 carbohydrate	gg	t
kefA	CDS	fused mechanosensitive channel proteins	Integral Membrane Protein	COG3264;Small-conductance mechanosensitive channel		o	tt

kefB	CDS	potassium:proton antiporter	Integral Membrane Protein	COG0475;Kef-type K+ transport systems, membrane components -!- COG1226;Kef-type K+ transport systems, predicted NAD-binding		o	tt
kefC	CDS	potassium:proton antiporter	Integral Membrane Protein	COG0475;Kef-type K+ transport systems, membrane components -!- COG1226;Kef-type K+ transport systems, predicted NAD-binding		o	tt
kefF	CDS	flavoprotein subunit for the KefC potassium efflux system	Cytoplasmic	COG2249;Putative NADPH-quinone reductase (modulator of drug activity B)		gg	o
kefG	CDS	component of potassium efflux complex with KefB	Cytoplasmic	COG2249;Putative NADPH-quinone reductase (modulator of drug activity		gg	o
kgtP	CDS	alpha-ketoglutarate transporter	Integral Membrane Protein	COG0477;Permeases of the major facilitator superfamily	GO:0016052 carbohydrate	o	tt
kil	CDS	Rac prophage; inhibitor of ftsZ, killing protein	Cytoplasmic			gg	tt
kptA	CDS	2'-phosphotransferase	Cytoplasmic				
ksgA	CDS	S-adenosylmethionine-6-N',N'-adenosyl (rRNA) dimethyltransferase	Cytoplasmic	COG0030;Dimethyladenosine transferase (rRNA methylation)	GO:0009451 RNA modification -!- GO:0042493 response to drug	gg	t
lacA	CDS	thiogalactoside acetyltransferase	Cytoplasmic	COG0110;Acetyltransferase (isoleucine patch superfamily)	GO:0016052 carbohydrate	gg	o
lacI	CDS	DNA-binding transcriptional repressor	Cytoplasmic		GO:0016052 carbohydrate catabolism -!- GO:0006350	o	tt
lacY	CDS	lactose/galactose transporter	Integral Membrane Protein	COG0477;Permeases of the major facilitator superfamily	GO:0016052 carbohydrate	o	tt
lacZ	CDS	beta-D-galactosidase	Cytoplasmic	COG3250;Beta-galactosidase/beta-glucuronidase	GO:0016052 carbohydrate	gg	t
lamB	CDS	maltose outer membrane porin (maltoporin)	Outer Membrane B-barrel protein	COG4580;Maltoporin (phage lambda and maltose receptor)		o	tt
lar	CDS	Rac prophage; restriction alleviation protein	Cytoplasmic				
ldcA	CDS	L,D-carboxypeptidase A	Cytoplasmic	COG1619;Uncharacterized proteins, homologs of microcin C7 resistance protein MccF	GO:0000270 peptidoglycan metabolism	gg	t
ldcC	CDS	lysine decarboxylase 2, constitutive	Cytoplasmic	COG1982;Arginine/lysine/ornithine decarboxylases	GO:0006096 glycolysis -!- GO:0016052 carbohydrate	o	tt
ldhA	CDS	fermentative D-lactate dehydrogenase, NAD-dependent	Cytoplasmic	COG1052;Lactate dehydrogenase and related dehydrogenases	GO:0006113 fermentation	gg	t
ldrA	CDS	toxic polypeptide, small	Cytoplasmic				
ldrB	CDS	toxic polypeptide, small	Cytoplasmic				
ldrC	CDS	toxic polypeptide, small	Cytoplasmic				
ldrD	CDS	toxic polypeptide, small	Cytoplasmic				
lepA	CDS	GTP binding membrane protein	Cytoplasmic	COG0481;Membrane GTPase LepA	GO:0006412 protein biosynthesis	o	tt
lepB	CDS	leader peptidase (signal peptidase I)	Integral Membrane Protein	COG0681;Signal peptidase I		o	tt
leuA	CDS	2-isopropylmalate synthase	Cytoplasmic	COG0119;Isopropylmalate/homocitrate/citramalate synthases	GO:0009098 leucine biosynthesis	gg	t
leuB	CDS	3-isopropylmalate dehydrogenase	Cytoplasmic		GO:0009098 leucine biosynthesis		
leuC	CDS	3-isopropylmalate isomerase subunit, dehydratase component	Cytoplasmic	COG0065;3-isopropylmalate dehydratase large subunit	GO:0009098 leucine biosynthesis	gg	t
leuD	CDS	3-isopropylmalate isomerase subunit	Cytoplasmic	COG0066;3-isopropylmalate dehydratase small subunit	GO:0009098 leucine biosynthesis	gg	o
leuL	CDS	leu operon leader peptide	Cytoplasmic		GO:0009098 leucine biosynthesis	gg	o
leuO	CDS	DNA-binding transcriptional activator	Cytoplasmic		GO:0009098 leucine biosynthesis -!- GO:0006350 transcription	gg	t
leuP	tRNA	tRNA-Leu(CAG) (Leucine tRNA1)					
leuQ	tRNA	tRNA-Leu(CAG) (Leucine tRNA1)					
leuS	CDS	leucyl-tRNA synthetase	Cytoplasmic	COG0495;Leucyl-tRNA synthetase	GO:0006418 amino acid activation	o	tt
leuT	tRNA	tRNA-Leu(CAG) (Leucine tRNA10					
leuU	tRNA	tRNA-Leu(GAG) (Leucine tRNA2)					
leuV	tRNA	tRNA-Leu(CAG) (Leucine tRNA1)					
leuW	tRNA	tRNA-Leu(UAG) (Leucine tRNA3)					
leuX	tRNA	tRNA-Leu(CAA) (Leucine tRNA5)					
leuZ	tRNA	tRNA-Leu(UAA) (Leucine tRNA4)					
lexA	CDS	DNA-binding transcriptional repressor	Cytoplasmic	COG1974;SOS-response transcriptional repressors (RecA-mediated autopeptidases)	GO:0006281 DNA repair -!- GO:0006350 transcription -!- GO:0009432 SOS response	o	tt
lgt	CDS	phosphatidylglycerol-prolipoprotein diacylglyceryl transferase	Integral Membrane Protein	COG0682;Prolipoprotein diacylglyceryltransferase	GO:0008654 phospholipid biosynthesis -!- GO:0006464 protein modification	o	tt
lhr	CDS	predicted ATP-dependent helicase	Cytoplasmic	COG1201;Lhr-like helicases	GO:0006261 DNA dependent DNA replication	o	t
ligA	CDS	DNA ligase, NAD(+)-dependent	Cytoplasmic	COG0272;NAD-dependent DNA ligase (contains BRCT domain type II)	GO:0006281 DNA repair	gg	tt
ligB	CDS	DNA ligase, NAD(+)-dependent	Cytoplasmic	COG0272;NAD-dependent DNA ligase (contains BRCT domain type II)	GO:0006259 DNA metabolism	o	tt
ligT	CDS	2'-5' RNA ligase	Cytoplasmic		GO:0009451 RNA modification		
lipA	CDS	lipoate synthase	Cytoplasmic	COG0320;Lipoate synthase	GO:0009107 lipoate biosynthesis	gg	t
lipB	CDS	lipoyl-protein ligase	Cytoplasmic	COG0321;Lipoate-protein ligase B	GO:0009107 lipoate biosynthesis		
lit	CDS	e14 prophage; cell death peptidase, inhibitor of T4 late gene expression	Cytoplasmic			o	o
livF	CDS	leucine/isoleucine/valine transporter subunit -!- ATP-binding component of ABC superfamily	Cytoplasmic		GO:0009063 amino acid catabolism -!- GO:0009098 leucine biosynthesis	gg	t
livG	CDS	leucine/isoleucine/valine transporter subunit -!- ATP-binding component of ABC superfamily	Cytoplasmic	COG0411;ABC-type branched-chain amino acid transport systems, ATPase component	GO:0009063 amino acid catabolism -!- GO:0009098 leucine biosynthesis	g	tt
livH	CDS	leucine/isoleucine/valine transporter subunit -!- membrane component of ABC superfamily	Integral Membrane Protein	COG0559;Branched-chain amino acid ABC-type transport system, permease components	GO:0009098 leucine biosynthesis	o	tt
livJ	CDS	leucine/isoleucine/valine transporter subunit -!- periplasmic-binding component of ABC superfamily	Periplasmic		GO:0009098 leucine biosynthesis	o	tt
livK	CDS	leucine transporter subunit -!- periplasmic-binding component of ABC superfamily	Periplasmic	COG0683;ABC-type branched-chain amino acid transport systems, periplasmic component	GO:0009098 leucine biosynthesis	o	tt
livM	CDS	leucine/isoleucine/valine transporter subunit -!- membrane component of ABC superfamily	Integral Membrane Protein	COG4177;ABC-type branched-chain amino acid transport system, permease component	GO:0009098 leucine biosynthesis	o	tt
lldD	CDS	L-lactate dehydrogenase, FMN-linked	Cytoplasmic	COG1304;L-lactate dehydrogenase (FMN-dependent) and related alpha-hydroxy acid dehydrogenases	GO:0009060 aerobic respiration -!- GO:0009061 anaerobic respiration	gg	t
lldP	CDS	L-lactate permease	Integral Membrane Protein	COG1620;L-lactate permease	GO:0016052 carbohydrate	o	tt
lldR	CDS	DNA-binding transcriptional repressor	Cytoplasmic	COG2186;Transcriptional regulators	GO:0009060 aerobic respiration -!- GO:0006350 transcription	o	tt
lnt	CDS	apolipoprotein N-acyltransferase	Integral Membrane Protein	COG0815;Apolipoprotein N-acyltransferase	GO:0042158 lipoprotein biosynthesis	o	tt
lolA	CDS	chaperone for lipoproteins	Periplasmic	COG2834;Outer membrane lipoprotein-sorting protein		o	tt
lolB	CDS	chaperone for lipoproteins	Outer Membrane Lipoprotein	COG3017;Outer membrane lipoprotein involved in outer membrane biogenesis		gg	tt
lolC	CDS	outer membrane-specific lipoprotein transporter subunit -!- membrane component of ABC superfamily	Integral Membrane Protein	COG4591;ABC-type transport system, involved in lipoprotein release, permease component			
lolD	CDS	outer membrane-specific lipoprotein transporter subunit -!- ATP-binding component of ABC superfamily	Cytoplasmic				
lolE	CDS	outer membrane-specific lipoprotein transporter subunit -!- membrane component of ABC superfamily	Integral Membrane Protein	COG4591;ABC-type transport system, involved in lipoprotein release, permease component		o	tt
lomR	CDS	Rac prophage; predicted protein, N-ter fragment (pseudogene)	Cytoplasmic	COG3637;Opacity protein and related surface antigens			
lomR	ancestral	Rac prophage; predicted protein (pseudogene)	Cytoplasmic	COG3637;Opacity protein and related surface antigens			
lomR	CDS	Rac prophage; predicted protein, C-ter fragment (pseudogene)	Cytoplasmic	COG3637;Opacity protein and related surface antigens			
lon	CDS	DNA-binding ATP-dependent protease La	Cytoplasmic	COG0466;ATP-dependent Lon protease, bacterial type		o	tt
lpcA	CDS	D-sedoheptulose 7-phosphate isomerase	Cytoplasmic	COG0279;Phosphoheptose isomerase	GO:0009244 lipopolysaccharide core region biosynthesis	g	t
lpd	CDS	lipoamide dehydrogenase, E3 component is part of three enzyme complexes	Cytoplasmic	COG1249;Pyruvate/2-oxoglutarate dehydrogenase complex, dihydrolipoamide dehydrogenase (E3) component, and related enzymes	GO:0016052 carbohydrate catabolism -!- GO:0009063 amino acid catabolism -!- GO:0006096 glycolysis -!- GO:0006086 acetyl-CoA biosynthesis from pyruvate -!- GO:0006099 tricarboxylic acid cycle -!- GO:0009060 aerobic respiration -!- GO:0009061 anaerobic res	gg	o
lplA	CDS	lipoate-protein ligase A	Cytoplasmic	COG0095;Lipoate-protein ligase A	GO:0042158 lipoprotein biosynthesis -!- GO:0006418 amino acid activation	gg	t
lpp	CDS	murein lipoprotein	Outer Membrane Lipoprotein	COG4238;Murein lipoprotein	GO:0009252 peptidoglycan biosynthesis	o	tt
lpxA	CDS	UDP-N-acetylglucosamine acetyltransferase	Cytoplasmic	COG1043;Acyl-[acyl carrier protein]--UDP-N-acetylglucosamine O-acyltransferase	GO:0009245 lipid A biosynthesis	gg	o
lpxB	CDS	tetraacyldisaccharide-1-P synthase	Cytoplasmic	COG0763;Lipid A disaccharide synthetase	GO:0009245 lipid A biosynthesis	o	tt
lpxC	CDS	UDP-3-O-acyl N-acetylglucosamine deacetylase	Cytoplasmic	COG0774;UDP-3-O-acyl-N-acetylglucosamine deacetylase	GO:0009245 lipid A biosynthesis	o	tt
lpxD	CDS	UDP-3-O-(3-hydroxymyristoyl)-glucosamine N-acyltransferase	Cytoplasmic	COG1044;UDP-3-O-[3-hydroxymyristoyl] glucosamine N-acyltransferase	GO:0009245 lipid A biosynthesis	gg	tt
lpxH	CDS	UDP-2,3-diacylglucosamine pyrophosphatase	Cytoplasmic	COG2908;Uncharacterized protein conserved in bacteria	GO:0009245 lipid A biosynthesis	o	tt
lpxK	CDS	lipid A 4'kinase	Integral Membrane Protein	COG1663;Tetraacyldisaccharide-1-P 4'-kinase	GO:0009244 lipopolysaccharide core region biosynthesis -!- GO:0009245 lipid A biosynthesis -!- GO:0009248 K antigen biosynthesis	o	t
lpxL	CDS	lauryl-acyl carrier protein (ACP)-dependent acyltransferase	Membrane Anchored	COG1560;Lauroyl/myristoyl acyltransferase	GO:0009245 lipid A biosynthesis	o	tt
lpxM	CDS	myristoyl-acyl carrier protein (ACP)-dependent acyltransferase	Membrane Anchored	COG1560;Lauroyl/myristoyl acyltransferase	GO:0009245 lipid A biosynthesis	gg	tt
lrhA	CDS	DNA-binding transcriptional repressor	Cytoplasmic	COG0583;Transcriptional regulator	GO:0009060 aerobic respiration -!- GO:0006350 transcription	gg	tt
lrp	CDS	DNA-binding transcriptional dual regulator, leucine-binding	Cytoplasmic	COG1522;Transcriptional regulators	GO:0009098 leucine biosynthesis -!- GO:0006350 transcription	gg	tt
lspA	CDS	prolipoprotein signal peptidase (signal peptidase II)	Integral Membrane Protein	COG0597;Lipoprotein signal peptidase		o	tt
lsrB	CDS	AI2 transporter -!- periplasmic-binding component of ABC superfamily	Periplasmic	COG1879;ABC-type sugar transport system, periplasmic component		o	tt
lsrC	CDS	AI2 transporter -!- membrane component of ABC superfamily	Integral Membrane Protein	COG1172;Ribose/xylose/arabinose/galactoside ABC-type transport systems, permease components		o	tt
lsrD	CDS	AI2 transporter -!- membrane component of ABC superfamily	Integral Membrane Protein	COG1172;Ribose/xylose/arabinose/galactoside ABC-type transport systems, permease components		o	tt
lsrF	CDS	predicted aldolase	Cytoplasmic	COG1830;DhnA-type fructose-1,6-bisphosphate aldolase and related enzymes		gg	t
lsrG	CDS	conserved protein	Cytoplasmic	COG1359;Uncharacterized conserved protein		gg	t

ltaE	CDS	L-allo-threonine aldolase, PLP-dependent	Cytoplasmic	COG2008;Threonine aldolase	GO:0006545 glycine biosynthesis	gg	t
luxS	CDS	S-ribosylhomocysteinase	Cytoplasmic	COG1854;LuxS protein involved in autoinducer AI2 synthesis		gg	o
lysA	CDS	diaminopimelate decarboxylase, PLP-binding	Cytoplasmic	COG0019;Diaminopimelate decarboxylase	GO:0009089 lysine biosynthesis via diaminopimelate	o	tt
lysC	CDS	aspartokinase III	Cytoplasmic	COG0527;Aspartokinases	GO:0009089 lysine biosynthesis via diaminopimelate -!- GO:0009090 homoserine biosynthesis	g	o
lysP	CDS	lysine transporter	Integral Membrane Protein	COG0833;Amino acid transporters	GO:0009089 lysine biosynthesis via diaminopimelate	o	tt
lysQ	tRNA	tRNA-Lys(UUU) (Lysine tRNA)					
lysR	CDS	DNA-binding transcriptional dual regulator	Cytoplasmic	COG0583;Transcriptional regulator	GO:0009089 lysine biosynthesis via diaminopimelate -!- GO:0006350 transcription	gg	tt
lysS	CDS	lysine tRNA synthetase, constitutive	Cytoplasmic	COG1190;Lysyl-tRNA synthetase (class II)	GO:0006418 amino acid activation	gg	t
lysT	tRNA	tRNA-Lys(UUU) (Lysine tRNA)					
lysU	CDS	lysine tRNA synthetase, inducible	Cytoplasmic	COG1190;Lysyl-tRNA synthetase (class II)	GO:0006418 amino acid activation	gg	t
lysV	tRNA	tRNA-Lys(UUU) (Lysine tRNA)					
lysW	tRNA	tRNA-Lys(UUU) (Lysine tRNA)					
lysY	tRNA	tRNA-Lys(UUU) (Lysine tRNA)					
lysZ	tRNA	tRNA-Lys(UUU), (Lysine tRNA)					
lyxK	CDS	L-xylulose kinase	Cytoplasmic	COG1070;Sugar (pentulose and hexulose) kinases	GO:0016052 carbohydrate	o	tt
maa	CDS	maltose O-acetyltransferase	Cytoplasmic	COG0110;Acetyltransferase (isoleucine patch superfamily)	GO:0016052 carbohydrate	gg	o
macA	CDS	macrolide transporter subunit, membrane fusion protein (MFP) component	Membrane Anchored			o	tt
macB	CDS	fused macrolide transporter subunits -!- ATP-binding component and membrane component of ABC superfamily	Integral Membrane Protein	COG1136;ABC-type antimicrobial peptide transport system, ATPase component -!- COG0577;ABC-type antimicrobial peptide transport system, permease component		o	tt
maeB	CDS	fused malic enzyme predicted oxidoreductase -!- predicted phosphotransacetylase	Cytoplasmic	COG0281;Malic enzyme -!- COG0280;Phosphotransacetylase		g	t
mak	CDS	manno(fructo)kinase	Cytoplasmic	COG1940;Transcriptional regulator/sugar kinase	GO:0016052 carbohydrate	gg	t
malE	CDS	maltose transporter subunit -!- periplasmic-binding component of ABC superfamily	Periplasmic	COG2182;Maltose-binding periplasmic proteins/domains	GO:0016052 carbohydrate catabolism -!- GO:0006457 protein	o	tt
malF	CDS	maltose transporter subunit -!- membrane component of ABC superfamily	Integral Membrane Protein	COG1175;ABC-type sugar transport systems, permease components	GO:0016052 carbohydrate catabolism	o	tt
malG	CDS	maltose transporter subunit -!- membrane component of ABC superfamily	Integral Membrane Protein	COG3833;ABC-type maltose transport systems, permease component	GO:0016052 carbohydrate catabolism	o	tt
malI	CDS	DNA-binding transcriptional repressor	Cytoplasmic	COG1609;Transcriptional regulators	GO:0016052 carbohydrate catabolism -!- GO:0006350	o	tt
malK	CDS	fused maltose transport subunit, ATP-binding component of ABC superfamily -!- regulatory protein	Cytoplasmic	COG3839;ABC-type sugar transport systems, ATPase components	GO:0016052 carbohydrate catabolism	gg	t
malM	CDS	maltose regulon periplasmic protein	Periplasmic		GO:0016052 carbohydrate	o	tt
malP	CDS	maltodextrin phosphorylase	Cytoplasmic		GO:0016052 carbohydrate catabolism -!- GO:0000272 polysaccharide catabolism		
malQ	CDS	4-alpha-glucanotransferase (amylomaltase)	Cytoplasmic	COG1640;4-alpha-glucanotransferase	GO:0016052 carbohydrate catabolism -!- GO:0000272 polysaccharide catabolism	o	tt
malS	CDS	alpha-amylase	Periplasmic	COG0366;Glycosidases	GO:0016052 carbohydrate catabolism -!- GO:0000272 polysaccharide catabolism -!- GO:0006508 proteolysis and	o	tt
malT	CDS	DNA-binding transcriptional activator, maltotriose-ATP-binding	Cytoplasmic	COG2909;ATP-dependent transcriptional regulator	GO:0016052 carbohydrate catabolism -!- GO:0006350	o	tt
malX	CDS	fused maltose and glucose-specific PTS enzyme IIBC components	Integral Membrane Protein	COG1263;Phosphotransferase system IIC components, glucose/maltose/N-acetylglucosamine-specific -!- COG1264;Phosphotransferase system IIB components	GO:0016052 carbohydrate catabolism	o	tt
malY	CDS	bifunctional beta-cystathionase, PLP-dependent -!- regulator of maltose regulon	Cytoplasmic	COG1168;Bifunctional PLP-dependent enzyme with beta-cystathionase and maltose regulon repressor activities	GO:0016052 carbohydrate catabolism	gg	o
malZ	CDS	maltodextrin glucosidase	Cytoplasmic	COG0366;Glycosidases	GO:0016052 carbohydrate catabolism -!- GO:0000272 polysaccharide catabolism	o	tt
manA	CDS	mannose-6-phosphate isomerase	Cytoplasmic		GO:0016052 carbohydrate catabolism -!- GO:0009226 nucleotide-sugar biosynthesis -!- GO:0009242 colanic acid biosynthesis -!- GO:0009269	gg	o
manX	CDS	fused mannose-specific PTS enzyme IIAB components	Cytoplasmic	COG2893;Phosphotransferase system, mannose/fructose-specific component IIA -!- COG3444;Phosphotransferase system, mannose/fructose/N-acetylgalactosamine-specific component IIB	GO:0016052 carbohydrate catabolism	gg	tt
manY	CDS	mannose-specific enzyme IIC component of PTS	Integral Membrane Protein	COG3715;Phosphotransferase system, mannose/fructose/N-acetylgalactosamine-specific component IIC	GO:0016052 carbohydrate catabolism	o	tt
manZ	CDS	mannose-specific enzyme IID component of PTS	Integral Membrane Protein		GO:0016052 carbohydrate	o	tt
maoC	CDS	fused aldehyde dehydrogenase -!- enoyl-CoA hydratase	Cytoplasmic	COG1012;NAD-dependent aldehyde dehydrogenases -!- COG2030;Acyl dehydratase	GO:0016052 carbohydrate catabolism	g	tt
map	CDS	methionine aminopeptidase	Cytoplasmic	COG0024;Methionine aminopeptidase	GO:0006457 protein folding	gg	o
marA	CDS	DNA-binding transcriptional dual regulator	Cytoplasmic	COG2207;AraC-type DNA-binding domain-containing proteins	GO:0006350 transcription -!- GO:0042493 response to drug -!- GO:0006805 xenobiotic metabolism		
marB	CDS	predicted protein	Periplasmic		GO:0042493 response to drug -!- GO:0006805 xenobiotic metabolism	g	tt
marC	CDS	predicted transporter	Integral Membrane Protein	COG2095;Multiple antibiotic transporter		g	tt
marR	CDS	DNA-binding transcriptional repressor	Cytoplasmic		GO:0006350 transcription -!- GO:0042493 response to drug		
mazG	CDS	nucleoside triphosphate pyrophosphohydrolase	Cytoplasmic	COG1694;Predicted pyrophosphatase		gg	tt
mbhA	CDS	flagellar system protein, promoterless fragment (pseudogene)	Cytoplasmic	COG1360;Flagellar motor protein	GO:0042330 taxis		
mcrA	CDS	e14 prophage; 5-methylcytosine-specific restriction endonuclease B	Cytoplasmic	COG1403;Restriction endonuclease		gg	t
mcrB	CDS	5-methylcytosine-specific restriction enzyme McrBC, subunit McrB	Cytoplasmic		GO:0006308 DNA catabolism -!- GO:0006308 DNA catabolism		
mcrC	CDS	5-methylcytosine-specific restriction enzyme McrBC, subunit McrC	Cytoplasmic	COG4268;McrBC 5-methylcytosine restriction system component	GO:0006308 DNA catabolism -!- GO:0006308 DNA catabolism		
mdaB	CDS	NADPH quinone reductase	Cytoplasmic	COG2249;Putative NADPH-quinone reductase (modulator of drug activity	GO:0042493 response to drug	gg	tt
mdh	CDS	malate dehydrogenase, NAD(P)-binding	Cytoplasmic	COG0039;Malate/lactate dehydrogenases	GO:0016052 carbohydrate catabolism -!- GO:0006099 tricarboxylic acid cycle -!- GO:0006113 fermentation -!- GO:0009061 anaerobic respiration -!- GO:0006094 gluconeogenesis -!-	gg	t
mdlA	CDS	fused predicted multidrug transporter subunits -!- ATP-binding components of ABC superfamily	Integral Membrane Protein	COG1132;ABC-type multidrug transport system, ATPase and permease components	GO:0042493 response to drug		
mdlB	CDS	fused predicted multidrug transporter subunits -!- ATP binding components of ABC superfamily	Integral Membrane Protein	COG1132;ABC-type multidrug transport system, ATPase and permease components			
mdoB	CDS	phosphoglycerol transferase I	Integral Membrane Protein	COG1368;Phosphoglycerol transferase and related proteins, alkaline phosphatase superfamily	GO:0008654 phospholipid biosynthesis -!- GO:0009250 glucan biosynthesis -!- GO:0006970 response to osmotic stress		
mdoC	CDS	membrane protein required for modification of periplasmic glucan	Integral Membrane Protein		GO:0009250 glucan biosynthesis -!- GO:0006970 response to osmotic stress	o	tt
mdoD	CDS	glucan biosynthesis protein, periplasmic	Periplasmic		GO:0006970 response to osmotic stress	gg	t
mdoG	CDS	glucan biosynthesis protein, periplasmic	Periplasmic	COG3131;Periplasmic glucans biosynthesis protein	GO:0009250 glucan biosynthesis -!- GO:0006970 response to osmotic stress	o	tt
mdoH	CDS	glucan biosynthesis: glycosyl transferase	Integral Membrane Protein	COG2943;Membrane glycosyltransferase	GO:0006970 response to osmotic stress	o	tt
mdtA	CDS	multidrug efflux system, subunit A	Membrane Anchored	COG0845;Membrane-fusion protein			
mdtB	CDS	multidrug efflux system, subunit B	Integral Membrane Protein	COG0841;Cation/multidrug efflux pump		o	tt
mdtC	CDS	multidrug efflux system, subunit C	Integral Membrane Protein	COG0841;Cation/multidrug efflux pump		o	tt
mdtD	CDS	multidrug efflux system protein	Integral Membrane Protein	COG0477;Permeases of the major facilitator superfamily		o	tt
mdtE	CDS	multidrug resistance efflux transporter	Inner Membrane Lipoprotein	COG0845;Membrane-fusion protein		o	tt
mdtF	CDS	multidrug transporter, RpoS-dependent	Integral Membrane Protein	COG0841;Cation/multidrug efflux pump		o	tt
mdtG	CDS	predicted drug efflux system	Integral Membrane Protein	COG0477;Permeases of the major facilitator superfamily		o	tt
mdtH	CDS	predicted drug efflux system	Integral Membrane Protein			o	tt
mdtI	CDS	multidrug efflux system transporter	Integral Membrane Protein	COG2076;Membrane transporters of cations and cationic drugs			
mdtJ	CDS	multidrug efflux system transporter	Integral Membrane Protein	COG2076;Membrane transporters of cations and cationic drugs		o	tt
mdtK	CDS	multidrug efflux system transporter	Integral Membrane Protein		GO:0042493 response to drug	gg	tt
mdtL	CDS	multidrug efflux system protein	Integral Membrane Protein	COG0477;Permeases of the major facilitator superfamily		o	tt
melA	CDS	alpha-galactosidase, NAD(P)-binding	Cytoplasmic	COG1486;Alpha-galactosidases/6-phospho-beta-glucos idases, family 4 of glycosyl hydrolases	GO:0016052 carbohydrate catabolism -!- GO:0009435 nicotinamide adenine dinucleotide	gg	t
melB	CDS	melibiose:sodium symporter	Integral Membrane Protein	COG2211;Na+/melibiose symporter and related transporters	GO:0016052 carbohydrate	o	tt
melR	CDS	DNA-binding transcriptional dual regulator	Cytoplasmic	COG2207;AraC-type DNA-binding domain-containing proteins	GO:0016052 carbohydrate catabolism -!- GO:0006350	o	tt
menA	CDS	1,4-dihydroxy-2-naphthoate octaprenyltransferase	Integral Membrane Protein	COG1575;1,4-dihydroxy-2-naphthoate octaprenyltransferase	GO:0009061 anaerobic respiration	o	tt
menB	CDS	dihydroxynaphthoic acid synthetase	Cytoplasmic	COG0447;Dihydroxynaphthoic acid synthase	GO:0009061 anaerobic respiration	gg	o
menC	CDS	o-succinylbenzoyl-CoA synthase	Cytoplasmic	COG1441;O-succinylbenzoate synthase	GO:0009061 anaerobic respiration	gg	tt
menD	CDS	bifunctional 2-oxoglutarate decarboxylase -!- SHCHC synthase	Cytoplasmic	COG1165;2-succinyl-6-hydroxy-2,4-cyclohexadiene-1- carboxylate synthase	GO:0009061 anaerobic respiration		

menE	CDS	o-succinylbenzoate-CoA ligase	Cytoplasmic	COG0318;Acyl-CoA synthetases (AMP-forming)/AMP-acid ligases II	GO:0009061 anaerobic respiration	gg	t
menF	CDS	isochorismate synthase 2	Cytoplasmic		GO:0009061 anaerobic respiration	gg	t
mepA	CDS	murein DD-endopeptidase	Periplasmic	COG3770;Murein endopeptidase	GO:0009252 peptidoglycan biosynthesis -!- GO:0042493 response to drug	o	tt
metA	CDS	homoserine transsuccinylase	Cytoplasmic	COG1897;Homoserine trans-succinylase	GO:0009086 methionine biosynthesis	gg	t
metB	CDS	cystathionine gamma-synthase, PLP-dependent	Cytoplasmic	COG0626;Cystathionine beta-lyases/cystathionine gamma-synthases	GO:0009063 amino acid catabolism -!- GO:0009086 methionine biosynthesis	gg	t
metC	CDS	cystathionine beta-lyase, PLP-dependent	Cytoplasmic	COG0626;Cystathionine beta-lyases/cystathionine gamma-synthases	GO:0009086 methionine biosynthesis	g	t
metE	CDS	5-methyltetrahydropteroyltriglutamate-homocysteine S-methyltransferase	Cytoplasmic	COG0620;Methionine synthase II (cobalamin-independent)	GO:0009086 methionine biosynthesis -!- GO:0006556 S-adenosylmethionine biosynthesis	o	tt
metF	CDS	5,10-methylenetetrahydrofolate reductase	Cytoplasmic	COG0685;5,10-methylenetetrahydrofolate reductase	GO:0009257 10-formyltetrahydrofolate biosynthesis	gg	t
metG	CDS	methionyl-tRNA synthetase	Cytoplasmic	COG0143;Methionyl-tRNA synthetase -!- COG0073;EMAP domain	GO:0006418 amino acid activation	g	t
metH	CDS	homocysteine-N5-methyltetrahydrofolate transmethylase, B12-dependent	Cytoplasmic	COG0646;Methionine synthase I (cobalamin-dependent), methyltransferase domain -!- COG1410;Methionine synthase I, cobalamin-binding domain	GO:0009086 methionine biosynthesis -!- GO:0006556 S-adenosylmethionine biosynthesis -!- GO:0009257 10-formyltetrahydrofolate biosynthesis -!- GO:0009236 vitamin B12	o	tt
metI	CDS	DL-methionine transporter subunit -!- membrane component protein of ABC superfamily	Integral Membrane Protein	COG2011;ABC-type metal ion transport system, permease component		o	o
metJ	CDS	DNA-binding transcriptional repressor, S-adenosylmethionine-binding	Cytoplasmic	COG3060;Transcriptional regulator of met regulon	GO:0009086 methionine biosynthesis -!- GO:0006350 transcription	o	tt
metK	CDS	methionine adenosyltransferase 1	Cytoplasmic	COG0192;S-adenosylmethionine synthetase	GO:0006556 S-adenosylmethionine biosynthesis	gg	t
metL	CDS	fused aspartokinase II -!- homoserine dehydrogenase II	Cytoplasmic	COG0527;Aspartokinases -!- COG0460;Homoserine dehydrogenase	GO:0009086 methionine biosynthesis -!- GO:0009090 homoserine biosynthesis	o	tt
metN	CDS	DL-methionine transporter subunit -!- ATP-binding component of ABC superfamily	Cytoplasmic	COG1135;ABC-type metal ion transport system, ATPase component		g	t
metQ	CDS	DL-methionine transporter subunit -!- periplasmic-binding component of ABC superfamily	Membrane Lipoprotein	COG1464;ABC-type metal ion transport system, periplasmic component/surface antigen		o	tt
metR	CDS	DNA-binding transcriptional activator, homocysteine-binding	Cytoplasmic	COG0583;Transcriptional regulator	GO:0009086 methionine biosynthesis -!- GO:0006350 transcription	o	tt
metT	tRNA	tRNA-Met(CAU) (Methionine tRNAm)					
metU	tRNA	tRNA-Met(CAU) (Methionine tRNAm)					
metV	tRNA	tRNA-initiator Met(CAU) (Initiator methionine tRNAf1)					
metW	tRNA	tRNA-initiator Met(CAU) (Initiator methionine tRNAf1)					
metY	tRNA	tRNA-initiator Met(CAU) (Initiator methionine tRNAf2)					
metZ	tRNA	tRNA-initiator Met(CAU) (Initiator methionine tRNAf1)					
mfd	CDS	transcription-repair coupling factor	Cytoplasmic	COG1197;Transcription-repair coupling factor (superfamily II helicase)	GO:0006350 transcription	o	tt
mglA	CDS	fused methyl-galactoside transporter subunitsl -!- ATP-binding components of ABC superfamily	Cytoplasmic	COG1129;ABC-type sugar transport system, ATPase component	GO:0016052 carbohydrate catabolism	o	tt
mglB	CDS	methyl-galactoside transporter subunit -!- periplasmic-binding component of ABC superfamily	Periplasmic	COG1879;ABC-type sugar transport system, periplasmic component	GO:0016052 carbohydrate catabolism -!- GO:0006457 protein	g	tt
mglC	CDS	methyl-galactoside transporter subunit -!- membrane component of ABC superfamily	Integral Membrane Protein	COG4211;ABC-type glucose/galactose transport system, permease component	GO:0016052 carbohydrate catabolism	o	tt
mgsA	CDS	methylglyoxal synthase	Cytoplasmic	COG1803;Methylglyoxal synthase	GO:0009438 methylglyoxal metabolism		
mgtA	CDS	magnesium transporter	Integral Membrane Protein	COG0474;Cation transport ATPase	GO:0015986 ATP synthesis coupled proton transport	o	tt
mhpA	CDS	3-(3-hydroxyphenyl)propionate hydroxylase	Cytoplasmic	COG0654;2-polyprenyl-6-methoxyphenol hydroxylase and related FAD-dependent oxidoreductases	GO:0019395 fatty acid oxidation	gg	t
mhpB	CDS	2,3-dihydroxyphenylpropionate 1,2-dioxygenase	Cytoplasmic		GO:0019395 fatty acid oxidation	gg	t
mhpC	CDS	2-hydroxy-6-ketonona-2,4-dienedioic acid hydrolase	Cytoplasmic		GO:0019395 fatty acid oxidation	gg	o
mhpD	CDS	2-keto-4-pentenoate hydratase	Cytoplasmic	COG3971;2-keto-4-pentenoate hydratase	GO:0019395 fatty acid oxidation	gg	t
mhpE	CDS	4-hyroxy-2-oxovalerate/4-hydroxy-2-oxopentanoic acid aldolase, class I	Cytoplasmic	COG4569;Acetaldehyde dehydrogenase (acetylating)	GO:0019395 fatty acid oxidation	gg	tt
mhpF	CDS	acetaldehyde-CoA dehydrogenase II, NAD-binding	Cytoplasmic	COG0119;Isopropylmalate/homocitrate/citramalate synthases	GO:0019395 fatty acid oxidation	gg	t
mhpR	CDS	DNA-binding transcriptional activator, 3HPP-binding	Cytoplasmic	COG1414;Transcriptional regulator	GO:0019395 fatty acid oxidation -!- GO:0006350 transcription	o	tt
mhpT	CDS	predicted 3-hydroxyphenylpropionic transporter	Integral Membrane Protein	COG0477;Permeases of the major facilitator superfamily	GO:0019395 fatty acid oxidation		
miaA	CDS	delta(2)-isopentenylpyrophosphate tRNA-adenosine transferase	Cytoplasmic	COG0324;tRNA delta(2)-isopentenylpyrophosphate transferase	GO:0009451 RNA modification	o	tt
miaB	CDS	isopentenyl-adenosine A37 tRNA methylthiolase	Cytoplasmic	COG0621;2-methylthioadenine synthetase	GO:0009451 RNA modification		
micC	misc_RNA	regulatory sRNA					
micF	misc_RNA	regulatory sRNA					
minC	CDS	cell division inhibitor	Cytoplasmic	COG0850;Septum formation inhibitor		gg	tt
minD	CDS	membrane ATPase of the MinC-MinD-MinE system	Cytoplasmic	COG2894;Septum formation inhibitor-activating ATPase		gg	tt
minE	CDS	cell division topological specificity factor	Cytoplasmic	COG0851;Septum formation topological specificity factor		gg	t
mioC	CDS	FMN-binding protein MioC	Cytoplasmic	COG0716;Flavodoxins	GO:0006261 DNA dependent DNA replication	gg	t
mipA	CDS	scaffolding protein for murein synthesizing machinery	Outer Membrane B-barrel protein	COG3713;Outer membrane protein V	GO:0009252 peptidoglycan biosynthesis	o	tt
mlrA	CDS	DNA-binding transcriptional regulator	Cytoplasmic	COG0789;Predicted transcriptional regulators	GO:0006350 transcription	gg	o
mltA	CDS	membrane-bound lytic murein transglycosylase A	Outer Membrane Lipoprotein	COG2821;Membrane-bound lytic murein transglycosylase	GO:0009252 peptidoglycan biosynthesis	g	tt
mltB	CDS	membrane-bound lytic murein transglycosylase B	Outer Membrane Lipoprotein	COG2951;Membrane-bound lytic murein transglycosylase B	GO:0009252 peptidoglycan biosynthesis	o	tt
mltC	CDS	membrane-bound lytic murein transglycosylase C	Outer Membrane Lipoprotein	COG0741;Soluble lytic murein transglycosylase and related regulatory proteins (some contain LysM/invasin domains)	GO:0009252 peptidoglycan biosynthesis		
mltD	CDS	predicted membrane-bound lytic murein transglycosylase D	Outer Membrane Lipoprotein	COG0741;Soluble lytic murein transglycosylase and related regulatory proteins (some contain LysM/invasin domains)	GO:0009061 anaerobic respiration -!- GO:0017004 cytochrome biogenesis -!- GO:0006350 transcription		
mmuM	CDS	CP4-6 prophage; S-methylmethionine:homocysteine methyltransferase	Cytoplasmic	COG2040;Homocysteine/selenocysteine methylase (S-methylmethionine-dependent)	GO:0006071 glycerol metabolism -!- GO:0009086 methionine biosynthesis	o	tt
mmuP	CDS	CP4-6 prophage; predicted S-methylmethionine transporter	Integral Membrane Protein		GO:0009086 methionine biosynthesis		
mngA	CDS	fused 2-O-a-mannosyl-D-glycerate specific PTS enzyme IIABC components	Integral Membrane Protein	COG1762;Phosphotransferase system mannitol/fructose-specific IIA domain (Ntr-type) -!- COG1445;Phosphotransferase system fructose-specific component IIB -!- COG1299;Phosphotransferase system, fructose-specific IIC component	GO:0016052 carbohydrate catabolism -!- GO:0006464 protein modification -!- GO:0009266 response to temperature	o	tt
mngB	CDS	alpha-mannosidase	Cytoplasmic	COG0383;Alpha-mannosidase	GO:0016052 carbohydrate	o	tt
mngR	CDS	DNA-binding transcriptional dual regulator, fatty-acyl-binding	Cytoplasmic	COG2188;Transcriptional regulators	GO:0006099 tricarboxylic acid cycle -!- GO:0006350 transcription -!- GO:0006355 regulation of transcription, DNA-dependent	o	tt
mntH	CDS	manganese/divalent cation transporter	Integral Membrane Protein	COG1914;Mn2+ and Fe2+ transporters of the NRAMP family	GO:0006805 xenobiotic metabolism	gg	tt
mntR	CDS	transcriptional regulator of mntH	Cytoplasmic	COG1321;Mn-dependent transcriptional regulator		o	tt
moaA	CDS	molybdopterin biosynthesis protein A	Cytoplasmic	COG2896;Molybdenum cofactor biosynthesis enzyme	GO:0006777 Mo-molybdopterin cofactor biosynthesis	gg	t
moaB	CDS	molybdopterin biosynthesis protein B	Cytoplasmic	COG0521;Molybdopterin biosynthesis enzymes	GO:0006777 Mo-molybdopterin cofactor biosynthesis	gg	t
moaC	CDS	molybdopterin biosynthesis, protein C	Cytoplasmic	COG0315;Molybdenum cofactor biosynthesis enzyme	GO:0006777 Mo-molybdopterin cofactor biosynthesis	gg	t
moaD	CDS	molybdopterin synthase, small subunit	Cytoplasmic	COG1977;Molybdopterin converting factor, small subunit	GO:0006777 Mo-molybdopterin cofactor biosynthesis	gg	t
moaE	CDS	molybdopterin synthase, large subunit	Cytoplasmic	COG0314;Molybdopterin converting factor, large subunit	GO:0006777 Mo-molybdopterin cofactor biosynthesis	gg	o
mobA	CDS	molybdopterin-guanine dinucleotide synthase	Cytoplasmic	COG0746;Molybdopterin-guanine dinucleotide biosynthesis protein A	GO:0006777 Mo-molybdopterin cofactor biosynthesis	gg	o
mobB	CDS	molybdopterin-guanine dinucleotide biosynthesis protein B	Cytoplasmic	COG1763;Molybdopterin-guanine dinucleotide biosynthesis protein	GO:0006777 Mo-molybdopterin cofactor biosynthesis		
modA	CDS	molybdate transporter subunit -!- periplasmic-binding component of ABC superfamily	Periplasmic	COG0725;ABC-type molybdate transport system, periplasmic component	GO:0006777 Mo-molybdopterin cofactor biosynthesis	o	tt
modB	CDS	molybdate transporter subunit -!- membrane component of ABC superfamily	Integral Membrane Protein	COG4149;ABC-type molybdate transport system, permease component		o	tt
modC	CDS	molybdate transporter subunit -!- ATP-binding component of ABC superfamily	Cytoplasmic	COG4148;ABC-type molybdate transport system, ATPase component	GO:0006777 Mo-molybdopterin cofactor biosynthesis	g	t
modE	CDS	DNA-binding transcriptional dual regulator	Cytoplasmic	COG2005;N-terminal domain of molybdenum-binding protein	GO:0006777 Mo-molybdopterin cofactor biosynthesis -!- GO:0006350 transcription	gg	o
modF	CDS	fused subunits of molybdate transporter -!- ATP-binding components of ABC superfamily	Cytoplasmic	COG1119;ABC-type molybdenum transport system, ATPase component/photorepair protein PhrA	GO:0006777 Mo-molybdopterin cofactor biosynthesis	gg	t
moeA	CDS	molybdopterin biosynthesis protein	Cytoplasmic	COG0303;Molybdopterin biosynthesis enzyme	GO:0006777 Mo-molybdopterin cofactor biosynthesis	gg	t
moeB	CDS	molybdopterin synthase sulfurylase	Cytoplasmic	COG0476;Dinucleotide-utilizing enzymes involved in molybdopterin and thiamine biosynthesis family 2	GO:0006777 Mo-molybdopterin cofactor biosynthesis	g	tt
mog	CDS	predicted molybdochelatase	Cytoplasmic	COG0521;Molybdopterin biosynthesis enzymes	GO:0006777 Mo-molybdopterin cofactor biosynthesis	gg	tt
mokB	CDS	regulatory peptide	Cytoplasmic				
mokC	CDS	regulatory protein for HokC, overlaps CDS of hokC	Periplasmic				
molR	CDS	DNA-binding transcriptional regulator, N-ter fragment (pseudogene)	Cytoplasmic			o	o
molR	ancestral	DNA-binding transcriptional regulator (pseudogene)	Cytoplasmic				
molR	CDS	DNA-binding transcriptional regulator, middle fragment (pseudogene)	Cytoplasmic				

molR	CDS	DNA-binding transcriptional regulator, C-ter fragment (pseudogene)	Cytoplasmic				
motA	CDS	proton conductor component of flagella motor	Integral Membrane Protein	COG1291;Flagellar motor component	GO:0042330 taxis	o	tt
motB	CDS	protein that enables flagellar motor rotation	Membrane Anchored	COG1360;Flagellar motor protein	GO:0042330 taxis	o	tt
mpaA	CDS	murein peptide amidase A	Cytoplasmic	COG2866;Predicted carboxypeptidase		gg	t
mpl	CDS	UDP-N-acetylmuramate:L-alanyl-gamma-D-glutamyl-meso-diaminopimelate ligase	Periplasmic	COG0773;UDP-N-acetylmuramate-alanine ligase	GO:0000270 peptidoglycan metabolism	gg	t
mppA	CDS	murein tripeptide (L-ala-gamma-D-glutamyl-meso-DAP) transporter subunit -!- periplasmic-binding component of ABC superfamily	Periplasmic	COG4166;ABC-type oligopeptide transport system, periplasmic component	GO:0000270 peptidoglycan metabolism	gg	tt
mprA	CDS	DNA-binding transcriptional regulator	Cytoplasmic	COG1846;Transcriptional regulators	GO:0006350 transcription -!- GO:0042493 response to drug	o	tt
mqo	CDS	malate dehydrogenase, FAD/NAD(P)-binding domain	Periplasmic	COG0579;Predicted dehydrogenase	GO:0006099 tricarboxylic acid cycle	g	tt
mraW	CDS	S-adenosyl-dependent methyltransferase activity on membrane-located substrates	Cytoplasmic	COG0275;Predicted S-adenosylmethionine-dependent methyltransferase involved in cell envelope biogenesis	GO:0042158 lipoprotein biosynthesis	gg	tt
mraY	CDS	phospho-N-acetylmuramoyl-pentapeptide transferase	Integral Membrane Protein	COG0472;UDP-N-acetylmuramyl pentapeptide phosphotransferase/UDP-N-acetylglucosamine-1-phosp hate transferase	GO:0009252 peptidoglycan biosynthesis	o	tt
mraZ	CDS	conserved protein	Cytoplasmic	COG2001;Uncharacterized protein conserved in bacteria		o	tt
mrcA	CDS	fused penicillin-binding protein 1a murein transglycosylase -!- murein transpeptidase	Membrane Anchored	COG5009;Membrane carboxypeptidase/penicillin-binding protein	GO:0009252 peptidoglycan biosynthesis -!- GO:0042493 response to drug	o	tt
mrcB	CDS	fused glycosyl transferase -!- transpeptidase	Membrane Anchored	COG0744;Membrane carboxypeptidase (penicillin-binding protein)	GO:0046349 amino sugar biosynthesis -!- GO:0009252 peptidoglycan biosynthesis -!- GO:0042493 response to drug	o	tt
mrdA	CDS	transpeptidase involved in peptidoglycan synthesis (penicillin-binding protein 2)	Membrane Anchored	COG0768;Cell division protein FtsI/penicillin-binding protein 2	GO:0009252 peptidoglycan biosynthesis -!- GO:0042493 response to drug	o	tt
mrdB	CDS	cell wall shape-determining protein	Integral Membrane Protein	COG0772;Bacterial cell division membrane protein		o	tt
mreB	CDS	cell wall structural complex MreBCD, actin-like component MreB	Cytoplasmic	COG1077;Actin-like ATPase involved in cell morphogenesis	GO:0042493 response to drug	o	tt
mreC	CDS	cell wall structural complex MreBCD transmembrane component MreC	Membrane Anchored	COG1792;Cell shape-determining protein	GO:0009252 peptidoglycan biosynthesis	o	tt
mreD	CDS	cell wall structural complex MreBCD transmembrane component MreD	Integral Membrane Protein	COG2891;Cell shape-determining protein	GO:0009252 peptidoglycan biosynthesis	o	tt
mrp	CDS	antiporter inner membrane protein	Cytoplasmic			gg	o
mrr	CDS	methylated adenine and cytosine restriction protein	Cytoplasmic	COG1715;Restriction endonuclease	GO:0006306 DNA methylation	o	tt
msbA	CDS	fused lipid transporter subunits -!- membrane component and ATP-binding component of ABC superfamily	Integral Membrane Protein	COG1132;ABC-type multidrug transport system, ATPase and permease components		o	tt
mscL	CDS	mechanosensitive channel	Integral Membrane Protein	COG1970;Large-conductance mechanosensitive channel		o	tt
mscS	CDS	mechanosensitive channel	Integral Membrane Protein	COG0668;Small-conductance mechanosensitive channel		o	tt
msrA	CDS	methionine sulfoxide reductase A	Cytoplasmic	COG0225;Peptide methionine sulfoxide reductase	GO:0006457 protein folding	gg	o
msyB	CDS	predicted protein	Cytoplasmic			o	tt
mtgA	CDS	biosynthetic peptidoglycan transglycosylase	Membrane Anchored	COG0744;Membrane carboxypeptidase (penicillin-binding protein)	GO:0009252 peptidoglycan biosynthesis	o	tt
mtlA	CDS	fused mannitol-specific PTS enzyme IIABC components	Integral Membrane Protein	COG2213;Phosphotransferase system, mannitol-specific IIBC component -!- COG4668;Mannitol/fructose-specific phosphotransferase system, IIA	GO:0016052 carbohydrate catabolism	o	tt
mtlD	CDS	mannitol-1-phosphate dehydrogenase, NAD(P)-binding	Cytoplasmic	COG0246;Mannitol-1-phosphate/altronate dehydrogenases	GO:0016052 carbohydrate catabolism	gg	t
mtlR	CDS	DNA-binding transcriptional repressor	Cytoplasmic	COG3722;Transcriptional regulator	GO:0016052 carbohydrate catabolism -!- GO:0006350	gg	o
mtr	CDS	tryptophan transporter of high affinity	Integral Membrane Protein	COG0814;Amino acid permeases	GO:0000162 tryptophan biosynthesis	o	tt
mukB	CDS	fused chromosome partitioning proteins	Cytoplasmic	COG3096;Uncharacterized protein involved in chromosome partitioning		o	tt
mukE	CDS	protein involved in chromosome partitioning	Cytoplasmic	COG3095;Uncharacterized protein involved in chromosome partitioning		gg	t
mukF	CDS	Involved in chromosome partioning, Ca2+ binding protein	Cytoplasmic	COG3006;Uncharacterized protein involved in chromosome partitioning		gg	tt
murA	CDS	UDP-N-acetylglucosamine 1-carboxyvinyltransferase	Cytoplasmic	COG0766;UDP-N-acetylglucosamine enolpyruvyl transferase	GO:0009252 peptidoglycan biosynthesis	o	tt
murB	CDS	UDP-N-acetylenolpyruvoylglucosamine reductase, FAD-binding	Cytoplasmic	COG0812;UDP-N-acetylmuramate dehydrogenase	GO:0009252 peptidoglycan biosynthesis	gg	t
murC	CDS	UDP-N-acetylmuramate:L-alanine ligase	Cytoplasmic	COG0773;UDP-N-acetylmuramate-alanine ligase	GO:0009252 peptidoglycan biosynthesis	g	o
murD	CDS	UDP-N-acetylmuramoyl-L-alanine:D-glutamate ligase	Cytoplasmic	COG0771;UDP-N-acetylmuramoylalanine-D-glutamate ligase	GO:0009252 peptidoglycan biosynthesis	gg	o
murE	CDS	UDP-N-acetylmuramoyl-L-alanyl-D-glutamate:meso-diaminopimelate ligase	Cytoplasmic	COG0769;UDP-N-acetylmuramyl tripeptide synthase	GO:0009252 peptidoglycan biosynthesis	gg	t
murF	CDS	UDP-N-acetylmuramoyl-tripeptide:D-alanyl-D-alanine ligase	Cytoplasmic	COG0770;UDP-N-acetylmuramyl pentapeptide synthase	GO:0009252 peptidoglycan biosynthesis -!- GO:0000270 peptidoglycan metabolism	gg	t
murG	CDS	N-acetylglucosaminyl transferase	Membrane Associated	COG0707;UDP-N-acetylglucosamine:LPS N-acetylglucosamine transferase	GO:0009252 peptidoglycan biosynthesis	o	tt
murI	CDS	glutamate racemase	Cytoplasmic	COG0796;Glutamate racemase	GO:0009252 peptidoglycan biosynthesis		
murP	CDS	fused predicted enzyme IIBC components of PTS	Integral Membrane Protein	COG1264;Phosphotransferase system IIB components -!- COG1263;Phosphotransferase system IIC components, glucose/maltose/N-acetylglucosamine-specific	GO:0016052 carbohydrate catabolism	o	tt
mutH	CDS	methyl-directed mismatch repair protein	Cytoplasmic	COG3066;DNA mismatch repair protein	GO:0006281 DNA repair	o	o
mutL	CDS	methyl-directed mismatch repair protein	Cytoplasmic	COG0323;DNA mismatch repair enzyme (predicted ATPase)	GO:0006281 DNA repair	o	o
mutM	CDS	formamidopyrimidine/5-formyluracil/ 5-hydroxymethyluracil DNA glycosylase	Cytoplasmic	COG0266;Formamidopyrimidine-DNA glycosylase	GO:0006281 DNA repair	o	tt
mutS	CDS	methyl-directed mismatch repair protein	Cytoplasmic	COG0249;Mismatch repair ATPase (MutS family)	GO:0006281 DNA repair	o	tt
mutT	CDS	nucleoside triphosphate pyrophosphohydrolase, marked preference for dGTP	Cytoplasmic	COG0494;NTP pyrophosphohydrolases including oxidative damage repair enzymes	GO:0009265 2'-deoxyribonucleotide biosynthesis	gg	o
mutY	CDS	adenine DNA glycosylase	Cytoplasmic	COG1194;A/G-specific DNA glycosylase	GO:0006281 DNA repair	g	tt
mviM	CDS	predicted oxidoreductase, NAD(P)-binding Rossmann-fold domain	Periplasmic	COG0673;Predicted dehydrogenases and related proteins		gg	t
mviN	CDS	predicted inner membrane protein	Integral Membrane Protein	COG0728;Uncharacterized membrane protein, putative virulence factor		o	tt
nac	CDS	DNA-binding transcriptional dual regulator	Cytoplasmic	COG0583;Transcriptional regulator	GO:0006807 nitrogen metabolism -!- GO:0006350 transcription	o	tt
nadA	CDS	quinolinate synthase, subunit A	Cytoplasmic	COG0379;Quinolinate synthase	GO:0009435 nicotinamide adenine dinucleotide biosynthesis	gg	t
nadB	CDS	quinolinate synthase, L-aspartate oxidase (B protein) subunit	Cytoplasmic	COG0029;Aspartate oxidase	GO:0009435 nicotinamide adenine dinucleotide biosynthesis	gg	t
nadC	CDS	quinolinate phosphoribosyltransferase	Cytoplasmic	COG0157;Nicotinate-nucleotide pyrophosphorylase	GO:0009435 nicotinamide adenine dinucleotide biosynthesis	gg	o
nadD	CDS	nicotinic acid mononucleotide adenylyltransferase, NAD(P)-dependent	Cytoplasmic	COG1057;Nicotinic acid mononucleotide adenylyltransferase	GO:0009435 nicotinamide adenine dinucleotide biosynthesis	gg	o
nadE	CDS	NAD synthetase, NH3/glutamine-dependent	Cytoplasmic	COG0171;NAD synthase	GO:0009435 nicotinamide adenine dinucleotide biosynthesis	gg	o
nadR	CDS	bifunctional DNA-binding transcriptional repressor -!- NMN adenylyltransferase	Cytoplasmic	COG1396;Predicted transcriptional regulators -!- COG1056;Nicotinamide mononucleotide adenylyltransferase -!- COG3172;Predicted ATPase/kinase involved in NAD metabolism	GO:0009435 nicotinamide adenine dinucleotide biosynthesis -!- GO:0006350 transcription		
nagA	CDS	N-acetylglucosamine-6-phosphate deacetylase	Cytoplasmic	COG1820;N-acetylglucosamine-6-phosphate deacetylase	GO:0046349 amino sugar biosynthesis	gg	t
nagB	CDS	glucosamine-6-phosphate deaminase	Cytoplasmic	COG0363;6-phosphogluconolactonase/Glucosamine-6-ph osphate isomerase/deaminase	GO:0046349 amino sugar biosynthesis	gg	o
nagC	CDS	DNA-binding transcriptional dual regulator	Cytoplasmic	COG1940;Transcriptional regulator/sugar kinase	GO:0046349 amino sugar biosynthesis -!- GO:0006350 transcription -!- GO:0009386 translational attenuation	gg	tt
nagD	CDS	UMP phosphatase	Cytoplasmic	COG0647;Predicted sugar phosphatases of the HAD superfamily	GO:0046349 amino sugar biosynthesis	gg	t
nagE	CDS	fused N-acetyl glucosamine specific PTS enzyme IICBA components	Integral Membrane Protein	COG1263;Phosphotransferase system IIC components, glucose/maltose/N-acetylglucosamine-specific -!- COG1264;Phosphotransferase system IIB components -!-	GO:0009310 amine catabolism -!- GO:0046349 amino sugar biosynthesis	o	tt
nagZ	CDS	beta N-acetyl-glucosaminidase	Cytoplasmic	COG1472;Beta-glucosidase-related glycosidases	GO:0000270 peptidoglycan metabolism	gg	t
nanA	CDS	N-acetylneuraminate lyase	Cytoplasmic	COG0329;Dihydrodipicolinate synthase/N-acetylneuraminate lyase	GO:0046349 amino sugar biosynthesis	g	t
nanE	CDS	predicted N-acetylmannosamine-6-P epimerase	Cytoplasmic	COG3010;Putative N-acetylmannosamine-6-phosphate epimerase	GO:0046349 amino sugar biosynthesis	gg	o
nanK	CDS	predicted N-acetylmannosamine kinase	Cytoplasmic		GO:0046349 amino sugar biosynthesis		
nanR	CDS	DNA-binding transcriptional dual regulator	Cytoplasmic		GO:0006350 transcription	o	tt
nanT	CDS	sialic acid transporter	Integral Membrane Protein		GO:0046349 amino sugar biosynthesis	o	tt
napA	CDS	nitrate reductase, periplasmic, large subunit	Periplasmic	COG0243;Anaerobic dehydrogenases, typically selenocysteine-containing	GO:0009061 anaerobic respiration	gg	tt
napB	CDS	nitrate reductase, small, cytochrome C550 subunit, periplasmic	Periplasmic		GO:0009061 anaerobic respiration -!- GO:0017004 cytochrome biogenesis		
napC	CDS	nitrate reductase, cytochrome c-type,periplasmic	Periplasmic with N-terminal Membrane	COG3005;Nitrate/TMAO reductases, membrane-bound tetraheme cytochrome c subunit	GO:0009061 anaerobic respiration -!- GO:0017004 cytochrome biogenesis	o	tt
napD	CDS	assembly protein for periplasmic nitrate reductase	Cytoplasmic	COG3062;Uncharacterized protein involved in formation of periplasmic nitrate reductase	GO:0009061 anaerobic respiration -!- GO:0006464 protein modification	gg	t
napF	CDS	ferredoxin-type protein, predicted role in electron transfer to periplasmic nitrate reductase (NapA)	Cytoplasmic	COG1145;Ferredoxin	GO:0009061 anaerobic respiration	o	o
napG	CDS	ferredoxin-type protein essential for electron transfer from ubiquinol to periplasmic nitrate reductase (NapAB)	Periplasmic	COG1145;Ferredoxin	GO:0009061 anaerobic respiration	g	t
napH	CDS	ferredoxin-type protein essential for electron transfer from ubiquinol to periplasmic nitrate reductase (NapAB)	Integral Membrane Protein	COG0348;Polyferredoxin	GO:0009061 anaerobic respiration	o	tt

narG	CDS	nitrate reductase 1, alpha subunit	Cytoplasmic	COG5013;Nitrate reductase alpha subunit	GO:0009061 anaerobic respiration	o	tt
narH	CDS	nitrate reductase 1, beta (Fe-S) subunit	Cytoplasmic	COG1140;Nitrate reductase beta subunit	GO:0009061 anaerobic respiration	gg	t
narI	CDS	nitrate reductase 1, gamma (cytochrome b(NR)) subunit	Integral Membrane Protein	COG2181;Nitrate reductase gamma subunit	GO:0009061 anaerobic respiration -!- GO:0017004 cytochrome biogenesis	o	tt
narJ	CDS	molybdenum-cofactor-assembly chaperone subunit (delta subunit) of nitrate reductase 1	Cytoplasmic	COG2180;Nitrate reductase delta subunit	GO:0009061 anaerobic respiration -!- GO:0006457 protein folding	gg	t
narK	CDS	nitrate/nitrite transporter	Integral Membrane Protein	COG2223;Nitrate/nitrite transporter	GO:0006807 nitrogen metabolism	o	tt
narL	CDS	DNA-binding response regulator in two-component regulatory system with NarX (or NarQ)	Cytoplasmic	COG2197;Response regulator containing a CheY-like receiver domain and an HTH DNA-binding domain	GO:0009061 anaerobic respiration -!- GO:0006350 transcription	gg	t
narP	CDS	DNA-binding response regulator in two-component regulatory system with NarQ or NarX	Cytoplasmic	COG2197;Response regulator containing a CheY-like receiver domain and an HTH DNA-binding domain	GO:0009061 anaerobic respiration -!- GO:0006350 transcription	o	tt
narQ	CDS	sensory histidine kinase in two-component regulatory system with NarP (NarL)	Integral Membrane Protein	COG3850;Signal transduction histidine kinase, nitrate/nitrite-specific	GO:0009061 anaerobic respiration -!- GO:0006464 protein modification	o	tt
narU	CDS	nitrate/nitrite transporter	Integral Membrane Protein	COG2223;Nitrate/nitrite transporter	GO:0006807 nitrogen metabolism	o	tt
narV	CDS	nitrate reductase 2 (NRZ), gamma subunit	Integral Membrane Protein	COG2181;Nitrate reductase gamma subunit	GO:0009061 anaerobic respiration	o	tt
narW	CDS	nitrate reductase 2 (NRZ), delta subunit (assembly subunit)	Cytoplasmic	COG2180;Nitrate reductase delta subunit	GO:0009061 anaerobic respiration -!- GO:0006457 protein folding	gg	tt
narX	CDS	sensory histidine kinase in two-component regulatory system with NarL	Integral Membrane Protein	COG3850;Signal transduction histidine kinase, nitrate/nitrite-specific	GO:0009061 anaerobic respiration -!- GO:0006464 protein modification	o	tt
narY	CDS	nitrate reductase 2 (NRZ), beta subunit	Cytoplasmic	COG1140;Nitrate reductase beta subunit	GO:0009061 anaerobic respiration	gg	t
narZ	CDS	nitrate reductase 2 (NRZ), alpha subunit	Cytoplasmic	COG5013;Nitrate reductase alpha subunit	GO:0009061 anaerobic respiration	o	tt
ndh	CDS	respiratory NADH dehydrogenase 2/cupric reductase	Cytoplasmic	COG1252;NADH dehydrogenase, FAD-containing subunit	GO:0009060 aerobic respiration -!- GO:0009061 anaerobic respiration	gg	tt
ndk	CDS	multifunctional nucleoside diphosphate kinase -!- apyrimidinic endonuclease -!- 3'-phosphodiesterase	Cytoplasmic	COG0105;Nucleoside diphosphate kinase	GO:0015949 nucleobase, nucleoside and nucleotide interconversion -!- GO:0009152 purine ribonucleotide biosynthesis	gg	t
nei	CDS	endonuclease VIII -!- 5-formyluracil/5-hydroxymethyluracil DNA glycosylase	Cytoplasmic	COG0266;Formamidopyrimidine-DNA glycosylase	GO:0006281 DNA repair	o	tt
nemA	CDS	N-ethylmaleimide reductase, FMN-linked	Cytoplasmic	COG1902;NADH:flavin oxidoreductases, Old Yellow Enzyme family	GO:0006805 xenobiotic metabolism	gg	t
nfi	CDS	endonuclease V	Cytoplasmic	COG1515;Deoxyinosine 3'endonuclease (endonuclease V)	GO:0006308 DNA catabolism -!- GO:0006308 DNA catabolism		
nfnB	CDS	dihydropteridine reductase, NAD(P)H-dependent, oxygen-insensitive	Cytoplasmic	COG0778;Nitroreductase	GO:0042493 response to drug	gg	t
nfo	CDS	endonuclease IV with intrinsic 3'-5' exonuclease activity	Periplasmic	COG0648;Endonuclease IV	GO:0006308 DNA catabolism -!- GO:0006308 DNA catabolism -!- GO:0009314 response to radiation	gg	t
nfrA	CDS	bacteriophage N4 receptor, outer membrane subunit	Outer Membrane B-barrel protein	COG0457;FOG: TPR repeat		o	tt
nfrB	CDS	bacteriophage N4 receptor, inner membrane subunit	Integral Membrane Protein			o	tt
nfsA	CDS	nitroreductase A, NADPH-dependent, FMN-	Cytoplasmic	COG0778;Nitroreductase	GO:0009061 anaerobic respiration	gg	t
nhaA	CDS	sodium-proton antiporter	Integral Membrane Protein	COG3004;Na+/H+ antiporter	GO:0009268 response to pH	o	tt
nhaB	CDS	sodium:proton antiporter	Integral Membrane Protein	COG3067;Na+/H+ antiporter	GO:0009268 response to pH	o	tt
nhaR	CDS	DNA-binding transcriptional activator	Cytoplasmic	COG0583;Transcriptional regulator	GO:0006350 transcription -!- GO:0009268 response to pH	o	tt
nhoA	CDS	N-hydroxyarylamine O-acetyltransferase	Cytoplasmic	COG2162;Arylamine N-acetyltransferase	GO:0008152 metabolism	gg	o
nikA	CDS	nickel transporter subunit -!- periplasmic-binding component of ABC superfamily	Periplasmic	COG0747;ABC-type dipeptide transport system, periplasmic component	GO:0042330 taxis	o	tt
nikB	CDS	nickel transporter subunit -!- membrane component of ABC superfamily	Integral Membrane Protein	COG0601;ABC-type dipeptide/oligopeptide/nickel transport systems, permease components		o	tt
nikC	CDS	nickel transporter subunit -!- membrane component of ABC superfamily	Integral Membrane Protein	COG1173;ABC-type dipeptide/oligopeptide/nickel transport systems, permease components		o	tt
nikD	CDS	nickel transporter subunit -!- ATP-binding component of ABC superfamily	Cytoplasmic	COG0444;ABC-type dipeptide/oligopeptide/nickel transport system, ATPase component	GO:0009060 aerobic respiration -!- GO:0009061 anaerobic respiration	gg	t
nikE	CDS	nickel transporter subunit -!- ATP-binding component of ABC superfamily	Cytoplasmic	COG1124;ABC-type dipeptide/oligopeptide/nickel transport system, ATPase component	GO:0009060 aerobic respiration -!- GO:0009061 anaerobic respiration	gg	tt
nikR	CDS	DNA-binding transcriptional regulator, Ni-binding	Cytoplasmic	COG0864;Predicted transcriptional regulators containing the CopG/Arc/MetJ DNA-binding domain and a metal-binding domain	GO:0006350 transcription	g	o
ninE	CDS	DLP12 prophage; conserved protein	Cytoplasmic			gg	tt
nirB	CDS	nitrite reductase, large subunit, NAD(P)H-binding	Cytoplasmic	COG1251;NAD(P)H-nitrite reductase	GO:0009061 anaerobic respiration	o	tt
nirC	CDS	nitrite transporter	Integral Membrane Protein		GO:0006807 nitrogen metabolism	o	tt
nirD	CDS	nitrite reductase, NAD(P)H-binding, small subunit	Cytoplasmic	COG2146;Ferredoxin subunits of nitrite reductase and ring-hydroxylating dioxygenases	GO:0009061 anaerobic respiration	gg	tt
nlpA	CDS	cytoplasmic membrane lipoprotein-28	Inner Membrane Lipoprotein	COG1464;ABC-type metal ion transport system, periplasmic component/surface antigen		o	tt
nlpB	CDS	lipoprotein	Outer Membrane Lipoprotein			o	tt
nlpC	CDS	predicted lipoprotein	Outer Membrane Lipoprotein	COG0791;Cell wall-associated hydrolases (invasion-associated proteins)		o	tt
nlpD	CDS	predicted outer membrane lipoprotein	Outer Membrane Lipoprotein	COG0739;Membrane proteins related to metalloendopeptidases		o	tt
nlpE	CDS	lipoprotein involved with copper homeostasis and adhesion	Outer Membrane Lipoprotein	COG3015;Uncharacterized lipoprotein NlpE involved in copper resistance	GO:0006805 xenobiotic metabolism	o	tt
nlpI	CDS	conserved protein	Outer Membrane Lipoprotein	COG4785;Lipoprotein NlpI, contains TPR repeats		o	tt
nmpC	CDS	DLP12 prophage; truncated outer membrane porin (pseudogene)	Outer Membrane B-barrel protein	COG3203;Outer membrane protein (porin)			
nohA	CDS	Qin prophage; predicted packaging protein	Cytoplasmic	COG4220;Phage DNA packaging protein, Nu1 subunit of terminase		gg	o
nohB	CDS	DLP12 prophage; DNA packaging protein	Cytoplasmic	COG4220;Phage DNA packaging protein, Nu1 subunit of terminase		gg	t
norR	CDS	DNA-binding transcriptional activator	Cytoplasmic	COG3604;Transcriptional regulator containing GAF, AAA-type ATPase, and DNA binding domains	GO:0006350 transcription		
norV	CDS	flavorubredoxin oxidoreductase	Cytoplasmic	COG0426;Uncharacterized flavoproteins -!- COG1773;Rubredoxin		gg	t
norW	CDS	NADH:flavorubredoxin oxidoreductase	Cytoplasmic	COG0446;Uncharacterized NAD(FAD)-dependent dehydrogenases		gg	t
npr	CDS	phosphohistidinoprotein-hexose phosphotransferase component of N-regulated PTS system (Npr)	Cytoplasmic	COG1925;Phosphotransferase system, HPr-related proteins	GO:0006464 protein modification	g	o
nrdA	CDS	ribonucleoside diphosphate reductase 1, alpha subunit	Cytoplasmic	COG0209;Ribonucleotide reductase, alpha subunit	GO:0015949 nucleobase, nucleoside and nucleotide interconversion	gg	t
nrdB	CDS	ribonucleoside diphosphate reductase 1, beta subunit, ferritin-like	Cytoplasmic	COG0208;Ribonucleotide reductase, beta subunit	GO:0015949 nucleobase, nucleoside and nucleotide interconversion	gg	t
nrdD	CDS	anaerobic ribonucleoside-triphosphate reductase	Cytoplasmic	COG1328;Oxygen-sensitive ribonucleoside-triphosphate reductase	GO:0009265 2'-deoxyribonucleotide biosynthesis	gg	t
nrdE	CDS	ribonucleoside-diphosphate reductase 2, alpha subunit	Cytoplasmic	COG0209;Ribonucleotide reductase, alpha subunit	GO:0015949 nucleobase, nucleoside and nucleotide interconversion		
nrdF	CDS	ribonucleoside-diphosphate reductase 2, beta subunit, ferritin-like	Cytoplasmic	COG0208;Ribonucleotide reductase, beta subunit	GO:0015949 nucleobase, nucleoside and nucleotide interconversion	gg	t
nrdG	CDS	anaerobic ribonucleotide reductase activating protein	Cytoplasmic	COG0602;Organic radical activating enzymes	GO:0015949 nucleobase, nucleoside and nucleotide interconversion -!- GO:0006464 protein modification	g	o
nrdH	CDS	glutaredoxin-like protein	Cytoplasmic	COG0695;Glutaredoxin and related proteins			
nrdI	CDS	protein that stimulates ribonucleotide reduction	Cytoplasmic	COG1780;Protein involved in ribonucleotide reduction		gg	o
nrfA	CDS	nitrite reductase, formate-dependent, cytochrome	Periplasmic	COG3303;Formate-dependent nitrite reductase, periplasmic cytochrome c552 subunit	GO:0009061 anaerobic respiration -!- GO:0017004 cytochrome biogenesis	o	tt
nrfB	CDS	nitrite reductase, formate-dependent, penta-heme cytochrome c	Periplasmic		GO:0009061 anaerobic respiration -!- GO:0017004 cytochrome biogenesis	o	tt
nrfC	CDS	formate-dependent nitrite reductase, 4Fe4S subunit	Periplasmic	COG0437;Fe-S-cluster-containing hydrogenase components 1	GO:0009061 anaerobic respiration	gg	t
nrfD	CDS	formate-dependent nitrite reductase, membrane subunit	Integral Membrane Protein	COG3301;Formate-dependent nitrite reductase, membrane component	GO:0009061 anaerobic respiration	o	tt
nrfE	CDS	heme lyase (NrfEFG) for insertion of heme into c552, subunit NrfE	Integral Membrane Protein	COG1138;Cytochrome c biogenesis factor	GO:0009061 anaerobic respiration -!- GO:0017004 cytochrome biogenesis -!- GO:0006457 protein folding	o	tt
nrfF	CDS	heme lyase (NrfEFG) for insertion of heme into c552, subunit NrfF	Membrane Anchored	COG3088;Uncharacterized protein involved in biosynthesis of c-type cytochromes	GO:0009061 anaerobic respiration -!- GO:0017004 cytochrome biogenesis -!- GO:0006457 protein folding	o	tt
nrfG	CDS	heme lyase (NrfEFG) for insertion of heme into c552, subunit NrfG	Outer Membrane Lipoprotein	COG4235;Cytochrome c biogenesis factor	GO:0009061 anaerobic respiration -!- GO:0017004 cytochrome biogenesis -!- GO:0006457 protein folding	o	tt
nth	CDS	DNA glycosylase and apyrimidinic (AP) lyase (endonuclease III)	Cytoplasmic	COG0177;Predicted EndoIII-related endonuclease	GO:0006308 DNA catabolism -!- GO:0006308 DNA catabolism -!- GO:0009314 response to radiation	gg	t
nudB	CDS	dATP pyrophosphohydrolase	Cytoplasmic	COG0494;NTP pyrophosphohydrolases including oxidative damage repair enzymes	GO:0015949 nucleobase, nucleoside and nucleotide interconversion	o	o
nudC	CDS	NADH pyrophosphatase	Cytoplasmic				
nudD	CDS	GDP-mannose mannosyl hydrolase	Cytoplasmic	COG0494;NTP pyrophosphohydrolases including oxidative damage repair enzymes	GO:0009226 nucleotide-sugar biosynthesis -!- GO:0009242 colanic acid biosynthesis		
nudE	CDS	ADP-ribose diphosphatase	Cytoplasmic	COG0494;NTP pyrophosphohydrolases including oxidative damage repair enzymes		g	t
nudF	CDS	ADP-ribose pyrophosphatase	Cytoplasmic	COG0494;NTP pyrophosphohydrolases including oxidative damage repair enzymes		gg	tt
nudG	CDS	pyrimidine (deoxy)nucleoside triphosphate pyrophosphohydrolase	Cytoplasmic	COG0494;NTP pyrophosphohydrolases including oxidative damage repair enzymes		gg	tt
nudH	CDS	nucleotide hydrolase	Cytoplasmic	COG0494;NTP pyrophosphohydrolases including oxidative damage repair enzymes		g	tt
nuoA	CDS	NADH:ubiquinone oxidoreductase, membrane subunit A	Integral Membrane Protein		GO:0009060 aerobic respiration -!- GO:0009061 anaerobic respiration	o	tt
nuoB	CDS	NADH:ubiquinone oxidoreductase, chain B	Cytoplasmic	COG0377;NADH:ubiquinone oxidoreductase 20 kD subunit and related Fe-S oxidoreductases	GO:0009060 aerobic respiration -!- GO:0009061 anaerobic respiration		
nuoC	CDS	NADH:ubiquinone oxidoreductase, chain C,D	Cytoplasmic	COG0852;NADH:ubiquinone oxidoreductase 27 kD subunit -!- COG0649;NADH:ubiquinone oxidoreductase 49 kD subunit 7	GO:0009060 aerobic respiration -!- GO:0009061 anaerobic respiration		
nuoE	CDS	NADH:ubiquinone oxidoreductase, chain E	Cytoplasmic	COG1905;NADH:ubiquinone oxidoreductase 24 kD subunit	GO:0009060 aerobic respiration -!- GO:0009061 anaerobic respiration	gg	o

nuoF	CDS	NADH:ubiquinone oxidoreductase, chain F	Cytoplasmic	COG1894;NADH:ubiquinone oxidoreductase, NADH-binding (51 kD) subunit	GO:0009060 aerobic respiration -!- GO:0009061 anaerobic respiration	gg	t
nuoG	CDS	NADH:ubiquinone oxidoreductase, chain G	Cytoplasmic		GO:0009060 aerobic respiration -!- GO:0009061 anaerobic respiration	o	tt
nuoH	CDS	NADH:ubiquinone oxidoreductase, membrane subunit H	Integral Membrane Protein	COG1005;NADH:ubiquinone oxidoreductase subunit 1 (chain H)	GO:0009060 aerobic respiration -!- GO:0009061 anaerobic respiration	o	tt
nuoI	CDS	NADH:ubiquinone oxidoreductase, chain I	Cytoplasmic	COG1143;Formate hydrogenlyase subunit 6/NADH:ubiquinone oxidoreductase 23 kD subunit (chain I)	GO:0009060 aerobic respiration -!- GO:0009061 anaerobic respiration	o	t
nuoJ	CDS	NADH:ubiquinone oxidoreductase, membrane subunit J	Integral Membrane Protein	COG0839;NADH:ubiquinone oxidoreductase subunit 6 (chain J)	GO:0009060 aerobic respiration -!- GO:0009061 anaerobic respiration	gg	tt
nuoK	CDS	NADH:ubiquinone oxidoreductase, membrane subunit K	Integral Membrane Protein	COG0713;NADH:ubiquinone oxidoreductase subunit 11 or 4L (chain K)	GO:0009060 aerobic respiration -!- GO:0009061 anaerobic respiration	o	tt
nuoL	CDS	NADH:ubiquinone oxidoreductase, membrane subunit L	Integral Membrane Protein	COG1009;NADH:ubiquinone oxidoreductase subunit 5 (chain L)/Multisubunit Na+/H+ antiporter, MnhA subunit	GO:0009060 aerobic respiration -!- GO:0009061 anaerobic respiration	o	tt
nuoM	CDS	NADH:ubiquinone oxidoreductase, membrane subunit M	Integral Membrane Protein	COG1008;NADH:ubiquinone oxidoreductase subunit 4 (chain M)	GO:0009060 aerobic respiration -!- GO:0009061 anaerobic respiration	o	tt
nuoN	CDS	NADH:ubiquinone oxidoreductase, membrane subunit N	Integral Membrane Protein	COG1007;NADH:ubiquinone oxidoreductase subunit 2 (chain N)	GO:0009060 aerobic respiration -!- GO:0009061 anaerobic respiration	o	tt
nupC	CDS	nucleoside (except guanosine) transporter	Integral Membrane Protein	COG1972;Nucleoside permease	GO:0015949 nucleobase, nucleoside and nucleotide interconversion	g	tt
nupG	CDS	nucleoside transporter	Integral Membrane Protein		GO:0015949 nucleobase, nucleoside and nucleotide interconversion	g	tt
nusA	CDS	transcription termination/antitermination L factor	Cytoplasmic	COG0195;Transcription elongation factor	GO:0006350 transcription -!- GO:0006355 regulation of transcription, DNA-dependent	gg	t
nusB	CDS	transcription antitermination protein	Cytoplasmic	COG0781;Transcription termination factor	GO:0006350 transcription	gg	o
nusG	CDS	transcription termination factor	Cytoplasmic	COG0250;Transcription antiterminator	GO:0006350 transcription	o	tt
obgE	CDS	GTPase involved in cell partioning and DNA repair	Cytoplasmic	COG0536;Predicted GTPase		gg	tt
ogrK	CDS	DNA-binding transcriptional regulator -!-prophage P2 remnant	Cytoplasmic			gg	o
ogt	CDS	O-6-alkylguanine-DNA:cysteine-protein methyltransferase	Cytoplasmic	COG0350;Methylated DNA-protein cysteine methyltransferase	GO:0006306 DNA methylation	gg	t
ompA	CDS	outer membrane protein A (3a;II*;G;d)	Outer Membrane B-barrel protein	COG2885;Outer membrane protein and related peptidoglycan-associated (lipo)proteins		o	o
ompC	CDS	outer membrane porin protein C	Outer Membrane B-barrel protein	COG3203;Outer membrane protein (porin)		o	tt
ompF	CDS	outer membrane porin 1a (Ia;b;F)	Outer Membrane B-barrel protein	COG3203;Outer membrane protein (porin)		o	tt
ompG	CDS	outer membrane porin	Outer Membrane B-barrel protein			o	tt
ompL	CDS	predicted outer membrane porin L	Outer Membrane B-barrel protein			g	o
ompN	CDS	outer membrane pore protein N, non-specific	Outer Membrane B-barrel protein	COG3203;Outer membrane protein (porin)		o	tt
ompR	CDS	DNA-binding response regulator in two-component regulatory system with EnvZ	Cytoplasmic	COG0745;Response regulators consisting of a CheY-like receiver domain and a winged-helix DNA-binding domain	GO:0006350 transcription	o	tt
ompT	CDS	DLP12 prophage; outer membrane protease VII (outer membrane protein 3b)	Outer Membrane B-barrel protein	COG4571;Outer membrane protease		o	tt
ompW	CDS	outer membrane protein W	Outer Membrane B-barrel protein	COG3047;Outer membrane protein W		o	tt
ompX	CDS	outer membrane protein	Outer Membrane B-barrel protein	COG3637;Opacity protein and related surface antigens		o	tt
oppA	CDS	oligopeptide transporter subunit -!- periplasmic-binding component of ABC superfamily	Periplasmic	COG4166;ABC-type oligopeptide transport system, periplasmic component	GO:0000270 peptidoglycan metabolism -!- GO:0006457 protein folding	o	tt
oppB	CDS	oligopeptide transporter subunit -!- membrane component of ABC superfamily	Integral Membrane Protein	COG0601;ABC-type dipeptide/oligopeptide/nickel transport systems, permease components	GO:0000270 peptidoglycan metabolism	o	tt
oppC	CDS	oligopeptide transporter subunit -!- membrane component of ABC superfamily	Integral Membrane Protein	COG1173;ABC-type dipeptide/oligopeptide/nickel transport systems, permease components	GO:0000270 peptidoglycan metabolism	o	tt
oppD	CDS	oligopeptide transporter subunit -!- ATP-binding component of ABC superfamily	Cytoplasmic		GO:0000270 peptidoglycan metabolism	g	o
oppF	CDS	oligopeptide transporter subunit -!- ATP-binding component of ABC superfamily	Cytoplasmic	COG4608;ABC-type oligopeptide transport system, ATPase component	GO:0000270 peptidoglycan metabolism	gg	tt
oriC	rep_origin	origin of replication					
orn	CDS	oligoribonuclease	Cytoplasmic		GO:0006401 RNA catabolism		
osmB	CDS	lipoprotein	Outer Membrane Lipoprotein		GO:0006970 response to osmotic stress	o	tt
osmC	CDS	osmotically inducible, stress-inducible membrane protein	Cytoplasmic	COG1764;Predicted redox protein, regulator of disulfide bond formation	GO:0006970 response to osmotic stress	gg	t
osmE	CDS	DNA-binding transcriptional regulator	Outer Membrane Lipoprotein		GO:0006350 transcription -!- GO:0006970 response to osmotic stress	o	tt
osmY	CDS	periplasmic protein	Periplasmic	COG2823;Predicted periplasmic or secreted lipoprotein	GO:0006970 response to osmotic stress	o	tt
otsA	CDS	trehalose-6-phosphate synthase	Cytoplasmic	COG0380;Trehalose-6-phosphate synthase	GO:0006006 glucose metabolism -!- GO:0006970 response to osmotic stress		
otsB	CDS	trehalose-6-phosphate phosphatase, biosynthetic	Cytoplasmic	COG1877;Trehalose-6-phosphatase	GO:0006006 glucose metabolism -!- GO:0006970 response to osmotic stress	gg	o
oxc	CDS	predicted oxalyl-CoA decarboxylase	Cytoplasmic	COG0028;Thiamine pyrophosphate-requiring enzymes [acetolactate synthase, pyruvate dehydrogenase (cytochrome), glyoxylate carboligase, phosphonopyruvate decarboxylase]		gg	tt
oxyR	CDS	DNA-binding transcriptional dual regulator	Cytoplasmic	COG0583;Transcriptional regulator	GO:0006350 transcription	gg	o
oxyS	misc_RNA	regulatory sRNA, global					
paaA	CDS	predicted multicomponent oxygenase/reductase subunit for phenylacetic acid degradation	Cytoplasmic	COG3396;Uncharacterized conserved protein	GO:0016052 carbohydrate catabolism	gg	t
paaB	CDS	predicted multicomponent oxygenase/reductase subunit for phenylacetic acid degradation	Cytoplasmic	COG3460;Uncharacterized enzyme of phenylacetate metabolism	GO:0016052 carbohydrate catabolism	gg	o
paaC	CDS	predicted multicomponent oxygenase/reductase subunit for phenylacetic acid degradation	Cytoplasmic	COG3396;Uncharacterized conserved protein	GO:0016052 carbohydrate catabolism	gg	t
paaD	CDS	predicted multicomponent oxygenase/reductase subunit for phenylacetic acid degradation	Cytoplasmic	COG2151;Predicted metal-sulfur cluster biosynthetic enzyme	GO:0016052 carbohydrate catabolism		
paaE	CDS	predicted multicomponent oxygenase/reductase subunit for phenylacetic acid degradation	Cytoplasmic	COG1018;Flavodoxin reductases (ferredoxin-NADPH reductases) family 1	GO:0016052 carbohydrate catabolism	o	tt
paaF	CDS	enoyl-CoA hydratase-isomerase	Cytoplasmic	COG1024;Enoyl-CoA hydratase/carnithine racemase	GO:0016052 carbohydrate	gg	t
paaG	CDS	acyl-CoA hydratase	Cytoplasmic	COG1024;Enoyl-CoA hydratase/carnithine racemase	GO:0016052 carbohydrate	gg	o
paaH	CDS	3-hydroxybutyryl-CoA dehydrogenase	Cytoplasmic	COG1250;3-hydroxyacyl-CoA dehydrogenase	GO:0016052 carbohydrate	o	t
paaI	CDS	predicted thioesterase	Cytoplasmic	COG2050;Uncharacterized protein, possibly involved in aromatic compounds catabolism	GO:0016052 carbohydrate catabolism	gg	t
paaJ	CDS	predicted beta-ketoadipyl CoA thiolase	Cytoplasmic	COG0183;Acetyl-CoA acetyltransferase	GO:0016052 carbohydrate	o	t
paaK	CDS	phenylacetyl-CoA ligase	Cytoplasmic	COG1541;Coenzyme F390 synthetase	GO:0016052 carbohydrate		
paaX	CDS	DNA-binding transcriptional regulator, aryl-CoA responsive	Cytoplasmic	COG3327;Phenylacetic acid-responsive transcriptional repressor	GO:0016052 carbohydrate catabolism	o	o
paaY	CDS	predicted hexapeptide repeat acetyltransferase	Cytoplasmic	COG0663;Carbonic anhydrases/acetyltransferases, isoleucine patch superfamily		gg	t
pabA	CDS	aminodeoxychorismate synthase, subunit II	Cytoplasmic	COG0512;Anthranilate/para-aminobenzoate synthases component II	GO:0046656 folic acid biosynthesis	gg	t
pabB	CDS	aminodeoxychorismate synthase, subunit I	Cytoplasmic	COG0147;Anthranilate/para-aminobenzoate synthases component I	GO:0046656 folic acid biosynthesis	gg	o
pabC	CDS	4-amino-4-deoxychorismate lyase component of para-aminobenzoate synthase multienzyme complex	Cytoplasmic	COG0115;Branched-chain amino acid aminotransferase/4-amino-4-deoxychorismate lyase	GO:0046656 folic acid biosynthesis	g	o
pal	CDS	peptidoglycan-associated outer membrane lipoprotein	Outer Membrane Lipoprotein	COG2885;Outer membrane protein and related peptidoglycan-associated (lipo)proteins		o	tt
panB	CDS	3-methyl-2-oxobutanoate hydroxymethyltransferase	Cytoplasmic	COG0413;Ketopantoate hydroxymethyltransferase	GO:0015937 coenzyme A biosynthesis	gg	t
panC	CDS	pantothenate synthetase	Cytoplasmic	COG0414;Panthothenate synthetase	GO:0015937 coenzyme A biosynthesis	gg	o
panD	CDS	aspartate 1-decarboxylase	Cytoplasmic	COG0853;Aspartate 1-decarboxylase	GO:0015937 coenzyme A biosynthesis	gg	t
panE	CDS	2-dehydropantoate reductase, NADPH-specific	Outer Membrane Lipoprotein	COG1893;Ketopantoate reductase	GO:0006221 pyrimidine nucleotide biosynthesis	o	tt
panF	CDS	pantothenate:sodium symporter	Integral Membrane Protein		GO:0015937 coenzyme A biosynthesis	o	tt
parC	CDS	DNA topoisomerase IV, subunit A	Cytoplasmic	COG0188;Type IIA topoisomerase (DNA gyrase/topo II, topoisomerase IV), A subunit	GO:0006261 DNA dependent DNA replication	o	tt
parE	CDS	DNA topoisomerase IV, subunit B	Cytoplasmic	COG0187;Type IIA topoisomerase (DNA gyrase/topo II, topoisomerase IV), B subunit	GO:0006261 DNA dependent DNA replication	gg	t
pbl	CDS	predicted peptidoglycan-binding enzyme (pseudogene)	Cytoplasmic	COG0741;Soluble lytic murein transglycosylase and related regulatory proteins (some contain LysM/invasin domains)			
pbpC	CDS	fused transglycosylase -!- transpeptidase	Periplasmic	COG4953;Membrane carboxypeptidase/penicillin-binding protein PbpC	GO:0009252 peptidoglycan biosynthesis		
pbpG	CDS	D-alanyl-D-alanine endopeptidase	Periplasmic	COG1686;D-alanyl-D-alanine carboxypeptidase	GO:0009252 peptidoglycan biosynthesis -!- GO:0042493 response to drug		
pck	CDS	phosphoenolpyruvate carboxykinase	Cytoplasmic	COG1866;Phosphoenolpyruvate carboxykinase (ATP)	GO:0006094 gluconeogenesis	gg	t
pcm	CDS	L-isoaspartate protein carboxylmethyltransferase type II	Cytoplasmic	COG2518;Protein-L-isoaspartate carboxylmethyltransferase	GO:0006464 protein modification	gg	t
pcnB	CDS	poly(A) polymerase I	Cytoplasmic	COG0617;tRNA nucleotidyltransferase/poly(A) polymerase	GO:0009451 RNA modification		
pdhR	CDS	DNA-binding transcriptional dual regulator	Cytoplasmic	COG2186;Transcriptional regulators	GO:0006096 glycolysis -!- GO:0006350 transcription	o	tt

pdxA	CDS	4-hydroxy-L-threonine phosphate dehydrogenase, NAD-dependent	Cytoplasmic	COG1995;Pyridoxal phosphate biosynthesis protein	GO:0008615 pyridoxine biosynthesis	gg	o
pdxB	CDS	erythronate-4-phosphate dehydrogenase	Cytoplasmic	COG0111;Phosphoglycerate dehydrogenase and related dehydrogenases	GO:0008615 pyridoxine biosynthesis	gg	t
pdxH	CDS	pyridoxine 5'-phosphate oxidase	Cytoplasmic	COG0259;Pyridoxamine-phosphate oxidase	GO:0008615 pyridoxine biosynthesis -!- GO:0009443 pyridoxal 5'-phosphate salvage	gg	t
pdxJ	CDS	pyridoxine 5'-phosphate synthase	Cytoplasmic	COG0854;Pyridoxal phosphate biosynthesis protein	GO:0008615 pyridoxine biosynthesis	gg	o
pdxK	CDS	pyridoxal-pyridoxamine kinase/hydroxymethylpyrimidine kinase	Cytoplasmic	COG2240;Pyridoxal/pyridoxine/pyridoxamine kinase	GO:0009443 pyridoxal 5'-phosphate salvage	gg	tt
pdxY	CDS	pyridoxal kinase 2/pyridoxine kinase	Cytoplasmic	COG2240;Pyridoxal/pyridoxine/pyridoxamine kinase	GO:0009443 pyridoxal 5'-phosphate salvage	gg	t
pepA	CDS	aminopeptidase A, a cyteinylglycinase	Cytoplasmic	COG0260;Leucyl aminopeptidase	GO:0006310 DNA recombination -!- GO:0006350 transcription	gg	t
pepB	CDS	aminopeptidase B	Cytoplasmic			gg	tt
pepD	CDS	aminoacyl-histidine dipeptidase (peptidase D)	Cytoplasmic	COG2195;Di- and tripeptidases		gg	o
pepE	CDS	(alpha)-aspartyl dipeptidase	Cytoplasmic	COG3340;Peptidase E		gg	t
pepN	CDS	aminopeptidase N	Cytoplasmic	COG0308;Aminopeptidase N		gg	tt
pepP	CDS	proline aminopeptidase P II	Cytoplasmic	COG0006;Xaa-Pro aminopeptidase		o	tt
pepQ	CDS	proline dipeptidase	Cytoplasmic	COG0006;Xaa-Pro aminopeptidase		gg	t
pepT	CDS	peptidase T	Cytoplasmic	COG2195;Di- and tripeptidases		gg	t
perR	CDS	CP4-6 prophage; predicted DNA-binding transcriptional regulator	Cytoplasmic	COG0583;Transcriptional regulator		gg	t
pfkA	CDS	6-phosphofructokinase I	Cytoplasmic	COG0205;6-phosphofructokinase	GO:0016052 carbohydrate	g	o
pfkB	CDS	6-phosphofructokinase II	Cytoplasmic		GO:0016052 carbohydrate		
pflA	CDS	pyruvate formate lyase activating enzyme 1	Cytoplasmic	COG1180;Pyruvate-formate lyase-activating enzyme	GO:0009061 anaerobic respiration -!- GO:0006464 protein modification	gg	o
pflB	CDS	pyruvate formate lyase I	Cytoplasmic	COG1882;Pyruvate-formate lyase	GO:0009061 anaerobic respiration -!- GO:0009063 amino acid catabolism	o	tt
pflC	CDS	pyruvate formate lyase II activase	Cytoplasmic		GO:0009061 anaerobic respiration -!- GO:0006464 protein modification	gg	t
pflD	CDS	predicted formate acetyltransferase 2 (pyruvate formate lyase II)	Cytoplasmic	COG1882;Pyruvate-formate lyase	GO:0009061 anaerobic respiration	o	tt
pfs	CDS	5'-methylthioadenosine/S-adenosylhomocysteine nucleosidase	Cytoplasmic	COG0775;Nucleoside phosphorylase	GO:0015949 nucleobase, nucleoside and nucleotide interconversion	gg	o
pgi	CDS	glucosephosphate isomerase	Cytoplasmic	COG0166;Glucose-6-phosphate isomerase	GO:0006096 glycolysis -!- GO:0006094 gluconeogenesis	gg	o
pgk	CDS	phosphoglycerate kinase	Cytoplasmic	COG0126;3-phosphoglycerate kinase	GO:0006096 glycolysis -!- GO:0006094 gluconeogenesis	gg	o
pgm	CDS	phosphoglucomutase	Cytoplasmic	COG0033;Phosphoglucomutase	GO:0016052 carbohydrate catabolism -!- GO:0006006 glucose	gg	tt
pgpA	CDS	phosphatidylglycerophosphatase A	Integral Membrane Protein	COG1267;Phosphatidylglycerophosphatase A and related proteins	GO:0008654 phospholipid biosynthesis	o	tt
pgpB	CDS	phosphatidylglycerophosphate phosphatase B	Integral Membrane Protein	COG0671;Membrane-associated phospholipid phosphatase	GO:0008654 phospholipid biosynthesis	o	tt
pgsA	CDS	phosphatidylglycerophosphate synthetase	Integral Membrane Protein	COG0558;Phosphatidylglycerophosphate synthase	GO:0008654 phospholipid biosynthesis	o	tt
pheA	CDS	fused chorismate mutase P -!- prephenate dehydratase	Cytoplasmic	COG1605;Chorismate mutase -!- COG0077;Prephenate dehydratase	GO:0009094 L-phenylalanine biosynthesis -!- GO:0006571 tyrosine biosynthesis	gg	o
pheL	CDS	pheA gene leader peptide	Cytoplasmic		GO:0009094 L-phenylalanine biosynthesis	gg	o
pheM	CDS	phenylalanyl-tRNA synthetase operon leader peptide	Cytoplasmic		GO:0006418 amino acid activation	o	o
pheP	CDS	phenylalanine transporter	Integral Membrane Protein	COG1113;Gamma-aminobutyrate permease and related permeases	GO:0009094 L-phenylalanine biosynthesis -!- GO:0009063 amino acid catabolism	o	tt
pheS	CDS	phenylalanine tRNA synthetase, alpha subunit	Cytoplasmic	COG0016;Phenylalanyl-tRNA synthetase alpha subunit	GO:0006418 amino acid activation		
pheT	CDS	phenylalanine tRNA synthetase, beta subunit	Cytoplasmic	COG0073;EMAP domain -!- COG0072;Phenylalanyl-tRNA synthetase beta subunit	GO:0006418 amino acid activation	o	tt
pheU	tRNA	tRNA-Phe(GAA) (Phenylalanine tRNA)					
pheV	tRNA	tRNA-Phe(GAA) (Phenylalanine tRNA)					
phnA	CDS	predicted phosphonate metabolizing protein	Cytoplasmic	COG2824;Uncharacterized Zn-ribbon-containing protein involved in phosphonate metabolism		gg	o
phnB	CDS	conserved protein	Cytoplasmic	COG2764;Uncharacterized protein conserved in bacteria		gg	o
phnC	CDS	phosphonate/organophosphate ester transporter subunit -!- ATP-binding component of ABC superfamily	Cytoplasmic	COG3638;ABC-type phosphate/phosphonate transport system, ATPase component	GO:0006793 phosphorus metabolism	o	tt
phnD	CDS	phosphonate/organophosphate ester transporter subunit -!- periplasmic binding component of ABC superfamily	Periplasmic	COG3221;ABC-type phosphate/phosphonate transport system, periplasmic component	GO:0006793 phosphorus metabolism	o	tt
phnE	CDS	phosphonate/organophosphate ester transporter (pseudogene)	Integral Membrane Protein	COG3639;ABC-type phosphate/phosphonate transport system, permease component		o	tt
phnE	CDS	phosphonate/organophosphate ester transporter subunit -!- membrane component of ABC superfamily	Integral Membrane Protein	COG3639;ABC-type phosphate/phosphonate transport system, permease component	GO:0006793 phosphorus metabolism	o	tt
phnF	CDS	transcriptional regulator of phosphonate uptake and biodegradation	Cytoplasmic	COG2188;Transcriptional regulators	GO:0006793 phosphorus metabolism -!- GO:0006350 transcription	gg	o
phnG	CDS	carbon-phosphorus lyase complex subunit	Cytoplasmic	COG3624;Uncharacterized enzyme of phosphonate metabolism	GO:0006793 phosphorus metabolism	gg	o
phnH	CDS	carbon-phosphorus lyase complex subunit	Cytoplasmic	COG3625;Uncharacterized enzyme of phosphonate metabolism	GO:0006793 phosphorus metabolism	gg	t
phnI	CDS	carbon-phosphorus lyase complex subunit	Cytoplasmic	COG3626;Uncharacterized enzyme of phosphonate metabolism	GO:0006793 phosphorus metabolism	gg	tt
phnJ	CDS	carbon-phosphorus lyase complex subunit	Cytoplasmic	COG3627;Uncharacterized enzyme of phosphonate metabolism		gg	t
phnK	CDS	carbon-phosphorus lyase complex subunit	Cytoplasmic		GO:0006793 phosphorus metabolism		
phnL	CDS	carbon-phosphorus lyase complex subunit	Cytoplasmic	COG4778;ABC-type phosphonate transport system, ATPase component	GO:0006793 phosphorus metabolism	gg	t
phnM	CDS	carbon-phosphorus lyase complex subunit	Cytoplasmic	COG3454;Metal-dependent hydrolase involved in phosphonate metabolism	GO:0006793 phosphorus metabolism	gg	t
phnN	CDS	ribose 1,5-bisphosphokinase	Cytoplasmic	COG3709;Uncharacterized component of phosphonate metabolism	GO:0006793 phosphorus metabolism	gg	tt
phnO	CDS	predicted acyltransferase with acyl-CoA N-acyltransferase domain	Cytoplasmic	COG0454;Histone acetyltransferase HPA2 and related acetyltransferases		gg	o
phnP	CDS	carbon-phosphorus lyase complex accessory protein	Periplasmic	COG1235;Metal-dependent hydrolases of the beta-lactamase superfamily I	GO:0006793 phosphorus metabolism	gg	o
phoA	CDS	alkaline phosphatase	Periplasmic		GO:0006793 phosphorus metabolism	o	tt
phoB	CDS	DNA-binding response regulator in two-component regulatory system with PhoR (or CreC)	Cytoplasmic	COG0745;Response regulators consisting of a CheY-like receiver domain and a winged-helix DNA-binding domain	GO:0006793 phosphorus metabolism -!- GO:0006350 transcription	gg	o
phoE	CDS	outer membrane phosphoporin protein E	Outer Membrane B-barrel protein	COG3203;Outer membrane protein (porin)		o	tt
phoH	CDS	conserved protein with nucleoside triphosphate hydrolase domain	Cytoplasmic	COG1702;Phosphate starvation-inducible protein PhoH, predicted ATPase	GO:0006793 phosphorus metabolism -!- GO:0042594 response to	gg	t
phoP	CDS	DNA-binding response regulator in two-component regulatory system with PhoQ	Cytoplasmic	COG0745;Response regulators consisting of a CheY-like receiver domain and a winged-helix DNA-binding domain	GO:0006350 transcription	gg	o
phoQ	CDS	sensory histidine kinase in two-compoent regulatory system with PhoP	Integral Membrane Protein	COG0642;Signal transduction histidine kinase	GO:0006464 protein modification	o	tt
phoR	CDS	sensory histidine kinase in two-component regulatory system with PhoB	Membrane Anchored	COG0642;Signal transduction histidine kinase	GO:0006793 phosphorus metabolism -!- GO:0006464 protein modification	o	t
phoU	CDS	DNA-binding transcriptional regulator	Cytoplasmic	COG0704;Phosphate uptake regulator	GO:0006793 phosphorus metabolism	gg	o
php	CDS	predicted hydrolase	Cytoplasmic	COG1735;Predicted metal-dependent hydrolase with the TIM-barrel fold		gg	t
phr	CDS	deoxyribodipyrimidine photolyase, FAD-binding	Cytoplasmic	COG0415;Deoxyribodipyrimidine photolyase	GO:0006281 DNA repair	o	tt
pin	CDS	e14 prophage; site-specific DNA recombinase	Cytoplasmic	COG1961;Site-specific recombinases, DNA invertase Pin homologs		o	tt
pinH	CDS	predicted invertase fragment (pseudogene)	Cytoplasmic				
pinQ	CDS	Qin prophage; predicted site-specific recombinase	Cytoplasmic	COG1961;Site-specific recombinases, DNA invertase Pin homologs		o	tt
pinR	CDS	Rac prophage; predicted site-specific recombinase	Cytoplasmic	COG1961;Site-specific recombinases, DNA invertase Pin homologs		o	tt
pioO	CDS	part of gsp divergon involved in type II protein secretion	Membrane Anchored		GO:0006261 DNA dependent DNA replication	o	tt
pitA	CDS	phosphate transporter, low-affinity	Integral Membrane Protein	COG0306;Phosphate/sulphate permeases	GO:0006793 phosphorus metabolism	o	tt
pitB	CDS	phosphate transporter	Integral Membrane Protein	COG0306;Phosphate/sulphate permeases	GO:0006793 phosphorus metabolism	o	tt
pldA	CDS	outer membrane phospholipase A	Outer Membrane B-barrel protein	COG2829;Outer membrane phospholipase A	GO:0006793 phosphorus metabolism	o	tt
pldB	CDS	lysophospholipase L(2)	Cytoplasmic		GO:0006793 phosphorus metabolism		
plsB	CDS	glycerol-3-phosphate O-acyltransferase	Cytoplasmic		GO:0008654 phospholipid biosynthesis	o	tt
plsC	CDS	1-acyl-sn-glycerol-3-phosphate acyltransferase	Membrane Anchored	COG0204;1-acyl-sn-glycerol-3-phosphate acyltransferase	GO:0008654 phospholipid biosynthesis	o	tt
plsX	CDS	fatty acid/phospholipid synthesis protein	Cytoplasmic	COG0416;Fatty acid/phospholipid biosynthesis enzyme	GO:0008654 phospholipid biosynthesis		
pmbA	CDS	predicted peptidase required for the maturation and secretion of the antibiotic peptide MccB17	Cytoplasmic	COG0312;Predicted Zn-dependent proteases and their inactivated homologs	GO:0006457 protein folding	gg	tt
pmrD	CDS	polymyxin resistance protein B	Cytoplasmic		GO:0042493 response to drug	gg	o
pncA	CDS	nicotinamidase/pyrazinamidase	Cytoplasmic		GO:0009435 nicotinamide adenine dinucleotide biosynthesis	gg	o
pncB	CDS	nicotinate phosphoribosyltransferase	Cytoplasmic	COG1488;Nicotinic acid phosphoribosyltransferase	GO:0009435 nicotinamide adenine dinucleotide biosynthesis	gg	tt
pnp	CDS	polynucleotide phosphorylase/polyadenylase	Cytoplasmic		GO:0006401 RNA catabolism -!- GO:0006401 RNA catabolism		
pntA	CDS	pyridine nucleotide transhydrogenase, alpha subunit	Integral Membrane Protein	COG3288;NAD/NADP transhydrogenase alpha subunit		o	tt
pntB	CDS	pyridine nucleotide transhydrogenase, beta subunit	Integral Membrane Protein	COG1282;NAD/NADP transhydrogenase beta subunit		o	tt
pnuC	CDS	predicted nicotinamide mononucleotide transporter	Integral Membrane Protein	COG3201;Nicotinamide mononucleotide transporter	GO:0015949 nucleobase, nucleoside and nucleotide interconversion	o	tt
polA	CDS	fused DNA polymerase I 5'->3' exonuclease -!- 3'->5' polymerase -!- 3'->5' exonuclease	Cytoplasmic	COG0258;5'-3' exonuclease (including N-terminal domain of PolI) -!- COG0749;DNA polymerase I - 3'-5' exonuclease and polymerase domains	GO:0006261 DNA dependent DNA replication	o	tt
polB	CDS	DNA polymerase II	Cytoplasmic	COG0417;DNA polymerase elongation subunit (family B)	GO:0006261 DNA dependent DNA replication -!- GO:0006281 DNA repair -!- GO:0009314 response to radiation -!- GO:0009432 SOS	g	tt
potA	CDS	polyamine transporter subunit -!- ATP-binding component of ABC superfamily	Cytoplasmic	COG3842;ABC-type spermidine/putrescine transport systems, ATPase components	GO:0006596 polyamine biosynthesis -!- GO:0009310 amine catabolism	gg	t

potB	CDS	polyamine transporter subunit -!- membrane component of ABC superfamily	Integral Membrane Protein	COG1176;ABC-type spermidine/putrescine transport system, permease component I	GO:0006596 polyamine biosynthesis -!- GO:0009310 amine catabolism	o	tt
potC	CDS	polyamine transporter subunit -!- membrane component of ABC superfamily	Integral Membrane Protein	COG1177;ABC-type spermidine/putrescine transport system, permease component II	GO:0006596 polyamine biosynthesis -!- GO:0009310 amine catabolism	o	tt
potD	CDS	polyamine transporter subunit -!- periplasmic-binding component of ABC superfamily	Periplasmic	COG0687;Spermidine/putrescine-binding periplasmic protein	GO:0006596 polyamine biosynthesis -!- GO:0009310 amine catabolism	o	tt
potE	CDS	putrescine/proton symporter -!- putrescine/ornithine antiporter	Integral Membrane Protein	COG0531;Amino acid transporters	GO:0009310 amine catabolism -!- GO:0006596 polyamine biosynthesis -!- GO:0006970 response to osmotic stress	o	tt
potF	CDS	putrescine transporter subunit -!- periplasmic-binding component of ABC superfamily	Periplasmic	COG0687;Spermidine/putrescine-binding periplasmic protein	GO:0006596 polyamine biosynthesis -!- GO:0009310 amine catabolism -!- GO:0015847 putrescine transport	o	tt
potG	CDS	putrescine transporter subunit -!- ATP-binding component of ABC superfamily	Cytoplasmic		GO:0006596 polyamine biosynthesis -!- GO:0009310 amine catabolism -!- GO:0015847 putrescine transport		
potH	CDS	putrescine transporter subunit -!- membrane component of ABC superfamily	Integral Membrane Protein	COG1176;ABC-type spermidine/putrescine transport system, permease component I	GO:0006596 polyamine biosynthesis -!- GO:0009310 amine catabolism -!- GO:0015847 putrescine transport	o	tt
potI	CDS	putrescine transporter subunit -!- membrane component of ABC superfamily	Integral Membrane Protein	COG1177;ABC-type spermidine/putrescine transport system, permease component II	GO:0006596 polyamine biosynthesis -!- GO:0009310 amine catabolism -!- GO:0015847 putrescine transport	o	tt
poxA	CDS	predicted lysyl-tRNA synthetase	Cytoplasmic	COG2269;Truncated, possibly inactive, lysyl-tRNA synthetase (class II)	GO:0006418 amino acid activation	o	tt
poxB	CDS	pyruvate dehydrogenase (pyruvate oxidase), thiamin-dependent, FAD-binding	Cytoplasmic	COG0028;Thiamine pyrophosphate-requiring enzymes [acetolactate synthase, pyruvate dehydrogenase (cytochrome), glyoxylate carboligase, phosphonopyruvate decarboxylase]	GO:0016052 carbohydrate catabolism -!- GO:0042867 pyruvate catabolism	gg	t
ppa	CDS	inorganic pyrophosphatase	Cytoplasmic	COG0221;Inorganic pyrophosphatase	GO:0006793 phosphorus metabolism	gg	o
ppc	CDS	phosphoenolpyruvate carboxylase	Cytoplasmic	COG2352;Phosphoenolpyruvate carboxylase	GO:0006113 fermentation -!- GO:0006099 tricarboxylic acid cycle	gg	tt
ppdA	CDS	conserved protein	Periplasmic	COG2165;Type II secretory pathway, pseudopilin PulG	GO:0009101 glycoprotein biosynthesis	o	tt
ppdB	CDS	conserved protein	Membrane Anchored	COG4795;Type II secretory pathway, component PulJ	GO:0009101 glycoprotein biosynthesis		
ppdC	CDS	predicted protein	Membrane Anchored	COG4967;Tfp pilus assembly protein PilV	GO:0009101 glycoprotein biosynthesis	o	tt
ppdD	CDS	predicted major pilin subunit	Membrane Anchored	COG4969;Tfp pilus assembly protein, major pilin PilA		o	tt
pphA	CDS	serine/threonine-specific protein phosphatase 1	Cytoplasmic		GO:0006464 protein modification -!- GO:0009266 response to	g	t
pphB	CDS	serine/threonine-specific protein phosphatase 2	Cytoplasmic	COG0639;Diadenosine tetraphosphatase and related serine/threonine protein phosphatases	GO:0006464 protein modification	g	o
ppiA	CDS	peptidyl-prolyl cis-trans isomerase A (rotamase A)	Periplasmic	COG0652;Peptidyl-prolyl cis-trans isomerase (rotamase) - cyclophilin family	GO:0006457 protein folding	o	tt
ppiB	CDS	peptidyl-prolyl cis-trans isomerase B (rotamase B)	Cytoplasmic	COG0652;Peptidyl-prolyl cis-trans isomerase (rotamase) - cyclophilin family	GO:0006457 protein folding	gg	t
ppiC	CDS	peptidyl-prolyl cis-trans isomerase C (rotamase C)	Cytoplasmic	COG0760;Parvulin-like peptidyl-prolyl isomerase	GO:0006457 protein folding	gg	t
ppiD	CDS	peptidyl-prolyl cis-trans isomerase (rotamase D)	Membrane Anchored	COG0760;Parvulin-like peptidyl-prolyl isomerase	GO:0006457 protein folding	o	tt
ppk	CDS	polyphosphate kinase, component of RNA degradosome	Cytoplasmic	COG0855;Polyphosphate kinase	GO:0006793 phosphorus metabolism	o	tt
pppA	CDS	bifunctional prepilin leader peptidase -!- methylase	Integral Membrane Protein			g	tt
pps	CDS	phosphoenolpyruvate synthase	Cytoplasmic	COG0574;Phosphoenolpyruvate synthase/pyruvate phosphate dikinase	GO:0006094 gluconeogenesis	o	tt
ppx	CDS	exopolyphosphatase	Cytoplasmic	COG0248;Exopolyphosphatase	GO:0006793 phosphorus metabolism	gg	tt
pqiA	CDS	paraquat-inducible membrane protein A	Integral Membrane Protein	COG2995;Uncharacterized paraquat-inducible protein A		o	tt
pqiB	CDS	paraquat-inducible protein B	Membrane Anchored	COG3008;Paraquat-inducible protein B		o	tt
pqqL	CDS	predicted peptidase	Cytoplasmic	COG0612;Predicted Zn-dependent peptidases		gg	t
prc	CDS	carboxy-terminal protease for penicillin-binding protein 3	Periplasmic	COG0793;Periplasmic protease	GO:0009252 peptidoglycan biosynthesis -!- GO:0006457 protein folding -!- GO:0042493 response to drug	o	o
prfA	CDS	peptide chain release factor RF-1	Cytoplasmic	COG0216;Protein chain release factor A	GO:0006412 protein biosynthesis	o	tt
prfB	CDS	peptide chain release factor RF-2	Cytoplasmic	COG1186;Protein chain release factor B	GO:0006412 protein biosynthesis		
prfC	CDS	peptide chain release factor RF-3	Cytoplasmic	COG4108;Peptide chain release factor RF-3	GO:0006412 protein biosynthesis		
prfH	CDS	predicted peptide chain release factor	Cytoplasmic	COG1186;Protein chain release factor B	GO:0006412 protein biosynthesis	g	o
priA	CDS	Primosome factor n' (replication factor Y)	Cytoplasmic	COG1198;Primosomal protein N' (replication factor Y) - superfamily II helicase	GO:0006261 DNA dependent DNA replication	o	tt
priB	CDS	primosomal protein N	Cytoplasmic	COG2965;Primosomal replication protein N	GO:0006261 DNA dependent DNA replication	o	tt
priC	CDS	primosomal replication protein N''	Cytoplasmic	COG3923;Primosomal replication protein N''	GO:0006261 DNA dependent DNA replication	gg	tt
prkB	CDS	predicted phosphoribulokinase	Periplasmic	COG3954;Phosphoribulokinase	GO:0006006 glucose metabolism	gg	t
prlC	CDS	oligopeptidase A	Cytoplasmic	COG0339;Zn-dependent oligopeptidases		gg	o
prmA	CDS	methylase for 50S ribosomal subunit protein L11	Cytoplasmic	COG2264;Ribosomal protein L11 methylase	GO:0009451 RNA modification	gg	tt
prmB	CDS	N5-glutamine methyltransferase	Cytoplasmic				
prmC	CDS	N5-glutamine methyltransferase, modifies release factors RF-1 and RF-2	Cytoplasmic	COG2890;Methylase of polypeptide chain release factors		gg	t
proA	CDS	gamma-glutamylphosphate reductase	Cytoplasmic	COG0014;Gamma-glutamyl phosphate reductase	GO:0006561 proline biosynthesis	g	t
proB	CDS	gamma-glutamate kinase	Cytoplasmic	COG0263;Glutamate 5-kinase	GO:0006561 proline biosynthesis	g	t
proC	CDS	pyrroline-5-carboxylate reductase, NAD(P)-binding	Cytoplasmic	COG0345;Pyrroline-5-carboxylate reductase	GO:0006561 proline biosynthesis	gg	o
proK	tRNA	tRNA-Pro(CGG) (Proline tRNA1)					
proL	tRNA	tRNA-Pro(GGG) (Proline tRNA2)					
proM	tRNA	tRNA-Pro(UGG) (Proline tRNA3)					
proP	CDS	proline/glycine betaine transporter	Integral Membrane Protein	COG0477;Permeases of the major facilitator superfamily	GO:0006561 proline biosynthesis -!- GO:0006970 response to osmotic stress	o	tt
proQ	CDS	predicted structural transport element	Cytoplasmic		GO:0006970 response to osmotic stress		
proS	CDS	prolyl-tRNA synthetase	Cytoplasmic	COG0442;Prolyl-tRNA synthetase	GO:0006418 amino acid activation	o	tt
proV	CDS	glycine betaine transporter subunit -!- ATP-binding compoent of ABC superfamily	Cytoplasmic	COG4175;ABC-type proline/glycine betaine transport system, ATPase component	GO:0006561 proline biosynthesis	gg	o
proW	CDS	glycine betaine transporter subunit -!- membrane component of ABC superfamily	Integral Membrane Protein	COG4176;ABC-type proline/glycine betaine transport system, permease component	GO:0006561 proline biosynthesis	o	tt
proX	CDS	glycine betaine transporter subunit -!- periplasmic-binding component of ABC superfamily	Periplasmic	COG2113;ABC-type proline/glycine betaine transport systems, periplasmic components	GO:0016052 carbohydrate catabolism -!- GO:0006561 proline biosynthesis -!- GO:0006545 glycine biosynthesis -!- GO:0006578 betaine biosynthesis -!- GO:0006970 response to osmotic stress	o	tt
proY	CDS	predicted cryptic proline transporter	Integral Membrane Protein	COG1113;Gamma-aminobutyrate permease and related permeases	GO:0009063 amino acid catabolism -!- GO:0006526 arginine biosynthesis		
prpB	CDS	2-methylisocitrate lyase	Cytoplasmic	COG2513;PEP phosphonomutase and related enzymes	GO:0019395 fatty acid oxidation	gg	t
prpC	CDS	2-methylcitrate synthase	Cytoplasmic	COG0372;Citrate synthase	GO:0019395 fatty acid oxidation	gg	t
prpD	CDS	2-methylcitrate dehydratase	Cytoplasmic	COG2079;Uncharacterized protein involved in propionate catabolism	GO:0016052 carbohydrate	g	o
prpE	CDS	predicted propionyl-CoA synthetase with ATPase domain	Cytoplasmic	COG0365;Acyl-coenzyme A synthetases/AMP-(fatty) acid ligases	GO:0019395 fatty acid oxidation	gg	t
prpR	CDS	DNA-binding transcriptional regulator	Cytoplasmic	COG1221;Transcriptional regulators containing an AAA-type ATPase domain and a DNA-binding domain	GO:0019395 fatty acid oxidation -!- GO:0006350 transcription	g	tt
prsA	CDS	phosphoribosylpyrophosphate synthase	Cytoplasmic	COG0462;Phosphoribosylpyrophosphate synthetase	GO:0009152 purine ribonucleotide biosynthesis	gg	t
psd	CDS	phosphatidylserine decarboxylase	Cytoplasmic	COG0688;Phosphatidylserine decarboxylase	GO:0008654 phospholipid biosynthesis	o	tt
psiF	CDS	conserved protein	Periplasmic		GO:0006793 phosphorus metabolism -!- GO:0042594 response to		
pspA	CDS	regulatory protein for phage-shock-protein operon	Cytoplasmic	COG1842;Phage shock protein A (IM30), suppresses sigma54-dependent transcription	GO:0006350 transcription	gg	o
pspB	CDS	transcriptional regulator of psp operon	Membrane Anchored		GO:0006350 transcription	gg	tt
pspC	CDS	transcriptional activator	Membrane Anchored	COG1983;Putative stress-responsive transcriptional regulator	GO:0006350 transcription	gg	tt
pspD	CDS	peripheral inner membrane phage-shock protein	Cytoplasmic			g	tt
pspE	CDS	thiosulfate:cyanide sulfurtransferase (rhodanese)	Periplasmic	COG0607;Rhodanese-related sulfurtransferase		g	tt
pspF	CDS	DNA-binding transcriptional activator	Cytoplasmic		GO:0006350 transcription	gg	tt
pssA	CDS	phosphatidylserine synthase (CDP-diacylglycerol-serine O-phosphatidyltransferase)	Cytoplasmic		GO:0008654 phospholipid biosynthesis	gg	tt
pstA	CDS	phosphate transporter subunit -!- membrane component of ABC superfamily	Integral Membrane Protein	COG0581;ABC-type phosphate transport system, permease component	GO:0006793 phosphorus metabolism	o	tt
pstB	CDS	phosphate transporter subunit -!- ATP-binding component of ABC superfamily	Cytoplasmic	COG1117;ABC-type phosphate transport system, ATPase component	GO:0006793 phosphorus metabolism	g	t
pstC	CDS	phosphate transporter subunit -!- membrane component of ABC superfamily	Integral Membrane Protein	COG0573;ABC-type phosphate transport system, permease component	GO:0006793 phosphorus metabolism	o	tt
pstS	CDS	phosphate transporter subunit -!- periplasmic-binding component of ABC superfamily	Periplasmic	COG0226;ABC-type phosphate transport system, periplasmic component	GO:0006793 phosphorus metabolism	o	tt
pta	CDS	phosphate acetyltransferase	Cytoplasmic	COG0857;BioD-like N-terminal domain of phosphotransacetylase -!- COG0280;Phosphotransacetylase	GO:0016052 carbohydrate catabolism -!- GO:0009063 amino acid catabolism -!- GO:0042867 pyruvate catabolism -!- GO:0045733 acetate catabolism	gg	t
pth	CDS	peptidyl-tRNA hydrolase	Cytoplasmic	COG0193;Peptidyl-tRNA hydrolase	GO:0006418 amino acid activation	gg	t
ptr	CDS	protease III	Periplasmic	COG1025;Secreted/periplasmic Zn-dependent peptidases, insulinase-like		o	tt
ptrB	CDS	protease II	Cytoplasmic	COG1770;Protease II		o	tt
ptsA	CDS	fused predicted PTS enzymes Hpr component -!- enzyme I component -!- enzyme IIA component	Cytoplasmic	COG1080;Phosphoenolpyruvate-protein kinase (PTS system EI component in bacteria) -!- COG1762;Phosphotransferase system mannitol/fructose-specific IIA domain (Ntr-type)	GO:0016052 carbohydrate catabolism		
ptsG	CDS	fused glucose-specific PTS enzyme IIBC components	Integral Membrane Protein	COG1263;Phosphotransferase system IIC components, glucose/maltose/N-acetylglucosamine-specific -!- COG1264;Phosphotransferase system IIB components	GO:0016052 carbohydrate catabolism -!- GO:0006464 protein modification	o	tt

ptsH	CDS	phosphohistidinoprotein-hexose phosphotransferase component of PTS system (Hpr)	Cytoplasmic	COG1925;Phosphotransferase system, HPr-related proteins	GO:0016052 carbohydrate catabolism	gg	o
ptsI	CDS	PEP-protein phosphotransferase of PTS system (enzyme I)	Cytoplasmic	COG1080;Phosphoenolpyruvate-protein kinase (PTS system EI component in bacteria)	GO:0016052 carbohydrate catabolism	gg	t
ptsN	CDS	sugar-specific enzyme IIA component of PTS	Cytoplasmic	COG1762;Phosphotransferase system mannitol/fructose-specific IIA domain (Ntr-type)		gg	o
ptsP	CDS	fused PEP-protein phosphotransferase (enzyme I) of PTS system	Cytoplasmic	COG3605;Signal transduction protein containing GAF and PtsI domains	GO:0016052 carbohydrate catabolism	o	t
purA	CDS	adenylosuccinate synthetase	Cytoplasmic	COG0104;Adenylosuccinate synthase	GO:0006164 purine nucleotide biosynthesis -!- GO:0015949 nucleobase, nucleoside and nucleotide interconversion	gg	o
purB	CDS	adenylosuccinate lyase	Cytoplasmic	COG0015;Adenylosuccinate lyase	GO:0006164 purine nucleotide biosynthesis	gg	t
purC	CDS	phosphoribosylaminoimidazole-succinocarboxamide synthetase	Cytoplasmic	COG0152;Phosphoribosylaminoimidazolesuccinocarboxa mide (SAICAR) synthase	GO:0006164 purine nucleotide biosynthesis	gg	o
purD	CDS	phosphoribosylglycinamide synthetase phosphoribosylamine-glycine ligase	Cytoplasmic	COG0151;Phosphoribosylamine-glycine ligase	GO:0006164 purine nucleotide biosynthesis	gg	tt
purE	CDS	N5-carboxyaminoimidazole ribonucleotide mutase	Cytoplasmic	COG0041;Phosphoribosylcarboxyaminoimidazole (NCAIR) mutase	GO:0006164 purine nucleotide biosynthesis	gg	o
purF	CDS	amidophosphoribosyltransferase	Cytoplasmic	COG0034;Glutamine phosphoribosylpyrophosphate amidotransferase	GO:0006164 purine nucleotide biosynthesis	gg	t
purH	CDS	fused IMP cyclohydrolase -!- phosphoribosylaminoimidazolecarboxamide formyltransferase	Cytoplasmic	COG0138;AICAR transformylase/IMP cyclohydrolase PurH (only IMP cyclohydrolase domain in Aful)	GO:0006164 purine nucleotide biosynthesis -!- GO:0015949 nucleobase, nucleoside and nucleotide interconversion	gg	t
purK	CDS	N5-carboxyaminoimidazole ribonucleotide synthase	Cytoplasmic	COG0026;Phosphoribosylaminoimidazole carboxylase (NCAIR synthetase)	GO:0006164 purine nucleotide biosynthesis	gg	t
purL	CDS	phosphoribosylformyl-glycineamide synthetase	Cytoplasmic	COG0046;Phosphoribosylformylglycinamidine (FGAM) synthase, synthetase domain -!- COG0047;Phosphoribosylformylglycinamidine (FGAM) synthase, glutamine amidotransferase domain	GO:0006164 purine nucleotide biosynthesis	o	tt
purM	CDS	phosphoribosylaminoimidazole synthetase	Cytoplasmic	COG0150;Phosphoribosylaminoimidazole (AIR) synthetase	GO:0006164 purine nucleotide biosynthesis	gg	t
purN	CDS	phosphoribosylglycinamide formyltransferase 1	Cytoplasmic	COG0299;Folate-dependent phosphoribosylglycinamide formyltransferase PurN	GO:0006164 purine nucleotide biosynthesis	gg	t
purR	CDS	DNA-binding transcriptional repressor, hypoxanthine-binding	Cytoplasmic	COG1609;Transcriptional regulators	GO:0006164 purine nucleotide biosynthesis -!- GO:0006596 polyamine biosynthesis -!- GO:0000105 histidine biosynthesis -!- GO:0009063 amino acid catabolism -!- GO:0006545 glycine biosynthesis -!- GO:0006807 nitrogen metabolism -!- GO:0009257 10-formyltetr	gg	t
purT	CDS	phosphoribosylglycinamide formyltransferase 2	Cytoplasmic	COG0027;Formate-dependent phosphoribosylglycinamide formyltransferase (GAR transformylase)	GO:0006164 purine nucleotide biosynthesis	gg	t
purU	CDS	formyltetrahydrofolate hydrolase	Cytoplasmic	COG0788;Formyltetrahydrofolate hydrolase	GO:0009257 10-formyltetrahydrofolate biosynthesis -!- GO:0009152 purine ribonucleotide	g	o
putA	CDS	fused DNA-binding transcriptional regulator -!- proline dehydrogenase -!- pyrroline-5-carboxylate dehydrogenase	Cytoplasmic	COG0506;Proline dehydrogenase -!- COG4230;Delta 1-pyrroline-5-carboxylate dehydrogenase	GO:0009063 amino acid catabolism -!- GO:0006350 transcription	g	tt
putP	CDS	proline:sodium symporter	Integral Membrane Protein	COG0591;Na+/proline symporter	GO:0006561 proline biosynthesis	o	tt
puuA	CDS	gamma-Glu-putrescine synthase	Cytoplasmic		GO:0006542 glutamine biosynthesis -!- GO:0006807 nitrogen metabolism		
puuB	CDS	gamma-Glu-putrescine oxidase, FAD/NAD(P)-binding	Cytoplasmic	COG0665;Glycine/D-amino acid oxidases (deaminating)		o	tt
puuC	CDS	gamma-Glu-gamma-aminobutyraldehyde dehydrogenase, NAD(P)H-dependent	Cytoplasmic	COG1012;NAD-dependent aldehyde dehydrogenases	GO:0006113 fermentation	o	tt
puuD	CDS	gamma-Glu-GABA hydrolase	Cytoplasmic		GO:0009310 amine catabolism	gg	o
puuE	CDS	GABA aminotransferase, PLP-dependent	Periplasmic	COG0160;4-aminobutyrate aminotransferase and related aminotransferases	GO:0009310 amine catabolism -!- GO:0008615 pyridoxine biosynthesis	gg	tt
puuP	CDS	putrescine importer	Integral Membrane Protein			gg	tt
puuR	CDS	DNA-binding transcriptional repressor	Cytoplasmic	COG1396;Predicted transcriptional regulators		g	o
pykA	CDS	pyruvate kinase II	Cytoplasmic	COG0469;Pyruvate kinase	GO:0006096 glycolysis -!- GO:0006113 fermentation -!- GO:0009061 anaerobic respiration	gg	t
pykF	CDS	pyruvate kinase I	Cytoplasmic	COG0469;Pyruvate kinase	GO:0006096 glycolysis -!- GO:0006113 fermentation -!- GO:0009061 anaerobic respiration	o	t
pyrB	CDS	aspartate carbamoyltransferase, catalytic subunit	Cytoplasmic	COG0540;Aspartate carbamoyltransferase, catalytic chain		o	t
pyrC	CDS	dihydro-orotase	Cytoplasmic	COG0418;Dihydroorotase	GO:0006221 pyrimidine nucleotide biosynthesis	gg	t
pyrD	CDS	dihydro-orotate oxidase, FMN-linked	Cytoplasmic	COG0167;Dihydroorotate dehydrogenase	GO:0006221 pyrimidine nucleotide biosynthesis	gg	o
pyrE	CDS	orotate phosphoribosyltransferase	Cytoplasmic	COG0461;Orotate phosphoribosyltransferase	GO:0006221 pyrimidine nucleotide biosynthesis	g	t
pyrF	CDS	orotidine-5'-phosphate decarboxylase	Cytoplasmic	COG0284;Orotidine-5'-phosphate decarboxylase	GO:0006221 pyrimidine nucleotide biosynthesis -!- GO:0015949 nucleobase, nucleoside and nucleotide interconversion	gg	o
pyrG	CDS	CTP synthetase	Cytoplasmic	COG0504;CTP synthase (UTP-ammonia lyase)	GO:0015949 nucleobase, nucleoside and nucleotide interconversion	gg	t
pyrH	CDS	uridylate kinase	Cytoplasmic	COG0528;Uridylate kinase	GO:0015949 nucleobase, nucleoside and nucleotide interconversion	gg	o
pyrI	CDS	aspartate carbamoyltransferase, regulatory subunit	Cytoplasmic	COG1781;Aspartate carbamoyltransferase, regulatory subunit	GO:0006221 pyrimidine nucleotide biosynthesis	gg	o
pyrL	CDS	pyrBI operon leader peptide	Cytoplasmic		GO:0006221 pyrimidine nucleotide biosynthesis	o	o
qor	CDS	quinone oxidoreductase, NADPH-dependent	Cytoplasmic	COG0604;NADPH:quinone reductase and related Zn-dependent oxidoreductases		gg	t
qseB	CDS	DNA-binding response regulator in two-component regulatory system with QseC	Cytoplasmic	COG0745;Response regulators consisting of a CheY-like receiver domain and a winged-helix DNA-binding domain	GO:0006355 regulation of transcription, DNA-dependent	gg	t
qseC	CDS	sensory histidine kinase in two-component regulatory system with QseB	Integral Membrane Protein	COG0642;Signal transduction histidine kinase	GO:0006464 protein modification	o	tt
queA	CDS	S-adenosylmethionine:tRNA ribosyltransferase-isomerase	Cytoplasmic	COG0809;S-adenosylmethionine:tRNA-ribosyltransfera se-isomerase (queuine synthetase)	GO:0009451 RNA modification	gg	o
racC	CDS	Rac prophage; predicted protein	Cytoplasmic			g	o
racR	CDS	Rac prophage; predicted DNA-binding transcriptional regulator	Cytoplasmic			g	tt
radA	CDS	predicted repair protein	Cytoplasmic	COG1066;Predicted ATP-dependent serine protease		o	t
rarA	CDS	predicted hydrolase	Cytoplasmic	COG0596;Predicted hydrolases or acyltransferases (alpha/beta hydrolase superfamily)		gg	o
rarD	CDS	predicted chloramphenical resistance permease	Integral Membrane Protein		GO:0042493 response to drug -!- GO:0042892 chloramphenicol transport		
rbbA	CDS	fused ribosome-associated ATPases	Cytoplasmic	COG1131;ABC-type multidrug transport system, ATPase component -!- COG0842;ABC-type multidrug transport system, permease component	GO:0006412 protein biosynthesis		
rbfA	CDS	30s ribosome binding factor	Cytoplasmic	COG0858;Ribosome-binding factor A	GO:0009451 RNA modification	gg	o
rbn	CDS	tRNA processing exoribonuclease BN	Cytoplasmic	COG1295;Predicted membrane protein	GO:0009451 RNA modification	o	tt
rbsA	CDS	fused D-ribose transporter subunits -!- ATP-binding components ABC superfamily	Cytoplasmic	COG1129;ABC-type sugar transport system, ATPase component	GO:0016052 carbohydrate catabolism	gg	o
rbsB	CDS	D-ribose transporter subunit -!- periplasmic-binding compoent of ABC superfamily	Periplasmic	COG1879;ABC-type sugar transport system, periplasmic component	GO:0016052 carbohydrate catabolism	o	tt
rbsC	CDS	D-ribose transporter subunit -!- membrane component of ABC superfamily	Integral Membrane Protein	COG1172;Ribose/xylose/arabinose/galactoside ABC-type transport systems, permease components	GO:0016052 carbohydrate catabolism	o	tt
rbsD	CDS	predicted cytoplasmic sugar-binding protein	Cytoplasmic	COG1869;ABC-type ribose transport system, auxiliary component	GO:0016052 carbohydrate		
rbsK	CDS	ribokinase	Cytoplasmic	COG0524;Sugar kinases, ribokinase family	GO:0016052 carbohydrate	gg	t
rbsR	CDS	DNA-binding transcriptional repressor	Cytoplasmic	COG1609;Transcriptional regulators	GO:0016052 carbohydrate catabolism -!- GO:0006350	g	o
rcsA	CDS	DNA-binding transcriptional co-regulator with RcsB	Cytoplasmic	COG2771;DNA-binding HTH domain-containing proteins	GO:0009242 colanic acid biosynthesis -!- GO:0009248 K antigen biosynthesis -!- GO:0006350 transcription	gg	t
rcsB	CDS	DNA-binding response regulator in two-component regulatory system with RcsC and YojN	Cytoplasmic	COG2197;Response regulator containing a CheY-like receiver domain and an HTH DNA-binding domain	GO:0009242 colanic acid biosynthesis -!- GO:0006350	o	tt
rcsC	CDS	hybrid sensory kinase in two-component regulatory system with RcsB and YojN	Integral Membrane Protein	COG0642;Signal transduction histidine kinase -!- COG0784;FOG: CheY-like receiver	GO:0009242 colanic acid biosynthesis -!- GO:0006464 protein		
rcsC	CDS	hybrid sensory kinase in two-component regulatory system with RcsB and YojN	Integral Membrane Protein				
rcsC	CDS	hybrid sensory kinase in two-component regulatory system with RcsB and YojN	Integral Membrane Protein				
rcsD	CDS	phosphotransfer intermediate protein in two-component regulatory system with RcsBC	Membrane Anchored	COG0642;Signal transduction histidine kinase -!- COG2198;FOG: HPt domain	GO:0006464 protein modification	o	tt
rcsF	CDS	predicted outer membrane protein, signal	Outer Membrane Lipoprotein		GO:0009242 colanic acid biosynthesis	gg	o
rdgC	CDS	DNA-binding protein, non-specific	Cytoplasmic	COG2974;DNA recombination-dependent growth factor C	GO:0006261 DNA dependent DNA replication	o	tt
rdlA	misc_RNA	regulatory, antisense RNA					
rdlB	misc_RNA	regulatory, antisense sRNA					
rdlC	misc_RNA	regulatory, antisense sRNA					

rdlD	misc_RNA	regulatory, antisense sRNA					
recA	CDS	DNA strand exchange and recombination protein with protease and nuclease activity	Cytoplasmic	COG0468;RecA/RadA recombinase	GO:0006310 DNA recombination -!- GO:0006281 DNA repair -!- GO:0006508 proteolysis and peptidolysis -!- GO:0009432 SOS response	gg	t
recB	CDS	exonuclease V (RecBCD complex), beta subunit	Cytoplasmic	COG1074;ATP-dependent exoDNAse (exonuclease V) beta subunit (contains helicase and exonuclease domains)	GO:0006308 DNA catabolism -!- GO:0006308 DNA catabolism -!- GO:0006310 DNA recombination	o	tt
recC	CDS	exonuclease V (RecBCD complex), gamma chain	Cytoplasmic	COG1330;Exonuclease V gamma subunit	GO:0006308 DNA catabolism -!- GO:0006308 DNA catabolism -!- GO:0006310 DNA recombination	o	tt
recD	CDS	exonuclease V (RecBCD complex), alpha chain	Cytoplasmic	COG0507;ATP-dependent exoDNAse (exonuclease V), alpha subunit - helicase superfamily I member	GO:0006308 DNA catabolism -!- GO:0006308 DNA catabolism -!- GO:0006310 DNA recombination	gg	t
recE	CDS	Rac prophage; exonuclease VIII, 5' -> 3' specific dsDNA exonuclease	Cytoplasmic		GO:0006308 DNA catabolism -!- GO:0006281 DNA repair -!- GO:0006308 DNA catabolism	gg	tt
recF	CDS	gap repair protein	Cytoplasmic	COG1195;Recombinational DNA repair ATPase (RecF pathway)	GO:0006261 DNA dependent DNA replication -!- GO:0006281 DNA repair -!- GO:0006310 DNA	o	tt
recG	CDS	ATP-dependent DNA helicase	Cytoplasmic	COG1200;RecG-like helicase	GO:0006261 DNA dependent DNA replication	o	tt
recJ	CDS	ssDNA exonuclease, 5' --> 3'-specific	Cytoplasmic	COG0608;Single-stranded DNA-specific exonuclease	GO:0006308 DNA catabolism -!- GO:0006308 DNA catabolism	g	t
recN	CDS	recombination and repair protein	Cytoplasmic		GO:0006310 DNA recombination -!- GO:0006281 DNA repair -!- GO:0009432 SOS response		
recO	CDS	gap repair protein	Cytoplasmic	COG1381;Recombinational DNA repair protein (RecF pathway)	GO:0006281 DNA repair -!- GO:0006310 DNA recombination	gg	tt
recQ	CDS	ATP-dependent DNA helicase	Cytoplasmic		GO:0006261 DNA dependent DNA replication -!- GO:0006281 DNA repair -!- GO:0006310 DNA		
recR	CDS	gap repair protein	Cytoplasmic	COG0353;Recombinational DNA repair protein (RecF pathway)	GO:0006281 DNA repair -!- GO:0006310 DNA recombination	o	tt
recT	CDS	Rac prophage; recombination and repair protein	Cytoplasmic	COG3723;Recombinational DNA repair protein (RecE pathway)		gg	tt
recX	CDS	regulatory protein for RecA	Cytoplasmic	COG2137;Uncharacterized protein conserved in bacteria		o	tt
relA	CDS	(p)ppGpp synthetase I/GTP pyrophosphokinase	Cytoplasmic	COG0317;Guanosine polyphosphate pyrophosphohydrolases/synthetases	GO:0015949 nucleobase, nucleoside and nucleotide interconversion	o	t
relB	CDS	Qin prophage; bifunctional antitoxin of the RelE-RelB toxin-antitoxin system -!- transcriptional repressor	Cytoplasmic	COG3077;DNA-damage-inducible protein J	GO:0006350 transcription	gg	o
relE	CDS	Qin prophage; toxin of the RelE-RelB toxin-antitoxin system	Cytoplasmic	COG2026;Cytotoxic translational repressor of toxin-antitoxin stability system	GO:0006350 transcription	o	tt
rem	CDS	Qin prophage; predicted protein	Cytoplasmic			o	tt
renD	CDS	DLP12 prophage; predicted protein	Cytoplasmic			gg	o
rep	CDS	DNA helicase and single-stranded DNA-dependent ATPase	Cytoplasmic		GO:0006261 DNA dependent DNA replication		
rfaB	CDS	UDP-D-galactose:(glucosyl)lipopolysaccharide-1,6-D-galactosyltransferase	Cytoplasmic	COG0438;Glycosyltransferase		o	tt
rfaC	CDS	ADP-heptose:LPS heptosyl transferase I	Cytoplasmic	COG0859;ADP-heptose:LPS heptosyltransferase	GO:0009244 lipopolysaccharide core region biosynthesis	o	tt
rfaD	CDS	ADP-L-glycero-D-mannoheptose-6-epimerase, NAD(P)-binding	Cytoplasmic	COG0451;Nucleoside-diphosphate-sugar epimerases	GO:0009435 nicotinamide adenine dinucleotide biosynthesis	o	o
rfaE	CDS	fused heptose 7-phosphate kinase -!- heptose 1-phosphate adenyltransferase	Cytoplasmic	COG2870;ADP-heptose synthase, bifunctional sugar kinase/adenylyltransferase	GO:0016052 carbohydrate catabolism -!- GO:0009244 lipopolysaccharide core region	gg	tt
rfaF	CDS	ADP-heptose:LPS heptosyltransferase II	Cytoplasmic	COG0859;ADP-heptose:LPS heptosyltransferase	GO:0009244 lipopolysaccharide core region biosynthesis	g	t
rfaG	CDS	glucosyltransferase I	Cytoplasmic	COG0438;Glycosyltransferase	GO:0009244 lipopolysaccharide core region biosynthesis -!- GO:0009432 SOS response	o	tt
rfaH	CDS	DNA-binding transcriptional antiterminator	Cytoplasmic	COG0250;Transcription antiterminator	GO:0009244 lipopolysaccharide core region biosynthesis -!- GO:0009297 fimbrial biogenesis -!- GO:0006350 transcription	gg	t
rfaI	CDS	UDP-D-galactose:(glucosyl)lipopolysaccharide-alpha-1,3-D-galactosyltransferase	Cytoplasmic	COG1442;Lipopolysaccharide biosynthesis proteins, LPS:glycosyltransferases		o	tt
rfaJ	CDS	UDP-D-glucose:(galactosyl)lipopolysaccharide glucosyltransferase	Cytoplasmic	COG1442;Lipopolysaccharide biosynthesis proteins, LPS:glycosyltransferases		o	o
rfaK	CDS	lipopolysaccharide core biosynthesis	Cytoplasmic	COG0859;ADP-heptose:LPS heptosyltransferase	GO:0009244 lipopolysaccharide core region biosynthesis	o	tt
rfaL	CDS	O-antigen ligase	Integral Membrane Protein	COG3307;Lipid A core - O-antigen ligase and related enzymes	GO:0009244 lipopolysaccharide core region biosynthesis	o	tt
rfaP	CDS	kinase that phosphorylates core heptose of lipopolysaccharide	Cytoplasmic		GO:0009244 lipopolysaccharide core region biosynthesis	o	tt
rfaQ	CDS	lipopolysaccharide core biosynthesis protein	Cytoplasmic	COG0859;ADP-heptose:LPS heptosyltransferase	GO:0009244 lipopolysaccharide core region biosynthesis	gg	t
rfaS	CDS	lipopolysaccharide core biosynthesis protein	Cytoplasmic		GO:0009244 lipopolysaccharide core region biosynthesis	g	o
rfaY	CDS	lipopolysaccharide core biosynthesis protein	Cytoplasmic		GO:0009244 lipopolysaccharide core region biosynthesis	o	tt
rfaZ	CDS	lipopolysaccharide core biosynthesis protein	Cytoplasmic		GO:0009244 lipopolysaccharide core region biosynthesis	g	t
rfbA	CDS	glucose-1-phosphate thymidylyltransferase	Cytoplasmic	COG1209;dTDP-glucose pyrophosphorylase	GO:0009243 O antigen biosynthesis -!- GO:0019305 dTDP-rhamnose biosynthesis -!- GO:0009103 lipopolysaccharide biosynthesis	gg	t
rfbB	CDS	dTDP-glucose 4,6 dehydratase, NAD(P)-binding	Cytoplasmic	COG1088;dTDP-D-glucose 4,6-dehydratase	GO:0009243 O antigen biosynthesis -!- GO:0009226 nucleotide-sugar biosynthesis -!- GO:0009435 nicotinamide adenine dinucleotide biosynthesis	gg	t
rfbC	CDS	dTDP-4-deoxyrhamnose-3,5-epimerase	Cytoplasmic	COG1898;dTDP-4-dehydrorhamnose 3,5-epimerase and related enzymes	GO:0009226 nucleotide-sugar biosynthesis -!- GO:0009243 O antigen biosynthesis -!- GO:0009243 O antigen biosynthesis -!- GO:0009103 lipopolysaccharide biosynthesis -!- GO:0019305 dTDP-rhamnose biosynthesis	g	o
rfbD	CDS	dTDP-4-dehydrorhamnose reductase subunit, NAD(P)-binding, of dTDP-L-rhamnose synthase	Cytoplasmic	COG1091;dTDP-4-dehydrorhamnose reductase	GO:0009243 O antigen biosynthesis -!- GO:0009226 nucleotide-sugar biosynthesis	gg	o
rfbX	CDS	predicted polisoprenol-linked O-antigen transporter	Integral Membrane Protein	COG2244;Membrane protein involved in the export of O-antigen and teichoic acid	GO:0009243 O antigen biosynthesis	o	tt
rfe	CDS	UDP-GlcNAc:undecaprenylphosphate GlcNAc-1-phosphate transferase	Integral Membrane Protein	COG0472;UDP-N-acetylmuramyl pentapeptide phosphotransferase/UDP-N-acetylglucosamine-1-phosp hate transferase	GO:0009246 enterobacterial common antigen biosynthesis	o	tt
rffA	CDS	TDP-4-oxo-6-deoxy-D-glucose transaminase	Cytoplasmic	COG0399;Predicted pyridoxal phosphate-dependent enzyme apparently involved in regulation of cell wall biogenesis	GO:0009246 enterobacterial common antigen biosynthesis	gg	o
rffC	CDS	TDP-fucosamine acetyltransferase	Cytoplasmic		GO:0009244 lipopolysaccharide core region biosynthesis		
rffD	CDS	UDP-N-acetyl-D-mannosaminuronic acid dehydrogenase	Periplasmic		GO:0009246 enterobacterial common antigen biosynthesis		
rffE	CDS	UDP-N-acetyl glucosamine-2-epimerase	Cytoplasmic		GO:0009246 enterobacterial common antigen biosynthesis		
rffG	CDS	dTDP-glucose 4,6-dehydratase	Cytoplasmic		GO:0009243 O antigen biosynthesis -!- GO:0009246 enterobacterial common antigen biosynthesis -!- GO:0009435 nicotinamide adenine dinucleotide biosynthesis		
rffH	CDS	glucose-1-phosphate thymidylyltransferase	Cytoplasmic	COG1209;dTDP-glucose pyrophosphorylase	GO:0009243 O antigen biosynthesis -!- GO:0009246 enterobacterial common antigen biosynthesis	gg	t
rffM	CDS	UDP-N-acetyl-D-mannosaminuronic acid transferase	Cytoplasmic	COG1922;Teichoic acid biosynthesis proteins	GO:0009246 enterobacterial common antigen biosynthesis	gg	tt
rffT	CDS	TDP-Fuc4NAc:lipidII transferase	Cytoplasmic				
rhaA	CDS	L-rhamnose isomerase	Cytoplasmic		GO:0016052 carbohydrate		
rhaB	CDS	rhamnulokinase	Cytoplasmic	COG1070;Sugar (pentulose and hexulose) kinases	GO:0016052 carbohydrate	o	tt
rhaD	CDS	rhamnulose-1-phosphate aldolase	Cytoplasmic	COG0235;Ribulose-5-phosphate 4-epimerase and related epimerases and aldolases	GO:0016052 carbohydrate catabolism	gg	o
rhaR	CDS	DNA-binding transcriptional activator, L-rhamnose-binding	Cytoplasmic	COG2207;AraC-type DNA-binding domain-containing proteins	GO:0016052 carbohydrate catabolism -!- GO:0006350	g	t
rhaS	CDS	DNA-binding transcriptional activator, L-rhamnose-binding	Cytoplasmic	COG2207;AraC-type DNA-binding domain-containing proteins	GO:0016052 carbohydrate catabolism -!- GO:0006350	gg	o
rhaT	CDS	L-rhamnose:proton symporter	Integral Membrane Protein	COG0697;Permeases of the drug/metabolite transporter (DMT) superfamily	GO:0016052 carbohydrate	o	tt
rhlB	CDS	ATP-dependent RNA helicase	Cytoplasmic	COG0513;Superfamily II DNA and RNA helicases		g	t
rhlE	CDS	RNA helicase	Cytoplasmic	COG0513;Superfamily II DNA and RNA helicases	GO:0009451 RNA modification	o	tt
rho	CDS	transcription termination factor	Cytoplasmic	COG1158;Transcription termination factor	GO:0006350 transcription	o	tt
rhoL	CDS	rho operon leader peptide	Cytoplasmic		GO:0006350 transcription	gg	o
rhsA	CDS	rhsA element core protein RshA	Cytoplasmic	COG3209;Rhs family protein		o	tt
rhsB	CDS	rhsB element core protein RshB	Cytoplasmic				
rhsC	CDS	rhsC element core protein RshC	Cytoplasmic	COG3209;Rhs family protein		o	t

rhsD	CDS	rhsD element protein	Cytoplasmic	COG3209;Rhs family protein		o	t
rhsE	CDS	rhsE element core protein RshE	Cytoplasmic	COG3209;Rhs family protein		o	tt
rhtA	CDS	threonine and homoserine efflux system	Integral Membrane Protein	COG5006;Predicted permease, DMT superfamily		o	tt
rhtB	CDS	neutral amino-acid efflux system	Integral Membrane Protein		GO:0006564 L-serine biosynthesis		
rhtC	CDS	threonine efflux system	Integral Membrane Protein		GO:0009088 threonine biosynthesis		
ribA	CDS	GTP cyclohydrolase II	Cytoplasmic	COG0807;GTP cyclohydrolase II		gg	t
ribB	CDS	3,4 dihydroxy-2-butanone-4-phosphate synthase	Cytoplasmic	COG0108;3,4-dihydroxy-2-butanone 4-phosphate synthase		gg	t
ribC	CDS	riboflavin synthase, alpha subunit	Cytoplasmic	COG0307;Riboflavin synthase alpha chain		gg	t
ribD	CDS	fused diaminohydroxyphosphoribosylaminopyrimidine deaminase -!- 5-amino-6-(5-phosphoribosylamino) uracil reductase	Cytoplasmic	COG0117;Pyrimidine deaminase -!- COG1985;Pyrimidine reductase, riboflavin biosynthesis		gg	t
ribE	CDS	riboflavin synthase beta chain	Cytoplasmic	COG0054;Riboflavin synthase beta-chain		o	o
ribF	CDS	bifunctional riboflavin kinase -!- FAD synthetase	Cytoplasmic	COG0196;FAD synthase		o	tt
rihA	CDS	ribonucleoside hydrolase 1	Cytoplasmic	COG1957;Inosine-uridine nucleoside N-ribohydrolase	GO:0009226 nucleotide-sugar biosynthesis	gg	t
rihB	CDS	ribonucleoside hydrolase 2	Cytoplasmic	COG1957;Inosine-uridine nucleoside N-ribohydrolase		g	t
rihC	CDS	ribonucleoside hydrolase 3	Cytoplasmic	COG1957;Inosine-uridine nucleoside N-ribohydrolase	GO:0009226 nucleotide-sugar biosynthesis	g	o
rimI	CDS	acetylase for 30S ribosomal subunit protein S18	Cytoplasmic	COG0456;Acetyltransferases	GO:0006464 protein modification	gg	o
rimJ	CDS	ribosomal-protein-S5-alanine N-acetyltransferase	Cytoplasmic	COG1670;Acetyltransferases, including N-acetylases of ribosomal proteins	GO:0006464 protein modification	gg	o
rimK	CDS	ribosomal protein S6 modification protein	Cytoplasmic	COG0189;Glutathione synthase/Ribosomal protein S6 modification enzyme (glutaminyl transferase)	GO:0006412 protein biosynthesis	gg	t
rimL	CDS	ribosomal-protein-L7/L12-serine acetyltransferase	Cytoplasmic	COG1670;Acetyltransferases, including N-acetylases of ribosomal proteins	GO:0006464 protein modification	gg	o
rimM	CDS	16S rRNA processing protein	Cytoplasmic		GO:0009451 RNA modification		
rlmB	CDS	23S rRNA (Gm2251)-methyltransferase	Cytoplasmic	COG0566;rRNA methylases	GO:0009451 RNA modification	gg	t
rlpA	CDS	minor lipoprotein	Outer Membrane Lipoprotein	COG0797;Lipoproteins		o	tt
rlpB	CDS	minor lipoprotein	Membrane Lipoprotein	COG2980;Rare lipoprotein B	GO:0042158 lipoprotein biosynthesis	o	tt
rluA	CDS	pseudouridine synthase for 23S rRNA (position 746) and tRNAphe(position 32)	Cytoplasmic	COG0564;Pseudouridylate synthases, 23S RNA-specific	GO:0009451 RNA modification	gg	t
rluB	CDS	23S rRNA pseudouridylate synthase	Cytoplasmic	COG1187;16S rRNA uridine-516 pseudouridylate synthase and related pseudouridylate synthases	GO:0009451 RNA modification	gg	t
rluC	CDS	23S rRNA pseudouridylate synthase	Cytoplasmic	COG0564;Pseudouridylate synthases, 23S RNA-specific	GO:0009451 RNA modification	g	tt
rluD	CDS	23S rRNA pseudouridine synthase	Cytoplasmic	COG0564;Pseudouridylate synthases, 23S RNA-specific	GO:0009451 RNA modification		
rmf	CDS	ribosome modulation factor	Cytoplasmic	COG3130;Ribosome modulation factor	GO:0006457 protein folding	gg	t
rmuC	CDS	predicted recombination limiting protein	Cytoplasmic	COG1322;Uncharacterized protein conserved in bacteria		o	tt
rna	CDS	ribonuclease I	Periplasmic	COG3719;Ribonuclease I	GO:0006401 RNA catabolism -!- GO:0006401 RNA catabolism	o	tt
rnb	CDS	ribonuclease II	Cytoplasmic	COG4776;Exoribonuclease II	GO:0006401 RNA catabolism -!- GO:0006401 RNA catabolism	gg	o
rnc	CDS	RNase III	Cytoplasmic	COG0571;dsRNA-specific ribonuclease	GO:0006401 RNA catabolism -!- GO:0006401 RNA catabolism	gg	tt
rnd	CDS	ribonuclease D	Cytoplasmic	COG0349;Ribonuclease D	GO:0006401 RNA catabolism -!- GO:0009451 RNA modification	gg	tt
rne	CDS	fused ribonucleaseE endoribonuclease -!- scaffold for formation of degradosome	Cytoplasmic	COG1530;Ribonucleases G and E	GO:0006401 RNA catabolism -!- GO:0006401 RNA catabolism	o	tt
rng	CDS	ribonuclease G	Cytoplasmic	COG1530;Ribonucleases G and E	GO:0009451 RNA modification	g	o
rnhA	CDS	ribonuclease HI, degrades RNA of DNA-RNA hybrids	Cytoplasmic	COG0328;Ribonuclease HI	GO:0006401 RNA catabolism -!- GO:0006261 DNA dependent DNA replication -!- GO:0006508 proteolysis and peptidolysis	o	tt
rnhB	CDS	ribonuclease HII, degrades RNA of DNA-RNA hybrids	Cytoplasmic	COG0164;Ribonuclease HII	GO:0006401 RNA catabolism -!- GO:0006401 RNA catabolism	gg	o
rnk	CDS	regulator of nucleoside diphosphate kinase	Cytoplasmic	COG0782;Transcription elongation factor	GO:0015949 nucleobase, nucleoside and nucleotide interconversion	gg	t
rnpA	CDS	protein C5 component of RNase P	Cytoplasmic	COG0594;RNase P protein component	GO:0006401 RNA catabolism -!- GO:0009451 RNA modification	g	tt
rnpB	misc_RNA	RNase P, RNA component precursor RnpB					
rnr	CDS	exoribonuclease R, RNase R	Cytoplasmic		GO:0006401 RNA catabolism -!- GO:0006401 RNA catabolism		
rnt	CDS	ribonuclease T (RNase T)	Cytoplasmic	COG0847;DNA polymerase III, epsilon subunit and related 3'-5' exonucleases	GO:0006401 RNA catabolism -!- GO:0006308 DNA catabolism -!- GO:0006401 RNA catabolism -!- GO:0006308 DNA catabolism	o	tt
rob	CDS	DNA-binding transcriptional activator	Cytoplasmic	COG2207;AraC-type DNA-binding domain-containing proteins	GO:0006261 DNA dependent DNA replication -!- GO:0006350 transcription	o	tt
rof	CDS	modulator of Rho-dependent transcription termination	Cytoplasmic	COG4568;Transcriptional antiterminator	GO:0006350 transcription	o	tt
rpe	CDS	D-ribulose-5-phosphate 3-epimerase	Cytoplasmic	COG0036;Pentose-5-phosphate-3-epimerase	GO:0016052 carbohydrate catabolism -!- GO:0009052 pentose-phosphate shunt, non-oxidative	gg	o
rph	CDS	ribonuclease PH (pseudogene)	Cytoplasmic	COG0689;RNase PH	GO:0006401 RNA catabolism -!- GO:0006401 RNA catabolism	g	o
rpiA	CDS	ribosephosphate isomerase, constitutive	Cytoplasmic	COG0120;Ribose 5-phosphate isomerase	GO:0016052 carbohydrate catabolism -!- GO:0009052 pentose-phosphate shunt, non-oxidative		
rpiB	CDS	ribose 5-phosphate isomerase B/allose 6-phosphate isomerase	Cytoplasmic	COG0698;Ribose 5-phosphate isomerase RpiB	GO:0016052 carbohydrate catabolism -!- GO:0009052 pentose-phosphate shunt, non-oxidative	g	o
rpiR	CDS	DNA-binding transcriptional repressor	Cytoplasmic		GO:0016052 carbohydrate catabolism -!- GO:0009052 pentose-phosphate shunt, non-oxidative branch -!- GO:0006350 transcription	gg	t
rplA	CDS	50S ribosomal subunit protein L1	Cytoplasmic	COG0081;Ribosomal protein L1	GO:0006412 protein biosynthesis -!- GO:0009386 translational attenuation	o	tt
rplB	CDS	50S ribosomal subunit protein L2	Cytoplasmic	COG0090;Ribosomal protein L2	GO:0006412 protein biosynthesis	o	tt
rplC	CDS	50S ribosomal subunit protein L3	Cytoplasmic	COG0087;Ribosomal protein L3	GO:0006412 protein biosynthesis	gg	tt
rplD	CDS	50S ribosomal subunit protein L4	Cytoplasmic	COG0088;Ribosomal protein L4	GO:0006412 protein biosynthesis -!- GO:0006355 regulation of transcription, DNA-dependent -!- GO:0009386 translational attenuation	o	tt
rplE	CDS	50S ribosomal subunit protein L5	Cytoplasmic	COG0094;Ribosomal protein L5	GO:0006412 protein biosynthesis	gg	t
rplF	CDS	50S ribosomal subunit protein L6	Cytoplasmic	COG0097;Ribosomal protein L6P/L9E	GO:0006412 protein biosynthesis	gg	tt
rplI	CDS	50S ribosomal subunit protein L9	Cytoplasmic	COG0359;Ribosomal protein L9	GO:0006412 protein biosynthesis	o	o
rplJ	CDS	50S ribosomal subunit protein L10	Cytoplasmic	COG0244;Ribosomal protein L10	GO:0006412 protein biosynthesis	o	tt
rplK	CDS	50S ribosomal subunit protein L11	Cytoplasmic	COG0080;Ribosomal protein L11	GO:0006412 protein biosynthesis	gg	t
rplL	CDS	50S ribosomal subunit protein L7/L12	Cytoplasmic	COG0222;Ribosomal protein L7/L12	GO:0006412 protein biosynthesis	o	o
rplM	CDS	50S ribosomal subunit protein L13	Cytoplasmic	COG0102;Ribosomal protein L13	GO:0006412 protein biosynthesis	gg	tt
rplN	CDS	50S ribosomal subunit protein L14	Cytoplasmic	COG0093;Ribosomal protein L14	GO:0006412 protein biosynthesis	o	tt
rplO	CDS	50S ribosomal subunit protein L15	Cytoplasmic	COG0200;Ribosomal protein L15	GO:0006412 protein biosynthesis	g	tt
rplP	CDS	50S ribosomal subunit protein L16	Cytoplasmic	COG0197;Ribosomal protein L16/L10E	GO:0006412 protein biosynthesis	o	tt
rplQ	CDS	50S ribosomal subunit protein L17	Cytoplasmic	COG0203;Ribosomal protein L17	GO:0006412 protein biosynthesis	o	tt
rplR	CDS	50S ribosomal subunit protein L18	Cytoplasmic	COG0256;Ribosomal protein L18	GO:0006412 protein biosynthesis	gg	o
rplS	CDS	50S ribosomal subunit protein L19	Cytoplasmic	COG0335;Ribosomal protein L19	GO:0006412 protein biosynthesis	o	tt
rplT	CDS	50S ribosomal subunit protein L20	Cytoplasmic	COG0292;Ribosomal protein L20	GO:0006412 protein biosynthesis	gg	t
rplU	CDS	50S ribosomal subunit protein L21	Cytoplasmic	COG0261;Ribosomal protein L21	GO:0006412 protein biosynthesis	g	o
rplV	CDS	50S ribosomal subunit protein L22	Cytoplasmic	COG0091;Ribosomal protein L22	GO:0006412 protein biosynthesis	g	tt
rplW	CDS	50S ribosomal subunit protein L23	Cytoplasmic	COG0089;Ribosomal protein L23	GO:0006412 protein biosynthesis	gg	tt
rplX	CDS	50S ribosomal subunit protein L24	Cytoplasmic	COG0198;Ribosomal protein L24	GO:0006412 protein biosynthesis	gg	t
rplY	CDS	50S ribosomal subunit protein L25	Cytoplasmic	COG1825;Ribosomal protein L25 (general stress protein Ctc)	GO:0006412 protein biosynthesis	gg	o
rpmA	CDS	50S ribosomal subunit protein L27	Cytoplasmic	COG0211;Ribosomal protein L27	GO:0006412 protein biosynthesis	gg	tt
rpmB	CDS	50S ribosomal subunit protein L28	Cytoplasmic	COG0227;Ribosomal protein L28	GO:0006412 protein biosynthesis	gg	tt
rpmC	CDS	50S ribosomal subunit protein L29	Cytoplasmic	COG0255;Ribosomal protein L29	GO:0006412 protein biosynthesis	gg	o
rpmD	CDS	50S ribosomal subunit protein L30	Cytoplasmic	COG1841;Ribosomal protein L30/L7E	GO:0006412 protein biosynthesis	gg	t
rpmE	CDS	50S ribosomal subunit protein L31	Cytoplasmic	COG0254;Ribosomal protein L31	GO:0006412 protein biosynthesis	gg	o
rpmF	CDS	50S ribosomal subunit protein L32	Cytoplasmic	COG0333;Ribosomal protein L32	GO:0006412 protein biosynthesis	gg	tt
rpmG	CDS	50S ribosomal subunit protein L33	Cytoplasmic	COG0267;Ribosomal protein L33	GO:0006412 protein biosynthesis	gg	tt
rpmH	CDS	50S ribosomal subunit protein L34	Cytoplasmic	COG0230;Ribosomal protein L34	GO:0006412 protein biosynthesis	gg	tt
rpmI	CDS	50S ribosomal subunit protein L35	Cytoplasmic		GO:0006412 protein biosynthesis	o	t
rpmJ	CDS	50S ribosomal subunit protein L36	Cytoplasmic	COG0257;Ribosomal protein L36	GO:0006412 protein biosynthesis	gg	o
rpoA	CDS	RNA polymerase, alpha subunit	Cytoplasmic	COG0202;DNA-directed RNA polymerase, alpha subunit/40 kD subunit	GO:0006350 transcription	gg	t
rpoB	CDS	RNA polymerase, beta subunit	Cytoplasmic	COG0085;DNA-directed RNA polymerase, beta subunit/140 kD subunit	GO:0006350 transcription	g	tt
rpoC	CDS	RNA polymerase, beta prime subunit	Cytoplasmic	COG0086;DNA-directed RNA polymerase, beta' subunit/160 kD subunit	GO:0006350 transcription	o	tt
rpoD	CDS	RNA polymerase, sigma 70 (sigma D) factor	Cytoplasmic	COG0568;DNA-directed RNA polymerase, sigma subunit (sigma70/sigma32)	GO:0006350 transcription	o	tt
rpoE	CDS	RNA polymerase, sigma 24 (sigma E) factor	Cytoplasmic	COG1595;DNA-directed RNA polymerase specialized sigma subunit, sigma24 homolog	GO:0006350 transcription -!- GO:0009266 response to	gg	t
rpoH	CDS	RNA polymerase, sigma 32 (sigma H) factor	Cytoplasmic	COG0568;DNA-directed RNA polymerase, sigma subunit (sigma70/sigma32)	GO:0006350 transcription -!- GO:0009266 response to	o	tt
rpoN	CDS	RNA polymerase, sigma 54 (sigma N) factor	Cytoplasmic	COG1508;DNA-directed RNA polymerase specialized sigma subunit, sigma54 homolog	GO:0006807 nitrogen metabolism -!- GO:0006350 transcription	o	tt
rpoS	CDS	RNA polymerase, sigma S (sigma 38) factor	Cytoplasmic	COG0568;DNA-directed RNA polymerase, sigma subunit (sigma70/sigma32)	GO:0006350 transcription -!- GO:0006970 response to osmotic stress		
rpoZ	CDS	RNA polymerase, omega subunit	Cytoplasmic	COG1758;DNA-directed RNA polymerase, subunit K/omega	GO:0006350 transcription	gg	o
rprA	misc_RNA	regulatory sRNA					
rpsA	CDS	30S ribosomal subunit protein S1	Cytoplasmic	COG0539;Ribosomal protein S1	GO:0006412 protein biosynthesis	o	tt
rpsB	CDS	30S ribosomal subunit protein S2	Cytoplasmic	COG0052;Ribosomal protein S2	GO:0006412 protein biosynthesis	o	tt
rpsC	CDS	30S ribosomal subunit protein S3	Cytoplasmic	COG0092;Ribosomal protein S3	GO:0006412 protein biosynthesis	o	tt

rpsD	CDS	30S ribosomal subunit protein S4	Cytoplasmic	COG0522;Ribosomal protein S4 and related proteins	GO:0006412 protein biosynthesis	o	tt
rpsE	CDS	30S ribosomal subunit protein S5	Cytoplasmic	COG0098;Ribosomal protein S5	GO:0006412 protein biosynthesis	gg	o
rpsF	CDS	30S ribosomal subunit protein S6	Cytoplasmic	COG0360;Ribosomal protein S6	GO:0006412 protein biosynthesis	o	tt
rpsG	CDS	30S ribosomal subunit protein S7	Cytoplasmic	COG0049;Ribosomal protein S7	GO:0006412 protein biosynthesis	o	tt
rpsH	CDS	30S ribosomal subunit protein S8	Cytoplasmic	COG0096;Ribosomal protein S8	GO:0006412 protein biosynthesis -!- GO:0009386 translational attenuation	o	tt
rpsI	CDS	30S ribosomal subunit protein S9	Cytoplasmic	COG0103;Ribosomal protein S9	GO:0006412 protein biosynthesis	gg	tt
rpsJ	CDS	30S ribosomal subunit protein S10	Cytoplasmic	COG0051;Ribosomal protein S10	GO:0006412 protein biosynthesis	o	o
rpsK	CDS	30S ribosomal subunit protein S11	Cytoplasmic	COG0100;Ribosomal protein S11	GO:0006412 protein biosynthesis	gg	t
rpsL	CDS	30S ribosomal subunit protein S12	Cytoplasmic	COG0048;Ribosomal protein S12	GO:0006412 protein biosynthesis	gg	tt
rpsM	CDS	30S ribosomal subunit protein S13	Cytoplasmic	COG0099;Ribosomal protein S13	GO:0006412 protein biosynthesis	o	tt
rpsN	CDS	30S ribosomal subunit protein S14	Cytoplasmic	COG0199;Ribosomal protein S14	GO:0006412 protein biosynthesis	gg	tt
rpsO	CDS	30S ribosomal subunit protein S15	Cytoplasmic	COG0184;Ribosomal protein S15P/S13E	GO:0006412 protein biosynthesis	gg	t
rpsP	CDS	30S ribosomal subunit protein S16	Cytoplasmic	COG0228;Ribosomal protein S16	GO:0006412 protein biosynthesis	gg	t
rpsQ	CDS	30S ribosomal subunit protein S17	Cytoplasmic	COG0186;Ribosomal protein S17	GO:0006412 protein biosynthesis	gg	t
rpsR	CDS	30S ribosomal subunit protein S18	Cytoplasmic	COG0238;Ribosomal protein S18	GO:0006412 protein biosynthesis	o	tt
rpsS	CDS	30S ribosomal subunit protein S19	Cytoplasmic	COG0185;Ribosomal protein S19	GO:0006412 protein biosynthesis	o	tt
rpsT	CDS	30S ribosomal subunit protein S20	Cytoplasmic	COG0268;Ribosomal protein S20	GO:0006412 protein biosynthesis	gg	t
rpsU	CDS	30S ribosomal subunit protein S21	Cytoplasmic	COG0828;Ribosomal protein S21	GO:0006412 protein biosynthesis	gg	t
rraA	CDS	ribonuclease E (RNase E) inhibitor protein	Cytoplasmic	COG0684;Demethylmenaquinone methyltransferase		o	tt
rrfA	rRNA	5S rRNA (rrfA)					
rrfB	rRNA	5S rRNA (rrfB)					
rrfC	rRNA	5S rRNA (rrfC)					
rrfD	rRNA	5S rRNA (rrfD)					
rrfE	rRNA	5S rRNA (rrfE)					
rrfF	rRNA	5S rRNA (rrfF)					
rrfG	rRNA	5S rRNA (rrfG)					
rrfH	rRNA	5S rRNA (rrfH)					
rrlA	rRNA	23S rRNA (rrlA)					
rrlB	rRNA	23S rRNA (rrlB)					
rrlC	rRNA	23S rRNA (rrlC)					
rrlD	rRNA	23S rRNA (rrlD)					
rrlE	rRNA	23S rRNA (rrlE)					
rrlG	rRNA	23S rRNA (rrlG)					
rrlH	rRNA	23S rRNA (rrlH)					
rrmA	CDS	23S rRNA m1G745 methyltransferase	Cytoplasmic	COG0500;SAM-dependent methyltransferases	GO:0009451 RNA modification	gg	t
rrmJ	CDS	23S rRNA methyltransferase	Cytoplasmic	COG0293;23S rRNA methylase		gg	tt
rrsA	rRNA	16S rRNA (rrnA)					
rrsB	rRNA	16S rRNA (rrnB)					
rrsC	rRNA	16S rRNA (rrnC)					
rrsD	rRNA	16S rRNA (rrnD)					
rrsE	rRNA	16S rRNA (rrnE)					
rrsG	rRNA	16S rRNA (rrnG)					
rrsH	rRNA	16S rRNA (rrsH)					
rsd	CDS	stationary phase protein, binds sigma 70 RNA polymerase subunit	Cytoplasmic	COG3160;Regulator of sigma D	GO:0006350 transcription	gg	tt
rseA	CDS	anti-sigma factor	Integral Membrane Protein	COG3073;Negative regulator of sigma E activity	GO:0006350 transcription -!- GO:0009266 response to	o	tt
rseB	CDS	anti-sigma factor	Periplasmic	COG3026;Negative regulator of sigma E activity	GO:0006350 transcription -!- GO:0042594 response to starvation	o	tt
rseC	CDS	RseC protein involved in reduction of the SoxR iron-sulfur cluster	Integral Membrane Protein	COG3086;Positive regulator of sigma E activity	GO:0006350 transcription -!- GO:0042594 response to starvation	o	tt
rsgA	CDS	ribosome small subunit-dependent GTPase A	Cytoplasmic			gg	t
rsmB	CDS	16S rRNA m5C967 methyltransferase, S-adenosyl-L-methionine-dependent	Cytoplasmic	COG0144;tRNA and rRNA cytosine-C5-methylases	GO:0009451 RNA modification	o	tt
rsmC	CDS	16S RNA m2G1207 methylase	Cytoplasmic	COG2813;16S RNA G1207 methylase RsmC	GO:0009451 RNA modification	gg	t
rspA	CDS	predicted dehydratase	Cytoplasmic	COG4948;L-alanine-DL-glutamate epimerase and related enzymes of enolase superfamily	GO:0042594 response to starvation	gg	t
rspB	CDS	predicted oxidoreductase, Zn-dependent and NAD(P)-binding	Cytoplasmic	COG1063;Threonine dehydrogenase and related Zn-dependent dehydrogenases	GO:0042594 response to starvation	gg	o
rssA	CDS	conserved protein	Cytoplasmic	COG1752;Predicted esterase of the alpha-beta hydrolase superfamily		gg	t
rssB	CDS	response regulator of RpoS	Cytoplasmic	COG0784;FOG: CheY-like receiver	GO:0006350 transcription	gg	t
rstA	CDS	DNA-bindng response regulator in two-component regulatory system with RstB	Cytoplasmic	COG0745;Response regulators consisting of a CheY-like receiver domain and a winged-helix DNA-binding domain	GO:0006350 transcription	gg	t
rstB	CDS	sensory histidine kinase in two-component regulatory system with RstA	Integral Membrane Protein	COG0642;Signal transduction histidine kinase	GO:0006464 protein modification	o	tt
rsuA	CDS	16S rRNA pseudouridylate 516 synthase	Cytoplasmic	COG1187;16S rRNA uridine-516 pseudouridylate synthase and related pseudouridylate synthases	GO:0009451 RNA modification	g	o
rsxA	CDS	predicted inner membrane subunit	Integral Membrane Protein	COG4657;Predicted NADH:ubiquinone oxidoreductase, subunit RnfA		o	tt
rsxB	CDS	predicted iron-sulfur protein	Periplasmic	COG2878;Predicted NADH:ubiquinone oxidoreductase, subunit RnfB		o	tt
rsxC	CDS	fused predicted 4Fe-4S ferredoxin-type protein	Cytoplasmic	COG4656;Predicted NADH:ubiquinone oxidoreductase, subunit RnfC		o	tt
rsxD	CDS	predicted inner membrane oxidoreductase	Integral Membrane Protein	COG4658;Predicted NADH:ubiquinone oxidoreductase, subunit RnfD		o	tt
rsxE	CDS	predicted inner membrane NADH-quinone reductase	Integral Membrane Protein	COG4660;Predicted NADH:ubiquinone oxidoreductase, subunit RnfE		o	tt
rsxG	CDS	predicted oxidoreductase	Periplasmic	COG4659;Predicted NADH:ubiquinone oxidoreductase, subunit RnfG		o	tt
rtcA	CDS	RNA 3'-terminal phosphate cyclase	Cytoplasmic		GO:0016070 RNA metabolism		
rtcB	CDS	conserved protein	Cytoplasmic	COG1690;Uncharacterized conserved protein		gg	t
rtcR	CDS	sigma 54-dependent transcriptional regulator of rtcBA expression	Cytoplasmic	COG4650;Sigma54-dependent transcription regulator containing an AAA-type ATPase domain and a DNA-binding domain	GO:0006350 transcription -!- GO:0006355 regulation of transcription, DNA-dependent	g	t
rtn	CDS	conserved protein	Membrane Anchored	COG2200;FOG: EAL domain		g	t
rttR	misc_RNA	regulatory sRNA					
rumA	CDS	23S rRNA (uracil-5)-methyltransferase	Cytoplasmic	COG2265;SAM-dependent methyltransferases related to tRNA (uracil-5-)-methyltransferase	GO:0009451 RNA modification	o	o
rumB	CDS	23S rRNA m(5)U747 methyltransferase	Cytoplasmic	COG2265;SAM-dependent methyltransferases related to tRNA (uracil-5-)-methyltransferase	GO:0009451 RNA modification	gg	t
rusA	CDS	DLP12 prophage; endonuclease RUS	Cytoplasmic	COG4570;Holliday junction resolvase	GO:0006308 DNA catabolism -!- GO:0006281 DNA repair -!- GO:0006308 DNA catabolism	o	tt
ruvA	CDS	component of RuvABC resolvasome, regulatory subunit	Cytoplasmic	COG0632;Holliday junction resolvasome, DNA-binding subunit	GO:0006281 DNA repair -!- GO:0006310 DNA recombination -!- GO:0009432 SOS response	g	tt
ruvB	CDS	ATP-dependent DNA helicase, component of RuvABC resolvasome	Cytoplasmic	COG2255;Holliday junction resolvasome, helicase subunit	GO:0006281 DNA repair -!- GO:0006310 DNA recombination -!- GO:0009432 SOS response	gg	t
ruvC	CDS	component of RuvABC resolvasome, endonuclease	Cytoplasmic	COG0817;Holliday junction resolvasome, endonuclease subunit	GO:0006281 DNA repair -!- GO:0006310 DNA recombination	g	tt
rybA	misc_RNA	small RNA					
rybB	misc_RNA	small RNA					
rydB	misc_RNA	regulatory sRNA					
ryeA	misc_RNA	small RNA					
ryeB	misc_RNA	small RNA					
ryeC	misc_RNA	small RNA					
ryeD	misc_RNA	small RNA					
ryeE	misc_RNA	small RNA					
ryfA	misc_RNA	small RNA					
rygA	misc_RNA	small RNA					
rygB	misc_RNA	small RNA					
rygC	misc_RNA	small RNA					
rygD	misc_RNA	small RNA					
ryhA	misc_RNA	small RNA					
ryhB	misc_RNA	regulatory sRNA					
ryiA	misc_RNA	regulatory sRNA					
ryjA	misc_RNA	small RNA					
rzoD	CDS	DLP12 prophage; predicted lipoprotein	Outer Membrane Lipoprotein				
rzoR	CDS	Rac prophage; predicted lipoprotein	Outer Membrane Lipoprotein				
rzpD	CDS	DLP12 prophage; predicted murein endopeptidase	Periplasmic				
rzpR	CDS	Rac prophage; predicted defective peptidase	Cytoplasmic				
sanA	CDS	predicted protein	Periplasmic	COG2949;Uncharacterized membrane protein	GO:0042493 response to drug	o	tt
sapA	CDS	predicted antimicrobial peptide transporter subunit -!- periplasmic-binding component of ABC superfamily	Periplasmic	COG4166;ABC-type oligopeptide transport system, periplasmic component	GO:0009063 amino acid catabolism	o	tt

sapB	CDS	predicted antimicrobial peptide transporter subunit -!- membrane component of ABC superfamily	Integral Membrane Protein	COG4168;ABC-type antimicrobial peptide transport system, permease component		gg	tt
sapC	CDS	predicted antimicrobial peptide transporter subunit -!- membrane component of ABC superfamily	Integral Membrane Protein	COG4171;ABC-type antimicrobial peptide transport system, permease component		o	tt
sapD	CDS	predicted antimicrobial peptide transporter subunit -!- ATP-binding component of ABC superfamily	Cytoplasmic	COG4170;ABC-type antimicrobial peptide transport system, ATPase component	GO:0009063 amino acid catabolism	o	tt
sapF	CDS	predicted antimicrobial peptide transporter subunit -!- ATP-binding component of ABC superfamily	Cytoplasmic	COG4167;ABC-type antimicrobial peptide transport system, ATPase component	GO:0009063 amino acid catabolism	gg	t
sbcB	CDS	exonuclease I	Cytoplasmic	COG2925;Exonuclease I	GO:0006308 DNA catabolism -!- GO:0006308 DNA catabolism	gg	tt
sbcC	CDS	exonuclease, dsDNA, ATP-dependent	Cytoplasmic	COG0419;ATPase involved in DNA repair	GO:0006308 DNA catabolism -!- GO:0006308 DNA catabolism	o	tt
sbcD	CDS	exonuclease, dsDNA, ATP-dependent	Cytoplasmic	COG0420;DNA repair exonuclease	GO:0006308 DNA catabolism -!- GO:0006308 DNA catabolism	gg	t
sbmA	CDS	predicted transporter	Integral Membrane Protein	COG1133;ABC-type long-chain fatty acid transport system, fused permease and ATPase components	GO:0042493 response to drug	o	tt
sbmC	CDS	DNA gyrase inhibitor	Cytoplasmic	COG3449;DNA gyrase inhibitor	GO:0006261 DNA dependent DNA replication -!- GO:0006281 DNA repair -!- GO:0009432 SOS response	gg	t
sbp	CDS	sulfate transporter subunit -!- periplasmic-binding component of ABC superfamily	Periplasmic	COG1613;ABC-type sulfate transport system, periplasmic component	GO:0006790 sulfur metabolism	o	tt
sdaA	CDS	L-serine deaminase I	Cytoplasmic	COG1760;L-serine deaminase	GO:0009063 amino acid catabolism	gg	t
sdaB	CDS	L-serine deaminase II	Cytoplasmic	COG1760;L-serine deaminase	GO:0019395 fatty acid oxidation -!- GO:0009063 amino acid catabolism	gg	t
sdaC	CDS	predicted serine transporter	Integral Membrane Protein	COG0814;Amino acid permeases	GO:0006564 L-serine biosynthesis	g	tt
sdhA	CDS	succinate dehydrogenase, flavoprotein subunit	Cytoplasmic	COG1053;Succinate dehydrogenase/fumarate reductase, flavoprotein subunit	GO:0006099 tricarboxylic acid cycle -!- GO:0009060 aerobic respiration	gg	t
sdhB	CDS	succinate dehydrogenase, FeS subunit	Cytoplasmic	COG0479;Succinate dehydrogenase/fumarate reductase, Fe-S protein subunit	GO:0006099 tricarboxylic acid cycle -!- GO:0009060 aerobic respiration	gg	t
sdhC	CDS	succinate dehydrogenase, membrane subunit, binds cytochrome b556	Integral Membrane Protein	COG2009;Succinate dehydrogenase/fumarate reductase, cytochrome b subunit	GO:0006099 tricarboxylic acid cycle -!- GO:0009060 aerobic respiration -!- GO:0017004 cytochrome biogenesis	o	tt
sdhD	CDS	succinate dehydrogenase, membrane subunit, binds cytochrome b556	Integral Membrane Protein	COG2142;Succinate dehydrogenase, hydrophobic anchor subunit	GO:0006099 tricarboxylic acid cycle -!- GO:0009060 aerobic respiration	o	tt
sdiA	CDS	DNA-binding transcriptional activator	Cytoplasmic	COG2771;DNA-binding HTH domain-containing proteins	GO:0006350 transcription	gg	t
secA	CDS	preprotein translocase subunit, ATPase that targets protein precursors to the SecYE core translocon	Cytoplasmic	COG0653;Preprotein translocase subunit SecA (ATPase, RNA helicase)		o	tt
secB	CDS	protein export chaperone	Cytoplasmic	COG1952;Preprotein translocase subunit SecB	GO:0006457 protein folding	o	tt
secD	CDS	SecYEG protein translocase auxillary subunit	Integral Membrane Protein	COG0342;Preprotein translocase subunit SecD		o	tt
secE	CDS	preprotein translocase membrane subunit	Integral Membrane Protein	COG0690;Preprotein translocase subunit SecE		o	tt
secF	CDS	SecYEG protein translocase auxillary subunit	Integral Membrane Protein	COG0341;Preprotein translocase subunit SecF		o	tt
secG	CDS	preprotein translocase membrane subunit	Integral Membrane Protein	COG1314;Preprotein translocase subunit SecG		o	tt
secM	CDS	regulator of secA translation	Periplasmic				
secY	CDS	preprotein translocase membrane subunit	Integral Membrane Protein	COG0201;Preprotein translocase subunit SecY		o	tt
selA	CDS	selenocysteine synthase	Cytoplasmic	COG1921;Selenocysteine synthase [seryl-tRNASer selenium transferase]	GO:0008615 pyridoxine biosynthesis -!- GO:0009451 RNA modification	g	t
selB	CDS	selenocysteinyl-tRNA-specific translation factor	Cytoplasmic	COG3276;Selenocysteine-specific translation elongation factor	GO:0006412 protein biosynthesis	o	t
selC	tRNA	tRNA-Sec(UCA) (Selenocysteyl tRNA-UCA, converted from serine tRNA)					
selD	CDS	selenophosphate synthase	Cytoplasmic	COG0709;Selenophosphate synthase	GO:0006418 amino acid activation	gg	t
seqA	CDS	regulatory protein for replication initiation	Cytoplasmic	COG3057;Negative regulator of replication initiationR	GO:0006261 DNA dependent DNA replication	o	tt
serA	CDS	D-3-phosphoglycerate dehydrogenase	Cytoplasmic	COG0111;Phosphoglycerate dehydrogenase and related dehydrogenases	GO:0006564 L-serine biosynthesis	gg	t
serB	CDS	3-phosphoserine phosphatase	Cytoplasmic	COG0560;Phosphoserine phosphatase	GO:0006545 glycine biosynthesis -!- GO:0006564 L-serine biosynthesis	gg	t
serC	CDS	3-phosphoserine/phosphohydroxythreonine aminotransferase	Cytoplasmic	COG1932;Phosphoserine aminotransferase	GO:0006564 L-serine biosynthesis	gg	t
serS	CDS	seryl-tRNA synthetase, also charges selenocysteinyl-tRNA with serine	Cytoplasmic	COG0172;Seryl-tRNA synthetase	GO:0006418 amino acid activation	gg	tt
serT	tRNA	tRNA-Ser(UGA) (Serine tRNA1)					
serU	tRNA	tRNA-Ser(CGA) (Serine tRNA2)					
serV	tRNA	tRNA-Ser(GCU) (Serine tRNA3)					
serW	tRNA	tRNA-Ser(GGA) (Serine tRNA5)					
serX	tRNA	tRNA-Ser(GGA) (Serine tRNA5)					
setA	CDS	broad specificity sugar efflux system	Integral Membrane Protein		GO:0016052 carbohydrate	o	tt
setB	CDS	lactose/glucose efflux system	Integral Membrane Protein	COG0477;Permeases of the major facilitator superfamily	GO:0016052 carbohydrate	o	tt
setC	CDS	predicted sugar efflux system	Integral Membrane Protein	COG0477;Permeases of the major facilitator superfamily		o	tt
sfcA	CDS	malate dehydrogenase, NAD-requiring (malic	Cytoplasmic	COG0281;Malic enzyme	GO:0006094 gluconeogenesis		
sfmA	CDS	predicted fimbrial-like adhesin protein	Periplasmic			o	tt
sfmC	CDS	pilin chaperone, periplasmic	Periplasmic	COG3121;P pilus assembly protein, chaperone PapD	GO:0006457 protein folding	o	tt
sfmD	CDS	predicted outer membrane export usher protein	Outer Membrane B-barrel protein	COG3188;P pilus assembly protein, porin PapC		o	tt
sfmF	CDS	predicted fimbrial-like adhesin protein	Periplasmic	COG3539;P pilus assembly protein, pilin FimA			
sfmH	CDS	predicted fimbrial-like adhesin protein	Cytoplasmic	COG3539;P pilus assembly protein, pilin FimA	GO:0009296 flagella biogenesis -!- GO:0042330 taxis		
sfsA	CDS	predicted DNA-binding transcriptional regulator	Cytoplasmic	COG1489;DNA-binding protein, stimulates sugar fermentation	GO:0016052 carbohydrate	g	o
sfsB	CDS	DNA-binding transcriptional regulator	Cytoplasmic	COG3423;Predicted transcriptional regulator	GO:0016052 carbohydrate catabolism -!- GO:0006350	o	tt
sgbE	CDS	L-ribulose-5-phosphate 4-epimerase	Cytoplasmic	COG0235;Ribulose-5-phosphate 4-epimerase and related epimerases and aldolases	GO:0016052 carbohydrate catabolism	gg	o
sgbH	CDS	3-keto-L-gulonate 6-phosphate decarboxylase	Cytoplasmic	COG0269;3-hexulose-6-phosphate synthase and related proteins		gg	t
sgbU	CDS	predicted L-xylulose 5-phosphate 3-epimerase	Cytoplasmic	COG3623;Putative L-xylulose-5-phosphate 3-epimerase			
sgcA	CDS	KpLE2 phage-like element; predicted phosphotransferase enzyme IIA component	Cytoplasmic	COG1762;Phosphotransferase system mannitol/fructose-specific IIA domain (Ntr-type)		o	t
sgcB	CDS	predicted enzyme IIB component of PTS	Cytoplasmic				
sgcC	CDS	KpLE2 phage-like element; predicted phosphotransferase enzyme IIC component	Integral Membrane Protein	COG3775;Phosphotransferase system, galactitol-specific IIC component	GO:0009401 phosphoenolpyruvate-dependent sugar phosphotransferase system	o	tt
sgcE	CDS	KpLE2 phage-like element; predicted epimerase	Cytoplasmic	COG0036;Pentose-5-phosphate-3-epimerase		o	t
sgcQ	CDS	KpLE2 phage-like element; predicted nucleoside triphosphatase	Cytoplasmic	COG0434;Predicted TIM-barrel enzyme		o	o
sgcR	CDS	KpLE2 phage-like element; predicted DNA-binding transcriptional regulator	Cytoplasmic	COG1349;Transcriptional regulators of sugar metabolism	GO:0006350 transcription	g	t
sgcX	CDS	KpLE2 phage-like element; predicted endoglucanase with Zn-dependent exopeptidase domain	Cytoplasmic				
sgrR	CDS	DNA-binding transcriptional regulator	Cytoplasmic	COG4533;ABC-type uncharacterized transport system, periplasmic component		o	tt
sgrS	misc_RNA	small antisense RNA					
shiA	CDS	shikimate transporter	Integral Membrane Protein	COG0477;Permeases of the major facilitator superfamily	GO:0009423 chorismate biosynthesis	o	tt
sieB	CDS	Rac prophage; phage superinfection exclusion	Integral Membrane Protein				
sixA	CDS	phosphohistidine phosphatase	Cytoplasmic	COG2062;Phosphohistidine phosphatase SixA	GO:0006464 protein modification	g	t
slp	CDS	outer membrane lipoprotein	Outer Membrane Lipoprotein		GO:0042594 response to starvation	o	o
slt	CDS	lytic murein transglycosylase, soluble	Periplasmic		GO:0009252 peptidoglycan biosynthesis	o	tt
slyA	CDS	DNA-binding transcriptional activator	Cytoplasmic	COG1846;Transcriptional regulators	GO:0006350 transcription		
slyB	CDS	outer membrane lipoprotein	Outer Membrane Lipoprotein			o	tt
slyD	CDS	FKBP-type peptidyl prolyl cis-trans isomerase (rotamase)	Cytoplasmic	COG1047;FKBP-type peptidyl-prolyl cis-trans isomerases 2	GO:0006457 protein folding	gg	tt
slyX	CDS	conserved protein	Cytoplasmic	COG2900;Uncharacterized protein conserved in bacteria		gg	o
smf	CDS	conserved protein	Cytoplasmic				
smg	CDS	conserved protein	Cytoplasmic	COG2922;Uncharacterized protein conserved in bacteria		gg	tt
smpA	CDS	small membrane lipoprotein	Outer Membrane Lipoprotein	COG2913;Small protein A (tmRNA-binding)		o	tt
smpB	CDS	trans-translation protein	Cytoplasmic	COG0691;tmRNA-binding protein		gg	tt
smtA	CDS	S-adenosylmethionine-dependent methyltransferase	Cytoplasmic	COG0500;SAM-dependent methyltransferases	GO:0006071 glycerol metabolism	gg	t
sodA	CDS	superoxide dismutase, Mn	Cytoplasmic		GO:0006805 xenobiotic metabolism	gg	o
sodB	CDS	superoxide dismutase, Fe	Cytoplasmic	COG0605;Superoxide dismutase	GO:0006805 xenobiotic metabolism	gg	o
sodC	CDS	superoxide dismutase, Cu, Zn	Periplasmic	COG2032;Cu/Zn superoxide dismutase	GO:0006805 xenobiotic metabolism	o	tt
sohA	CDS	predicted regulator	Cytoplasmic	COG2002;Regulators of stationary/sporulation gene expression		gg	tt
sohB	CDS	predicted inner membrane peptidase	Integral Membrane Protein	COG0616;Periplasmic serine proteases (ClpP class)		o	tt
sokB	misc_RNA	regulatory, antisense sRNA					
sokC	misc_RNA	regulatory, antisense RNA					
solA	CDS	N-methyltryptophan oxidase, FAD-binding	Cytoplasmic	COG0665;Glycine/D-amino acid oxidases (deaminating)		gg	t
soxR	CDS	DNA-binding transcriptional dual regulator, Fe-S center for redox-sensing	Cytoplasmic	COG0789;Predicted transcriptional regulators	GO:0006350 transcription -!- GO:0006805 xenobiotic metabolism	o	tt
soxS	CDS	DNA-binding transcriptional dual regulator	Cytoplasmic	COG2207;AraC-type DNA-binding domain-containing proteins	GO:0006350 transcription -!- GO:0006805 xenobiotic metabolism	o	tt
speA	CDS	biosynthetic arginine decarboxylase, PLP-binding	Cytoplasmic	COG1166;Arginine decarboxylase (spermidine biosynthesis)	GO:0006596 polyamine biosynthesis	o	tt
speB	CDS	agmatinase	Cytoplasmic	COG0010;Arginase/agmatinase/formimionoglutamate hydrolase, arginase family	GO:0006596 polyamine biosynthesis	gg	t
speC	CDS	ornithine decarboxylase, constitutive	Cytoplasmic		GO:0006596 polyamine biosynthesis		
speD	CDS	S-adenosylmethionine decarboxylase	Cytoplasmic	COG1586;S-adenosylmethionine decarboxylase	GO:0006596 polyamine biosynthesis	gg	o
speE	CDS	spermidine synthase (putrescine aminopropyltransferase)	Cytoplasmic	COG0421;Spermidine synthase	GO:0006596 polyamine biosynthesis	gg	o
speF	CDS	ornithine decarboxylase isozyme, inducible	Cytoplasmic	COG1982;Arginine/lysine/ornithine decarboxylases	GO:0006596 polyamine biosynthesis	gg	tt
speG	CDS	spermidine N1-acetyltransferase	Cytoplasmic	COG1670;Acetyltransferases, including N-acetylases of ribosomal proteins	GO:0006596 polyamine biosynthesis	gg	t

spf	misc_RNA	Spot 42 RNA					
spoT	CDS	bifunctional (p)ppGpp synthetase II -!- guanosine-3',5'-bis pyrophosphate 3'-pyrophosphohydrolase	Cytoplasmic	COG0317;Guanosine polyphosphate pyrophosphohydrolases/synthetases	GO:0015949 nucleobase, nucleoside and nucleotide interconversion -!- GO:0042594 response to starvation	o	tt
sppA	CDS	protease IV (signal peptide peptidase)	Membrane Anchored	COG0616;Periplasmic serine proteases (ClpP class)		o	tt
spr	CDS	predicted peptidase, outer membrane lipoprotein	Outer Membrane Lipoprotein	COG0791;Cell wall-associated hydrolases (invasion-associated proteins)		gg	t
sprT	CDS	conserved protein	Cytoplasmic	COG3091;Uncharacterized protein conserved in bacteria		o	tt
spy	CDS	envelope stress induced periplasmic protein	Periplasmic	COG3678;P pilus assembly/Cpx signaling pathway, periplasmic inhibitor/zinc-resistance associated protein		o	tt
sra	CDS	30S ribosomal subunit protein S22	Cytoplasmic		GO:0006412 protein biosynthesis		
sraB	misc_RNA	small RNA					
sraD	misc_RNA	small RNA					
sraF	misc_RNA	small RNA					
sraG	misc_RNA	small RNA					
srlA	CDS	glucitol/sorbitol-specific enzyme IIC component of	Integral Membrane Protein		GO:0016052 carbohydrate		
srlB	CDS	glucitol/sorbitol-specific enzyme IIA component of	Cytoplasmic	COG3731;Phosphotransferase system sorbitol-specific component IIA	GO:0016052 carbohydrate	gg	t
srlD	CDS	sorbitol-6-phosphate dehydrogenase	Outer Membrane Lipoprotein	COG1028;Dehydrogenases with different specificities (related to short-chain alcohol dehydrogenases)	GO:0016052 carbohydrate catabolism	gg	t
srlE	CDS	glucitol/sorbitol-specific enzyme IIB component of	Integral Membrane Protein		GO:0016052 carbohydrate		
srlR	CDS	DNA-bindng transcriptional repressor	Cytoplasmic	COG1349;Transcriptional regulators of sugar metabolism	GO:0016052 carbohydrate catabolism -!- GO:0006350 transcription -!- GO:0006355 regulation of transcription, DNA-	o	tt
srmB	CDS	ATP-dependent RNA helicase	Cytoplasmic	COG0513;Superfamily II DNA and RNA helicases	GO:0009451 RNA modification	gg	tt
sroF	misc_RNA	small RNA					
ssb	CDS	Single-stranded DNA-binding protein	Cytoplasmic	COG0629;Single-stranded DNA-binding protein	GO:0006310 DNA recombination -!- GO:0009432 SOS response	o	tt
sseA	CDS	3-mercaptopyruvate sulfurtransferase	Cytoplasmic		GO:0006790 sulfur metabolism	o	o
sseB	CDS	rhodanase-like enzyme, sulfur transfer from	Cytoplasmic		GO:0006790 sulfur metabolism		
ssnA	CDS	predicted chlorohydrolase/aminohydrolase	Cytoplasmic		GO:0007049 cell cycle	o	tt
sspA	CDS	stringent starvation protein A	Cytoplasmic	COG0625;Glutathione S-transferase	GO:0006350 transcription -!- GO:0006355 regulation of transcription, DNA-dependent -!- GO:0042594 response to starvation	gg	t
sspB	CDS	ClpXP protease specificity-enhancing factor	Cytoplasmic	COG2969;Stringent starvation protein B	GO:0042594 response to starvation	gg	t
ssrA	misc_RNA	10Sa RNA SsrA (tmRNA)					
ssrS	misc_RNA	6S regulatory RNA					
sstT	CDS	sodium:serine/threonine symporter	Integral Membrane Protein	COG3633;Na+/serine symporter	GO:0006564 L-serine biosynthesis -!- GO:0009088 threonine biosynthesis	o	tt
ssuA	CDS	alkanesulfonate transporter subunit -!- periplasmic-binding component of ABC superfamily	Periplasmic		GO:0006793 phosphorus metabolism	o	tt
ssuB	CDS	alkanesulfonate transporter subunit -!- ATP-binding component of ABC superfamily	Cytoplasmic	COG1116;ABC-type nitrate/sulfonate/bicarbonate transport system, ATPase component	GO:0006790 sulfur metabolism	gg	t
ssuC	CDS	alkanesulfonate transporter subunit -!- membrane component of ABC superfamily	Integral Membrane Protein		GO:0006793 phosphorus metabolism		
ssuD	CDS	alkanesulfonate monooxygenase, FMNH(2)-dependent	Cytoplasmic	COG2141;Coenzyme F420-dependent N5,N10-methylene tetrahydromethanopterin reductase and related flavin-dependent oxidoreductases	GO:0006790 sulfur metabolism	gg	t
ssuE	CDS	NAD(P)H-dependent FMN reductase	Cytoplasmic	COG0431;Predicted flavoprotein	GO:0006790 sulfur metabolism -!- GO:0042594 response to starvation	gg	t
stfE	CDS	e14 prophage; predicted side tail fiber protein fragment (pseudogene)	Cytoplasmic				
stfQ	CDS	Qin prophage; predicted side tail fibre assembly protein	Cytoplasmic			gg	o
stfR	CDS	Rac prophage; predicted tail fiber protein	Cytoplasmic			o	tt
sthA	CDS	pyridine nucleotide transhydrogenase, soluble	Cytoplasmic	COG1249;Pyruvate/2-oxoglutarate dehydrogenase complex, dihydrolipoamide dehydrogenase (E3) component, and related enzymes	GO:0009226 nucleotide-sugar biosynthesis		
stpA	CDS	DNA binding protein, nucleoid-associated	Cytoplasmic	COG2916;DNA-binding protein H-NS	GO:0006457 protein folding	o	tt
sucA	CDS	2-oxoglutarate decarboxylase, thiamin-requiring	Cytoplasmic	COG0567;2-oxoglutarate dehydrogenase complex, dehydrogenase (E1) component, and related enzymes	GO:0006099 tricarboxylic acid cycle	gg	t
sucB	CDS	dihydrolipoyltranssuccinase	Cytoplasmic	COG0508;Pyruvate/2-oxoglutarate dehydrogenase complex, dihydrolipoamide acyltransferase (E2) component, and related enzymes	GO:0006099 tricarboxylic acid cycle	g	t
sucC	CDS	succinyl-CoA synthetase, beta subunit	Cytoplasmic	COG0045;Succinyl-CoA synthetase, beta subunit	GO:0006099 tricarboxylic acid cycle	gg	t
sucD	CDS	succinyl-CoA synthetase, NAD(P)-binding, alpha subunit	Cytoplasmic	COG0074;Succinyl-CoA synthetase, alpha subunit	GO:0006099 tricarboxylic acid cycle	g	t
sufA	CDS	Fe-S cluster assembly protein	Cytoplasmic	COG0316;Uncharacterized conserved protein		gg	o
sufB	CDS	component of SufBCD complex	Cytoplasmic	COG0719;ABC-type transport system involved in Fe-S cluster assembly, permease component	GO:0006457 protein folding		
sufC	CDS	component of SufBCD complex, ATP-binding component of ABC superfamily	Cytoplasmic	COG0396;ABC-type transport system involved in Fe-S cluster assembly, ATPase component		gg	tt
sufD	CDS	component of SufBCD complex	Cytoplasmic	COG0719;ABC-type transport system involved in Fe-S cluster assembly, permease component		gg	t
sufE	CDS	sulfur acceptor protein	Cytoplasmic	COG2166;SufE protein probably involved in Fe-S center assembly		gg	o
sufI	CDS	repressor protein for FtsI	Periplasmic	COG2132;Putative multicopper oxidases		o	tt
sufS	CDS	selenocysteine lyase, PLP-dependent	Cytoplasmic	COG0520;Selenocysteine lyase		gg	t
sugE	CDS	multidrug efflux system protein	Integral Membrane Protein				
suhB	CDS	inositol monophosphatase	Cytoplasmic	COG0483;Archaeal fructose-1,6-bisphosphatase and related enzymes of inositol monophosphatase family	GO:0006350 transcription	gg	t
sulA	CDS	SOS cell division inhibitor	Cytoplasmic	COG5404;SOS-response cell division inhibitor, blocks FtsZ ring formation	GO:0006281 DNA repair -!- GO:0009432 SOS response	o	tt
surA	CDS	peptidyl-prolyl cis-trans isomerase (PPIase)	Periplasmic	COG0760;Parvulin-like peptidyl-prolyl isomerase	GO:0006457 protein folding	o	tt
surE	CDS	broad specificity 5'(3')-nucleotidase and polyphosphatase	Cytoplasmic	COG0496;Predicted acid phosphatase	GO:0006950 response to stress	gg	t
syd	CDS	predicted protein	Cytoplasmic			gg	t
tadA	CDS	tRNA-specific adenosine deaminase	Cytoplasmic	COG0590;Cytosine/adenosine deaminases	GO:0009451 RNA modification	o	t
tag	CDS	3-methyl-adenine DNA glycosylase I, constitutive	Cytoplasmic	COG2818;3-methyladenine DNA glycosylase	GO:0006281 DNA repair	g	t
talA	CDS	transaldolase A	Cytoplasmic	COG0176;Transaldolase	GO:0009052 pentose-phosphate shunt, non-oxidative branch	gg	t
talB	CDS	transaldolase B	Cytoplasmic	COG0176;Transaldolase	GO:0009052 pentose-phosphate shunt, non-oxidative branch	gg	o
tam	CDS	trans-aconitate methyltransferase	Cytoplasmic	COG4106;Trans-aconitate methyltransferase		gg	o
tap	CDS	methyl-accepting protein IV	Integral Membrane Protein	COG0840;Methyl-accepting chemotaxis protein	GO:0042330 taxis	o	tt
tar	CDS	methyl-accepting chemotaxis protein II	Integral Membrane Protein	COG0840;Methyl-accepting chemotaxis protein	GO:0042330 taxis	o	tt
tas	CDS	predicted oxidoreductase, NADP(H)-dependent aldo-keto reductase	Cytoplasmic	COG0667;Predicted oxidoreductases (related to aryl-alcohol dehydrogenases)		g	t
tatA	CDS	TatABCE protein translocation system subunit	Membrane Anchored			o	tt
tatB	CDS	TatABCE protein translocation system subunit	Membrane Anchored				
tatC	CDS	TatABCE protein translocation system subunit	Integral Membrane Protein	COG0805;Sec-independent protein secretion pathway component TatC		o	tt
tatD	CDS	DNase, magnesium-dependent	Cytoplasmic				
tatE	CDS	TatABCE protein translocation system subunit	Membrane Anchored	COG1826;Sec-independent protein secretion pathway components		o	t
tauA	CDS	taurine transporter subunit -!- periplasmic-binding component of ABC superfamily	Periplasmic	COG4521;ABC-type taurine transport system, periplasmic component	GO:0006790 sulfur metabolism -!- GO:0009310 amine catabolism	o	tt
tauB	CDS	taurine transporter subunit -!- ATP-binding component of ABC superfamily	Cytoplasmic	COG4525;ABC-type taurine transport system, ATPase component	GO:0006790 sulfur metabolism -!- GO:0009310 amine catabolism	gg	t
tauC	CDS	taurine transporter subunit -!- membrane component of ABC superfamily	Integral Membrane Protein	COG0600;ABC-type nitrate/sulfonate/bicarbonate transport system, permease component	GO:0006790 sulfur metabolism -!- GO:0009310 amine catabolism	o	tt
tauD	CDS	taurine dioxygenase, 2-oxoglutarate-dependent	Cytoplasmic	COG2175;Probable taurine catabolism dioxygenase	GO:0009310 amine catabolism -!- GO:0006790 sulfur metabolism	gg	t
tbpA	CDS	thiamin transporter subunit -!- periplasmic-binding component of ABC superfamily	Periplasmic	COG4143;ABC-type thiamine transport system, periplasmic component	GO:0009228 thiamin biosynthesis	o	tt
tdcA	CDS	DNA-binding transcriptional activator	Cytoplasmic	COG0583;Transcriptional regulator	GO:0009063 amino acid catabolism -!- GO:0006350 transcription	g	o
tdcB	CDS	catabolic threonine dehydratase, PLP-dependent	Cytoplasmic	COG1171;Threonine dehydratase	GO:0009063 amino acid catabolism	gg	t
tdcC	CDS	L-threonine/L-serine transporter	Integral Membrane Protein	COG0814;Amino acid permeases	GO:0009088 threonine biosynthesis	o	tt
tdcD	CDS	propionate kinase/acetate kinase C, anaerobic	Cytoplasmic	COG0282;Acetate kinase	GO:0009063 amino acid catabolism		
tdcE	CDS	pyruvate formate-lyase 4/2-ketobutyrate formate-lyase	Cytoplasmic		GO:0009063 amino acid catabolism -!- GO:0006113 fermentation -!- GO:0009061 anaerobic respiration		
tdcF	CDS	predicted L-PSP (mRNA) endoribonuclease	Cytoplasmic				
tdcG	CDS	L-serine dehydratase 3	Cytoplasmic		GO:0009063 amino acid catabolism		
tdcR	CDS	DNA-binding transcriptional activator	Cytoplasmic		GO:0009063 amino acid catabolism -!- GO:0006350 transcription		
tdh	CDS	threonine 3-dehydrogenase, NAD(P)-binding	Cytoplasmic	COG1063;Threonine dehydrogenase and related Zn-dependent dehydrogenases	GO:0009063 amino acid catabolism	g	t
tdk	CDS	thymidine kinase/deoxyuridine kinase	Cytoplasmic	COG1435;Thymidine kinase	GO:0015949 nucleobase, nucleoside and nucleotide interconversion	gg	t
tehA	CDS	potassium-tellurite ethidium and proflavin transporter	Integral Membrane Protein	COG1275;Tellurite resistance protein and related permeases	GO:0042493 response to drug	o	tt
tehB	CDS	predicted S-adenosyl-L-methionine-dependent methyltransferase	Cytoplasmic	COG0500;SAM-dependent methyltransferases	GO:0042493 response to drug	gg	t
tesA	CDS	multifunctional acyl-CoA thioesterase I -!- protease I -!- lysophospholipase L1	Periplasmic	COG2755;Lysophospholipase L1 and related esterases		o	tt
tesB	CDS	acyl-CoA thioesterase II	Cytoplasmic	COG1946;Acyl-CoA thioesterase		gg	o

tfaD	CDS	DLP12 prophage; predicted tail fiber assembly protein (pseudogene)	Cytoplasmic				
tfaE	CDS	e14 prophage; predicted tail fiber assembly protein	Cytoplasmic			gg	t
tfaQ	CDS	Qin prophage; predicted tail fibre assembly protein	Cytoplasmic			gg	o
tfaR	CDS	Rac prophage; predicted tail fiber assembly protein	Cytoplasmic			gg	o
tfaS	CDS	CPS-53 (KpLE1) prophage; tail fiber assembly protein fragment (pseudogene)	Cytoplasmic				
tff	misc_RNA	small RNA					
tgt	CDS	tRNA-guanine transglycosylase	Cytoplasmic	COG0343;Queuine/archaeosine tRNA-ribosyltransferase	GO:0009451 RNA modification	gg	t
thiC	CDS	thiamin (pyrimidine moiety) biosynthesis protein	Cytoplasmic	COG0422;Thiamine biosynthesis protein ThiC	GO:0009228 thiamin biosynthesis	gg	t
thiD	CDS	bifunctional hydroxy-methylpyrimidine kinase -!- hydroxy-phosphomethylpyrimidine kinase	Cytoplasmic	COG0351;Hydroxymethylpyrimidine/phosphomethylpyrim idine kinase	GO:0009228 thiamin biosynthesis	g	t
thiE	CDS	thiamin phosphate synthase (thiamin phosphate pyrophosphorylase)	Cytoplasmic	COG0352;Thiamine monophosphate synthase	GO:0009228 thiamin biosynthesis	gg	t
thiF	CDS	thiamin (thiazole moiety) biosynthesis protein	Membrane Anchored		GO:0009228 thiamin biosynthesis	gg	o
thiG	CDS	thiamin biosynthesis ThiGH complex subunit	Cytoplasmic		GO:0009228 thiamin biosynthesis		
thiH	CDS	thiamin biosynthesis ThiGH complex subunit	Cytoplasmic	COG1060;Thiamine biosynthesis enzyme ThiH and related uncharacterized enzymes	GO:0009228 thiamin biosynthesis	gg	t
thiI	CDS	sulfurtransferase required for thiamine and 4-thiouridine biosynthesis	Cytoplasmic	COG0301;Thiamine biosynthesis ATP pyrophosphatase -!- COG0607;Rhodanese-related sulfurtransferase	GO:0009228 thiamin biosynthesis -!- GO:0009451 RNA modification	gg	tt
thiL	CDS	thiamin-monophosphate kinase	Cytoplasmic	COG0611;Thiamine monophosphate kinase	GO:0009228 thiamin biosynthesis	gg	o
thiM	CDS	hydoxyethylthiazole kinase	Cytoplasmic	COG2145;Hydroxyethylthiazole kinase, sugar kinase family	GO:0009228 thiamin biosynthesis	gg	o
thiP	CDS	fused subunits of thiamin transporter -!- membrane components of ABC superfamily	Integral Membrane Protein	COG1178;ABC-type Fe3+ transport system, permease component	GO:0009228 thiamin biosynthesis	o	tt
thiQ	CDS	thiamin transporter subunit -!- ATP-binding component of ABC superfamily	Cytoplasmic	COG3840;ABC-type thiamine transport system, ATPase component	GO:0009228 thiamin biosynthesis	o	tt
thiS	CDS	sulphur carrier protein	Cytoplasmic			gg	t
thrA	CDS	fused aspartokinase I -!- homoserine dehydrogenase I	Cytoplasmic	COG0527;Aspartokinases -!- COG0460;Homoserine dehydrogenase	GO:0009088 threonine biosynthesis -!- GO:0009086 methionine biosynthesis -!- GO:0009090 homoserine biosynthesis		t
thrB	CDS	homoserine kinase	Cytoplasmic	COG0083;Homoserine kinase	GO:0009088 threonine biosynthesis	g	o
thrC	CDS	threonine synthase	Cytoplasmic	COG0498;Threonine synthase	GO:0009088 threonine biosynthesis	gg	t
thrL	CDS	thr operon leader peptide	Cytoplasmic		GO:0009088 threonine biosynthesis		
thrS	CDS	threonyl-tRNA synthetase	Cytoplasmic	COG0441;Threonyl-tRNA synthetase	GO:0006418 amino acid activation	gg	t
thrT	tRNA	tRNA-Thr(GGU) (Threonine tRNA3)					
thrU	tRNA	tRNA-Thr(UGU) (Threonine tRNA4)					
thrV	tRNA	tRNA-Thr(GGU) (Threonine tRNA1)					
thrW	tRNA	tRNA-Thr(CGU) (Threonine tRNA2)					
thyA	CDS	thymidylate synthetase	Cytoplasmic	COG0207;Thymidylate synthase	GO:0015949 nucleobase, nucleoside and nucleotide interconversion -!- GO:0009257 10-formyltetrahydrofolate biosynthesis	gg	t
tiaE	CDS	2-keto-D-gluconate reductase (glyoxalate reductase) (2-ketoaldonate reductase)	Cytoplasmic	COG1052;Lactate dehydrogenase and related dehydrogenases	GO:0016052 carbohydrate catabolism		
tig	CDS	peptidyl-prolyl cis/trans isomerase (trigger factor)	Cytoplasmic	COG0544;FKBP-type peptidyl-prolyl cis-trans isomerase (trigger factor)	GO:0006457 protein folding	o	tt
tilS	CDS	tRNA(Ile)-lysidine synthetase	Cytoplasmic	COG0037;Predicted ATPase of the PP-loop superfamily implicated in cell cycle control		o	tt
tktA	CDS	transketolase 1, thiamin-binding	Cytoplasmic		GO:0016052 carbohydrate catabolism -!- GO:0009052 pentose-phosphate shunt, non-oxidative branch -!- GO:0015949 nucleobase, nucleoside and nucleotide		
tktB	CDS	transketolase 2, thiamin-binding	Cytoplasmic	COG0021;Transketolase	GO:0016052 carbohydrate catabolism -!- GO:0009052 pentose-phosphate shunt, non-oxidative branch -!- GO:0015949 nucleobase, nucleoside and nucleotide	gg	t
tldD	CDS	predicted peptidase	Cytoplasmic	COG0312;Predicted Zn-dependent proteases and their inactivated		o	tt
tmk	CDS	thymidylate kinase	Cytoplasmic	COG0125;Thymidylate kinase	GO:0015949 nucleobase, nucleoside and nucleotide interconversion	gg	o
tnaA	CDS	tryptophanase/L-cysteine desulfhydrase, PLP-dependent	Cytoplasmic		GO:0009063 amino acid catabolism	gg	o
tnaB	CDS	tryptophan transporter of low affinity	Integral Membrane Protein	COG0814;Amino acid permeases	GO:0000162 tryptophan biosynthesis		
tnaB	CDS	tryptophan transporter of low affinity	Integral Membrane Protein				
tnaB	CDS	tryptophan transporter of low affinity	Integral Membrane Protein				
tnaC	CDS	tryptophanase leader peptide	Cytoplasmic		GO:0009063 amino acid catabolism	gg	o
tolA	CDS	membrane anchored protein in TolA-TolQ-TolR complex	Membrane Anchored	COG3064;Membrane protein involved in colicin uptake		o	tt
tolB	CDS	periplasmic protein	Periplasmic	COG0823;Periplasmic component of the Tol biopolymer transport system			
tolC	CDS	transport channel	Outer Membrane B-barrel protein	COG1538;Outer membrane protein			
tolQ	CDS	membrane spanning protein in TolA-TolQ-TolR complex	Integral Membrane Protein	COG0811;Biopolymer transport proteins		o	tt
tolR	CDS	membrane spanning protein in TolA-TolQ-TolR complex	Membrane Anchored	COG0848;Biopolymer transport protein		o	tt
tonB	CDS	membrane spanning protein in TonB-ExbB-ExbD complex	Membrane Anchored	COG0810;Periplasmic protein TonB, links inner and outer membranes			
topA	CDS	DNA topoisomerase I, omega subunit	Cytoplasmic	COG0550;Topoisomerase IA -!- COG0551;Zn-finger domain associated with topoisomerase type I	GO:0006261 DNA dependent DNA replication -!- GO:0006350 transcription	g	tt
topB	CDS	DNA topoisomerase III	Cytoplasmic	COG0550;Topoisomerase IA	GO:0006261 DNA dependent DNA replication -!- GO:0006350 transcription	o	tt
torA	CDS	trimethylamine N-oxide (TMAO) reductase I, catalytic subunit	Periplasmic	COG0243;Anaerobic dehydrogenases, typically selenocysteine-containing	GO:0009061 anaerobic respiration	gg	tt
torC	CDS	trimethylamine N-oxide (TMAO) reductase I, cytochrome c-type subunit	Periplasmic with N-terminal Membrane Anchor	COG3005;Nitrate/TMAO reductases, membrane-bound tetraheme cytochrome c subunit	GO:0009061 anaerobic respiration -!- GO:0017004 cytochrome biogenesis -!- GO:0006350 transcription	o	tt
torD	CDS	chaperone involved in maturation of TorA subunit of trimethylamine N-oxide reductase system I	Cytoplasmic	COG3381;Uncharacterized component of anaerobic dehydrogenases	GO:0006457 protein folding	gg	t
torI	CDS	response regulator inhibitor for tor operon					
torR	CDS	DNA-binding response regulator in two-component regulatory system with TorS	Cytoplasmic	COG0745;Response regulators consisting of a CheY-like receiver domain and a winged-helix DNA-binding domain	GO:0009061 anaerobic respiration -!- GO:0006350 transcription	gg	o
torS	CDS	hybrid sensory histidine kinase in two-component regulatory system with TorR	Integral Membrane Protein	COG0642;Signal transduction histidine kinase -!- COG2198;FOG: HPt domain	GO:0009061 anaerobic respiration -!- GO:0006464 protein modification		
torT	CDS	periplasmic sensory protein associated with the TorRS two-component regulatory system	Periplasmic	COG1879;ABC-type sugar transport system, periplasmic component	GO:0009061 anaerobic respiration -!- GO:0006464 protein modification	o	tt
torY	CDS	TMAO reductase III (TorYZ), cytochrome c-type subunit	Periplasmic with N-terminal Membrane	COG3005;Nitrate/TMAO reductases, membrane-bound tetraheme cytochrome c subunit		o	tt
torZ	CDS	trimethylamine N-oxide reductase system III, catalytic subunit	Periplasmic			gg	tt
tpiA	CDS	triosephosphate isomerase	Cytoplasmic	COG0149;Triosephosphate isomerase	GO:0006096 glycolysis	gg	t
tpr	CDS	predicted protamine-like protein	Cytoplasmic			gg	t
tpx	CDS	lipid hydroperoxide peroxidase	Periplasmic	COG2077;Peroxiredoxin	GO:0006805 xenobiotic metabolism	gg	o
treA	CDS	periplasmic trehalase	Periplasmic	COG1626;Neutral trehalase	GO:0006006 glucose metabolism -!- GO:0006970 response to osmotic stress	o	tt
treB	CDS	fused trehalose(maltose)-specific enzyme IIBC component of PTS	Integral Membrane Protein	COG1264;Phosphotransferase system IIB components -!- COG1263;Phosphotransferase system IIC components, glucose/maltose/N-acetylglucosamine-specific	GO:0006006 glucose metabolism	o	tt
treC	CDS	trehalose-6-P hydrolase	Cytoplasmic	COG0366;Glycosidases	GO:0016052 carbohydrate	o	tt
treF	CDS	cytoplasmic trehalase	Cytoplasmic	COG1626;Neutral trehalase	GO:0016052 carbohydrate	o	tt
treR	CDS	DNA-binding transcriptional repressor	Cytoplasmic	COG1609;Transcriptional regulators	GO:0006006 glucose metabolism -!- GO:0006350 transcription -!- GO:0006970 response to osmotic stress	o	tt
trg	CDS	methyl-accepting chemotaxis protein III, ribose and galactose sensor receptor	Integral Membrane Protein	COG0840;Methyl-accepting chemotaxis protein	GO:0042330 taxis	o	tt
trkA	CDS	NAD-binding component of TrK potassium	Cytoplasmic	COG0569;K+ transport systems, NAD-binding component		g	t
trkD	CDS	potassium transporter	Integral Membrane Protein		GO:0006970 response to osmotic stress		
trkG	CDS	Rac prophage; potassium transporter subunit	Integral Membrane Protein	COG0168;Trk-type K+ transport systems, membrane components		o	tt
trkH	CDS	potassium transporter	Integral Membrane Protein				
trmA	CDS	tRNA (uracil-5-)-methyltransferase	Cytoplasmic	COG2265;SAM-dependent methyltransferases related to tRNA (uracil-5-)-methyltransferase	GO:0009451 RNA modification	gg	tt
trmC	CDS	fused 5-methylaminomethyl-2-thiouridine forming enzyme methyltransferase -!- FAD-dependent demodification enzyme	Cytoplasmic				
trmD	CDS	tRNA (guanine-1-)-methyltransferase	Cytoplasmic	COG0336;tRNA-(guanine-N1)-methyltransferase	GO:0009451 RNA modification	gg	o
trmE	CDS	GTPase	Cytoplasmic	COG0486;Predicted GTPase	GO:0009451 RNA modification -!- GO:0006805 xenobiotic metabolism	gg	tt
trmH	CDS	tRNA (Guanosine-2'-O-)-methyltransferase	Cytoplasmic	COG0566;rRNA methylases	GO:0009451 RNA modification	gg	t
trmU	CDS	tRNA (5-methylaminomethyl-2-thiouridylate)-methyltransferase	Cytoplasmic		GO:0009451 RNA modification	o	tt
trpA	CDS	tryptophan synthase, alpha subunit	Cytoplasmic	COG0159;Tryptophan synthase alpha chain	GO:0000162 tryptophan biosynthesis	gg	o
trpB	CDS	tryptophan synthase, beta subunit	Cytoplasmic	COG0133;Tryptophan synthase beta chain	GO:0000162 tryptophan biosynthesis	gg	tt
trpC	CDS	fused indole-3-glycerolphosphate synthetase -!- N-(5-phosphoribosyl)anthranilate isomerase	Cytoplasmic	COG0134;Indole-3-glycerol phosphate synthase -!- COG0135;Phosphoribosylanthranilate isomerase	GO:0000162 tryptophan biosynthesis	o	tt

trpD	CDS	fused glutamine amidotransferase (component II) of anthranilate synthase -!- anthranilate phosphoribosyl transferase	Cytoplasmic	COG0512;Anthranilate/para-aminobenzoate synthases component II -!- COG0547;Anthranilate phosphoribosyltransferase	GO:0000162 tryptophan biosynthesis	gg	t
trpE	CDS	component I of anthranilate synthase	Cytoplasmic	COG0147;Anthranilate/para-aminobenzoate synthases component I	GO:0000162 tryptophan biosynthesis	o	tt
trpL	CDS	trp operon leader peptide	Cytoplasmic		GO:0000162 tryptophan biosynthesis	gg	o
trpR	CDS	DNA-binding transcriptional repressor, tryptophan-binding	Cytoplasmic	COG2973;Trp operon repressor	GO:0000162 tryptophan biosynthesis -!- GO:0006350 transcription	o	tt
trpS	CDS	tryptophanyl-tRNA synthetase	Cytoplasmic	COG0180;Tryptophanyl-tRNA synthetase	GO:0006418 amino acid activation	gg	tt
trpT	tRNA	tRNA-Trp(CCA) (Tryptophan tRNA)					
truA	CDS	pseudouridylate synthase I	Cytoplasmic	COG0101;Pseudouridylate synthase	GO:0006418 amino acid activation	o	tt
truB	CDS	tRNA pseudouridine synthase	Cytoplasmic	COG0130;Pseudouridine synthase	GO:0009451 RNA modification	gg	tt
truD	CDS	pseudoruidine synthase	Cytoplasmic	COG0585;Uncharacterized conserved protein		gg	o
trxA	CDS	thioredoxin 1	Cytoplasmic	COG0526;Thiol-disulfide isomerase and thioredoxins			
trxB	CDS	thioredoxin reductase, FAD/NAD(P)-binding	Cytoplasmic	COG0492;Thioredoxin reductase		gg	t
trxC	CDS	thioredoxin 2	Cytoplasmic	COG0526;Thiol-disulfide isomerase and thioredoxins		gg	tt
tsf	CDS	protein chain elongation factor EF-Ts	Cytoplasmic	COG0264;Translation elongation factor Ts	GO:0006412 protein biosynthesis	gg	o
tsgA	CDS	predicted transporter	Integral Membrane Protein	COG0477;Permeases of the major facilitator superfamily		o	tt
tsr	CDS	methyl-accepting chemotaxis protein I, serine sensor receptor	Integral Membrane Protein	COG0840;Methyl-accepting chemotaxis protein	GO:0042330 taxis	o	tt
tsx	CDS	nucleoside channel, receptor of phage T6 and colicin K	Outer Membrane B-barrel protein	COG3248;Nucleoside-binding outer membrane protein	GO:0015949 nucleobase, nucleoside and nucleotide interconversion	o	tt
ttdA	CDS	L-tartrate dehydratase, alpha subunit	Cytoplasmic	COG1951;Tartrate dehydratase alpha subunit/Fumarate hydratase class I, N-terminal domain	GO:0006113 fermentation	g	t
ttdB	CDS	L-tartrate dehydratase, beta subunit	Cytoplasmic	COG1838;Tartrate dehydratase beta subunit/Fumarate hydratase class I, C-terminal domain	GO:0006113 fermentation	o	t
ttk	CDS	division inhibitor	Cytoplasmic		GO:0006350 transcription		
tufA	CDS	protein chain elongation factor EF-Tu (duplicate of tufB)	Cytoplasmic	COG0050;GTPases - translation elongation factors	GO:0006412 protein biosynthesis -!- GO:0006970 response to osmotic stress	o	t
tufB	CDS	protein chain elongation factor EF-Tu (duplicate of tufA)	Cytoplasmic	COG0050;GTPases - translation elongation factors	GO:0006412 protein biosynthesis -!- GO:0006970 response to osmotic stress	o	tt
tus	CDS	inhibitor of replication at Ter, DNA-binding protein	Cytoplasmic		GO:0006261 DNA dependent DNA replication	gg	t
tynA	CDS	tyramine oxidase, copper-requiring	Periplasmic	COG3733;Cu2+-containing amine oxidase	GO:0009063 amino acid catabolism -!- GO:0009310 amine catabolism	o	tt
tyrA	CDS	fused chorismate mutase T -!- prephenate dehydrogenase	Cytoplasmic	COG1605;Chorismate mutase -!- COG0287;Prephenate dehydrogenase	GO:0009094 L-phenylalanine biosynthesis -!- GO:0006571 tyrosine biosynthesis	gg	o
tyrB	CDS	tyrosine aminotransferase, tyrosine-repressible, PLP-dependent	Cytoplasmic	COG1448;Aspartate/tyrosine/aromatic aminotransferase	GO:0009094 L-phenylalanine biosynthesis -!- GO:0006571 tyrosine biosynthesis -!- GO:0009098 leucine biosynthesis	gg	t
tyrP	CDS	tyrosine transporter	Integral Membrane Protein	COG0814;Amino acid permeases	GO:0006571 tyrosine biosynthesis	o	tt
tyrR	CDS	DNA-binding transcriptional dual regulator, tyrosine-binding	Cytoplasmic	COG3283;Transcriptional regulator of aromatic amino acids metabolism	GO:0006350 transcription	gg	tt
tyrS	CDS	tyrosyl-tRNA synthetase	Cytoplasmic	COG0162;Tyrosyl-tRNA synthetase	GO:0006418 amino acid activation	o	tt
tyrT	tRNA	tRNA-Tyr(GUA) (Tyrosine tRNA1)					
tyrU	tRNA	tRNA-Tyr(GUA) (Tyrosine tRNA2)					
tyrV	tRNA	tRNA-Tyr(GUA) (Tyrosine tRNA1)					
ubiA	CDS	p-hydroxybenzoate octaprenyltransferase	Integral Membrane Protein	COG0382;4-hydroxybenzoate polyprenyltransferase and related prenyltransferases	GO:0009060 aerobic respiration	o	tt
ubiB	CDS	2-octaprenylphenol hydroxylase	Integral Membrane Protein	COG0661;Predicted unusual protein kinase		o	tt
ubiC	CDS	chorismate pyruvate lyase	Cytoplasmic		GO:0009060 aerobic respiration		
ubiD	CDS	3-octaprenyl-4-hydroxybenzoate decarboxylase	Cytoplasmic	COG0043;3-polyprenyl-4-hydroxybenzoate decarboxylase and related decarboxylases		g	t
ubiE	CDS	bifunctional 2-octaprenyl-6-methoxy-1,4-benzoquinone methylase -!- S-adenosylmethionine:2-DMK methyltransferase	Cytoplasmic		GO:0009060 aerobic respiration -!- GO:0009061 anaerobic respiration		
ubiF	CDS	2-octaprenyl-3-methyl-6-methoxy-1,4-benzoquinol oxygenase	Cytoplasmic	COG0654;2-polyprenyl-6-methoxyphenol hydroxylase and related FAD-dependent oxidoreductases		o	tt
ubiG	CDS	bifunctional 3-demethylubiquinone-9 3-methyltransferase -!- 2-octaprenyl-6-hydroxy phenol methylase	Cytoplasmic	COG2227;2-polyprenyl-3-methyl-5-hydroxy-6-metoxy-1 ,4-benzoquinol methylase	GO:0009060 aerobic respiration	gg	o
ubiH	CDS	2-octaprenyl-6-methoxyphenol hydroxylase, FAD/NAD(P)-binding	Cytoplasmic	COG0654;2-polyprenyl-6-methoxyphenol hydroxylase and related FAD-dependent oxidoreductases	GO:0009060 aerobic respiration	gg	t
ubiX	CDS	3-octaprenyl-4-hydroxybenzoate carboxy-lyase	Cytoplasmic	COG0163;3-polyprenyl-4-hydroxybenzoate decarboxylase	GO:0009060 aerobic respiration	g	o
ucpA	CDS	predicted oxidoredutase, sulfate metabolism protein	Cytoplasmic				
udk	CDS	uridine/cytidine kinase	Cytoplasmic	COG0572;Uridine kinase	GO:0015949 nucleobase, nucleoside and nucleotide interconversion	gg	t
udp	CDS	uridine phosphorylase	Cytoplasmic	COG2820;Uridine phosphorylase	GO:0015949 nucleobase, nucleoside and nucleotide interconversion	gg	o
ugd	CDS	UDP-glucose 6-dehydrogenase	Cytoplasmic	COG1004;Predicted UDP-glucose 6-dehydrogenase	GO:0009242 colanic acid biosynthesis -!- GO:0009269 response to dessication	gg	t
ugpA	CDS	glycerol-3-phosphate transporter subunit -!- membrane component of ABC superfamily	Integral Membrane Protein	COG1175;ABC-type sugar transport systems, permease components	GO:0009060 aerobic respiration -!- GO:0009061 anaerobic respiration -!- GO:0006071 glycerol metabolism -!- GO:0008654 phospholipid biosynthesis	o	tt
ugpB	CDS	glycerol-3-phosphate transporter subunit -!- periplasmic-binding component of ABC superfamily	Periplasmic	COG1653;ABC-type sugar transport system, periplasmic component	GO:0009060 aerobic respiration -!- GO:0009061 anaerobic respiration -!- GO:0006071 glycerol metabolism -!- GO:0008654 phospholipid biosynthesis	o	tt
ugpC	CDS	glycerol-3-phosphate transporter subunit -!- ATP-binding component of ABC superfamily	Cytoplasmic	COG3839;ABC-type sugar transport systems, ATPase components	GO:0009060 aerobic respiration -!- GO:0009061 anaerobic respiration -!- GO:0006071 glycerol metabolism -!- GO:0008654 phospholipid biosynthesis	g	o
ugpE	CDS	glycerol-3-phosphate transporter subunit -!- membrane component of ABC superfamily	Integral Membrane Protein	COG0395;ABC-type sugar transport system, permease component	GO:0009060 aerobic respiration -!- GO:0009061 anaerobic respiration -!- GO:0006071 glycerol metabolism -!- GO:0008654 phospholipid biosynthesis	o	tt
ugpQ	CDS	glycerophosphodiester phosphodiesterase, cytosolic	Cytoplasmic	COG0584;Glycerophosphoryl diester phosphodiesterase	GO:0006071 glycerol metabolism	gg	t
uhpA	CDS	DNA-binding response regulator in two-component regulatory system wtih UhpB	Cytoplasmic	COG2197;Response regulator containing a CheY-like receiver domain and an HTH DNA-binding domain	GO:0016052 carbohydrate catabolism -!- GO:0006350	o	tt
uhpB	CDS	sensory histidine kinase in two-component regulatory sytem with UhpA	Integral Membrane Protein		GO:0016052 carbohydrate catabolism -!- GO:0006464 protein	o	tt
uhpC	CDS	membrane protein regulates uhpT expression	Integral Membrane Protein	COG2271;Sugar phosphate permease	GO:0016052 carbohydrate catabolism -!- GO:0006350	o	tt
uhpT	CDS	hexose phosphate transporter	Integral Membrane Protein	COG2271;Sugar phosphate permease	GO:0016052 carbohydrate	o	tt
uidA	CDS	beta-D-glucuronidase	Cytoplasmic	COG3250;Beta-galactosidase/beta-glucuronidase	GO:0016052 carbohydrate	gg	tt
uidB	CDS	glucuronide transporter	Integral Membrane Protein	COG2211;Na+/melibiose symporter and related transporters	GO:0016052 carbohydrate	o	tt
uidC	CDS	predicted outer membrane porin protein	Outer Membrane B-barrel protein			o	tt
uidR	CDS	DNA-binding transcriptional repressor	Cytoplasmic	COG1309;Transcriptional regulator	GO:0016052 carbohydrate catabolism -!- GO:0006350	o	tt
ulaA	CDS	L-ascorbate-specific enzyme IIC component of PTS	Integral Membrane Protein		GO:0009401 phosphoenolpyruvate-dependent sugar phosphotransferase system		
ulaB	CDS	L-ascorbate-specific enzyme IIB component of PTS	Cytoplasmic	COG3414;Phosphotransferase system, galactitol-specific IIB component	GO:0009401 phosphoenolpyruvate-dependent sugar phosphotransferase system	o	t
ulaC	CDS	L-ascorbate-specific enzyme IIA component of PTS	Cytoplasmic	COG1762;Phosphotransferase system mannitol/fructose-specific IIA domain (Ntr-type)		gg	o
ulaD	CDS	3-keto-L-gulonate 6-phosphate decarboxylase	Cytoplasmic	COG0269;3-hexulose-6-phosphate synthase and related proteins		g	t
ulaE	CDS	L-xylulose 5-phosphate 3-epimerase	Cytoplasmic	COG3623;Putative L-xylulose-5-phosphate 3-epimerase		o	tt
ulaF	CDS	L-ribulose 5-phosphate 4-epimerase	Cytoplasmic	COG0235;Ribulose-5-phosphate 4-epimerase and related epimerases and aldolases		gg	t
ulaG	CDS	L-ascorbate 6-phosphate lactonase	Cytoplasmic	COG2220;Predicted Zn-dependent hydrolases of the beta-lactamase fold			
ulaR	CDS	DNA-binding transriptional dual regulator	Cytoplasmic	COG1349;Transcriptional regulators of sugar metabolism	GO:0006350 transcription	o	tt
umuC	CDS	DNA polymerase V, subunit C	Cytoplasmic	COG0389;Nucleotidyltransferase/DNA polymerase involved in DNA repair	GO:0006261 DNA dependent DNA replication -!- GO:0006281 DNA repair -!- GO:0009432 SOS response	o	tt
umuD	CDS	DNA polymerase V, subunit D	Cytoplasmic	COG1974;SOS-response transcriptional repressors (RecA-mediated autopeptidases)	GO:0006261 DNA dependent DNA replication -!- GO:0006281 DNA repair -!- GO:0006350 transcription -!- GO:0009432 SOS response	g	o
ung	CDS	uracil-DNA-glycosylase	Cytoplasmic	COG0692;Uracil DNA glycosylase	GO:0006281 DNA repair	gg	t
upp	CDS	uracil phosphoribosyltransferase	Cytoplasmic	COG0035;Uracil phosphoribosyltransferase	GO:0015949 nucleobase, nucleoside and nucleotide interconversion	gg	t
uppS	CDS	NAD kinase	Cytoplasmic	COG0061;Predicted sugar kinase		g	t
uraA	CDS	uracil transporter	Integral Membrane Protein	COG2233;Xanthine/uracil permeases	GO:0015949 nucleobase, nucleoside and nucleotide interconversion	o	tt
usg	CDS	predicted semialdehyde dehydrogenase	Cytoplasmic	COG0136;Aspartate-semialdehyde dehydrogenase		gg	tt
ushA	CDS	bifunctional UDP-sugar hydrolase -!- 5'-nucleotidase	Periplasmic	COG0737;5'-nucleotidase/2',3'-cyclic phosphodiesterase and related esterases	GO:0009226 nucleotide-sugar biosynthesis	o	tt
uspA	CDS	universal stress global response regulator	Cytoplasmic	COG0589;Universal stress protein UspA and related nucleotide-binding proteins	GO:0006950 response to stress	gg	o

uspE	CDS	stress-induced protein	Cytoplasmic	COG0589;Universal stress protein UspA and related nucleotide-binding proteins		gg	tt
uspF	CDS	stress-induced protein, ATP-binding protein	Cytoplasmic			gg	t
uspG	CDS	universal stress protein UP12	Cytoplasmic	COG0589;Universal stress protein UspA and related nucleotide-binding proteins		gg	o
uup	CDS	fused predicted transporter subunits -!- ATP-binding components of ABC superfamily	Cytoplasmic	COG0488;ATPase components of ABC transporters with duplicated ATPase domains		gg	tt
uvrA	CDS	ATPase and DNA damage recognition protein of nucleotide excision repair excinuclease UvrABC	Cytoplasmic	COG0178;Excinuclease ATPase subunit	GO:0006281 DNA repair -!- GO:0009314 response to radiation -!- GO:0009432 SOS response	o	tt
uvrB	CDS	excinulease of nucleotide excision repair, DNA damage recognition component	Cytoplasmic	COG0556;Helicase subunit of the DNA excision repair complex	GO:0006308 DNA catabolism -!- GO:0006281 DNA repair -!- GO:0006308 DNA catabolism -!- GO:0009314 response to radiation -!- GO:0009432 SOS response	gg	t
uvrC	CDS	excinuclease UvrABC, endonuclease subunit	Cytoplasmic		GO:0006308 DNA catabolism -!- GO:0006281 DNA repair -!- GO:0006308 DNA catabolism -!- GO:0009314 response to radiation	o	tt
uvrD	CDS	DNA-dependent ATPase I and helicase II	Cytoplasmic	COG0210;Superfamily I DNA and RNA helicases	GO:0006261 DNA dependent DNA replication -!- GO:0006281 DNA repair -!- GO:0009432 SOS response	o	tt
uvrY	CDS	DNA-binding response regulator in two-component regulatory system with BarA	Cytoplasmic	COG2197;Response regulator containing a CheY-like receiver domain and an HTH DNA-binding domain	GO:0006350 transcription	gg	t
uxaA	CDS	altronate hydrolase	Cytoplasmic	COG2721;Altronate dehydratase	GO:0016052 carbohydrate	gg	t
uxaB	CDS	altronate oxidoreductase, NAD-dependent	Cytoplasmic		GO:0016052 carbohydrate	gg	t
uxaC	CDS	uronate isomerase	Cytoplasmic	COG1904;Glucuronate isomerase	GO:0016052 carbohydrate	o	o
uxuA	CDS	mannonate hydrolase	Cytoplasmic	COG1312;D-mannonate dehydratase	GO:0016052 carbohydrate	gg	o
uxuB	CDS	D-mannonate oxidoreductase, NAD-binding	Cytoplasmic	COG0246;Mannitol-1-phosphate/altronate dehydrogenases	GO:0016052 carbohydrate	gg	t
uxuR	CDS	DNA-binding transcriptional repressor	Cytoplasmic	COG2186;Transcriptional regulators	GO:0016052 carbohydrate catabolism -!- GO:0006350	o	tt
vacJ	CDS	predicted lipoprotein	Outer Membrane Lipoprotein	COG2853;Surface lipoprotein		gg	t
valS	CDS	valyl-tRNA synthetase	Cytoplasmic	COG0525;Valyl-tRNA synthetase	GO:0006418 amino acid activation	gg	tt
valT	tRNA	tRNA-Val(UAC) (Valine tRNA1)					
valU	tRNA	tRNA-Val(UAC) (Valine tRNA1)					
valV	tRNA	tRNA-Val(GAC) (Valine tRNA2B)					
valW	tRNA	tRNA-Val(GAC) (Valine tRNA2A)					
valX	tRNA	tRNA-Val(UAC), (Valine tRNA1)					
valY	tRNA	tRNA-Val(UAC) (Valine tRNA1)					
valZ	tRNA	tRNA-Val(UAC) (Valine tRNA1)					
visC	CDS	predicted oxidoreductase, FAD/NAD(P)-binding domain	Membrane Lipoprotein	COG0654;2-polyprenyl-6-methoxyphenol hydroxylase and related FAD-dependent oxidoreductases		gg	t
vsr	CDS	DNA mismatch endonuclease of very short patch repair	Cytoplasmic	COG3727;DNA G:T-mismatch repair endonuclease	GO:0006308 DNA catabolism -!- GO:0006281 DNA repair -!- GO:0006308 DNA catabolism	gg	o
wbbH	CDS	O-antigen polymerase	Integral Membrane Protein		GO:0009243 O antigen biosynthesis -!- GO:0009243 O antigen biosynthesis -!- GO:0009103 lipopolysaccharide biosynthesis	o	tt
wbbI	CDS	conserved protein	Cytoplasmic			o	tt
wbbJ	CDS	predicted acyl transferase	Cytoplasmic	COG0110;Acetyltransferase (isoleucine patch superfamily)	GO:0009103 lipopolysaccharide biosynthesis	g	tt
wbbK	CDS	lipopolysaccharide biosynthesis protein	Cytoplasmic	COG0438;Glycosyltransferase		o	tt
wbbL	CDS	lipopolysaccharide biosynthesis protein, C-ter fragment (pseudogene)	Cytoplasmic			o	o
wbbL	ancestral	lipopolysaccharide biosynthesis protein	Cytoplasmic				
wbbL	CDS	lipopolysaccharide biosynthesis protein, N-ter fragment (pseudogene)	Cytoplasmic			g	o
wcaA	CDS	predicted glycosyl transferase	Cytoplasmic	COG0463;Glycosyltransferases involved in cell wall biogenesis	GO:0009242 colanic acid	gg	tt
wcaB	CDS	predicted acyl transferase	Cytoplasmic	COG1045;Serine acetyltransferase	GO:0009242 colanic acid	o	tt
wcaC	CDS	predicted glycosyl transferase	Cytoplasmic	COG0438;Glycosyltransferase	GO:0009242 colanic acid	gg	t
wcaD	CDS	predicted colanic acid polymerase	Integral Membrane Protein			o	tt
wcaE	CDS	predicted glycosyl transferase	Cytoplasmic	COG0463;Glycosyltransferases involved in cell wall biogenesis	GO:0009242 colanic acid	gg	tt
wcaF	CDS	predicted acyl transferase	Cytoplasmic	COG0110;Acetyltransferase (isoleucine patch superfamily)	GO:0009242 colanic acid	o	tt
wcaI	CDS	predicted glycosyl transferase	Cytoplasmic	COG0438;Glycosyltransferase	GO:0009242 colanic acid	gg	tt
wcaJ	CDS	predicted UDP-glucose lipid carrier transferase	Integral Membrane Protein	COG2148;Sugar transferases involved in lipopolysaccharide synthesis	GO:0009242 colanic acid	o	tt
wcaK	CDS	predicted pyruvyl transferase	Cytoplasmic	COG2327;Uncharacterized conserved protein	GO:0009242 colanic acid	gg	tt
wcaL	CDS	predicted glycosyl transferase	Cytoplasmic	COG0438;Glycosyltransferase	GO:0009242 colanic acid	gg	t
wcaM	CDS	predicted colanic acid biosynthesis protein	Periplasmic		GO:0009242 colanic acid	gg	t
wrbA	CDS	predicted flavoprotein in Trp regulation	Cytoplasmic	COG0655;Multimeric flavodoxin WrbA	GO:0000162 tryptophan biosynthesis	gg	t
wza	CDS	lipoprotein required for capsular polysaccharide translocation through the outer membrane	Outer Membrane Lipoprotein	COG1596;Periplasmic protein involved in polysaccharide export	GO:0009242 colanic acid biosynthesis	o	tt
wzb	CDS	protein-tyrosine phosphatase	Cytoplasmic	COG0394;Protein-tyrosine-phosphatase	GO:0009242 colanic acid	gg	o
wzc	CDS	protein-tyrosine kinase	Integral Membrane Protein	COG3206;Uncharacterized protein involved in exopolysaccharide biosynthesis -!- COG0489;ATPases involved in chromosome partitioning	GO:0009242 colanic acid biosynthesis -!- GO:0006464 protein	o	tt
wzxC	CDS	colanic acid exporter	Integral Membrane Protein	COG2244;Membrane protein involved in the export of O-antigen and teichoic acid		g	tt
wzxE	CDS	O-antigen translocase	Integral Membrane Protein	COG2244;Membrane protein involved in the export of O-antigen and teichoic acid	GO:0017004 cytochrome biogenesis	o	tt
wzyE	CDS	predicted Wzy protein involved in ECA polysaccharide chain elongation	Integral Membrane Protein		GO:0009246 enterobacterial common antigen biosynthesis	o	tt
wzzE	CDS	Entobacterial Common Antigen (ECA) polysaccharide chain length modulation protein	Integral Membrane Protein	COG3765;Chain length determinant protein	GO:0009246 enterobacterial common antigen biosynthesis		
xapA	CDS	purine nucleoside phosphorylase II	Cytoplasmic	COG0005;Purine nucleoside phosphorylase	GO:0015949 nucleobase, nucleoside and nucleotide interconversion	g	o
xapB	CDS	xanthosine transporter	Integral Membrane Protein	COG0477;Permeases of the major facilitator superfamily	GO:0016052 carbohydrate	o	tt
xapR	CDS	DNA-binding transcriptional activator	Cytoplasmic	COG0583;Transcriptional regulator	GO:0015949 nucleobase, nucleoside and nucleotide interconversion -!- GO:0006350 transcription	o	t
xdhA	CDS	xanthine dehydrogenase, molybdenum binding subunit	Cytoplasmic	COG1529;Aerobic-type carbon monoxide dehydrogenase, large subunit CoxL/CutL homologs			
xdhB	CDS	xanthine dehydrogenase, FAD-binding subunit	Cytoplasmic	COG1319;Aerobic-type carbon monoxide dehydrogenase, middle subunit CoxM/CutM homologs		gg	t
xdhC	CDS	xanthine dehydrogenase, Fe-S binding subunit	Cytoplasmic	COG2080;Aerobic-type carbon monoxide dehydrogenase, small subunit CoxS/CutS homologs		gg	t
xdhD	CDS	fused predicted xanthine/hypoxanthine oxidase, molybdopterin-binding subunit -!- Fe-S binding subunit	Cytoplasmic	COG2080;Aerobic-type carbon monoxide dehydrogenase, small subunit CoxS/CutS homologs -!- COG1529;Aerobic-type carbon monoxide dehydrogenase, large subunit CoxL/CutL homologs		o	tt
xerC	CDS	site-specific tyrosine recombinase	Cytoplasmic	COG4973;Site-specific recombinase XerC	GO:0006310 DNA recombination	o	tt
xerD	CDS	site-specific tyrosine recombinase	Cytoplasmic	COG4974;Site-specific recombinase XerD	GO:0006310 DNA recombination	o	tt
xseA	CDS	exonuclease VII, large subunit	Cytoplasmic	COG1570;Exonuclease VII, large subunit	GO:0006308 DNA catabolism -!- GO:0006308 DNA catabolism	gg	tt
xseB	CDS	exonuclease VII small subunit	Cytoplasmic	COG1722;Exonuclease VII small subunit	GO:0006308 DNA catabolism -!- GO:0006281 DNA repair -!- GO:0006308 DNA catabolism	gg	t
xthA	CDS	exonuclease III	Cytoplasmic	COG0708;Exonuclease III	GO:0006308 DNA catabolism -!- GO:0006308 DNA catabolism -!- GO:0009314 response to radiation	gg	o
xylA	CDS	D-xylose isomerase	Cytoplasmic	COG2115;Xylose isomerase	GO:0016052 carbohydrate catabolism -!- GO:0006006 glucose	g	t
xylB	CDS	xylulokinase	Cytoplasmic	COG1070;Sugar (pentulose and hexulose) kinases	GO:0016052 carbohydrate	o	tt
xylE	CDS	D-xylose transporter	Integral Membrane Protein	COG0477;Permeases of the major facilitator superfamily	GO:0016052 carbohydrate catabolism -!- GO:0015753 D-xylose	o	tt
xylF	CDS	D-xylose transporter subunit -!- periplasmic-binding component of ABC superfamily	Periplasmic	COG4213;ABC-type xylose transport system, periplasmic component	GO:0016052 carbohydrate catabolism	o	tt
xylG	CDS	fused subunits of D-xylose transporter -!- ATP-binding components of ABC superfamily	Cytoplasmic	COG1129;ABC-type sugar transport system, ATPase component	GO:0016052 carbohydrate catabolism	o	t
xylH	CDS	D-xylose transporter subunit -!- membrane component of ABC superfamily	Integral Membrane Protein	COG4214;ABC-type xylose transport system, permease component	GO:0016052 carbohydrate catabolism	o	tt
xylR	CDS	DNA-binding transcriptional activator, xylose-binding	Cytoplasmic	COG1609;Transcriptional regulators -!- COG4753;Response regulator containing CheY-like receiver domain and AraC-type DNA-binding domain	GO:0016052 carbohydrate catabolism -!- GO:0006350	gg	t
yaaA	CDS	conserved protein	Cytoplasmic	COG3022;Uncharacterized protein conserved in bacteria		g	t
yaaH	CDS	conserved inner membrane protein associated with acetate transport	Integral Membrane Protein	COG1584;Predicted membrane protein		o	tt
yaaI	CDS	predicted protein	Periplasmic			o	tt
yaaJ	CDS	predicted transporter	Integral Membrane Protein	COG1115;Na+/alanine symporter		o	tt
yaaU	CDS	predicted transporter	Integral Membrane Protein	COG0477;Permeases of the major facilitator superfamily		o	tt
yaaW	CDS	conserved protein	Cytoplasmic	COG4735;Uncharacterized protein conserved in bacteria			
yaaX	CDS	predicted protein	Periplasmic			o	tt
yaaY	CDS	predicted protein	Membrane Anchored				
yabI	CDS	conserved inner membrane protein	Integral Membrane Protein	COG0586;Uncharacterized membrane-associated protein			
yabP	CDS	predicted protein	Cytoplasmic			g	o
yabQ	CDS	predicted protein	Cytoplasmic			gg	o
yacC	CDS	predicted protein	Periplasmic			o	tt
yacF	CDS	conserved protein	Cytoplasmic	COG4582;Uncharacterized protein conserved in bacteria		gg	t
yacG	CDS	conserved protein	Cytoplasmic	COG3024;Uncharacterized protein conserved in bacteria			
yacH	CDS	predicted protein	Periplasmic			gg	t
yacL	CDS	conserved protein	Cytoplasmic	COG3112;Uncharacterized protein conserved in bacteria		gg	t
yadB	CDS	glutamyl-Q tRNA(Asp) synthetase	Cytoplasmic	COG0008;Glutamyl- and glutaminyl-tRNA synthetases	GO:0006418 amino acid activation		
yadC	CDS	predicted fimbrial-like adhesin protein	Periplasmic	COG3539;P pilus assembly protein, pilin FimA		o	tt

yadD	CDS	predicted transposase	Cytoplasmic				
yadE	CDS	predicted polysaccharide deacetylase lipoprotein	Periplasmic	COG0726;Predicted xylanase/chitin deacetylase		o	tt
yadG	CDS	predicted transporter subunit -!- ATP-binding component of ABC superfamily	Cytoplasmic	COG1131;ABC-type multidrug transport system, ATPase component		gg	tt
yadH	CDS	predicted transporter subunit -!- membrane component of ABC superfamily	Integral Membrane Protein	COG0842;ABC-type multidrug transport system, permease component		o	tt
yadI	CDS	predicted PTS Enzyme IIA	Cytoplasmic	COG2893;Phosphotransferase system, mannose/fructose-specific component IIA	GO:0009401 phosphoenolpyruvate-dependent sugar phosphotransferase system	gg	o
yadK	CDS	predicted fimbrial-like adhesin protein	Periplasmic			o	tt
yadL	CDS	predicted fimbrial-like adhesin protein	Periplasmic			o	tt
yadM	CDS	predicted fimbrial-like adhesin protein	Periplasmic			o	tt
yadN	CDS	predicted fimbrial-like adhesin protein	Periplasmic	COG3539;P pilus assembly protein, pilin FimA		o	tt
yadR	CDS	conserved protein	Cytoplasmic	COG0316;Uncharacterized conserved protein		gg	t
yadS	CDS	conserved inner membrane protein	Integral Membrane Protein	COG2860;Predicted membrane protein		o	tt
yaeB	CDS	conserved protein	Cytoplasmic	COG1720;Uncharacterized conserved protein		gg	o
yaeF	CDS	predicted lipoprotein	Outer Membrane Lipoprotein				
yaeH	CDS	conserved protein	Cytoplasmic			o	tt
yaeI	CDS	predicted phosphatase	Membrane Anchored	COG1408;Predicted phosphohydrolases			
yaeJ	CDS	conserved protein	Cytoplasmic	COG1186;Protein chain release factor B		gg	o
yaeL	CDS	zinc metallopeptidase	Integral Membrane Protein	COG0750;Predicted membrane-associated Zn-dependent proteases 1		o	tt
yaeP	CDS	conserved protein	Cytoplasmic			gg	o
yaeQ	CDS	conserved protein	Cytoplasmic	COG4681;Uncharacterized protein conserved in bacteria		o	tt
yaeR	CDS	predicted lyase	Cytoplasmic			gg	t
yaeT	CDS	conserved protein	Outer Membrane B-barrel protein	COG4775;Outer membrane protein/protective antigen OMA87		o	tt
yafC	CDS	predicted DNA-binding transcriptional regulator	Cytoplasmic	COG0583;Transcriptional regulator	GO:0006350 transcription	o	tt
yafD	CDS	conserved protein	Cytoplasmic	COG3021;Uncharacterized protein conserved in bacteria			
yafE	CDS	predicted S-adenosyl-L-methionine-dependent methyltransferase	Cytoplasmic	COG0500;SAM-dependent methyltransferases	GO:0009102 biotin biosynthesis	o	o
yafF	CDS	conserved protein	Cytoplasmic			gg	o
yafJ	CDS	predicted amidotransfease	Cytoplasmic	COG0121;Predicted glutamine amidotransferase		gg	t
yafK	CDS	conserved protein	Periplasmic	COG3034;Uncharacterized protein conserved in bacteria		o	tt
yafL	CDS	predicted lipoprotein and C40 family peptidase	Periplasmic	COG0791;Cell wall-associated hydrolases (invasion-associated proteins)		o	t
yafM	CDS	conserved protein	Cytoplasmic	COG1943;Transposase and inactivated derivatives		gg	o
yafN	CDS	predicted antitoxin of the YafO-YafN toxin-antitoxin system	Cytoplasmic	COG2161;Antitoxin of toxin-antitoxin stability system			
yafO	CDS	predicted toxin of the YafO-YafN toxin-antitoxin	Cytoplasmic			gg	o
yafP	CDS	predicted acyltransferase with acyl-CoA N-acyltransferase domain	Cytoplasmic	COG0454;Histone acetyltransferase HPA2 and related acetyltransferases		gg	tt
yafQ	CDS	predicted toxin of the YafQ-DinJ toxin-antitoxin	Cytoplasmic	COG3041;Uncharacterized protein conserved in bacteria		gg	o
yafS	CDS	predicted S-adenosyl-L-methionine-dependent methyltransferase	Cytoplasmic	COG0500;SAM-dependent methyltransferases		o	tt
yafT	CDS	predicted aminopeptidase	Outer Membrane Lipoprotein			o	tt
yafU	CDS	predicted inner membrane protein	Integral Membrane Protein				
yafV	CDS	predicted C-N hydrolase family amidase, NAD(P)-binding	Cytoplasmic	COG0388;Predicted amidohydrolase			
yafW	CDS	CP4-6 prophage; antitoxin of the YkfI-YafW toxin-antitoxin system	Cytoplasmic			o	o
yafX	CDS	CP4-6 prophage; predicted protein	Cytoplasmic				
yafY	CDS	CP4-6 prophage; predicted DNA-binding transcriptional regulator	Cytoplasmic			o	tt
yafZ	CDS	CP4-6 prophage; conserved protein	Cytoplasmic			g	o
yagA	CDS	CP4-6 prophage; predicted DNA-binding transcriptional regulator	Cytoplasmic	COG2801;Transposase and inactivated derivatives		o	tt
yagB	CDS	CP4-6 prophage; conserved protein	Cytoplasmic			o	o
yagE	CDS	CP4-6 prophage; predicted lyase/synthase	Cytoplasmic	COG0329;Dihydrodipicolinate synthase/N-acetylneuraminate lyase		gg	tt
yagF	CDS	CP4-6 prophage; predicted dehydratase	Cytoplasmic	COG0129;Dihydroxyacid dehydratase/phosphogluconate dehydratase			
yagG	CDS	CP4-6 prophage; predicted sugar transporter	Integral Membrane Protein	COG2211;Na+/melibiose symporter and related transporters		o	tt
yagH	CDS	CP4-6 prophage; predicted xylosidase/arabinosidase	Cytoplasmic	COG3507;Beta-xylosidase		gg	tt
yagI	CDS	CP4-6 prophage; predicted DNA-binding transcriptional regulator	Cytoplasmic	COG1414;Transcriptional regulator	GO:0006350 transcription	o	tt
yagJ	CDS	CP4-6 prophage; predicted protein	Cytoplasmic			gg	o
yagK	CDS	CP4-6 prophage; conserved protein	Cytoplasmic			o	tt
yagL	CDS	CP4-6 prophage; DNA-binding protein	Cytoplasmic	COG1961;Site-specific recombinases, DNA invertase Pin homologs		o	tt
yagM	CDS	CP4-6 prophage; predicted protein	Cytoplasmic			o	o
yagN	CDS	CP4-6 prophage; predicted protein	Cytoplasmic				
yagP	CDS	predicted transcriptional regulator	Cytoplasmic	COG0583;Transcriptional regulator	GO:0006355 regulation of transcription, DNA-dependent	o	tt
yagQ	CDS	conserved protein	Cytoplasmic	COG1975;Xanthine and CO dehydrogenases maturation factor, XdhC/CoxF family		gg	tt
yagR	CDS	predicted oxidoreductase with molybdenum-binding domain	Periplasmic	COG1529;Aerobic-type carbon monoxide dehydrogenase, large subunit CoxL/CutL homologs		gg	tt
yagS	CDS	predicted oxidoreductase with FAD-binding domain	Membrane Anchored	COG1319;Aerobic-type carbon monoxide dehydrogenase, middle subunit CoxM/CutM homologs		o	tt
yagT	CDS	predicted xanthine dehydrogenase, 2Fe-2S subunit	Cytoplasmic	COG2080;Aerobic-type carbon monoxide dehydrogenase, small subunit CoxS/CutS homologs		gg	o
yagU	CDS	conserved inner membrane protein	Integral Membrane Protein	COG3477;Predicted periplasmic/secreted protein		o	tt
yagV	CDS	conserved protein	Periplasmic				
yagW	CDS	predicted receptor	Periplasmic			o	tt
yagX	CDS	predicted aromatic compound dioxygenase	Periplasmic			o	tt
yagY	CDS	predicted protein	Periplasmic			o	tt
yagZ	CDS	conserved protein	Periplasmic			o	tt
yahA	CDS	predicted DNA-binding transcriptional regulator	Cytoplasmic	COG2771;DNA-binding HTH domain-containing proteins -!- COG2200;FOG: EAL domain	GO:0006355 regulation of transcription, DNA-dependent	o	tt
yahB	CDS	predicted DNA-bindng transcriptional regulator	Periplasmic	COG0583;Transcriptional regulator	GO:0006350 transcription	gg	t
yahC	CDS	predicted inner membrane protein	Integral Membrane Protein			o	tt
yahD	CDS	predicted transcriptional regulator with ankyrin	Cytoplasmic	COG0666;FOG: Ankyrin repeat		gg	o
yahE	CDS	predicted protein	Cytoplasmic			gg	o
yahF	CDS	predicted acyl-CoA synthetase with NAD(P)-binding domain and succinyl-CoA synthetase domain	Cytoplasmic	COG0074;Succinyl-CoA synthetase, alpha subunit		o	tt
yahG	CDS	conserved protein	Cytoplasmic			o	tt
yahH	CDS	predicted protein	Membrane Anchored				
yahI	CDS	predicted carbamate kinase-like protein	Cytoplasmic	COG0549;Carbamate kinase	GO:0006526 arginine biosynthesis -!- GO:0006221 pyrimidine nucleotide biosynthesis	gg	t
yahJ	CDS	predicted deaminase with metallo-dependent hydrolase domain	Periplasmic	COG0402;Cytosine deaminase and related metal-dependent hydrolases	GO:0009310 amine catabolism	gg	t
yahK	CDS	predicted oxidoreductase, Zn-dependent and NAD(P)-binding	Cytoplasmic	COG1064;Zn-dependent alcohol dehydrogenases		gg	t
yahL	CDS	predicted protein	Cytoplasmic			o	tt
yahM	CDS	predicted protein	Cytoplasmic				
yahN	CDS	neutral amino-acid efflux system	Integral Membrane Protein	COG1280;Putative threonine efflux protein		o	tt
yahO	CDS	predicted protein	Periplasmic			o	tt
yaiA	CDS	predicted protein	Cytoplasmic			gg	o
yaiB	CDS	predicted protein	Cytoplasmic			gg	o
yaiC	CDS	predicted diguanylate cyclase	Integral Membrane Protein	COG2199;FOG: GGDEF domain		o	tt
yaiE	CDS	conserved protein	Cytoplasmic	COG3123;Uncharacterized protein conserved in bacteria		gg	o
yaiF	CDS	predicted protein	Cytoplasmic				
yaiI	CDS	conserved protein	Cytoplasmic	COG1671;Uncharacterized protein conserved in bacteria		gg	o
yaiL	CDS	nucleoprotein/polynucleotide-associated enzyme	Cytoplasmic			gg	tt
yaiO	CDS	predicted protein	Periplasmic			o	tt
yaiP	CDS	predicted glucosyltransferase	Integral Membrane Protein	COG1215;Glycosyltransferases, probably involved in cell wall biogenesis	GO:0000271 polysaccharide biosynthesis	o	tt
yaiS	CDS	conserved protein	Cytoplasmic			o	t
yaiT	CDS	predicted protein	Periplasmic			o	tt
yaiU	CDS	predicted protein	Outer Membrane B-barrel protein	COG3468;Type V secretory pathway, adhesin AidA	GO:0009296 flagella biogenesis -!- GO:0042330 taxis		
yaiV	CDS	predicted DNA-binding transcriptional regulator	Cytoplasmic			gg	o
yaiW	CDS	predicted DNA-binding transcriptional regulator	Outer Membrane Lipoprotein			o	tt
yaiX	CDS	predicted acyl transferase (pseudogene)	Cytoplasmic	COG0110;Acetyltransferase (isoleucine patch superfamily)		gg	t
yaiY	CDS	predicted inner membrane protein	Integral Membrane Protein			o	tt
yaiZ	CDS	predicted inner membrane protein	Integral Membrane Protein				
yajB	CDS	conserved protein	Cytoplasmic	COG3124;Uncharacterized protein conserved in bacteria		gg	t
yajC	CDS	SecYEG protein translocase auxillary subunit	Membrane Anchored	COG1862;Preprotein translocase subunit YajC		o	tt
yajD	CDS	conserved protein	Cytoplasmic			gg	o
yajG	CDS	predicted lipoprotein	Outer Membrane Lipoprotein			o	tt
yajI	CDS	predicted lipoprotein	Outer Membrane Lipoprotein				
yajL	CDS	conserved protein	Cytoplasmic		GO:0009102 biotin biosynthesis		
yajO	CDS	predicted oxidoreductase, NAD(P)-binding	Cytoplasmic	COG0667;Predicted oxidoreductases (related to aryl-alcohol dehydrogenases)		gg	t
yajQ	CDS	predicted nucleotide binding protein	Cytoplasmic	COG1666;Uncharacterized protein conserved in bacteria			
yajR	CDS	predicted transporter	Integral Membrane Protein		GO:0042493 response to drug		
ybaA	CDS	conserved protein	Cytoplasmic			gg	o
ybaB	CDS	conserved protein	Cytoplasmic	COG0718;Uncharacterized protein conserved in bacteria		gg	t

ybaD	CDS	conserved protein	Cytoplasmic	COG1327;Predicted transcriptional regulator, consists of a Zn-ribbon and ATP-cone domains		gg	o
ybaE	CDS	predicted transporter subunit -!- periplasmic-binding component of ABC superfamily	Cytoplasmic	COG4533;ABC-type uncharacterized transport system, periplasmic component		gg	t
ybaJ	CDS	predicted protein	Cytoplasmic			gg	t
ybaK	CDS	conserved protein	Cytoplasmic	COG2606;Uncharacterized conserved protein		gg	o
ybaL	CDS	predicted transporter with NAD(P)-binding Rossmann-fold domain	Integral Membrane Protein	COG4651;Kef-type K+ transport system, predicted NAD-binding component -!- COG1226;Kef-type K+ transport systems, predicted NAD-binding component		o	tt
ybaM	CDS	predicted protein	Cytoplasmic			gg	o
ybaN	CDS	conserved inner membrane protein	Integral Membrane Protein	COG2832;Uncharacterized protein conserved in bacteria		o	tt
ybaO	CDS	predicted DNA-binding transcriptional regulator	Cytoplasmic		GO:0006350 transcription	o	o
ybaP	CDS	conserved protein	Cytoplasmic	COG3735;Uncharacterized protein conserved in bacteria		gg	tt
ybaQ	CDS	predicted DNA-binding transcriptional regulator	Cytoplasmic			o	tt
ybaS	CDS	predicted glutaminase	Cytoplasmic	COG2066;Glutaminase	GO:0009063 amino acid catabolism	gg	t
ybaT	CDS	predicted transporter	Integral Membrane Protein	COG0531;Amino acid transporters	GO:0009063 amino acid catabolism	o	tt
ybaV	CDS	conserved protein	Periplasmic	COG1555;DNA uptake protein and related DNA-binding proteins		o	tt
ybaW	CDS	conserved protein	Cytoplasmic	COG0824;Predicted thioesterase		o	tt
ybaX	CDS	predicted aluminum resistance protein	Cytoplasmic	COG0603;Predicted PP-loop superfamily ATPase	GO:0006805 xenobiotic metabolism	gg	t
ybaY	CDS	predicted outer membrane lipoprotein	Outer Membrane Lipoprotein		GO:0042158 lipoprotein biosynthesis -!- GO:0009101 glycoprotein biosynthesis	o	tt
ybaZ	CDS	predicted methyltransferase	Cytoplasmic	COG3695;Predicted methylated DNA-protein cysteine methyltransferase		o	tt
ybbA	CDS	predicted transporter subunit -!- ATP-binding component of ABC superfamily	Cytoplasmic	COG4181;Predicted ABC-type transport system involved in lysophospholipase L1 biosynthesis, ATPase component		gg	tt
ybbB	CDS	tRNA 2-selenouridine synthase, selenophosphate-dependent	Cytoplasmic	COG2603;Predicted ATPase		o	t
ybbC	CDS	predicted protein	Outer Membrane Lipoprotein			o	tt
ybbD	CDS	predicted protein	Membrane Anchored			o	tt
ybbJ	CDS	conserved inner membrane protein	Integral Membrane Protein	COG1585;Membrane protein implicated in regulation of membrane protease activity			
ybbK	CDS	predicted protease, membrane anchored	Membrane Anchored	COG0330;Membrane protease subunits, stomatin/prohibitin homologs		o	tt
ybbL	CDS	predicted transporter subunit -!- ATP-binding component of ABC superfamily	Cytoplasmic	COG4619;ABC-type uncharacterized transport system, ATPase component		gg	o
ybbM	CDS	predicted inner membrane protein	Integral Membrane Protein		GO:0042493 response to drug		
ybbN	CDS	predicted thioredoxin domain-containing protein	Cytoplasmic				
ybbO	CDS	predicted oxidoreductase with NAD(P)-binding Rossmann-fold domain	Cytoplasmic	COG1028;Dehydrogenases with different specificities (related to short-chain alcohol dehydrogenases)		o	tt
ybbP	CDS	predicted inner membrane protein	Integral Membrane Protein	COG3127;Predicted ABC-type transport system involved in lysophospholipase L1 biosynthesis, permease component		o	tt
ybbS	CDS	transcriptional activator of the allD operon	Cytoplasmic	COG0583;Transcriptional regulator	GO:0006350 transcription	gg	t
ybbV	CDS	predicted protein	Membrane Anchored				
ybbW	CDS	predicted allantoin transporter	Integral Membrane Protein			o	tt
ybbY	CDS	predicted uracil/xanthine transporter	Integral Membrane Protein			o	tt
ybcC	CDS	DLP12 prophage; predicted exonuclease	Cytoplasmic			o	t
ybcD	CDS	DLP12 prophage; predicted replication protein fragment (pseudogene)	Cytoplasmic			gg	t
ybcF	CDS	predicted carbamate kinase	Cytoplasmic	COG0549;Carbamate kinase	GO:0006526 arginine biosynthesis -!- GO:0006221 pyrimidine nucleotide biosynthesis	gg	t
ybcH	CDS	predicted protein	Periplasmic			o	tt
ybcI	CDS	conserved inner membrane protein	Integral Membrane Protein	COG1988;Predicted membrane-bound metal-dependent hydrolases		o	o
ybcJ	CDS	predicted RNA-binding protein	Cytoplasmic	COG2501;Uncharacterized conserved protein			
ybcK	CDS	DLP12 prophage; predicted recombinase	Cytoplasmic	COG1961;Site-specific recombinases, DNA invertase Pin homologs		gg	t
ybcL	CDS	DLP12 prophage; predicted kinase inhibitor	Periplasmic	COG1881;Phospholipid-binding protein		o	tt
ybcM	CDS	DLP12 prophage; predicted DNA-binding transcriptional regulator	Cytoplasmic	COG2207;AraC-type DNA-binding domain-containing proteins		o	tt
ybcN	CDS	DLP12 prophage; predicted protein	Cytoplasmic			gg	o
ybcO	CDS	DLP12 prophage; predicted protein	Cytoplasmic			gg	o
ybcQ	CDS	DLP12 prophage; predicted antitermination protein	Cytoplasmic			o	tt
ybcS	CDS	DLP12 prophage; predicted lysozyme	Periplasmic	COG3772;Phage-related lysozyme (muraminidase)			
ybcV	CDS	DLP12 prophage; predicted protein	Cytoplasmic				
ybcW	CDS	DLP12 prophage; predicted protein	Cytoplasmic				
ybcY	CDS	DLP12 prophage; predicted SAM-dependent methyltransferase	Cytoplasmic				
ybdA	CDS	predicted transporter	Integral Membrane Protein	COG0477;Permeases of the major facilitator superfamily		o	tt
ybdB	CDS	conserved protein	Cytoplasmic	COG2050;Uncharacterized protein, possibly involved in aromatic compounds catabolism		gg	t
ybdD	CDS	conserved protein	Cytoplasmic			gg	o
ybdF	CDS	conserved protein	Cytoplasmic	COG2315;Uncharacterized protein conserved in bacteria			
ybdG	CDS	predicted mechanosensitive channel	Integral Membrane Protein	COG0668;Small-conductance mechanosensitive channel		o	tt
ybdH	CDS	predicted oxidoreductase	Cytoplasmic	COG0371;Glycerol dehydrogenase and related enzymes		gg	o
ybdJ	CDS	predicted inner membrane protein	Integral Membrane Protein			o	tt
ybdK	CDS	gamma-glutamyl:cysteine ligase	Cytoplasmic	COG2170;Uncharacterized conserved protein		gg	t
ybdL	CDS	methionine aminotransferase, PLP-dependent	Cytoplasmic	COG0436;Aspartate/tyrosine/aromatic aminotransferase	GO:0008152 metabolism	gg	o
ybdM	CDS	conserved protein	Cytoplasmic	COG1475;Predicted transcriptional regulators		gg	t
ybdN	CDS	conserved protein	Cytoplasmic	COG3969;Predicted phosphoadenosine phosphosulfate sulfotransferase		g	o
ybdO	CDS	predicted DNA-binding transcriptional regulator	Cytoplasmic	COG0583;Transcriptional regulator	GO:0006350 transcription -!- GO:0006355 regulation of transcription, DNA-dependent	g	o
ybdR	CDS	predicted oxidoreductase, Zn-dependent and NAD(P)-binding	Cytoplasmic	COG1063;Threonine dehydrogenase and related Zn-dependent dehydrogenases		gg	t
ybdZ	CDS	conserved protein	Cytoplasmic			gg	o
ybeA	CDS	conserved protein	Cytoplasmic	COG1576;Uncharacterized conserved protein		gg	t
ybeB	CDS	predicted protein	Cytoplasmic				
ybeD	CDS	conserved protein	Cytoplasmic	COG2921;Uncharacterized conserved protein		o	tt
ybeF	CDS	predicted DNA-binding transcriptional regulator	Cytoplasmic		GO:0006350 transcription	g	o
ybeH	CDS	predicted protein	Cytoplasmic			gg	t
ybeL	CDS	conserved protein	Cytoplasmic			gg	t
ybeM	CDS	predicted C-N hydrolase superfamily, NAD(P)-binding amidase/nitrilase	Cytoplasmic	COG0388;Predicted amidohydrolase		gg	t
ybeQ	CDS	conserved protein	Cytoplasmic				
ybeR	CDS	predicted protein	Cytoplasmic			gg	t
ybeT	CDS	conserved outer membrane protein	Outer Membrane Lipoprotein	COG0790;FOG: TPR repeat, SEL1 subfamily		o	tt
ybeU	CDS	predicted tRNA ligase	Cytoplasmic			o	tt
ybeX	CDS	predicteed ion transport	Cytoplasmic	COG4535;Putative Mg2+ and Co2+ transporter CorC		gg	o
ybeY	CDS	conserved protein	Cytoplasmic	COG0319;Predicted metal-dependent hydrolase		gg	tt
ybeZ	CDS	predicted protein with nucleoside triphosphate hydrolase domain	Cytoplasmic	COG1702;Phosphate starvation-inducible protein PhoH, predicted ATPase		g	t
ybfA	CDS	predicted protein	Membrane Anchored			o	tt
ybfB	CDS	predicted inner membrane protein	Integral Membrane Protein			o	tt
ybfC	CDS	predicted protein	Periplasmic			o	o
ybfD	CDS	conserved protein	Cytoplasmic			g	t
ybfE	CDS	LexA regulated protein	Cytoplasmic		GO:0009432 SOS response		
ybfF	CDS	conserved protein	Cytoplasmic	COG0596;Predicted hydrolases or acyltransferases (alpha/beta hydrolase superfamily)		g	o
ybfG	CDS	predicted protein	Cytoplasmic				
ybfH	CDS	predicted protein	Cytoplasmic				
ybfL	CDS	predicted transposase (pseudogene)	Cytoplasmic			o	tt
ybfM	CDS	predicted outer membrane porin	Outer Membrane B-barrel protein			o	o
ybfN	CDS	predicted lipoprotein	Outer Membrane Lipoprotein			o	tt
ybfO	CDS	conserved protein, rhs-like	Cytoplasmic	COG3209;Rhs family protein		gg	t
ybfP	CDS	predicted protein	Outer Membrane Lipoprotein				
ybfQ	CDS	predicted transposase	Cytoplasmic			o	t
ybgA	CDS	conserved protein	Cytoplasmic	COG3272;Uncharacterized conserved protein		gg	t
ybgC	CDS	predicted acyl-CoA thioesterase	Cytoplasmic	COG0824;Predicted thioesterase		gg	o
ybgD	CDS	predicted fimbrial-like adhesin protein	Periplasmic	COG3539;P pilus assembly protein, pilin FimA		o	tt
ybgE	CDS	conserved inner membrane protein	Integral Membrane Protein	COG3790;Predicted membrane protein		o	tt
ybgF	CDS	predicted protein	Periplasmic	COG1729;Uncharacterized protein conserved in bacteria		o	tt
ybgH	CDS	predicted transporter	Integral Membrane Protein	COG3104;Dipeptide/tripeptide permease	GO:0009063 amino acid catabolism	o	tt
ybgI	CDS	conserved metal-binding protein	Cytoplasmic	COG0327;Uncharacterized conserved protein		gg	tt
ybgJ	CDS	predicted enzyme subunit	Cytoplasmic	COG2049;Allophanate hydrolase subunit 1		g	t
ybgK	CDS	predicted enzyme subunit	Cytoplasmic	COG1984;Allophanate hydrolase subunit 2		gg	t
ybgL	CDS	predicted lactam utilization protein	Cytoplasmic	COG1540;Uncharacterized proteins, homologs of lactam utilization protein B	GO:0016052 carbohydrate catabolism -!- GO:0042493 response	gg	t
ybgO	CDS	predicted fimbrial-like adhesin protein	Periplasmic				
ybgP	CDS	predicted assembly protein	Periplasmic	COG3121;P pilus assembly protein, chaperone PapD	GO:0006457 protein folding	o	tt
ybgQ	CDS	predicted outer membrane protein	Outer Membrane B-barrel protein	COG3188;P pilus assembly protein, porin PapC			
ybgS	CDS	conserved protein	Periplasmic			o	tt
ybgT	CDS	conserved protein	Cytoplasmic			o	tt
ybhA	CDS	predicted hydrolase	Cytoplasmic	COG0561;Predicted hydrolases of the HAD superfamily		o	t
ybhB	CDS	predicted kinase inhibitor	Cytoplasmic	COG1881;Phospholipid-binding protein		o	t

ybhC	CDS	predicted pectinesterase	Outer Membrane Lipoprotein	COG4677;Pectin methylesterase	GO:0008152 metabolism -!- GO:0000272 polysaccharide catabolism	o	tt
ybhD	CDS	predicted DNA-binding transcriptional regulator	Membrane Anchored		GO:0006350 transcription		
ybhE	CDS	6-phosphogluconolactonase	Cytoplasmic	COG2706;3-carboxymuconate cyclase		gg	tt
ybhF	CDS	fused predicted transporter subunits -!- ATP-binding components of ABC superfamily	Cytoplasmic				
ybhG	CDS	predicted membrane fusion protein (MFP) component of efflux pump, membrane anchor	Membrane Anchored	COG0845;Membrane-fusion protein		o	tt
ybhH	CDS	conserved protein	Cytoplasmic	COG2828;Uncharacterized protein conserved in bacteria		gg	o
ybhI	CDS	predicted transporter	Integral Membrane Protein	COG0471;Di- and tricarboxylate transporters		o	tt
ybhJ	CDS	predicted hydratase	Cytoplasmic	COG1048;Aconitase A			
ybhK	CDS	predicted transferase with NAD(P)-binding Rossmann-fold domain	Cytoplasmic	COG0391;Uncharacterized conserved protein		gg	tt
ybhL	CDS	predicted inner membrane protein	Integral Membrane Protein	COG0670;Integral membrane protein, interacts with FtsH		o	tt
ybhM	CDS	conserved inner membrane protein	Integral Membrane Protein	COG0670;Integral membrane protein, interacts with FtsH		o	o
ybhN	CDS	conserved inner membrane protein	Integral Membrane Protein	COG0392;Predicted integral membrane protein		o	tt
ybhO	CDS	cardiolipin synthase 2	Cytoplasmic	COG1502;Phosphatidylserine/phosphatidylglycerophos phate/cardiolipin synthases and related enzymes	GO:0008654 phospholipid biosynthesis	gg	t
ybhP	CDS	predicted DNase	Cytoplasmic	COG3568;Metal-dependent hydrolase	GO:0006308 DNA catabolism	gg	t
ybhQ	CDS	predicted inner membrane protein	Integral Membrane Protein			o	tt
ybhR	CDS	predicted transporter subunit -!- membrane component of ABC superfamily	Integral Membrane Protein	COG0842;ABC-type multidrug transport system, permease component			
ybhS	CDS	predicted transporter subunit -!- membrane component of ABC superfamily	Integral Membrane Protein	COG0842;ABC-type multidrug transport system, permease component		o	tt
ybhT	CDS	predicted protein	Membrane Anchored				
ybiA	CDS	conserved protein	Cytoplasmic	COG3236;Uncharacterized protein conserved in bacteria		gg	t
ybiB	CDS	predicted transferase/phosphorylase	Cytoplasmic	COG0547;Anthranilate phosphoribosyltransferase		o	tt
ybiC	CDS	predicted dehydrogenase	Cytoplasmic	COG2055;Malate/L-lactate dehydrogenases		gg	t
ybiH	CDS	predicted DNA-binding transcriptional regulator	Cytoplasmic	COG1309;Transcriptional regulator	GO:0006350 transcription	o	tt
ybiI	CDS	conserved protein	Cytoplasmic	COG1734;DnaK suppressor protein		gg	o
ybiJ	CDS	predicted protein	Periplasmic			o	tt
ybiM	CDS	predicted protein	Periplasmic				
ybiN	CDS	predicted SAM-dependent methyltransferase	Cytoplasmic	COG3129;Predicted SAM-dependent methyltransferase			
ybiO	CDS	predicted mechanosensitive channel	Integral Membrane Protein				
ybiP	CDS	predicted hydrolase, inner membrane	Integral Membrane Protein	COG2194;Predicted membrane-associated, metal-dependent hydrolase		o	tt
ybiR	CDS	predicted transporter	Integral Membrane Protein	COG0471;Di- and tricarboxylate transporters		o	tt
ybiS	CDS	conserved protein	Periplasmic	COG1376;Uncharacterized protein conserved in bacteria		o	tt
ybiT	CDS	fused predicted transporter subunits -!- ATP-binding components of ABC superfamily	Cytoplasmic	COG0488;ATPase components of ABC transporters with duplicated ATPase domains		gg	t
ybiU	CDS	predicted protein	Cytoplasmic			gg	tt
ybiV	CDS	predicted hydrolase	Cytoplasmic	COG0561;Predicted hydrolases of the HAD superfamily		gg	t
ybiW	CDS	predicted pyruvate formate lyase	Cytoplasmic	COG1882;Pyruvate-formate lyase	GO:0006113 fermentation	gg	t
ybiX	CDS	conserved protein	Cytoplasmic				
ybiY	CDS	predicted pyruvate formate lyase activating enzyme	Cytoplasmic	COG1180;Pyruvate-formate lyase-activating enzyme	GO:0009061 anaerobic respiration -!- GO:0006464 protein modification	gg	t
ybjC	CDS	predicted inner membrane protein	Integral Membrane Protein			o	tt
ybjD	CDS	conserved protein with nucleoside triphosphate hydrolase domain	Cytoplasmic	COG3593;Predicted ATP-dependent endonuclease of the OLD family		gg	tt
ybjE	CDS	predicted transporter	Integral Membrane Protein	COG2431;Predicted membrane protein		o	tt
ybjG	CDS	undecaprenyl pyrophosphate phosphatase	Integral Membrane Protein	COG0671;Membrane-associated phospholipid phosphatase			
ybjH	CDS	predicted protein	Periplasmic			o	tt
ybjI	CDS	conserved protein	Cytoplasmic				
ybjJ	CDS	predicted transporter	Integral Membrane Protein	COG0477;Permeases of the major facilitator superfamily		o	tt
ybjK	CDS	predicted DNA-binding transcriptional regulator	Cytoplasmic	COG3226;Uncharacterized protein conserved in bacteria			
ybjL	CDS	predicted transporter	Integral Membrane Protein	COG2985;Predicted permease		o	tt
ybjM	CDS	predicted inner membrane protein	Integral Membrane Protein				
ybjN	CDS	predicted oxidoreductase	Cytoplasmic			o	tt
ybjO	CDS	predicted inner membrane protein	Integral Membrane Protein			o	tt
ybjP	CDS	predicted lipoprotein	Outer Membrane Lipoprotein			o	tt
ybjQ	CDS	conserved protein	Cytoplasmic	COG0393;Uncharacterized conserved protein		gg	t
ybjR	CDS	predicted amidase and lipoprotein	Outer Membrane Lipoprotein	COG3023;Negative regulator of beta-lactamase expression		o	tt
ybjS	CDS	predicted NAD(P)H oxidoreductase with NAD(P)-binding Rossmann-fold domain	Cytoplasmic	COG0451;Nucleoside-diphosphate-sugar epimerases			
ybjT	CDS	conserved protein with NAD(P)-binding Rossmann-fold domain	Membrane Anchored				
ybjX	CDS	conserved protein	Cytoplasmic	COG2990;Uncharacterized protein conserved in bacteria		o	tt
ycaC	CDS	predicted hydrolase	Cytoplasmic	COG1335;Amidases related to nicotinamidase	GO:0009063 amino acid catabolism	gg	o
ycaD	CDS	predicted transporter	Integral Membrane Protein	COG0477;Permeases of the major facilitator superfamily		o	tt
ycaI	CDS	conserved inner membrane protein	Integral Membrane Protein	COG0658;Predicted membrane metal-binding protein -!- COG2333;Predicted hydrolase (metallo-beta-lactamase superfamily)			
ycaJ	CDS	recombination protein	Cytoplasmic	COG2256;ATPase related to the helicase subunit of the Holliday junction resolvase		o	tt
ycaK	CDS	conserved protein	Cytoplasmic	COG2249;Putative NADPH-quinone reductase (modulator of drug activity		gg	o
ycaL	CDS	predicted peptidase with chaperone function	Outer Membrane Lipoprotein	COG0501;Zn-dependent protease with chaperone function	GO:0009266 response to temperature	o	tt
ycaM	CDS	predicted transporter	Integral Membrane Protein	COG0531;Amino acid transporters			
ycaN	CDS	predicted DNA-binding transcriptional regulator	Cytoplasmic	COG0583;Transcriptional regulator	GO:0006350 transcription	gg	t
ycaO	CDS	conserved protein	Cytoplasmic			gg	tt
ycaP	CDS	conserved inner membrane protein	Integral Membrane Protein	COG2323;Predicted membrane protein		o	tt
ycaQ	CDS	conserved protein	Cytoplasmic	COG3214;Uncharacterized protein conserved in bacteria		o	o
ycaR	CDS	conserved protein	Cytoplasmic	COG2835;Uncharacterized conserved protein		gg	o
ycbB	CDS	predicted carboxypeptidase	Periplasmic	COG2989;Uncharacterized protein conserved in bacteria		o	tt
ycbC	CDS	conserved inner membrane protein	Integral Membrane Protein	COG1434;Uncharacterized conserved protein		o	tt
ycbF	CDS	predicted periplasmic pilini chaperone	Periplasmic		GO:0006457 protein folding		
ycbG	CDS	conserved protein	Cytoplasmic	COG3120;Uncharacterized protein conserved in bacteria		o	o
ycbJ	CDS	conserved protein	Cytoplasmic			o	t
ycbK	CDS	conserved protein	Periplasmic	COG3108;Uncharacterized protein conserved in bacteria		gg	tt
ycbL	CDS	predicted metal-binding enzyme	Cytoplasmic	COG0491;Zn-dependent hydrolases, including glyoxylases		gg	o
ycbQ	CDS	predicted fimbrial-like adhesin protein	Periplasmic	COG3539;P pilus assembly protein, pilin FimA			
ycbR	CDS	predicted periplasmic pilin chaperone	Periplasmic	COG3121;P pilus assembly protein, chaperone PapD	GO:0006457 protein folding	o	tt
ycbS	CDS	predicted outer membrane usher protein	Outer Membrane B-barrel protein	COG3188;P pilus assembly protein, porin PapC		o	tt
ycbT	CDS	predicted fimbrial-like adhesin protein	Periplasmic	COG3539;P pilus assembly protein, pilin FimA		o	tt
ycbU	CDS	predicted fimbrial-like adhesin protein	Periplasmic	COG3539;P pilus assembly protein, pilin FimA		o	tt
ycbV	CDS	predicted fimbrial-like adhesin protein	Periplasmic	COG3539;P pilus assembly protein, pilin FimA			
ycbW	CDS	predicted protein	Cytoplasmic				
ycbX	CDS	predicted 2Fe-2S cluster-containing protein	Cytoplasmic	COG3217;Uncharacterized Fe-S protein -!- COG0633;Ferredoxin			
ycbY	CDS	predicted methyltransferase	Cytoplasmic	COG0116;Predicted N6-adenine-specific DNA methylase -!- COG1092;Predicted SAM-dependent methyltransferases		gg	tt
ycbZ	CDS	predicted peptidase	Cytoplasmic	COG1067;Predicted ATP-dependent protease	GO:0006508 proteolysis and peptidolysis	o	t
yccA	CDS	inner membrane protein	Integral Membrane Protein	COG0670;Integral membrane protein, interacts with FtsH		o	tt
yccC	CDS	cryptic autophosphorylating protein tyrosine kinase Etk	Integral Membrane Protein	COG3206;Uncharacterized protein involved in exopolysaccharide biosynthesis -!- COG0489;ATPases involved in chromosome partitioning	GO:0006464 protein modification	o	tt
yccE	CDS	predicted protein	Cytoplasmic			gg	tt
yccF	CDS	conserved inner membrane protein	Integral Membrane Protein	COG3304;Predicted membrane protein		o	tt
yccJ	CDS	predicted protein	Cytoplasmic			gg	o
yccK	CDS	predicted sulfite reductase subunit	Cytoplasmic			gg	tt
yccM	CDS	predicted 4Fe-4S membrane protein	Integral Membrane Protein	COG0348;Polyferredoxin		o	tt
yccR	CDS	conserved protein	Cytoplasmic	COG3070;Regulator of competence-specific genes		o	tt
yccS	CDS	predicted inner membrane protein	Integral Membrane Protein	COG1289;Predicted membrane protein			
yccT	CDS	conserved protein	Periplasmic	COG3110;Uncharacterized protein conserved in bacteria		o	tt
yccU	CDS	predicted CoA-binding protein with NAD(P)-binding Rossmann-fold domain	Cytoplasmic				
yccV	CDS	DNA-binding protein, hemimethylated	Cytoplasmic				
yccW	CDS	predicted methyltransferase	Cytoplasmic				
yccX	CDS	predicted acylphosphatase	Cytoplasmic	COG1254;Acylphosphatases			
yccZ	CDS	predicted exopolysaccharide export protein	Outer Membrane Lipoprotein	COG1596;Periplasmic protein involved in polysaccharide export		o	tt
ycdB	CDS	conserved protein	Periplasmic	COG2837;Predicted iron-dependent peroxidase		gg	t
ycdC	CDS	predicted DNA-binding transcriptional regulator	Cytoplasmic	COG1309;Transcriptional regulator	GO:0006350 transcription	o	tt
ycdG	CDS	predicted transporter	Integral Membrane Protein				
ycdH	CDS	predicted oxidoreductase, flavin:NADH component	Cytoplasmic				
ycdI	CDS	predicted oxidoreductase	Cytoplasmic	COG0778;Nitroreductase		gg	o
ycdK	CDS	conserved protein	Periplasmic	COG0251;Putative translation initiation inhibitor, yjgF family		g	o
ycdL	CDS	predicted enzyme	Cytoplasmic	COG1335;Amidases related to nicotinamidase			
ycdM	CDS	predicted monooxygenase	Cytoplasmic			gg	t
ycdN	ancestral	predicted membrane protein (pseudogene)	Cytoplasmic				
ycdN	CDS	predicted protein, N-ter fragment (pseudogene)	Cytoplasmic				
ycdN	CDS	predicted protein, C-ter fragment (pseudogene)	Cytoplasmic			o	tt
ycdO	CDS	conserved protein	Periplasmic	COG2822;Predicted periplasmic lipoprotein involved in iron transport		gg	o
ycdP	CDS	predicted inner membrane protein	Integral Membrane Protein			o	tt
ycdQ	CDS	predicted glycosyl transferase	Integral Membrane Protein	COG1215;Glycosyltransferases, probably involved in cell wall biogenesis		o	tt
ycdR	CDS	predicted enzyme associated with biofilm formation	Outer Membrane Lipoprotein	COG0726;Predicted xylanase/chitin deacetylase			
ycdS	CDS	predicted outer membrane protein	Outer Membrane B-barrel protein			o	tt

ycdT	CDS	predicted diguanylate cyclase	Integral Membrane Protein	COG2199;FOG: GGDEF domain			
ycdU	CDS	predicted inner membrane protein	Integral Membrane Protein			o	tt
ycdW	CDS	2-ketoacid reductase	Cytoplasmic	COG0111;Phosphoglycerate dehydrogenase and related dehydrogenases			
ycdX	CDS	predicted zinc-binding hydrolase	Cytoplasmic	COG1387;Histidinol phosphatase and related hydrolases of the PHP family	GO:0006261 DNA dependent DNA replication	gg	o
ycdY	CDS	conserved protein	Cytoplasmic	COG3381;Uncharacterized component of anaerobic dehydrogenases		gg	o
ycdZ	CDS	predicted inner membrane protein	Integral Membrane Protein				
yceA	CDS	conserved protein	Cytoplasmic	COG1054;Predicted sulfurtransferase	GO:0007049 cell cycle	o	tt
yceB	CDS	predicted lipoprotein	Outer Membrane Lipoprotein			o	tt
yceD	CDS	conserved protein	Cytoplasmic	COG1399;Predicted metal-binding, possibly nucleic acid-binding protein		gg	tt
yceF	CDS	predicted protein	Cytoplasmic	COG0424;Nucleotide-binding protein implicated in inhibition of septum formation			
yceG	CDS	predicted aminodeoxychorismate lyase	Membrane Anchored	COG1559;Predicted periplasmic solute-binding protein		tt	tt
yceH	CDS	conserved protein	Cytoplasmic	COG3132;Uncharacterized protein conserved in bacteria		g	tt
yceI	CDS	predicted protein	Periplasmic	COG2353;Uncharacterized conserved protein		o	tt
yceJ	CDS	predicted cytochrome b561	Integral Membrane Protein	COG3038;Cytochrome B561	GO:0017004 cytochrome biogenesis	o	tt
yceK	CDS	predicted lipoprotein	Membrane Lipoprotein				
yceO	CDS	predicted protein	Membrane Anchored				
yceP	CDS	predicted protein	Cytoplasmic				
yceQ	CDS	predicted protein	Cytoplasmic				
ycfD	CDS	conserved protein	Cytoplasmic			gg	tt
ycfH	CDS	predicted metallodependent hydrolase	Cytoplasmic	COG0084;Mg-dependent DNase		gg	o
ycfJ	CDS	predicted protein	Membrane Anchored	COG3134;Predicted outer membrane lipoprotein		o	tt
ycfK	CDS	e14 prophage; predicted protein	Cytoplasmic			o	t
ycfL	CDS	predicted protein	Outer Membrane Lipoprotein			o	tt
ycfM	CDS	predicted outer membrane lipoprotein	Outer Membrane Lipoprotein	COG3417;Collagen-binding surface adhesin SpaP (antigen I/II family)			
ycfN	CDS	thiamin kinase	Cytoplasmic	COG0510;Predicted choline kinase involved in LPS biosynthesis		gg	o
ycfP	CDS	conserved protein	Cytoplasmic				
ycfQ	CDS	predicted DNA-binding transcriptional regulator	Cytoplasmic		GO:0006355 regulation of transcription, DNA-dependent		
ycfR	CDS	predicted protein	Periplasmic			o	tt
ycfS	CDS	conserved protein	Periplasmic	COG1376;Uncharacterized protein conserved in bacteria			
ycfT	CDS	predicted inner membrane protein	Integral Membrane Protein	COG4763;Predicted membrane protein		gg	tt
ycfX	CDS	predicted DNA-binding transcriptional regulator	Cytoplasmic	COG1940;Transcriptional regulator/sugar kinase	GO:0006355 regulation of transcription, DNA-dependent	gg	t
ycfZ	CDS	predicted inner membrane protein	Integral Membrane Protein			o	tt
ycgB	CDS	conserved protein	Cytoplasmic	COG2719;Uncharacterized conserved protein		o	tt
ycgE	CDS	predicted DNA-binding transcriptional regulator	Cytoplasmic	COG0789;Predicted transcriptional regulators	GO:0006350 transcription	o	tt
ycgF	CDS	predicted FAD-binding phosphodiesterase	Cytoplasmic	COG2200;FOG: EAL domain		gg	o
ycgG	CDS	conserved inner membrane protein	Integral Membrane Protein	COG2200;FOG: EAL domain			
ycgH	ancestral	predicted protein (pseudogene)	Outer Membrane B-barrel protein				
ycgH	CDS	predicted protein, N-ter fragment (pseudogene)	Outer Membrane B-barrel protein				
ycgH	CDS	predicted protein, C-ter fragment (pseudogene)	Outer Membrane B-barrel protein				
ycgI	CDS	predicted protein	Cytoplasmic				
ycgJ	CDS	predicted protein	Periplasmic			o	tt
ycgK	CDS	predicted protein	Periplasmic			o	tt
ycgL	CDS	conserved protein	Cytoplasmic			gg	t
ycgM	CDS	predicted isomerase/hydrolase	Cytoplasmic	COG0179;2-keto-4-pentenoate hydratase/2-oxohepta-3-ene-1,7-dioic acid hydratase (catechol pathway)		o	o
ycgN	CDS	conserved protein	Cytoplasmic				
ycgR	CDS	protein involved in flagellar function	Cytoplasmic		GO:0006355 regulation of transcription, DNA-dependent	gg	t
ycgV	CDS	predicted adhesin	Cytoplasmic	COG3468;Type V secretory pathway, adhesin AidA		o	tt
ycgX	CDS	predicted protein	Cytoplasmic			gg	o
ycgY	CDS	predicted protein	Cytoplasmic			gg	o
ycgZ	CDS	predicted protein	Cytoplasmic			gg	o
ychA	CDS	predicted transcriptional regulator	Cytoplasmic	COG2912;Uncharacterized conserved protein	GO:0006355 regulation of transcription, DNA-dependent	gg	tt
ychE	CDS	predicted inner membrane protein	Integral Membrane Protein	COG2095;Multiple antibiotic transporter		o	o
ychF	CDS	predicted GTP-binding protein	Cytoplasmic	COG0012;Predicted GTPase, probable translation factor		gg	t
ychG	CDS	predicted protein, C-ter fragment (pseudogene)	Cytoplasmic			gg	t
ychG	ancestral	predicted protein (pseudogene)	Cytoplasmic				
ychG	CDS	predicted protein, N-ter fragment (pseudogene)	Cytoplasmic				
ychH	CDS	predicted inner membrane protein	Integral Membrane Protein			o	o
ychJ	CDS	conserved protein	Cytoplasmic	COG3012;Uncharacterized protein conserved in bacteria		gg	t
ychM	CDS	predicted transporter	Integral Membrane Protein	COG0659;Sulfate permease and related transporters (MFS superfamily)			
ychN	CDS	conserved protein	Cytoplasmic	COG1553;Uncharacterized conserved protein involved in intracellular sulfur reduction		g	t
ychP	CDS	predicted invasin	Cytoplasmic			gg	t
ychQ	CDS	predicted transcriptional regulator	Integral Membrane Protein	COG3094;Uncharacterized protein conserved in bacteria	GO:0006355 regulation of transcription, DNA-dependent	o	tt
ychS	CDS	predicted protein	Cytoplasmic				
yciA	CDS	predicted hydrolase	Cytoplasmic	COG1607;Acyl-CoA hydrolase		gg	o
yciB	CDS	predicted inner membrane protein	Integral Membrane Protein	COG2917;Intracellular septation protein A		o	tt
yciC	CDS	predicted inner membrane protein	Integral Membrane Protein			o	tt
yciE	CDS	conserved protein	Cytoplasmic	COG3685;Uncharacterized protein conserved in bacteria		gg	o
yciF	CDS	conserved protein	Cytoplasmic	COG3685;Uncharacterized protein conserved in bacteria		gg	t
yciG	CDS	predicted protein	Cytoplasmic			gg	o
yciH	CDS	conserved protein	Cytoplasmic	COG0023;Translation initiation factor 1 (eIF-1/SUI1) and related proteins		gg	t
yciI	CDS	predicted enzyme	Cytoplasmic	COG2350;Uncharacterized protein conserved in bacteria		gg	o
yciK	CDS	predicted oxoacyl-(acyl carrier protein) reductase, EmrKY-TolC system	Cytoplasmic	COG1028;Dehydrogenases with different specificities (related to short-chain alcohol dehydrogenases)		gg	t
yciM	CDS	conserved protein	Periplasmic	COG2956;Predicted N-acetylglucosaminyl transferase		gg	tt
yciN	CDS	predicted protein	Cytoplasmic			gg	t
yciO	CDS	conserved protein	Cytoplasmic				
yciQ	CDS	predicted inner membrane protein	Integral Membrane Protein				
yciS	CDS	conserved inner membrane protein	Integral Membrane Protein	COG3771;Predicted membrane protein		o	tt
yciT	CDS	predicted DNA-binding transcriptional regulator	Cytoplasmic	COG1349;Transcriptional regulators of sugar metabolism	GO:0006350 transcription		
yciU	CDS	predicted protein	Cytoplasmic			gg	tt
yciV	CDS	conserved protein	Cytoplasmic	COG0613;Predicted metal-dependent phosphoesterases (PHP family)		gg	t
yciW	CDS	predicted oxidoreductase	Cytoplasmic	COG4950;Uncharacterized protein conserved in bacteria -!- COG2128;Uncharacterized conserved protein			
yciX	CDS	predicted protein, N-ter fragment (pseudogene)	Membrane Anchored				
yciX	ancestral	predicted protein (pseudogene)	Membrane Anchored				
yciX	CDS	predicted protein, C-ter fragment (pseudogene)	Cytoplasmic				
ycjD	CDS	conserved protein	Cytoplasmic	COG2852;Uncharacterized protein conserved in bacteria		gg	t
ycjF	CDS	conserved inner membrane protein	Integral Membrane Protein	COG3768;Predicted membrane protein		gg	tt
ycjG	CDS	L-Ala-D/L-Glu epimerase	Cytoplasmic			o	t
ycjM	CDS	predicted glucosyltransferase	Cytoplasmic		GO:0000272 polysaccharide catabolism	gg	t
ycjN	CDS	predicted sugar transporter subunit -!- periplasmic-binding component of ABC superfamily	Outer Membrane Lipoprotein	COG1653;ABC-type sugar transport system, periplasmic component		o	tt
ycjO	CDS	predicted sugar transporter subunit -!- membrane component of ABC superfamily	Integral Membrane Protein	COG1175;ABC-type sugar transport systems, permease components		o	tt
ycjP	CDS	predicted sugar transporter subunit -!- membrane component of ABC superfamily	Integral Membrane Protein	COG0395;ABC-type sugar transport system, permease component		o	tt
ycjQ	CDS	predicted oxidoreductase, Zn-dependent and NAD(P)-binding	Cytoplasmic	COG1063;Threonine dehydrogenase and related Zn-dependent dehydrogenases		gg	o
ycjR	CDS	predicted enzyme	Cytoplasmic	COG1082;Sugar phosphate isomerases/epimerases			
ycjS	CDS	predicted oxidoreductase, NADH-binding	Cytoplasmic	COG0673;Predicted dehydrogenases and related proteins		o	o
ycjT	CDS	predicted hydrolase	Cytoplasmic	COG1554;Trehalose and maltose hydrolases (possible phosphorylases)		o	o
ycjU	CDS	predicted beta-phosphoglucomutase	Cytoplasmic	COG0637;Predicted phosphatase/phosphohexomutase		g	t
ycjV	CDS	predicted sugar transporter subunit -!- ATP-binding component of ABC superfamily	Cytoplasmic	COG3839;ABC-type sugar transport systems, ATPase components		gg	tt
ycjW	CDS	predicted DNA-binding transcriptional regulator	Cytoplasmic	COG1609;Transcriptional regulators	GO:0006350 transcription	gg	tt
ycjX	CDS	conserved protein with nucleoside triphosphate hydrolase domain	Cytoplasmic	COG3106;Predicted ATPase		ttgg	ttgg
ycjY	CDS	predicted hydrolase	Cytoplasmic	COG1073;Hydrolases of the alpha/beta superfamily			
ycjZ	CDS	predicted DNA-binding transcriptional regulator	Cytoplasmic	COG0583;Transcriptional regulator	GO:0006350 transcription	gg	t
ydaC	CDS	Rac prophage; predicted protein	Cytoplasmic			gg	o
ydaE	CDS	Rac prophage; conserved protein	Cytoplasmic			gg	o
ydaF	CDS	Rac prophage; predicted protein	Cytoplasmic			gg	o
ydaG	CDS	Rac prophage; predicted protein	Cytoplasmic				
ydaL	CDS	conserved protein	Cytoplasmic	COG2840;Uncharacterized protein conserved in bacteria		gg	t
ydaM	CDS	predicted diguanylate cyclase, GGDEF domain signalling protein	Cytoplasmic	COG2199;FOG: GGDEF domain			
ydaN	CDS	predicted Zn(II) transporter	Integral Membrane Protein	COG0598;Mg2+ and Co2+ transporters		o	tt
ydaO	CDS	predicted C32 tRNA thiolase	Cytoplasmic	COG0037;Predicted ATPase of the PP-loop superfamily implicated in cell cycle control		gg	o
ydaQ	CDS	Rac prophage; conserved protein	Cytoplasmic				
ydaS	CDS	Rac prophage; predicted DNA-binding transcriptional regulator	Periplasmic	COG4197;Uncharacterized protein conserved in bacteria, prophage-related		o	tt
ydaT	CDS	Rac prophage; predicted protein	Cytoplasmic			o	tt
ydaU	CDS	Rac prophage; conserved protein	Cytoplasmic	COG3756;Uncharacterized protein conserved in bacteria		g	tt

ydaV	CDS	Rac prophage; predicted DNA replication protein	Cytoplasmic	COG1484;DNA replication protein		gg	tt
ydaW	CDS	Rac prophage; predicted DNA-binding protein	Cytoplasmic				
ydaY	CDS	Rac prophage; predicted protein	Cytoplasmic			gg	o
ydbA	CDS	predicted outer membrane protein, N-ter fragment (pseudogene)	Cytoplasmic				
ydbA	ancestral	predicted outer membrane protein (pseudogene)	Cytoplasmic				
ydbA	CDS	predicted outer membrane protein, C-ter fragment (pseudogene)	Cytoplasmic			o	tt
ydbC	CDS	predicted oxidoreductase, NAD(P)-binding	Cytoplasmic	COG0667;Predicted oxidoreductases (related to aryl-alcohol dehydrogenases)		gg	o
ydbD	CDS	predicted protein	Periplasmic				
ydbH	CDS	predicted protein	Membrane Anchored			o	tt
ydbJ	CDS	predicted protein	Outer Membrane Lipoprotein				
ydbK	CDS	fused predicted Fe-S subunit of pyruvate-flavodoxin oxidoreductase	Cytoplasmic	COG0674;Pyruvate:ferredoxin oxidoreductase and related 2-oxoacid:ferredoxin oxidoreductases, alpha subunit -!- COG1014;Pyruvate:ferredoxin oxidoreductase and related 2-oxoacid:ferredoxin oxidoreductases, gamma subunit -!- COG1013;Pyruvate:ferredoxin oxidoreductase and related 2-oxoacid:ferredoxin oxidoreductases, beta subunit		o	tt
ydbL	CDS	conserved protein	Periplasmic	COG3784;Uncharacterized protein conserved in bacteria			
ydcA	CDS	predicted protein	Periplasmic			o	tt
ydcC	CDS	conserved protein	Cytoplasmic			gg	t
ydcD	CDS	predicted protein	Membrane Anchored			o	t
ydcE	CDS	4-oxalocrotonate tautomerase	Cytoplasmic	COG1942;Uncharacterized protein, 4-oxalocrotonate tautomerase homolog		gg	o
ydcF	CDS	conserved protein	Cytoplasmic	COG1434;Uncharacterized conserved protein		gg	o
ydcH	CDS	predicted protein	Cytoplasmic				
ydcI	CDS	predicted DNA-binding transcriptional regulator	Cytoplasmic	COG0583;Transcriptional regulator	GO:0006350 transcription		
ydcJ	CDS	conserved protein	Cytoplasmic	COG5383;Uncharacterized protein conserved in bacteria		gg	t
ydcK	CDS	predicted enzyme	Cytoplasmic			o	tt
ydcL	CDS	predicted lipoprotein	Outer Membrane Lipoprotein			o	tt
ydcM	CDS	predicted transposase	Cytoplasmic	COG0675;Transposase and inactivated derivatives			
ydcN	CDS	predicted DNA-binding transcriptional regulator	Cytoplasmic	COG1396;Predicted transcriptional regulators		g	t
ydcO	CDS	predicted benzoate transporter	Integral Membrane Protein				
ydcP	CDS	predicted peptidase	Cytoplasmic	COG0826;Collagenase and related proteases		gg	t
ydcQ	CDS	predicted DNA-binding transcriptional regulator	Cytoplasmic	COG1598;Uncharacterized conserved protein		o	o
ydcR	CDS	fused predicted DNA-binding transcriptional regulator -!- predicted amino transferase	Cytoplasmic	COG1167;Transcriptional regulators containing a DNA-binding HTH domain and an aminotransferase domain (MocR family) and their eukaryotic orthologs	GO:0006355 regulation of transcription, DNA-dependent	o	tt
ydcS	CDS	predicted spermidine/putrescine transporter subunit -!- periplasmic-binding component of ABC superfamily	Periplasmic	COG0687;Spermidine/putrescine-binding periplasmic protein		gg	tt
ydcT	CDS	predicted spermidine/putrescine transporter subunit -!- ATP-binding component of ABC superfamily	Cytoplasmic	COG3842;ABC-type spermidine/putrescine transport systems, ATPase components		gg	o
ydcU	CDS	predicted spermidine/putrescine transporter subunit -!- membrane component of ABC superfamily	Integral Membrane Protein	COG1176;ABC-type spermidine/putrescine transport system, permease component I		o	tt
ydcV	CDS	predicted spermidine/putrescine transporter subunit -!- membrane component of ABC superfamily	Integral Membrane Protein	COG1177;ABC-type spermidine/putrescine transport system, permease component II		o	tt
ydcW	CDS	medium chain aldehyde dehydrogenase	Cytoplasmic	COG1012;NAD-dependent aldehyde dehydrogenases		o	tt
ydcX	CDS	predicted inner membrane protein	Integral Membrane Protein				
ydcY	CDS	predicted protein	Cytoplasmic			gg	t
ydcZ	CDS	predicted inner membrane protein	Integral Membrane Protein	COG3238;Uncharacterized protein conserved in bacteria		o	tt
yddA	CDS	fused predicted multidrug transporter subunits -!- membrane component and ATP-binding component of ABC superfamily	Integral Membrane Protein	COG4178;ABC-type uncharacterized transport system, permease and ATPase components			
yddB	CDS	predicted porin protein	Periplasmic			o	tt
yddE	CDS	conserved protein	Cytoplasmic	COG0384;Predicted epimerase, PhzC/PhzF homolog		gg	o
yddG	CDS	predicted methyl viologen efflux pump	Integral Membrane Protein			o	tt
yddH	CDS	conserved protein	Cytoplasmic	COG1853;Conserved protein/domain typically associated with flavoprotein oxygenases, DIM6/NTAB family		gg	o
yddJ	CDS	predicted protein	Cytoplasmic				
yddK	CDS	predicted protein	Cytoplasmic	COG4886;Leucine-rich repeat (LRR) protein	GO:0009101 glycoprotein biosynthesis	o	o
yddL	CDS	predicted lipoprotein	Periplasmic	COG3203;Outer membrane protein (porin)		o	tt
yddM	CDS	predicted DNA-binding transcriptional regulator	Cytoplasmic		GO:0006355 regulation of transcription, DNA-dependent		
yddV	CDS	predicted diguanylate cyclase	Cytoplasmic	COG2199;FOG: GGDEF domain			
yddW	CDS	predicted liprotein	Outer Membrane Lipoprotein	COG1649;Uncharacterized protein conserved in bacteria		gg	tt
ydeA	CDS	predicted arabinose transporter	Integral Membrane Protein	COG2814;Arabinose efflux permease	GO:0016052 carbohydrate	g	t
ydeE	CDS	predicted transporter	Integral Membrane Protein	COG0477;Permeases of the major facilitator superfamily		o	tt
ydeH	CDS	conserved protein	Cytoplasmic	COG2199;FOG: GGDEF domain		gg	t
ydeI	CDS	conserved protein	Periplasmic	COG3111;Uncharacterized conserved protein		g	tt
ydeJ	CDS	conserved protein	Cytoplasmic	COG1546;Uncharacterized protein (competence- and mitomycin-induced)		gg	t
ydeK	CDS	predicted lipoprotein	Outer Membrane Lipoprotein				
ydeM	CDS	conserved protein	Cytoplasmic			gg	o
ydeN	CDS	conserved protein	Periplasmic	COG3119;Arylsulfatase A and related enzymes	GO:0006790 sulfur metabolism		
ydeO	CDS	predicted DNA-binding transcriptional acfivator	Cytoplasmic	COG2207;AraC-type DNA-binding domain-containing proteins	GO:0006350 transcription	gg	tt
ydeP	CDS	predicted oxidoreductase	Cytoplasmic	COG0243;Anaerobic dehydrogenases, typically selenocysteine-containing		o	tt
ydeQ	CDS	predicted fimbrial-like adhesin protein	Periplasmic			g	tt
ydeR	CDS	predicted fimbrial-like adhesin protein	Periplasmic	COG3539;P pilus assembly protein, pilin FimA		g	tt
ydeS	CDS	predicted fimbrial-like adhesin protein	Membrane Anchored	COG3539;P pilus assembly protein, pilin FimA		o	tt
ydeT	CDS	predicted protein	Cytoplasmic	COG3188;P pilus assembly protein, porin PapC		gg	t
ydeU	CDS	conserved protein, predicted pseudogene	Cytoplasmic	COG3468;Type V secretory pathway, adhesin AidA		gg	t
ydeV	CDS	predicted sugar kinase	Cytoplasmic			gg	tt
ydeW	CDS	predicted DNA-binding transcriptional regulator	Cytoplasmic	COG2390;Transcriptional regulator, contains sigma factor-related N-terminal domain			
ydfA	CDS	Qin prophage; predicted protein	Cytoplasmic			gg	o
ydfB	CDS	Qin prophage; predicted protein	Cytoplasmic			gg	o
ydfC	CDS	Qin prophage; predicted protein	Cytoplasmic			gg	o
ydfD	CDS	Qin prophage; predicted protein	Cytoplasmic			o	tt
ydfE	CDS	Qin prophage; predicted protein	Cytoplasmic			gg	tt
ydfG	CDS	L-allo-threonine dehydrogenase, NAD(P)-binding	Cytoplasmic	COG4221;Short-chain alcohol dehydrogenase of unknown specificity		gg	t
ydfH	CDS	predicted DNA-binding transcriptional regulator	Cytoplasmic	COG1802;Transcriptional regulators	GO:0006350 transcription	o	tt
ydfI	CDS	predicted mannonate dehydrogenase	Cytoplasmic	COG0246;Mannitol-1-phosphate/altronate dehydrogenases		g	tt
ydfJ	CDS	predicted transporter	Integral Membrane Protein	COG0477;Permeases of the major facilitator superfamily		o	tt
ydfK	CDS	Qin prophage; predicted DNA-binding transcriptional regulator	Cytoplasmic			gg	o
ydfO	CDS	Qin prophage; predicted protein	Cytoplasmic				
ydfP	CDS	Qin prophage; conserved protein	Periplasmic			o	tt
ydfQ	CDS	Qin prophage; predicted lysozyme	Membrane Anchored	COG3772;Phage-related lysozyme (muraminidase)		o	tt
ydfR	CDS	Qin prophage; predicted protein	Cytoplasmic			g	o
ydfT	CDS	Qin prophage; predicted antitermination protein Q	Cytoplasmic			o	tt
ydfU	CDS	Qin prophage; predicted protein	Cytoplasmic				
ydfV	CDS	Qin prophage; predicted protein	Cytoplasmic			o	t
ydfW	CDS	Qin prophage; predicted protein	Cytoplasmic			gg	o
ydfX	CDS	Qin prophage; predicted protein	Cytoplasmic			gg	o
ydfZ	CDS	conserved protein	Cytoplasmic			gg	o
ydgA	CDS	conserved protein	Periplasmic	COG5339;Uncharacterized protein conserved in bacteria		o	tt
ydgC	CDS	conserved inner membrane protein associated with alginate biosynthesis	Integral Membrane Protein	COG3136;Uncharacterized membrane protein required for alginate biosynthesis		o	tt
ydgD	CDS	predicted peptidase	Periplasmic	COG3591;V8-like Glu-specific endopeptidase		o	tt
ydgG	CDS	predicted inner membrane protein	Integral Membrane Protein	COG0628;Predicted permease		o	tt
ydgH	CDS	predicted protein	Periplasmic			o	tt
ydgI	CDS	predicted arginine/ornithine antiporter transporter	Integral Membrane Protein	COG0531;Amino acid transporters		o	tt
ydgJ	CDS	predicted oxidoreductase	Cytoplasmic				
ydgK	CDS	conserved inner membrane protein	Integral Membrane Protein			o	tt
ydgR	CDS	predicted transporter	Integral Membrane Protein	COG3104;Dipeptide/tripeptide permease		o	tt
ydgT	CDS	predicted regulator	Cytoplasmic			gg	o
ydhA	CDS	predicted lipoprotein	Outer Membrane Lipoprotein			gg	o
ydhB	CDS	predicted DNA-binding transcriptional regulator	Cytoplasmic	COG0583;Transcriptional regulator	GO:0006350 transcription	g	t
ydhC	CDS	predicted transporter	Integral Membrane Protein			gg	tt
ydhD	CDS	conserved protein	Cytoplasmic	COG0278;Glutaredoxin-related protein		gg	o
ydhF	CDS	predicted oxidoreductase	Cytoplasmic			gg	t
ydhH	CDS	conserved protein	Cytoplasmic	COG2377;Predicted molecular chaperone distantly related to HSP70-fold metalloproteases		gg	t
ydhI	CDS	predicted inner membrane protein	Integral Membrane Protein				
ydhJ	CDS	undecaprenyl pyrophosphate phosphatase	Membrane Anchored			o	tt
ydhK	CDS	conserved inner membrane protein	Integral Membrane Protein	COG1289;Predicted membrane protein		o	tt
ydhL	CDS	conserved protein	Cytoplasmic	COG3313;Predicted Fe-S protein			
ydhM	CDS	predicted DNA-binding transcriptional regulator	Cytoplasmic	COG1309;Transcriptional regulator	GO:0006355 regulation of transcription, DNA-dependent		
ydhO	CDS	predicted lipoprotein	Periplasmic	COG0791;Cell wall-associated hydrolases (invasion-associated proteins)			
ydhP	CDS	predicted transporter	Integral Membrane Protein	COG2814;Arabinose efflux permease		gg	tt
ydhQ	CDS	conserved protein	Cytoplasmic	COG3468;Type V secretory pathway, adhesin AidA		gg	t
ydhR	CDS	predicted protein	Cytoplasmic			gg	o
ydhS	CDS	conserved protein with FAD/NAD(P)-binding domain	Cytoplasmic	COG4529;Uncharacterized protein conserved in bacteria		gg	t
ydhT	CDS	conserved protein	Cytoplasmic			g	t

ydhU	CDS	predicted cytochrome	Integral Membrane Protein	COG4117;Thiosulfate reductase cytochrome B subunit (membrane anchoring protein)		o	tt
ydhV	CDS	predicted oxidoreductase	Cytoplasmic	COG2414;Aldehyde:ferredoxin oxidoreductase			
ydhW	CDS	predicted protein	Cytoplasmic			gg	t
ydhX	CDS	predicted 4Fe-4S ferridoxin-type protein	Periplasmic	COG0437;Fe-S-cluster-containing hydrogenase components 1			
ydhY	CDS	predicted 4Fe-4S ferridoxin-type protein	Cytoplasmic	COG0437;Fe-S-cluster-containing hydrogenase components 1		o	t
ydhZ	CDS	predicted protein	Cytoplasmic			gg	o
ydiA	CDS	conserved protein	Cytoplasmic	COG1806;Uncharacterized protein conserved in bacteria		gg	o
ydiB	CDS	quinate/shikimate 5-dehydrogenase, NAD(P)-binding	Cytoplasmic	COG0169;Shikimate 5-dehydrogenase		gg	o
ydiD	CDS	short chain acyl-CoA synthetase, anaerobic	Cytoplasmic				
ydiE	CDS	conserved protein	Cytoplasmic	COG4256;Hemin uptake protein		gg	o
ydiF	CDS	fused predicted acetyl-CoA:acetoacetyl-CoA transferase: alpha subunit -!- beta subunit	Cytoplasmic	COG4670;Acyl CoA:acetate/3-ketoacid CoA transferase	GO:0019395 fatty acid oxidation	g	t
ydiH	CDS	predicted protein	Cytoplasmic			o	t
ydiI	CDS	conserved protein	Cytoplasmic	COG2050;Uncharacterized protein, possibly involved in aromatic compounds catabolism		g	t
ydiJ	CDS	predicted FAD-linked oxidoreductase	Cytoplasmic	COG0277;FAD/FMN-containing dehydrogenases -!- COG0247;Fe-S oxidoreductase		o	tt
ydiK	CDS	predicted inner membrane protein	Integral Membrane Protein	COG0628;Predicted permease		o	tt
ydiL	CDS	conserved protein	Cytoplasmic			gg	o
ydiM	CDS	predicted transporter	Integral Membrane Protein	COG0477;Permeases of the major facilitator superfamily		o	tt
ydiN	CDS	predicted transporter	Integral Membrane Protein				
ydiO	CDS	predicted acyl-CoA dehydrogenase	Cytoplasmic				
ydiP	CDS	predicted DNA-binding transcriptional regulator	Cytoplasmic	COG2207;AraC-type DNA-binding domain-containing proteins	GO:0006350 transcription -!- GO:0006355 regulation of transcription, DNA-dependent	gg	o
ydiQ	CDS	conserved protein	Cytoplasmic	COG2086;Electron transfer flavoprotein, beta subunit			
ydiR	CDS	predicted electron transfer flavoprotein, FAD-binding	Cytoplasmic	COG2025;Electron transfer flavoprotein, alpha subunit		gg	t
ydiS	CDS	predicted oxidoreductase, FAD/NAD(P)-binding domain	Cytoplasmic	COG0644;Dehydrogenases (flavoproteins)		gg	t
ydiT	CDS	predicted 4Fe-4S ferredoxin-type protein	Cytoplasmic	COG2440;Ferredoxin-like protein		gg	o
ydiU	CDS	conserved protein	Cytoplasmic	COG0397;Uncharacterized conserved protein		gg	t
ydiV	CDS	conserved protein	Cytoplasmic	COG2200;FOG: EAL domain		g	o
ydiY	CDS	conserved protein	Periplasmic	COG3137;Putative salt-induced outer membrane protein		o	tt
ydiZ	CDS	predicted protein	Cytoplasmic			gg	t
ydjA	CDS	predicted oxidoreductase	Cytoplasmic	COG0778;Nitroreductase		gg	t
ydjE	CDS	predicted transporter	Integral Membrane Protein	COG0477;Permeases of the major facilitator superfamily		o	tt
ydjF	CDS	predicted DNA-binding transcriptional regulator	Cytoplasmic	COG1349;Transcriptional regulators of sugar metabolism	GO:0006350 transcription -!- GO:0006355 regulation of transcription, DNA-dependent	o	t
ydjG	CDS	predicted oxidoreductase	Periplasmic	COG0667;Predicted oxidoreductases (related to aryl-alcohol dehydrogenases)		gg	t
ydjH	CDS	predicted kinase	Cytoplasmic				
ydjI	CDS	predicted aldolase	Cytoplasmic	COG0191;Fructose/tagatose bisphosphate aldolase		gg	t
ydjJ	CDS	predicted oxidoreductase, Zn-dependent and NAD(P)-binding	Cytoplasmic	COG1063;Threonine dehydrogenase and related Zn-dependent dehydrogenases		gg	t
ydjK	CDS	predicted transporter	Integral Membrane Protein	COG0477;Permeases of the major facilitator superfamily			
ydjL	CDS	predicted oxidoreductase, Zn-dependent and NAD(P)-binding	Cytoplasmic	COG1063;Threonine dehydrogenase and related Zn-dependent dehydrogenases		o	t
ydjM	CDS	predicted inner membrane protein regulated by LexA	Integral Membrane Protein		GO:0009432 SOS response		
ydjN	CDS	predicted transporter	Integral Membrane Protein	COG1823;Predicted Na+/dicarboxylate symporter		o	tt
ydjO	CDS	predicted protein	Cytoplasmic			gg	o
ydjQ	CDS	endonuclease of nucleotide excision repair	Cytoplasmic	COG0322;Nuclease subunit of the excinuclease complex		gg	t
ydjR	CDS	conserved protein	Cytoplasmic	COG3758;Uncharacterized protein conserved in bacteria		g	o
ydjX	CDS	predicted inner membrane protein	Integral Membrane Protein			o	tt
ydjY	CDS	predicted protein	Inner Membrane Lipoprotein				
ydjZ	CDS	conserved inner membrane protein	Integral Membrane Protein	COG0398;Uncharacterized conserved protein		o	tt
yeaA	CDS	methionine sulfoxide reductase B	Cytoplasmic	COG0229;Conserved domain frequently associated with peptide methionine sulfoxide reductase		gg	o
yeaB	CDS	predicted NUDIX hydrolase	Cytoplasmic	COG0494;NTP pyrophosphohydrolases including oxidative damage repair enzymes		g	o
yeaC	CDS	conserved protein	Cytoplasmic			gg	o
yeaD	CDS	conserved protein	Cytoplasmic			gg	o
yeaE	CDS	predicted oxidoreductase	Cytoplasmic	COG0656;Aldo/keto reductases, related to diketogulonate reductase		gg	t
yeaG	CDS	conserved protein with nucleoside triphosphate hydrolase domain	Cytoplasmic	COG2766;Putative Ser protein kinase		gg	tt
yeaH	CDS	conserved protein	Cytoplasmic	COG2718;Uncharacterized conserved protein		gg	tt
yeaI	CDS	predicted diguanylate cyclase	Integral Membrane Protein	COG2199;FOG: GGDEF domain		o	tt
yeaJ	CDS	predicted diguanylate cyclase	Integral Membrane Protein	COG2199;FOG: GGDEF domain			
yeaK	CDS	conserved protein	Cytoplasmic	COG2606;Uncharacterized conserved protein		gg	o
yeaL	CDS	conserved inner membrane protein	Integral Membrane Protein	COG2707;Predicted membrane protein		o	tt
yeaM	CDS	predicted DNA-binding transcriptional regulator	Cytoplasmic	COG2207;AraC-type DNA-binding domain-containing proteins	GO:0006350 transcription -!- GO:0006355 regulation of transcription, DNA-dependent	gg	t
yeaN	CDS	predicted transporter	Integral Membrane Protein	COG2807;Cyanate permease	GO:0042886 amide transport	o	tt
yeaO	CDS	conserved protein	Cytoplasmic	COG3189;Uncharacterized conserved protein		gg	t
yeaP	CDS	predicted diguanylate cyclase	Cytoplasmic	COG2203;FOG: GAF domain -!- COG2199;FOG: GGDEF domain			
yeaQ	CDS	conserved inner membrane protein	Integral Membrane Protein	COG2261;Predicted membrane protein		o	tt
yeaR	CDS	conserved protein	Cytoplasmic	COG3615;Uncharacterized protein/domain, possibly involved in tellurite resistance	GO:0006805 xenobiotic metabolism	gg	o
yeaS	CDS	neutral amino-acid efflux system	Integral Membrane Protein	COG1280;Putative threonine efflux protein		o	tt
yeaT	CDS	predicted DNA-binding transcriptional regulator	Cytoplasmic		GO:0006350 transcription	g	t
yeaU	CDS	predicted dehydrogenase	Cytoplasmic	COG0473;Isocitrate/isopropylmalate dehydrogenase	GO:0006113 fermentation	gg	t
yeaV	CDS	predicted transporter	Integral Membrane Protein	COG1292;Choline-glycine betaine transporter			
yeaW	CDS	predicted 2Fe-2S cluster-containing protein	Cytoplasmic	COG4638;Phenylpropionate dioxygenase and related ring-hydroxylating dioxygenases, large terminal subunit			
yeaX	CDS	predicted oxidoreductase	Cytoplasmic	COG1018;Flavodoxin reductases (ferredoxin-NADPH reductases) family 1		gg	t
yeaY	CDS	predicted lipoprotein	Outer Membrane Lipoprotein	COG3065;Starvation-inducible outer membrane lipoprotein		o	tt
yeaZ	CDS	predicted peptidase	Cytoplasmic	COG1214;Inactive homolog of metal-dependent proteases, putative molecular chaperone		gg	o
yebA	CDS	predicted peptidase	Cytoplasmic	COG0739;Membrane proteins related to metalloendopeptidases			
yebB	CDS	predicted protein	Cytoplasmic				
yebC	CDS	conserved protein	Cytoplasmic	COG0217;Uncharacterized conserved protein		gg	o
yebE	CDS	conserved protein	Membrane Anchored	COG2979;Uncharacterized protein conserved in bacteria		gg	t
yebF	CDS	predicted protein	Periplasmic			o	tt
yebG	CDS	conserved protein regulated by LexA	Cytoplasmic	COG3141;Uncharacterized protein conserved in bacteria	GO:0006281 DNA repair -!- GO:0009432 SOS response	gg	t
yebK	CDS	predicted DNA-binding transcriptional regulator	Cytoplasmic	COG1737;Transcriptional regulators	GO:0006355 regulation of transcription, DNA-dependent	o	tt
yebN	CDS	conserved inner membrane protein	Integral Membrane Protein	COG1971;Predicted membrane protein			
yebO	CDS	predicted protein	Membrane Anchored			o	t
yebQ	CDS	predicted transporter	Integral Membrane Protein				
yebR	CDS	conserved protein	Cytoplasmic			gg	t
yebS	CDS	conserved inner membrane protein	Integral Membrane Protein	COG2995;Uncharacterized paraquat-inducible protein A		o	tt
yebT	CDS	conserved protein	Membrane Anchored	COG3008;Paraquat-inducible protein B		o	tt
yebU	CDS	predicted methyltransferase	Cytoplasmic	COG0144;tRNA and rRNA cytosine-C5-methylases -!- COG3270;Uncharacterized conserved protein	GO:0009451 RNA modification		
yebV	CDS	predicted protein	Cytoplasmic				
yebW	CDS	predicted protein	Cytoplasmic				
yebY	CDS	predicted protein	Periplasmic			o	o
yebZ	CDS	predicted inner membrane protein	Integral Membrane Protein	COG1276;Putative copper export protein	GO:0042493 response to drug	o	tt
yecA	CDS	conserved metal-binding protein	Cytoplasmic	COG3318;Predicted metal-binding protein related to the C-terminal domain of SecA		gg	tt
yecC	CDS	predicted transporter subunit -!- ATP-binding component of ABC superfamily	Cytoplasmic	COG1126;ABC-type polar amino acid transport system, ATPase component		gg	tt
yecD	CDS	predicted hydrolase	Cytoplasmic	COG1335;Amidases related to nicotinamidase			
yecE	CDS	conserved protein	Cytoplasmic	COG1801;Uncharacterized conserved protein		gg	t
yecF	CDS	predicted protein	Cytoplasmic			gg	o
yecG	CDS	universal stress protein	Cytoplasmic	COG0589;Universal stress protein UspA and related nucleotide-binding proteins		gg	o
yecH	CDS	predicted protein	Cytoplasmic			gg	o
yecI	CDS	predicted ferritin-like protein	Cytoplasmic	COG1528;Ferritin-like protein	GO:0006826 iron ion transport	gg	o
yecJ	CDS	predicted protein	Cytoplasmic			gg	o
yecM	CDS	predicted metal-binding enzyme	Cytoplasmic				
yecN	CDS	predicted inner membrane protein	Integral Membrane Protein				
yecO	CDS	predicted methyltransferase	Cytoplasmic	COG0500;SAM-dependent methyltransferases		gg	o
yecP	CDS	predicted methyltransferase	Cytoplasmic	COG0500;SAM-dependent methyltransferases		gg	t
yecR	CDS	predicted protein	Outer Membrane Lipoprotein			o	tt
yecS	CDS	predicted transporter subunit -!- membrane component of ABC superfamily	Integral Membrane Protein	COG0765;ABC-type amino acid transport system, permease component		o	tt
yecT	CDS	predicted protein	Periplasmic				
yedA	CDS	predicted inner membrane protein	Integral Membrane Protein	COG0697;Permeases of the drug/metabolite transporter (DMT) superfamily		gg	tt
yedD	CDS	predicted protein	Outer Membrane Lipoprotein			o	o
yedE	CDS	predicted inner membrane protein	Integral Membrane Protein	COG2391;Predicted transporter component		o	tt
yedF	CDS	conserved protein	Cytoplasmic	COG0425;Predicted redox protein, regulator of disulfide bond formation		gg	o
yedI	CDS	conserved inner membrane protein	Integral Membrane Protein	COG2354;Uncharacterized protein conserved in bacteria		g	tt

yedJ	CDS	predicted phosphohydrolase	Cytoplasmic	COG1418;Predicted HD superfamily hydrolase		gg	t
yedK	CDS	predicted protein	Cytoplasmic			gg	t
yedL	CDS	predicted acyltransferase	Cytoplasmic	COG0454;Histone acetyltransferase HPA2 and related acetyltransferases		g	o
yedM	CDS	conserved protein	Cytoplasmic	COG4886;Leucine-rich repeat (LRR) protein		gg	o
yedN	CDS	predicted protein, N-ter fragment (pseudogene)	Cytoplasmic				
yedN	ancestral	predicted protein (pseudogene)	Cytoplasmic				
yedN	CDS	predicted protein, C-ter fragment (pseudogene)	Cytoplasmic				
yedO	CDS	D-cysteine desulfhydrase, PLP-dependent	Cytoplasmic				
yedP	CDS	conserved protein	Cytoplasmic	COG3769;Predicted hydrolase (HAD superfamily)		gg	t
yedQ	CDS	predicted diguanylate cyclase	Integral Membrane Protein	COG2199;FOG: GGDEF domain			
yedR	CDS	predicted inner membrane protein	Integral Membrane Protein			gg	o
yedS	ancestral	predicted protein (pseudogene)	Cytoplasmic				
yedS	CDS	predicted protein, N-ter fragment (pseudogene)	Cytoplasmic				
yedS	CDS	predicted protein, middle fragment (pseudogene)	Cytoplasmic			o	o
yedS	CDS	predicted protein, C-ter fragment (pseudogene)	Cytoplasmic				
yedV	CDS	predicted sensory kinase in two-component regulatory system with YedW	Membrane Anchored	COG0642;Signal transduction histidine kinase	GO:0006464 protein modification	o	tt
yedW	CDS	predicted DNA-binding response regulator in two-component system withYedV	Cytoplasmic	COG0745;Response regulators consisting of a CheY-like receiver domain and a winged-helix DNA-binding domain	GO:0006350 transcription		
yedX	CDS	conserved protein	Periplasmic	COG2351;Transthyretin-like protein		gg	o
yedY	CDS	predicted reductase	Periplasmic	COG2041;Sulfite oxidase and related enzymes		gg	t
yedZ	CDS	conserved inner membrane protein	Integral Membrane Protein	COG2717;Predicted membrane protein		o	tt
yeeA	CDS	conserved inner membrane protein	Integral Membrane Protein	COG1289;Predicted membrane protein		o	tt
yeeD	CDS	conserved protein	Cytoplasmic	COG0425;Predicted redox protein, regulator of disulfide bond formation		gg	o
yeeE	CDS	predicted inner membrane protein	Integral Membrane Protein	COG2391;Predicted transporter component		o	o
yeeF	CDS	predicted amino-acid transporter	Integral Membrane Protein	COG0531;Amino acid transporters			
yeeI	CDS	conserved protein	Cytoplasmic	COG3228;Uncharacterized protein conserved in bacteria		gg	tt
yeeJ	CDS	adhesin	Cytoplasmic		GO:0006355 regulation of transcription, DNA-dependent		
yeeL	CDS	predicted protein, C-ter fragment (pseudogene)	Cytoplasmic				
yeeL	ancestral	predicted protein (pseudogene)	Cytoplasmic				
yeeL	CDS	predicted protein, N-ter fragment (pseudogene)	Cytoplasmic			gg	t
yeeN	CDS	conserved protein	Cytoplasmic	COG0217;Uncharacterized conserved protein		gg	t
yeeO	CDS	predicted multidrug efflux system	Integral Membrane Protein	COG0534;Na+-driven multidrug efflux pump		o	tt
yeeP	CDS	CP4-44 prophage; predicted GTP-binding protein (pseudogene)	Cytoplasmic	COG3596;Predicted GTPase			
yeeR	CDS	CP4-44 prophage; predicted membrane protein	Integral Membrane Protein			o	tt
yeeS	CDS	CP4-44 prophage; predicted DNA repair protein	Cytoplasmic	COG2003;DNA repair proteins		gg	o
yeeT	CDS	CP4-44 prophage; predicted protein	Cytoplasmic			gg	o
yeeU	CDS	CP4-44 prophage; antitoxin of the YeeV-YeeU toxin-antitoxin system	Cytoplasmic			gg	o
yeeV	CDS	CP4-44 prophage; toxin of the YeeV-YeeU toxin-antitoxin system	Cytoplasmic			gg	tt
yeeW	CDS	CP4-44 prophage; predicted protein	Cytoplasmic			gg	o
yeeX	CDS	conserved protein	Cytoplasmic			gg	t
yeeY	CDS	predicted DNA-binding transcriptional regulator	Cytoplasmic		GO:0006350 transcription		
yeeZ	CDS	predicted epimerase, with NAD(P)-binding Rossmann-fold domain	Periplasmic	COG0451;Nucleoside-diphosphate-sugar epimerases	GO:0016052 carbohydrate catabolism		
yefM	CDS	antitoxin of the YoeB-YefM toxin-antitoxin system	Cytoplasmic	COG2161;Antitoxin of toxin-antitoxin stability system			
yegD	CDS	predicted chaperone	Cytoplasmic		GO:0006457 protein folding	o	tt
yegE	CDS	predicted diguanylate cyclase, GGDEF domain signalling protein	Integral Membrane Protein	COG3447;Predicted integral membrane sensor domain -!- COG2202;FOG: PAS/PAC domain -!- COG2199;FOG: GGDEF domain -!- COG2200;FOG: EAL domain	GO:0006464 protein modification	o	tt
yegH	CDS	fused predicted membrane proteins	Integral Membrane Protein				
yegI	CDS	conserved protein	Cytoplasmic	COG4248;Uncharacterized protein with protein kinase and helix-hairpin-helix DNA-binding domains		o	tt
yegJ	CDS	predicted protein	Periplasmic	COG3779;Uncharacterized protein conserved in bacteria		o	tt
yegK	CDS	predicted protein	Cytoplasmic			o	tt
yegL	CDS	conserved protein	Cytoplasmic	COG4245;Uncharacterized protein encoded in toxicity protection region of plasmid R478, contains von Willebrand factor (vWF) domain		gg	t
yegP	CDS	predicted protein	Cytoplasmic				
yegQ	CDS	predicted peptidase	Cytoplasmic	COG0826;Collagenase and related proteases		gg	o
yegR	CDS	predicted protein	Outer Membrane Lipoprotein				
yegS	CDS	conserved protein	Cytoplasmic	COG1597;Sphingosine kinase and enzymes related to eukaryotic diacylglycerol kinase		g	t
yegT	CDS	predicted nucleoside transporter	Integral Membrane Protein	COG0477;Permeases of the major facilitator superfamily		o	tt
yegU	CDS	predicted hydrolase	Cytoplasmic	COG1397;ADP-ribosylglycohydrolase		gg	o
yegV	CDS	predicted kinase	Cytoplasmic	COG0524;Sugar kinases, ribokinase family			
yegW	CDS	predicted DNA-binding transcriptional regulator	Cytoplasmic	COG2188;Transcriptional regulators	GO:0006350 transcription	o	tt
yegX	CDS	predicted hydrolase	Membrane Anchored	COG3757;Lyzozyme M1 (1,4-beta-N-acetylmuramidase)			
yegZ	CDS	predicted protein fragment (pseudogene)	Cytoplasmic	COG3500;Phage protein D			
yehA	CDS	predicted fimbrial-like adhesin protein	Periplasmic			o	tt
yehB	CDS	predicted outer membrane protein	Outer Membrane B-barrel protein	COG3188;P pilus assembly protein, porin PapC		o	tt
yehC	CDS	predicted periplasmic pilin chaperone	Periplasmic	COG3121;P pilus assembly protein, chaperone PapD	GO:0006457 protein folding	o	tt
yehD	CDS	predicted fimbrial-like adhesin protein	Periplasmic	COG3539;P pilus assembly protein, pilin FimA		o	tt
yehE	CDS	predicted protein	Periplasmic			o	tt
yehI	CDS	conserved protein	Cytoplasmic			o	tt
yehK	CDS	predicted protein	Cytoplasmic			gg	o
yehL	CDS	predicted transporter subunit -!- ATP-binding compoents of ABC superfamily	Cytoplasmic	COG0714;MoxR-like ATPases			
yehM	CDS	predicted protein	Cytoplasmic			gg	tt
yehP	CDS	conserved protein	Cytoplasmic				
yehQ	CDS	predicted protein	Cytoplasmic			o	tt
yehR	CDS	conserved protein	Membrane Lipoprotein	COG4808;Uncharacterized protein conserved in bacteria			
yehS	CDS	conserved protein	Cytoplasmic	COG4807;Uncharacterized protein conserved in bacteria		gg	t
yehT	CDS	predicted response regulator in two-component system withYehU	Cytoplasmic				
yehU	CDS	predicted sensory kinase in two-component system with YehT	Integral Membrane Protein	COG3275;Putative regulator of cell autolysis	GO:0006464 protein modification		
yehW	CDS	predicted transporter subunit -!- membrane component of ABC superfamily	Integral Membrane Protein	COG1174;ABC-type proline/glycine betaine transport systems, permease component		o	tt
yehX	CDS	predicted transporter subunit -!- ATP-binding component of ABC superfamily	Cytoplasmic	COG1125;ABC-type proline/glycine betaine transport systems, ATPase components		g	t
yehY	CDS	predicted transporter subunit -!- membrane component of ABC superfamily	Integral Membrane Protein	COG1174;ABC-type proline/glycine betaine transport systems, permease component		o	tt
yehZ	CDS	predicted transporter subunit -!- periplasmic-binding component of ABC superfamily	Periplasmic	COG1732;Periplasmic glycine betaine/choline-binding (lipo)protein of an ABC-type transport system (osmoprotectant binding protein)		gg	o
yeiA	CDS	predicted oxidoreductase	Cytoplasmic	COG0167;Dihydroorotate dehydrogenase -!- COG1146;Ferredoxin		g	t
yeiB	CDS	conserved inner membrane protein	Integral Membrane Protein	COG2311;Predicted membrane protein		o	tt
yeiC	CDS	predicted kinase	Cytoplasmic	COG0524;Sugar kinases, ribokinase family		gg	t
yeiE	CDS	predicted DNA-binding transcriptional regulator	Cytoplasmic	COG0583;Transcriptional regulator	GO:0006350 transcription	gg	t
yeiG	CDS	predicted esterase	Cytoplasmic	COG0627;Predicted esterase		gg	o
yeiH	CDS	conserved inner membrane protein	Integral Membrane Protein	COG2855;Predicted membrane protein		o	tt
yeiI	CDS	predicted kinase	Cytoplasmic	COG2771;DNA-binding HTH domain-containing proteins -!- COG0524;Sugar kinases, ribokinase family		g	tt
yeiJ	CDS	predicted nucleoside transporter	Integral Membrane Protein	COG1972;Nucleoside permease		o	tt
yeiL	CDS	DNA-binding transcriptional activator	Cytoplasmic		GO:0006355 regulation of transcription, DNA-dependent	gg	o
yeiM	CDS	predicted nucleoside transporter	Integral Membrane Protein	COG1972;Nucleoside permease		g	tt
yeiN	CDS	conserved protein	Cytoplasmic	COG2313;Uncharacterized enzyme involved in pigment biosynthesis		gg	o
yeiP	CDS	predicted elongtion factor	Cytoplasmic		GO:0006412 protein biosynthesis		
yeiQ	CDS	predicted dehydrogenase, NAD-dependent	Cytoplasmic	COG0246;Mannitol-1-phosphate/altronate dehydrogenases		g	t
yeiR	CDS	predicted enzyme	Cytoplasmic	COG0523;Putative GTPases (G3E family)		gg	t
yeiS	CDS	predicted inner membrane protein	Integral Membrane Protein				
yeiT	CDS	predicted oxidoreductase	Cytoplasmic	COG0493;NADPH-dependent glutamate synthase beta chain and related oxidoreductases		gg	t
yeiU	CDS	undecaprenyl pyrophosphate phosphatase	Integral Membrane Protein			o	tt
yeiW	CDS	conserved protein					
yejA	CDS	predicted oligopeptide transporter subunit -!- periplasmic-binding component of ABC superfamily	Periplasmic			o	tt
yejB	CDS	predicted oligopeptide transporter subunit -!- membrane component of ABC superfamily	Integral Membrane Protein	COG4174;ABC-type uncharacterized transport system, permease component		o	tt
yejE	CDS	predicted oligopeptide transporter subunit -!- membrane component of ABC superfamily	Integral Membrane Protein	COG4239;ABC-type uncharacterized transport system, permease component		o	tt
yejF	CDS	fused predicted oligopeptide transporter subunits -!- ATP-binding components of ABC superfamily	Cytoplasmic	COG4172;ABC-type uncharacterized transport system, duplicated ATPase component		o	tt
yejG	CDS	predicted protein	Cytoplasmic			gg	o
yejH	CDS	predicted ATP-dependet helicase	Cytoplasmic	COG1061;DNA or RNA helicases of superfamily II		g	t
yejK	CDS	nucleotide associated protein	Cytoplasmic	COG3081;Nucleoid-associated protein		o	tt
yejL	CDS	conserved protein	Cytoplasmic	COG3082;Uncharacterized protein conserved in bacteria		gg	o
yejM	CDS	predicted hydrolase, inner membrane	Integral Membrane Protein	COG3083;Predicted hydrolase of alkaline phosphatase superfamily		o	tt
yejO	CDS	predicted autotransporter outer membrane protein	Cytoplasmic	COG3468;Type V secretory pathway, adhesin AidA			
yfaA	CDS	predicted protein	Periplasmic			g	tt
yfaD	CDS	conserved protein	Cytoplasmic			gg	o
yfaE	CDS	predicted 2Fe-2S cluster-containing protein	Cytoplasmic	COG0633;Ferredoxin		g	t
yfaH	CDS	conserved protein	Cytoplasmic	COG0583;Transcriptional regulator		g	o
yfaL	CDS	adhesin	Periplasmic	COG3468;Type V secretory pathway, adhesin AidA		gg	tt

yfaO	CDS	predicted NUDIX hydrolase	Cytoplasmic	COG0494;NTP pyrophosphohydrolases including oxidative damage repair enzymes		g	t
yfaP	CDS	conserved protein	Periplasmic	COG4676;Uncharacterized protein conserved in bacteria		o	tt
yfaQ	CDS	predicted protein	Periplasmic			g	tt
yfaS	CDS	predicted protein, C-ter fragment (pseudogene)	Periplasmic			gg	o
yfaS	ancestral	predicted protein (pseudogene)	Periplasmic				
yfaS	CDS	predicted protein, N-ter fragment (pseudogene)	Periplasmic			o	tt
yfaT	CDS	predicted protein	Periplasmic			o	tt
yfaU	CDS	predicted 2,4-dihydroxyhept-2-ene-1,7-dioic acid aldolase	Cytoplasmic	COG3836;2,4-dihydroxyhept-2-ene-1,7-dioic acid aldolase		o	o
yfaV	CDS	predicted transporter	Integral Membrane Protein			o	tt
yfaW	CDS	predicted enolase	Cytoplasmic	COG4948;L-alanine-DL-glutamate epimerase and related enzymes of enolase superfamily		gg	t
yfaX	CDS	predicted DNA-binding transcriptional regulator	Cytoplasmic	COG1414;Transcriptional regulator	GO:0006350 transcription -!- GO:0006355 regulation of transcription, DNA-dependent	gg	t
yfaY	CDS	conserved protein	Cytoplasmic	COG1058;Predicted nucleotide-utilizing enzyme related to molybdopterin-biosynthesis enzyme MoeA		g	tt
yfaZ	CDS	predicted outer membrane porin protein	Outer Membrane B-barrel protein				
yfbB	CDS	predicted peptidase	Cytoplasmic	COG0596;Predicted hydrolases or acyltransferases (alpha/beta hydrolase superfamily)		gg	tt
yfbE	CDS	uridine 5'-(beta-1-threo-pentapyranosyl-4-ulose diphosphate) aminotransferase, PLP-dependent	Cytoplasmic				
yfbF	CDS	undecaprenyl phosphate-L-Ara4FN transferase	Integral Membrane Protein	COG0463;Glycosyltransferases involved in cell wall biogenesis		gg	tt
yfbG	CDS	fused UDP-L-Ara4N formyltransferase -!- UDP-GlcA C-4'-decarboxylase	Cytoplasmic	COG0223;Methionyl-tRNA formyltransferase -!- COG0451;Nucleoside-diphosphate-sugar epimerases		g	tt
yfbH	CDS	conserved protein	Cytoplasmic	COG0726;Predicted xylanase/chitin deacetylase		gg	t
yfbJ	CDS	predicted inner membrane protein	Integral Membrane Protein				
yfbK	CDS	conserved protein	Membrane Lipoprotein	COG2304;Uncharacterized protein containing a von Willebrand factor type A (vWA) domain		g	tt
yfbL	CDS	predicted peptidase	Membrane Anchored	COG2234;Predicted aminopeptidases		o	tt
yfbM	CDS	predicted protein	Cytoplasmic			gg	t
yfbN	CDS	predicted protein	Cytoplasmic			gg	t
yfbO	CDS	predicted protein	Cytoplasmic			g	t
yfbP	CDS	predicted protein	Cytoplasmic			gg	o
yfbQ	CDS	predicted aminotransferase	Cytoplasmic	COG0436;Aspartate/tyrosine/aromatic aminotransferase		gg	t
yfbR	CDS	deoxyribonucleoside 5'-monophosphatase	Cytoplasmic	COG1896;Predicted hydrolases of HD superfamily		gg	o
yfbS	CDS	predicted transporter	Integral Membrane Protein	COG0471;Di- and tricarboxylate transporters		o	tt
yfbT	CDS	predicted hydrolase or phosphatase	Cytoplasmic	COG0637;Predicted phosphatase/phosphohexomutase			
yfbU	CDS	conserved protein	Cytoplasmic	COG3013;Uncharacterized conserved protein		g	o
yfbV	CDS	conserved inner membrane protein	Integral Membrane Protein	COG3092;Uncharacterized protein conserved in bacteria		o	tt
yfbW	CDS	conserved protein	Integral Membrane Protein			o	tt
yfcA	CDS	conserved inner membrane protein	Integral Membrane Protein	COG0730;Predicted permeases		o	tt
yfcC	CDS	predicted inner membrane protein	Integral Membrane Protein	COG1288;Predicted membrane protein		o	tt
yfcD	CDS	predicted NUDIX hydrolase	Cytoplasmic	COG0494;NTP pyrophosphohydrolases including oxidative damage repair enzymes		gg	tt
yfcE	CDS	predicted phosphatase	Cytoplasmic				
yfcF	CDS	predicted enzyme	Cytoplasmic	COG0625;Glutathione S-transferase		g	o
yfcG	CDS	predicted glutathione S-transferase	Cytoplasmic	COG0625;Glutathione S-transferase		gg	o
yfcH	CDS	conserved protein with NAD(P)-binding Rossmann-fold domain	Cytoplasmic	COG1090;Predicted nucleoside-diphosphate sugar epimerase		gg	tt
yfcI	CDS	conserved protein	Cytoplasmic			gg	t
yfcJ	CDS	predicted transporter	Integral Membrane Protein	COG0477;Permeases of the major facilitator superfamily		o	tt
yfcL	CDS	predicted protein	Cytoplasmic			gg	tt
yfcM	CDS	conserved protein	Cytoplasmic	COG3101;Uncharacterized protein conserved in bacteria			
yfcN	CDS	conserved protein	Cytoplasmic	COG2840;Uncharacterized protein conserved in bacteria		gg	t
yfcO	CDS	predicted protein	Periplasmic			o	tt
yfcP	CDS	predicted fimbrial-like adhesin protein	Periplasmic	COG3539;P pilus assembly protein, pilin FimA		o	tt
yfcQ	CDS	predicted fimbrial-like adhesin protein	Periplasmic	COG3539;P pilus assembly protein, pilin FimA		o	tt
yfcR	CDS	predicted fimbrial-like adhesin protein	Periplasmic	COG3539;P pilus assembly protein, pilin FimA		o	tt
yfcS	CDS	predicted periplasmic pilus chaperone	Periplasmic	COG3121;P pilus assembly protein, chaperone PapD	GO:0006457 protein folding	o	tt
yfcT	CDS	predicted outer membrane export usher protein	Cytoplasmic			gg	o
yfcU	CDS	predicted export usher protein	Periplasmic			o	tt
yfcV	CDS	predicted fimbrial-like adhesin protein	Periplasmic	COG3539;P pilus assembly protein, pilin FimA		o	tt
yfcX	CDS	fused enoyl-CoA hydratase-!-epimerase-!-isomerase -!- 3-hydroxyacyl-CoA dehydrogenase, subunit of anaerobic fatty acid oxidation complex	Cytoplasmic	COG1024;Enoyl-CoA hydratase/carnithine racemase -!- COG1250;3-hydroxyacyl-CoA dehydrogenase	GO:0019395 fatty acid oxidation	o	tt
yfcY	CDS	beta-ketoacyl-CoA thiolase, anaerobic, subunit	Cytoplasmic	COG0183;Acetyl-CoA acetyltransferase		o	o
yfcZ	CDS	conserved protein	Cytoplasmic	COG3691;Uncharacterized protein conserved in bacteria		gg	t
yfdC	CDS	predicted inner membrane protein	Integral Membrane Protein	COG2116;Formate/nitrite family of transporters		o	tt
yfdE	CDS	predicted CoA-transferase, NAD(P)-binding	Cytoplasmic	COG1804;Predicted acyl-CoA transferases/carnitine dehydratase		gg	t
yfdF	CDS	predicted protein	Cytoplasmic		GO:0006259 DNA metabolism	gg	o
yfdG	CDS	CPS-53 (KpLE1) prophage; bactoprenol-linked glucose translocase (flippase)	Integral Membrane Protein	COG2246;Predicted membrane protein		o	tt
yfdH	CDS	CPS-53 (KpLE1) prophage; bactoprenol glucosyl transferase	Integral Membrane Protein	COG0463;Glycosyltransferases involved in cell wall biogenesis		gg	tt
yfdI	CDS	CPS-53 (KpLE1) prophage; predicted inner membrane protein	Integral Membrane Protein				
yfdK	CDS	CPS-53 (KpLE1) prophage; conserved protein	Cytoplasmic			gg	t
yfdL	CDS	CPS-53 (KpLE1) prophage; conserved protein	Cytoplasmic				
yfdM	CDS	CPS-53 (KpLE1) prophage; predicted methyltransferase	Cytoplasmic			gg	t
yfdN	CDS	CPS-53 (KpLE1) prophage; predicted protein	Cytoplasmic				
yfdO	CDS	CPS-53 (KpLE1) prophage; predicted defective phage replication protein O	Cytoplasmic			gg	t
yfdP	CDS	CPS-53 (KpLE1) prophage; predicted protein	Cytoplasmic			o	tt
yfdQ	CDS	CPS-53 (KpLE1) prophage; predicted protein	Cytoplasmic			g	o
yfdR	CDS	CPS-53 (KpLE1) prophage; conserved protein	Cytoplasmic	COG1896;Predicted hydrolases of HD superfamily		gg	t
yfdS	CDS	CPS-53 (KpLE1) prophage; predicted protein	Cytoplasmic			gg	o
yfdT	CDS	CPS-53 (KpLE1) prophage; predicted protein	Cytoplasmic				
yfdV	CDS	predicted transporter	Integral Membrane Protein	COG0679;Predicted permeases			
yfdX	CDS	predicted protein	Periplasmic			o	tt
yfdY	CDS	predicted inner membrane protein	Integral Membrane Protein			o	tt
yfdZ	CDS	prediected aminotransferase, PLP-dependent	Cytoplasmic	COG0436;Aspartate/tyrosine/aromatic aminotransferase		gg	t
yfeA	CDS	predicted diguanylate cyclase	Integral Membrane Protein	COG2199;FOG: GGDEF domain -!- COG2200;FOG: EAL domain			
yfeC	CDS	predicted DNA-binding transcriptional regulator	Cytoplasmic			gg	tt
yfeD	CDS	predicted DNA-binding transcriptional regulator	Cytoplasmic			o	tt
yfeG	CDS	predicted DNA-binding transcriptional regulator	Cytoplasmic	COG2207;AraC-type DNA-binding domain-containing proteins	GO:0009310 amine catabolism -!- GO:0006350 transcription	gg	t
yfeH	CDS	predicted inner membrane protein	Integral Membrane Protein	COG0385;Predicted Na+-dependent transporter			
yfeK	CDS	predicted protein	Periplasmic			o	tt
yfeN	CDS	conserved outer membrane protein	Outer Membrane B-barrel protein			o	tt
yfeO	CDS	predicted ion channel protein	Integral Membrane Protein	COG0038;Chloride channel protein EriC		o	tt
yfeR	CDS	predicted DNA-binding transcriptional regulator	Cytoplasmic	COG0583;Transcriptional regulator	GO:0006350 transcription	o	tt
yfeS	CDS	conserved protein	Cytoplasmic	COG3831;Uncharacterized conserved protein -!- COG4884;Uncharacterized protein conserved in bacteria		g	tt
yfeT	CDS	predicted DNA-binding transcriptional regulator	Cytoplasmic	COG1737;Transcriptional regulators		o	tt
yfeU	CDS	predicted PTS component	Cytoplasmic	COG2103;Predicted sugar phosphate isomerase			
yfeW	CDS	predicted periplasmic esterase	Periplasmic	COG1680;Beta-lactamase class C and other penicillin binding proteins	GO:0016052 carbohydrate		
yfeX	CDS	conserved protein	Cytoplasmic			gg	o
yfeY	CDS	predicted protein	Outer Membrane Lipoprotein			o	tt
yfeZ	CDS	predicted inner membrane protein	Integral Membrane Protein			o	tt
yffB	CDS	conserved protein	Cytoplasmic	COG1393;Arsenate reductase and related proteins, glutaredoxin family		gg	tt
yffH	CDS	predicted NUDIX hydrolase	Cytoplasmic	COG0494;NTP pyrophosphohydrolases including oxidative damage repair enzymes		gg	t
yffI	CDS	predicted carboxysome structural protein with predicted role in ethanolamine utilization	Cytoplasmic		GO:0009310 amine catabolism	gg	t
yffL	CDS	CPZ-55 prophage; predicted protein	Cytoplasmic				
yffM	CDS	CPZ-55 prophage; predicted protein	Cytoplasmic				
yffN	CDS	CPZ-55 prophage; predicted protein	Cytoplasmic				
yffO	CDS	CPZ-55 prophage; predicted protein	Cytoplasmic				
yffP	CDS	CPZ-55 prophage; predicted protein	Cytoplasmic				
yffQ	CDS	CPZ-55 prophage; predicted protein	Periplasmic				
yffR	CDS	CPZ-55 prophage; predicted protein	Cytoplasmic				
yffS	CDS	CPZ-55 prophage; predicted protein	Cytoplasmic				
yfgA	CDS	conserved protein	Membrane Anchored	COG1426;Uncharacterized protein conserved in bacteria		o	tt
yfgB	CDS	predicted enzyme	Cytoplasmic	COG0820;Predicted Fe-S-cluster redox enzyme		gg	t
yfgC	CDS	predicted peptidase	Periplasmic	COG4783;Putative Zn-dependent protease, contains TPR repeats			
yfgD	CDS	predicted oxidoreductase	Cytoplasmic	COG1393;Arsenate reductase and related proteins, glutaredoxin family	GO:0006805 xenobiotic metabolism	o	o
yfgF	CDS	predicted inner membrane protein	Integral Membrane Protein	COG2199;FOG: GGDEF domain -!- COG2200;FOG: EAL domain		o	tt
yfgG	CDS	predicted protein	Membrane Anchored				
yfgH	CDS	predicted outer membrane lipoprotein	Outer Membrane Lipoprotein				
yfgI	CDS	conserved protein	Periplasmic			gg	tt
yfgJ	CDS	predicted protein	Cytoplasmic		GO:0042966 biotin carboxyl carrier protein biosynthesis		

yfgL	CDS	protein assembly complex, lipoprotein component	Outer Membrane Lipoprotein	COG1520;FOG: WD40-like repeat	GO:0006464 protein modification	o	tt
yfgM	CDS	conserved protein	Membrane Anchored	COG2976;Uncharacterized protein conserved in bacteria			
yfgO	CDS	predicted inner membrane protein	Integral Membrane Protein	COG0628;Predicted permease		o	tt
yfhA	CDS	predicted DNA-binding response regulator in two-component system	Cytoplasmic	COG2204;Response regulator containing CheY-like receiver, AAA-type ATPase, and DNA-binding domains	GO:0006464 protein modification	gg	t
yfhB	CDS	conserved protein	Membrane Anchored				
yfhD	CDS	predicted transglycosylase	Periplasmic	COG4623;Predicted soluble lytic transglycosylase fused to an ABC-type amino acid-binding protein		gg	t
yfhG	CDS	conserved protein	Outer Membrane Lipoprotein			gg	tt
yfhH	CDS	predicted DNA-binding transcriptional regulator	Cytoplasmic	COG1737;Transcriptional regulators		o	tt
yfhJ	CDS	conserved protein	Cytoplasmic	COG2975;Uncharacterized protein conserved in bacteria	GO:0006457 protein folding	gg	o
yfhK	CDS	predicted sensory kinase in two-component system	Integral Membrane Protein	COG0642;Signal transduction histidine kinase	GO:0006464 protein modification		
yfhL	CDS	predicted 4Fe-4S cluster-containing protein	Cytoplasmic	COG1145;Ferredoxin		gg	t
yfhM	CDS	conserved protein	Inner Membrane Lipoprotein	COG2373;Large extracellular alpha-helical protein		o	tt
yfhQ	CDS	predicted methyltransferase	Cytoplasmic	COG0565;rRNA methylase		gg	t
yfhR	CDS	predicted peptidase	Membrane Anchored			o	tt
yfiA	CDS	cold shock protein associated with 30S ribosomal subunit	Cytoplasmic	COG1544;Ribosome-associated protein Y (PSrp-1)	GO:0006412 protein biosynthesis	gg	t
yfiB	CDS	predicted outer membrane lipoprotein	Outer Membrane Lipoprotein	COG2885;Outer membrane protein and related peptidoglycan-associated (lipo)proteins		o	tt
yfiC	CDS	predicted methyltransferase	Cytoplasmic	COG4123;Predicted O-methyltransferase		gg	t
yfiD	CDS	pyruvate formate lyase subunit	Cytoplasmic	COG3445;Acid-induced glycyl radical enzyme	GO:0009061 anaerobic respiration	gg	t
yfiE	CDS	predicted DNA-binding transcriptional regulator	Cytoplasmic		GO:0006350 transcription	g	t
yfiF	CDS	predicted methyltransferase	Cytoplasmic	COG0566;rRNA methylases	GO:0009451 RNA modification	g	tt
yfiH	CDS	conserved protein	Cytoplasmic	COG1496;Uncharacterized conserved protein		g	o
yfiK	CDS	neutral amino-acid efflux system	Integral Membrane Protein	COG1280;Putative threonine efflux protein		o	tt
yfiL	CDS	predicted protein	Outer Membrane Lipoprotein				
yfiM	CDS	predicted protein	Cytoplasmic			o	t
yfiN	CDS	predicted diguanylate cyclase	Integral Membrane Protein	COG2199;FOG: GGDEF domain			
yfiO	CDS	predicted lipoprotein	Outer Membrane Lipoprotein	COG4105;DNA uptake lipoprotein		o	tt
yfiP	CDS	conserved protein	Cytoplasmic				
yfiQ	CDS	fused predicted acyl-CoA synthetase NAD(P)-binding subunit -!- ATP-binding subunit	Cytoplasmic	COG1042;Acyl-CoA synthetase (NDP forming) -!- COG0454;Histone acetyltransferase HPA2 and related acetyltransferases		gg	tt
yfiR	CDS	predicted protein	Periplasmic			o	tt
yfjD	CDS	predicted inner membrane protein	Integral Membrane Protein				
yfjF	CDS	predicted protein	Cytoplasmic			gg	o
yfjG	CDS	conserved protein	Cytoplasmic	COG2867;Oligoketide cyclase/lipid transport protein		o	tt
yfjH	CDS	CP4-57 prophage; predicted protein	Cytoplasmic			g	tt
yfjI	CDS	CP4-57 prophage; predicted protein	Cytoplasmic			o	tt
yfjJ	CDS	CP4-57 prophage; predicted protein	Cytoplasmic			gg	o
yfjK	CDS	CP4-57 prophage; conserved protein	Cytoplasmic	COG1204;Superfamily II helicase		gg	o
yfjL	CDS	CP4-57 prophage; predicted protein	Cytoplasmic			o	t
yfjM	CDS	CP4-57 prophage; predicted protein	Cytoplasmic			gg	o
yfjN	CDS	CP4-57 prophage; predicted protein	Cytoplasmic			g	t
yfjO	CDS	CP4-57 prophage; predicted protein	Cytoplasmic				
yfjP	CDS	CP4-57 prophage; predicted GTP-binding protein	Cytoplasmic				
yfjQ	CDS	CP4-57 prophage; predicted protein	Cytoplasmic			g	t
yfjR	CDS	CP4-57 prophage; predicted DNA-binding transcriptional regulator	Cytoplasmic	COG2378;Predicted transcriptional regulator		o	tt
yfjS	CDS	CP4-57 prophage; predicted protein	Inner Membrane Lipoprotein				
yfjT	CDS	CP4-57 prophage; predicted protein	Periplasmic			o	tt
yfjU	CDS	CP4-57 prophage; conserved protein	Cytoplasmic	COG1393;Arsenate reductase and related proteins, glutaredoxin family		g	o
yfjV	CDS	CP4-57 prophage; predicted protein	Cytoplasmic			o	tt
yfjW	CDS	CP4-57 prophage; predicted inner membrane protein	Integral Membrane Protein			o	tt
yfjX	CDS	CP4-57 prophage; predicted antirestriction protein	Cytoplasmic			gg	t
yfjY	CDS	CP4-57 prophage; predicted DNA repair protein	Cytoplasmic	COG2003;DNA repair proteins		gg	t
yfjZ	CDS	CP4-57 prophage; antitoxin of the YpjF-YfjZ toxin-antitoxin system	Cytoplasmic			gg	t
ygaC	CDS	predicted protein	Cytoplasmic			gg	o
ygaD	CDS	conserved protein	Cytoplasmic	COG1546;Uncharacterized protein (competence- and mitomycin-induced)		gg	t
ygaF	CDS	predicted enzyme	Cytoplasmic	COG0579;Predicted dehydrogenase		gg	t
ygaH	CDS	predicted inner membrane protein	Integral Membrane Protein			o	tt
ygaM	CDS	predicted protein	Membrane Anchored			g	t
ygaP	CDS	predicted inner membrane protein with hydrolase activity	Integral Membrane Protein	COG0607;Rhodanese-related sulfurtransferase		o	tt
ygaQ	CDS	predicted protein	Cytoplasmic				
ygaR	CDS	predicted protein	Cytoplasmic			o	tt
ygaT	CDS	predicted protein	Cytoplasmic				
ygaU	CDS	predicted protein	Cytoplasmic	COG1652;Uncharacterized protein containing LysM domain		gg	t
ygaV	CDS	predicted DNA-binding transcriptional regulator	Cytoplasmic	COG0640;Predicted transcriptional regulators		o	tt
ygaW	CDS	predicted inner membrane protein	Integral Membrane Protein			o	tt
ygaX	CDS	predicted transporter	Integral Membrane Protein		GO:0006950 response to stress	o	tt
ygaY	CDS	predicted transporter (pseudogene)	Integral Membrane Protein				
ygaZ	CDS	predicted transporter	Integral Membrane Protein	COG1296;Predicted branched-chain amino acid permease (azaleucine resistance)		o	tt
ygbA	CDS	predicted protein	Cytoplasmic			gg	tt
ygbE	CDS	conserved inner membrane protein	Integral Membrane Protein			o	tt
ygbF	CDS	predicted protein	Cytoplasmic				
ygbI	CDS	predicted DNA-binding transcriptional regulator	Cytoplasmic	COG1349;Transcriptional regulators of sugar metabolism	GO:0006350 transcription	o	tt
ygbJ	CDS	predicted dehydrogenase, with NAD(P)-binding Rossmann-fold domain	Membrane Anchored	COG2084;3-hydroxyisobutyrate dehydrogenase and related beta-hydroxyacid dehydrogenases		gg	t
ygbK	CDS	conserved protein	Cytoplasmic	COG3395;Uncharacterized protein conserved in bacteria		gg	t
ygbL	CDS	predicted class II aldolase	Cytoplasmic	COG0235;Ribulose-5-phosphate 4-epimerase and related epimerases and aldolases		gg	t
ygbM	CDS	conserved protein	Cytoplasmic	COG3622;Hydroxypyruvate isomerase		g	t
ygbN	CDS	predicted transporter	Integral Membrane Protein	COG2610;H+/gluconate symporter and related permeases		o	tt
ygbT	CDS	conserved protein	Cytoplasmic	COG1518;Uncharacterized protein predicted to be involved in DNA repair		gg	tt
ygcB	CDS	conserved protein, member of DEAD box family	Cytoplasmic	COG1203;Predicted helicases		g	o
ygcE	CDS	predicted kinase	Cytoplasmic	COG1070;Sugar (pentulose and hexulose) kinases			
ygcF	CDS	conserved protein	Cytoplasmic	COG0602;Organic radical activating enzymes	GO:0046138 coenzymes and prosthetic group biosynthesis	gg	t
ygcG	CDS	predicted protein	Integral Membrane Protein				
ygcH	CDS	predicted protein	Cytoplasmic			g	t
ygcI	CDS	predicted protein	Cytoplasmic				
ygcJ	CDS	predicted protein	Cytoplasmic			gg	t
ygcK	CDS	predicted protein	Cytoplasmic			gg	t
ygcL	CDS	predicted protein	Cytoplasmic			gg	o
ygcM	CDS	6-pyruvoyl tetrahydrobiopterin synthase (PTPS)	Cytoplasmic	COG0720;6-pyruvoyl-tetrahydropterin synthase		o	o
ygcN	CDS	predicted oxidoreductase, FAD/NAD(P)-binding domain	Cytoplasmic			g	t
ygcO	CDS	predicted 4Fe-4S cluster-containing protein	Cytoplasmic	COG2440;Ferredoxin-like protein		gg	t
ygcP	CDS	predicted anti-terminator regulatory protein	Cytoplasmic	COG1954;Glycerol-3-phosphate responsive antiterminator (mRNA-binding)		gg	t
ygcQ	CDS	predicted flavoprotein	Cytoplasmic				
ygcR	CDS	predicted flavoprotein	Cytoplasmic				
ygcS	CDS	predicted transporter	Integral Membrane Protein	COG0477;Permeases of the major facilitator superfamily			
ygcU	CDS	predicted FAD containing dehydrogenase	Cytoplasmic				
ygcW	CDS	predicted deoxygluconate dehydrogenase	Cytoplasmic				
ygdB	CDS	predicted protein	Periplasmic				
ygdD	CDS	conserved inner membrane protein	Integral Membrane Protein	COG2363;Uncharacterized small membrane protein		o	tt
ygdE	CDS	predicted methyltransferase	Cytoplasmic	COG2933;Predicted SAM-dependent methyltransferase		gg	tt
ygdH	CDS	conserved protein	Cytoplasmic	COG1611;Predicted Rossmann fold nucleotide-binding protein		gg	t
ygdI	CDS	predicted protein	Outer Membrane Lipoprotein				
ygdK	CDS	predicted Fe-S metabolism protein	Cytoplasmic	COG2166;SufE protein probably involved in Fe-S center assembly		gg	t
ygdL	CDS	conserved protein	Membrane Anchored	COG1179;Dinucleotide-utilizing enzymes involved in molybdopterin and thiamine biosynthesis family 1		gg	tt
ygdQ	CDS	predicted inner membrane protein	Integral Membrane Protein	COG0861;Membrane protein TerC, possibly involved in tellurium resistance		o	tt
ygdR	CDS	predicted protein	Outer Membrane Lipoprotein			o	tt
ygeA	CDS	predicted racemase	Cytoplasmic	COG1794;Aspartate racemase	GO:0042493 response to drug	o	tt
ygeD	CDS	predicted inner membrane protein	Integral Membrane Protein	COG0477;Permeases of the major facilitator superfamily	GO:0042493 response to drug	o	tt
ygeF	CDS	predicted protein	Cytoplasmic			g	o
ygeG	CDS	predicted chaperone	Cytoplasmic	COG0457;FOG: TPR repeat		gg	t
ygeH	CDS	predictedtranscriptional regulator	Cytoplasmic	COG3710;DNA-binding winged-HTH domains -!- COG0457;FOG: TPR repeat		g	o
ygeI	CDS	predicted protein	Cytoplasmic				
ygeK	CDS	predicted DNA-binding transcriptional regulator	Cytoplasmic	COG2197;Response regulator containing a CheY-like receiver domain and an HTH DNA-binding domain	GO:0006350 transcription		
ygeL	CDS	predicted protein	Cytoplasmic	COG2197;Response regulator containing a CheY-like receiver domain and an HTH DNA-binding domain		gg	o
ygeM	CDS	predicted protein	Cytoplasmic				
ygeN	CDS	predicted protein (pseudogene)	Cytoplasmic				
ygeO	CDS	predicted protein	Cytoplasmic				
ygeP	CDS	predicted protein	Cytoplasmic			g	o

ygeQ	CDS	predicted protein	Periplasmic				
ygeR	CDS	Tetratricopeptide repeat transcriptional regulator	Outer Membrane Lipoprotein			o	tt
ygeV	CDS	predicted DNA-binding transcriptional regulator	Cytoplasmic	COG3829;Transcriptional regulator containing PAS, AAA-type ATPase, and DNA-binding domains	GO:0006350 transcription	gg	t
ygeW	CDS	conserved protein	Cytoplasmic				
ygeX	CDS	2,3-diaminopropionate ammonia-lyase	Cytoplasmic	COG1171;Threonine dehydratase		gg	o
ygeY	CDS	predicted peptidase	Cytoplasmic	COG0624;Acetylornithine deacetylase/Succinyl-diaminopimelate desuccinylase and related deacylases		gg	tt
ygfA	CDS	predicted ligase	Cytoplasmic			gg	o
ygfB	CDS	predicted protein	Cytoplasmic				
ygfF	CDS	predicted NAD(P)-binding oxidoreductase with NAD(P)-binding Rossmann-fold domain	Cytoplasmic	COG1028;Dehydrogenases with different specificities (related to short-chain alcohol dehydrogenases)		gg	t
ygfG	CDS	methylmalonyl-CoA decarboxylase, biotin-	Cytoplasmic			gg	t
ygfH	CDS	propionyl-CoA:succinate-CoA transferase	Cytoplasmic	COG0427;Acetyl-CoA hydrolase		gg	t
ygfI	CDS	predicted DNA-binding transcriptional regulator	Cytoplasmic		GO:0006350 transcription		
ygfJ	CDS	conserved protein	Cytoplasmic	COG2068;Uncharacterized MobA-related protein		g	o
ygfK	CDS	predicted oxidoreductase, Fe-S subunit	Cytoplasmic	COG0493;NADPH-dependent glutamate synthase beta chain and related oxidoreductases			
ygfM	CDS	predicted oxidoreductase	Cytoplasmic	COG1319;Aerobic-type carbon monoxide dehydrogenase, middle subunit CoxM/CutM homologs		o	t
ygfO	CDS	predicted transporter	Integral Membrane Protein	COG2233;Xanthine/uracil permeases		o	tt
ygfQ	CDS	predicted transporter	Integral Membrane Protein				
ygfS	CDS	predicted oxidoreductase, 4Fe-4S ferredoxin-type subunit	Cytoplasmic				
ygfT	CDS	fused predicted oxidoreductase Fe-S subunit -!- nucleotide-binding subunit	Cytoplasmic	COG1142;Fe-S-cluster-containing hydrogenase components 2 -!- COG0493;NADPH-dependent glutamate synthase beta chain and related oxidoreductases			
ygfU	CDS	predicted transporter	Integral Membrane Protein	COG2233;Xanthine/uracil permeases			
ygfX	CDS	predicted protein	Membrane Anchored			o	tt
ygfY	CDS	conserved protein	Cytoplasmic	COG2938;Uncharacterized conserved protein		gg	o
ygfZ	CDS	predicted folate-dependent regulatory protein	Cytoplasmic	COG0354;Predicted aminomethyltransferase related to GcvT		gg	o
yggC	CDS	conserved protein with nucleoside triphosphate hydrolase domain	Cytoplasmic	COG1072;Panthothenate kinase		gg	tt
yggD	CDS	predicted DNA-binding transcriptional regulator	Cytoplasmic	COG3722;Transcriptional regulator		gg	t
yggE	CDS	conserved protein	Periplasmic	COG2968;Uncharacterized conserved protein		o	tt
yggF	CDS	predicted hexoseP phosphatase	Cytoplasmic	COG1494;Fructose-1,6-bisphosphatase/sedoheptulose 1,7-bisphosphatase and related proteins		g	t
yggG	CDS	predicted peptidase	Outer Membrane Lipoprotein	COG0501;Zn-dependent protease with chaperone function		gg	tt
yggH	CDS	tRNA (m7G46) methyltransferase, SAM-dependent	Cytoplasmic	COG0220;Predicted S-adenosylmethionine-dependent methyltransferase		gg	t
yggJ	CDS	predicted protein	Cytoplasmic			g	t
yggL	CDS	predicted protein	Cytoplasmic			o	tt
yggM	CDS	conserved protein	Periplasmic			o	tt
yggN	CDS	predicted protein	Periplasmic			o	tt
yggP	CDS	predicted dehydrogenase	Cytoplasmic				
yggR	CDS	predicted transporter	Cytoplasmic	COG2805;Tfp pilus assembly protein, pilus retraction ATPase PilT		o	tt
yggS	CDS	predicted enzyme	Cytoplasmic	COG0325;Predicted enzyme with a TIM-barrel fold		o	tt
yggT	CDS	predicted inner membrane protein	Integral Membrane Protein	COG0762;Predicted integral membrane protein		o	tt
yggU	CDS	conserved protein	Cytoplasmic	COG1872;Uncharacterized conserved protein			
yggV	CDS	dITP/XTP pyrophosphatase	Cytoplasmic	COG0127;Xanthosine triphosphate pyrophosphatase		gg	o
yggW	CDS	predicted oxidoreductase	Cytoplasmic	COG0635;Coproporphyrinogen III oxidase and related Fe-S		g	tt
yggX	CDS	protein that protects iron-sulfur proteins against oxidative damage	Cytoplasmic	COG2924;Fe-S cluster protector protein		gg	o
yghA	CDS	predicted glutathionylspermidine synthase, with NAD(P)-binding Rossmann-fold domain	Cytoplasmic	COG1028;Dehydrogenases with different specificities (related to short-chain alcohol dehydrogenases)		g	o
yghB	CDS	conserved inner membrane protein	Integral Membrane Protein	COG0586;Uncharacterized membrane-associated protein		o	tt
yghD	CDS	predicted secretion pathway M-type protein, membrane anchored	Membrane Anchored	COG3149;Type II secretory pathway, component PulM		g	tt
yghE	CDS	predicted secretion pathway protein, L-type protein (pseudogene)	Cytoplasmic	COG3297;Type II secretory pathway, component PulL			
yghF	CDS	predicted secretion pathway protein, C-type protein	Cytoplasmic	COG3031;Type II secretory pathway, component PulC			
yghG	CDS	predicted protein	Outer Membrane Lipoprotein			o	tt
yghJ	CDS	predicted inner membrane lipoprotein	Inner Membrane Lipoprotein				
yghK	CDS	glycolate transporter	Integral Membrane Protein	COG1620;L-lactate permease	GO:0016052 carbohydrate	o	tt
yghO	CDS	predicted DNA-binding transcriptional regulator	Cytoplasmic				
yghQ	CDS	predicted inner membrane protein	Integral Membrane Protein				
yghR	CDS	predicted protein with nucleoside triphosphate hydrolase domain	Cytoplasmic			o	tt
yghS	CDS	predicted protein with nucleoside triphosphate hydrolase domain	Cytoplasmic				
yghT	CDS	predicted protein with nucleoside triphosphate hydrolase domain	Cytoplasmic			g	t
yghU	CDS	predicted S-transferase	Membrane Anchored				
yghW	CDS	predicted protein	Cytoplasmic			gg	o
yghX	CDS	predicted hydrolase (pseudogene)	Cytoplasmic				
yghY	CDS	predicted dienlactone hydrolase (pseudogene)	Cytoplasmic				
yghZ	CDS	aldo-keto reductase	Cytoplasmic	COG0667;Predicted oxidoreductases (related to aryl-alcohol dehydrogenases)		gg	t
ygiA	CDS	predicted protein	Cytoplasmic				
ygiB	CDS	conserved outer membrane protein	Cytoplasmic				
ygiC	CDS	predicted enzyme	Cytoplasmic	COG0754;Glutathionylspermidine synthase		o	o
ygiE	CDS	zinc transporter	Integral Membrane Protein	COG0428;Predicted divalent heavy-metal cations transporter		o	tt
ygiF	CDS	predicted adenylate cyclase	Cytoplasmic	COG3025;Uncharacterized conserved protein		g	t
ygiH	CDS	conserved inner membrane protein	Integral Membrane Protein	COG0344;Predicted membrane protein		o	tt
ygiL	CDS	predicted fimbrial-like adhesin protein	Periplasmic	COG3539;P pilus assembly protein, pilin FimA		gg	tt
ygiN	CDS	quinol monooxygenase	Cytoplasmic	COG1359;Uncharacterized conserved protein		g	t
ygiP	CDS	predicted DNA-binding transcriptional regulator	Cytoplasmic	COG0583;Transcriptional regulator	GO:0006350 transcription	g	tt
ygiQ	CDS	conserved protein	Cytoplasmic				
ygiS	CDS	predicted transporter subunit -!- periplasmic-binding component of ABC superfamily	Periplasmic	COG4166;ABC-type oligopeptide transport system, periplasmic component		o	tt
ygiT	CDS	predicted DNA-binding transcriptional regulator	Cytoplasmic	COG1396;Predicted transcriptional regulators			
ygiU	CDS	predicted cyanide hydratase	Cytoplasmic				
ygiV	CDS	predicted transcriptional regulator	Cytoplasmic				
ygiW	CDS	conserved protein	Periplasmic	COG3111;Uncharacterized conserved protein		o	tt
ygiZ	CDS	conserved inner membrane protein	Integral Membrane Protein				
ygjD	CDS	predicted peptidase	Cytoplasmic	COG0533;Metal-dependent proteases with possible chaperone activity		gg	t
ygjE	CDS	predicted tartrate:succinate antiporter	Integral Membrane Protein	COG0471;Di- and tricarboxylate transporters		o	tt
ygjF	CDS	G/U mismatch-specific DNA glycosylase	Cytoplasmic	COG3663;G:T/U mismatch-specific DNA glycosylase	GO:0006281 DNA repair	gg	t
ygjG	CDS	putrescine:2-oxoglutaric acid aminotransferase, PLP-dependent	Periplasmic		GO:0006526 arginine biosynthesis -!- GO:0008615 pyridoxine biosynthesis		
ygjH	CDS	conserved protein	Cytoplasmic	COG0073;EMAP domain	GO:0006418 amino acid activation	gg	o
ygjI	CDS	predicted transporter	Integral Membrane Protein				
ygjJ	CDS	conserved protein	Periplasmic			o	tt
ygjK	CDS	predicted glycosyl hydrolase	Periplasmic			g	tt
ygjM	CDS	predicted DNA-binding transcriptional regulator	Cytoplasmic		GO:0006355 regulation of transcription, DNA-dependent	gg	o
ygjN	CDS	conserved protein	Cytoplasmic	COG4680;Uncharacterized protein conserved in bacteria		o	t
ygjO	CDS	predicted methyltransferase small domain	Cytoplasmic				
ygjP	CDS	predicted metal dependent hydrolase	Cytoplasmic	COG1451;Predicted metal-dependent hydrolase			
ygjQ	CDS	predicted thioredoxin-like	Periplasmic	COG2949;Uncharacterized membrane protein		o	tt
ygjR	CDS	predicted NAD(P)-binding dehydrogenase	Cytoplasmic	COG0673;Predicted dehydrogenases and related proteins		gg	t
ygjV	CDS	conserved inner membrane protein	Integral Membrane Protein			o	tt
yhaB	CDS	predicted protein	Cytoplasmic			g	o
yhaC	CDS	predicted protein	Cytoplasmic			gg	o
yhaH	CDS	predicted inner membrane protein	Integral Membrane Protein	COG3152;Predicted membrane protein		o	tt
yhaI	CDS	predicted inner membrane protein	Integral Membrane Protein	COG3152;Predicted membrane protein		o	tt
yhaJ	CDS	predicted DNA-binding transcriptional regulator	Cytoplasmic	COG0583;Transcriptional regulator	GO:0006350 transcription	o	tt
yhaK	CDS	predicted pirin-related protein	Cytoplasmic	COG1741;Pirin-related protein		g	t
yhaL	CDS	predicted protein	Cytoplasmic				
yhaM	CDS	conserved protein	Cytoplasmic				
yhaO	CDS	predicted transporter	Integral Membrane Protein				
yhaV	CDS	conserved protein	Cytoplasmic			o	tt
yhbC	CDS	conserved protein	Cytoplasmic				
yhbE	CDS	conserved inner membrane protein	Integral Membrane Protein	COG0697;Permeases of the drug/metabolite transporter (DMT) superfamily		o	tt
yhbG	CDS	predicted transporter subunit -!- ATP-binding component of ABC superfamily	Cytoplasmic	COG1137;ABC-type (unclassified) transport system, ATPase component		g	o
yhbH	CDS	predicted ribosome-associated, sigma 54 modulation protein	Cytoplasmic	COG1544;Ribosome-associated protein Y (PSrp-1)	GO:0006350 transcription	gg	o
yhbJ	CDS	predicted protein with nucleoside triphosphate hydrolase domain	Cytoplasmic	COG1660;Predicted P-loop-containing kinase		gg	o
yhbN	CDS	predicted transporter subunit -!- periplasmic-binding component of ABC superfamily	Periplasmic	COG1934;Uncharacterized protein conserved in bacteria		o	tt
yhbO	CDS	predicted intracellular protease	Cytoplasmic	COG0693;Putative intracellular protease/amidase			
yhbP	CDS	conserved protein	Cytoplasmic	COG3787;Uncharacterized protein conserved in bacteria		o	tt
yhbQ	CDS	predicted endonuclease	Cytoplasmic	COG2827;Predicted endonuclease containing a URI domain		gg	t
yhbS	CDS	predicted acyltransferase with acyl-CoA N-acyltransferase domain	Cytoplasmic	COG3153;Predicted acetyltransferase		gg	t

yhbT	CDS	predicted lipid carrier protein	Cytoplasmic	COG3154;Putative lipid carrier protein		o	tt
yhbU	CDS	predicted peptidase (collagenase-like)	Cytoplasmic	COG0826;Collagenase and related proteases		gg	t
yhbV	CDS	predicted protease	Cytoplasmic	COG0826;Collagenase and related proteases			
yhbW	CDS	predicted enzyme	Cytoplasmic	COG2141;Coenzyme F420-dependent N5,N10-methylene tetrahydromethanopterin reductase and related flavin-dependent oxidoreductases		g	t
yhbX	CDS	predicted hydrolase, inner membrane	Integral Membrane Protein				
yhbY	CDS	predicted RNA-binding protein	Cytoplasmic	COG1534;Predicted RNA-binding protein containing KH domain, possibly ribosomal protein		gg	t
yhcA	CDS	predicted periplasmic chaperone protein	Periplasmic	COG3121;P pilus assembly protein, chaperone PapD	GO:0006457 protein folding	o	tt
yhcB	CDS	conserved protein	Membrane Anchored	COG3105;Uncharacterized protein conserved in bacteria			
yhcC	CDS	predicted Fe-S oxidoreductase	Cytoplasmic	COG1242;Predicted Fe-S oxidoreductase		gg	t
yhcD	CDS	predicted outer membrane protein	Outer Membrane B-barrel protein	COG3188;P pilus assembly protein, porin PapC		o	tt
yhcE	CDS	predicted protein, N-ter fragment (pseudogene)	Periplasmic			g	o
yhcE	ancestral	predicted protein (pseudogene)	Periplasmic				
yhcE	CDS	predicted protein, C-ter fragment (pseudogene)	Cytoplasmic			g	o
yhcF	CDS	predicted transcriptional regulator	Periplasmic		GO:0006350 transcription -!- GO:0006355 regulation of transcription, DNA-dependent	o	tt
yhcG	CDS	conserved protein	Cytoplasmic	COG4804;Uncharacterized conserved protein		o	t
yhcH	CDS	conserved protein	Cytoplasmic	COG2731;Beta-galactosidase, beta subunit		g	o
yhcM	CDS	conserved protein with nucleoside triphosphate hydrolase domain	Cytoplasmic	COG1485;Predicted ATPase		o	tt
yhcN	CDS	conserved protein	Periplasmic				
yhcO	CDS	predicted barnase inhibitor	Cytoplasmic	COG2732;Barstar, RNAse (barnase) inhibitor		gg	t
yhdA	CDS	conserved inner membrane protein	Integral Membrane Protein	COG2199;FOG: GGDEF domain -!- COG2200;FOG: EAL domain		o	tt
yhdE	CDS	conserved protein	Cytoplasmic	COG0424;Nucleotide-binding protein implicated in inhibition of septum formation		gg	t
yhdH	CDS	predicted oxidoreductase, Zn-dependent and NAD(P)-binding	Cytoplasmic	COG0604;NADPH:quinone reductase and related Zn-dependent oxidoreductases		gg	t
yhdJ	CDS	predicted methyltransferase	Cytoplasmic				
yhdL	CDS	conserved protein	Cytoplasmic			o	o
yhdN	CDS	conserved protein	Cytoplasmic			gg	t
yhdP	CDS	conserved membrane protein, predicted transporter	Periplasmic				
yhdT	CDS	conserved inner membrane protein	Integral Membrane Protein	COG3924;Predicted membrane protein		o	tt
yhdU	CDS	predicted membrane protein	Membrane Anchored			o	tt
yhdV	CDS	predicted outer membrane protein	Outer Membrane Lipoprotein			o	tt
yhdW	CDS	predicted amino-acid transporter subunit -!- periplasmic-binding component of ABC superfamily	Cytoplasmic	COG0834;ABC-type amino acid transport/signal transduction systems, periplasmic component/domain	GO:0009063 amino acid catabolism	g	o
yhdX	CDS	predicted amino-acid transporter subunit -!- membrane component of ABC superfamily	Integral Membrane Protein				
yhdY	CDS	predicted amino-acid transporter subunit -!- membrane component of ABC superfamily	Integral Membrane Protein	COG0765;ABC-type amino acid transport system, permease component			
yhdZ	CDS	predicted amino-acid transporter subunit -!- ATP-binding component of ABC superfamily	Cytoplasmic	COG1126;ABC-type polar amino acid transport system, ATPase component		g	t
yheL	CDS	predicted intracellular sulfur oxidation protein	Cytoplasmic	COG2168;Uncharacterized conserved protein involved in oxidation of intracellular sulfur		g	t
yheM	CDS	predicted intraceullular sulfur oxidation protein	Cytoplasmic	COG2923;Uncharacterized protein involved in the oxidation of intracellular sulfur		g	o
yheN	CDS	predicted intracellular sulfur oxidation protein	Cytoplasmic	COG1553;Uncharacterized conserved protein involved in intracellular sulfur reduction		g	t
yheO	CDS	predicted DNA-binding transcriptional regulator	Cytoplasmic				
yheS	CDS	fused predicted transporter subunits -!- ATP-binding components of ABC superfamily	Cytoplasmic	COG0488;ATPase components of ABC transporters with duplicated ATPase domains		gg	o
yheT	CDS	predicted hydrolase	Cytoplasmic	COG0429;Predicted hydrolase of the alpha/beta-hydrolase fold		o	tt
yheU	CDS	conserved protein	Cytoplasmic	COG3089;Uncharacterized protein conserved in bacteria		gg	o
yheV	CDS	predicted protein	Cytoplasmic			gg	o
yhfA	CDS	conserved protein	Cytoplasmic	COG1765;Predicted redox protein, regulator of disulfide bond formation		g	o
yhfG	CDS	predicted protein	Cytoplasmic			gg	o
yhfK	CDS	conserved inner membrane protein	Integral Membrane Protein	COG1289;Predicted membrane protein	GO:0008152 metabolism		
yhfL	CDS	conserved secreted peptide	Outer Membrane Lipoprotein			o	tt
yhfS	CDS	conserved protein	Cytoplasmic			gg	t
yhfT	CDS	predicted inner membrane protein	Integral Membrane Protein			o	tt
yhfU	CDS	predicted protein	Membrane Anchored				
yhfW	CDS	predicted mutase	Cytoplasmic	COG1015;Phosphopentomutase		gg	t
yhfX	CDS	predicted amino acid racemase	Cytoplasmic	COG3457;Predicted amino acid racemase		o	tt
yhfY	CDS	conserved protein	Cytoplasmic				
yhfZ	CDS	conserved protein	Cytoplasmic				
yhgA	CDS	predicted transposase	Cytoplasmic			gg	t
yhgE	CDS	predicted inner membrane protein	Integral Membrane Protein			o	tt
yhgF	CDS	predicted transcriptional accessory protein	Cytoplasmic			g	tt
yhgG	CDS	predicted DNA-binding transcriptional regulator	Cytoplasmic		GO:0006355 regulation of transcription, DNA-dependent	gg	tt
yhgN	CDS	predicted antibiotic transporter	Integral Membrane Protein	COG2095;Multiple antibiotic transporter		o	tt
yhhA	CDS	conserved protein	Periplasmic			o	tt
yhhF	CDS	predicted methyltransferase	Cytoplasmic	COG0742;N6-adenine-specific methylase		gg	t
yhhH	CDS	predicted protein	Membrane Anchored			o	o
yhhI	CDS	predicted transposase	Cytoplasmic			gg	t
yhhJ	CDS	predicted transporter subunit -!- membrane component of ABC superfamily	Integral Membrane Protein				
yhhK	CDS	conserved protein	Cytoplasmic			gg	o
yhhL	CDS	conserved inner membrane protein	Integral Membrane Protein	COG3776;Predicted membrane protein			
yhhM	CDS	conserved protein	Membrane Anchored			o	tt
yhhN	CDS	conserved inner membrane protein	Integral Membrane Protein	COG3714;Predicted membrane protein		o	tt
yhhP	CDS	conserved protein required for cell growth	Cytoplasmic	COG0425;Predicted redox protein, regulator of disulfide bond formation		gg	t
yhhQ	CDS	conserved inner membrane protein	Integral Membrane Protein	COG1738;Uncharacterized conserved protein		o	tt
yhhS	CDS	predicted transporter	Integral Membrane Protein	COG0477;Permeases of the major facilitator superfamily			
yhhT	CDS	predicted inner membrane protein	Integral Membrane Protein				
yhhW	CDS	predicted protein	Cytoplasmic	COG1741;Pirin-related protein		g	t
yhhX	CDS	predicted oxidoreductase with NAD(P)-binding Rossmann-fold domain	Cytoplasmic	COG0673;Predicted dehydrogenases and related proteins		gg	t
yhhY	CDS	predicted acetyltransferase	Cytoplasmic	COG0454;Histone acetyltransferase HPA2 and related acetyltransferases		g	t
yhhZ	CDS	conserved protein	Cytoplasmic	COG3157;Hemolysin-coregulated protein (uncharacterized)		g	o
yhiD	CDS	predicted Mg(2+) transport ATPase inner membrane protein	Integral Membrane Protein	COG1285;Uncharacterized membrane protein			
yhiF	CDS	predicted DNA-binding ranscriptional regulator	Cytoplasmic	COG2771;DNA-binding HTH domain-containing proteins	GO:0006355 regulation of transcription, DNA-dependent	gg	t
yhiI	CDS	predicted HlyD family secretion protein	Membrane Anchored	COG0845;Membrane-fusion protein		o	tt
yhiJ	CDS	predicted protein	Cytoplasmic			gg	o
yhiK	CDS	predicted protein (pseudogene)	Cytoplasmic				
yhiL	CDS	predicted protein	Cytoplasmic			o	o
yhiM	CDS	conserved inner membrane protein	Integral Membrane Protein				
yhiN	CDS	predicted oxidoreductase, FAD/NAD(P)-binding domain	Periplasmic	COG2081;Predicted flavoproteins		g	t
yhiO	CDS	predicted universal stress (ethanol tolerance) protein B	Integral Membrane Protein		GO:0006950 response to stress -!- GO:0006805 xenobiotic metabolism	o	tt
yhiP	CDS	predicted transporter	Integral Membrane Protein	COG3104;Dipeptide/tripeptide permease		o	tt
yhiQ	CDS	predicted SAM-dependent methyltransferase	Cytoplasmic	COG0500;SAM-dependent methyltransferases			
yhiR	CDS	predicted DNA (exogenous) processing protein	Cytoplasmic	COG2961;Protein involved in catabolism of external DNA		gg	o
yhiS	CDS	predicted protein	Cytoplasmic			o	o
yhjA	CDS	predicted cytochrome C peroxidase	Membrane Anchored	COG1858;Cytochrome c peroxidase	GO:0017004 cytochrome biogenesis	o	tt
yhjB	CDS	predicted DNA-binding response regulator in two-component regulatory system	Cytoplasmic	COG2197;Response regulator containing a CheY-like receiver domain and an HTH DNA-binding domain	GO:0006350 transcription	gg	t
yhjC	CDS	predicted DNA-binding transcriptional regulator	Cytoplasmic	COG0583;Transcriptional regulator	GO:0006350 transcription	o	t
yhjD	CDS	conserved inner membrane protein	Integral Membrane Protein	COG1295;Predicted membrane protein		o	tt
yhjE	CDS	predicted transporter	Integral Membrane Protein	COG0477;Permeases of the major facilitator superfamily		o	tt
yhjG	CDS	predicted outer membrane biogenesis protein	Membrane Anchored	COG2982;Uncharacterized protein involved in outer membrane biogenesis		o	tt
yhjH	CDS	EAL domain containing protein involved in flagellar function	Cytoplasmic	COG2200;FOG: EAL domain		g	o
yhjJ	CDS	predicted zinc-dependent peptidase	Periplasmic	COG0612;Predicted Zn-dependent peptidases		o	tt
yhjK	CDS	predicted diguanylate cyclase	Integral Membrane Protein	COG3706;Response regulator containing a CheY-like receiver domain and a GGDEF domain -!- COG2200;FOG: EAL domain			
yhjQ	CDS	cell division protein (chromosome partitioning ATPase) (pseudogene)	Cytoplasmic	COG1192;ATPases involved in chromosome partitioning			
yhjR	CDS	conserved protein	Cytoplasmic			gg	t
yhjV	CDS	predicted transporter	Integral Membrane Protein	COG0814;Amino acid permeases		o	tt
yhjX	CDS	predicted transporter	Integral Membrane Protein	COG0477;Permeases of the major facilitator superfamily	GO:0042493 response to drug	o	tt
yhjY	CDS	conserved protein	Periplasmic		GO:0019395 fatty acid oxidation		
yiaA	CDS	conserved inner membrane protein	Integral Membrane Protein			o	tt
yiaB	CDS	conserved inner membrane protein	Integral Membrane Protein	COG4682;Predicted membrane protein			
yiaC	CDS	predicted acyltransferase with acyl-CoA N-acyltransferase domain	Cytoplasmic	COG0454;Histone acetyltransferase HPA2 and related acetyltransferases		gg	t
yiaD	CDS	predicted outer membrane lipoprotien	Outer Membrane Lipoprotein				
yiaF	CDS	conserved protein	Membrane Lipoprotein				
yiaG	CDS	predicted transcriptional regulator	Cytoplasmic	COG2944;Predicted transcriptional regulator	GO:0006355 regulation of transcription, DNA-dependent	gg	o

yiaH	CDS	conserved inner membrane protein	Integral Membrane Protein	COG3274;Uncharacterized protein conserved in bacteria		o	tt
yiaI	CDS	predicted hydrogenase, 4Fe-4S ferredoxin-type component	Cytoplasmic	COG1142;Fe-S-cluster-containing hydrogenase components 2		gg	t
yiaJ	CDS	predicted DNA-binding transcriptional repressor	Cytoplasmic	COG1414;Transcriptional regulator	GO:0006350 transcription	gg	tt
yiaK	CDS	2,3-diketo-L-gulonate dehydrogenase, NADH-dependent	Cytoplasmic	COG2055;Malate/L-lactate dehydrogenases		g	t
yiaL	CDS	conserved protein	Cytoplasmic	COG2731;Beta-galactosidase, beta subunit		o	t
yiaM	CDS	predicted transporter	Integral Membrane Protein	COG3090;TRAP-type C4-dicarboxylate transport system, small permease component		o	tt
yiaN	CDS	predicted transporter	Integral Membrane Protein				
yiaO	CDS	predicted transporter	Periplasmic	COG1638;TRAP-type C4-dicarboxylate transport system, periplasmic component		o	tt
yiaT	CDS	predicted protein	Outer Membrane B-barrel protein	COG3713;Outer membrane protein V		o	tt
yiaU	CDS	predicted DNA-binding transcriptional regulator	Cytoplasmic	COG0583;Transcriptional regulator	GO:0006350 transcription	gg	t
yiaV	CDS	membrane fusion protein (MFP) component of efflux pump, signal anchor	Integral Membrane Protein	COG1566;Multidrug resistance efflux pump		o	tt
yiaW	CDS	conserved inner membrane protein	Integral Membrane Protein			o	tt
yiaY	CDS	predicted Fe-containing alcohol dehydrogenase	Cytoplasmic				
yibA	CDS	lyase containing HEAT-repeat	Cytoplasmic	COG1413;FOG: HEAT repeat		o	tt
yibD	CDS	predicted glycosyl transferase	Cytoplasmic	COG0463;Glycosyltransferases involved in cell wall biogenesis		o	tt
yibF	CDS	predicted glutathione S-transferase	Cytoplasmic	COG0625;Glutathione S-transferase		gg	o
yibG	CDS	conserved protein	Periplasmic			o	tt
yibH	CDS	predicted protein	Membrane Anchored	COG1566;Multidrug resistance efflux pump		o	tt
yibI	CDS	predicted inner membrane protein	Integral Membrane Protein			o	tt
yibJ	CDS	predicted Rhs-family protein	Cytoplasmic				
yibK	CDS	predicted rRNA methylase	Cytoplasmic	COG0219;Predicted rRNA methylase (SpoU class)	GO:0009451 RNA modification	g	o
yibL	CDS	conserved protein	Cytoplasmic			gg	o
yibN	CDS	predicted rhodanese-related sulfurtransferase	Membrane Anchored	COG0607;Rhodanese-related sulfurtransferase		o	tt
yibQ	CDS	predicted polysaccharide deacetylase	Periplasmic				
yibT	CDS	predicted protein	Cytoplasmic			gg	o
yicC	CDS	conserved protein	Cytoplasmic	COG1561;Uncharacterized stress-induced protein		gg	tt
yicE	CDS	predicted transporter	Integral Membrane Protein	COG2233;Xanthine/uracil permeases		o	tt
yicG	CDS	conserved inner membrane protein	Integral Membrane Protein			o	tt
yicH	CDS	conserved protein	Membrane Anchored			o	tt
yicI	CDS	predicted alpha-glucosidase	Cytoplasmic	COG1501;Alpha-glucosidases, family 31 of glycosyl hydrolases		o	tt
yicJ	CDS	predicted transporter	Integral Membrane Protein				
yicL	CDS	predicted inner membrane protein	Integral Membrane Protein	COG0697;Permeases of the drug/metabolite transporter (DMT) superfamily		o	tt
yicM	CDS	predicted transporter	Integral Membrane Protein	COG2814;Arabinose efflux permease			
yicN	CDS	conserved protein	Membrane Anchored				
yicO	CDS	predicted xanthine/uracil permase	Integral Membrane Protein	COG2252;Permeases			
yicR	CDS	protein associated with replication fork, possible DNA repair protein	Cytoplasmic	COG2003;DNA repair proteins	GO:0006261 DNA dependent DNA replication -!- GO:0006281 DNA		
yicS	CDS	predicted protein	Periplasmic				
yidA	CDS	predicted hydrolase	Cytoplasmic	COG0561;Predicted hydrolases of the HAD superfamily		o	tt
yidB	CDS	conserved protein	Cytoplasmic			gg	t
yidC	CDS	cytoplasmic insertase into membrane protein, Sec system	Integral Membrane Protein	COG0706;Preprotein translocase subunit YidC		o	tt
yidD	CDS	predicted protein	Cytoplasmic			g	tt
yidE	CDS	predicted transporter	Integral Membrane Protein			o	tt
yidF	CDS	predicted DNA-binding transcriptional regulator	Cytoplasmic	COG0641;Arylsulfatase regulator (Fe-S oxidoreductase)		gg	o
yidG	CDS	predicted inner membrane protein	Integral Membrane Protein			o	tt
yidH	CDS	conserved inner membrane protein	Integral Membrane Protein	COG2149;Predicted membrane protein		o	tt
yidI	CDS	predicted inner membrane protein	Integral Membrane Protein			o	tt
yidJ	CDS	predicted sulfatase/phosphatase	Cytoplasmic	COG3119;Arylsulfatase A and related enzymes	GO:0006790 sulfur metabolism	gg	t
yidK	CDS	predicted transporter	Integral Membrane Protein	COG4146;Predicted symporter		o	tt
yidL	CDS	predicted DNA-binding transcriptional regulator	Cytoplasmic	COG2207;AraC-type DNA-binding domain-containing proteins	GO:0006350 transcription	gg	t
yidP	CDS	predicted DNA-binding transcriptional regulator	Cytoplasmic	COG2188;Transcriptional regulators	GO:0006350 transcription	g	t
yidQ	CDS	conserved outer membrane protein	Outer Membrane Lipoprotein				
yidR	CDS	conserved protein	Cytoplasmic				
yidX	CDS	predicted lipoproteinC	Outer Membrane Lipoprotein				
yidZ	CDS	predicted DNA-binding transcriptional regulator	Cytoplasmic	COG0583;Transcriptional regulator	GO:0006350 transcription	g	t
yieE	CDS	predicted phosphopantetheinyl transferase	Cytoplasmic	COG2091;Phosphopantetheinyl transferase		gg	o
yieF	CDS	chromate reductase, Class I, flavoprotein	Cytoplasmic	COG0431;Predicted flavoprotein		gg	t
yieG	CDS	predicted inner membrane protein	Integral Membrane Protein	COG2252;Permeases		o	tt
yieH	CDS	predicted hydrolase	Cytoplasmic	COG0637;Predicted phosphatase/phosphohexomutase		gg	tt
yieI	CDS	predicted inner membrane protein	Integral Membrane Protein			o	tt
yieK	CDS	predicted 6-phosphogluconolactonase	Cytoplasmic				
yieL	CDS	predicted xylanase	Periplasmic	COG2382;Enterochelin esterase and related enzymes	GO:0016052 carbohydrate		
yieM	CDS	predicted von Willibrand factor containing protein	Cytoplasmic				
yieN	CDS	fused predicted transcriptional regulators	Cytoplasmic			g	t
yieP	CDS	predicted transcriptional regulator	Cytoplasmic				
yifB	CDS	predicted bifunctional protein, enzyme and transcriptional regulator	Periplasmic	COG0606;Predicted ATPase with chaperone activity		o	t
yifE	CDS	conserved protein	Cytoplasmic	COG3085;Uncharacterized protein conserved in bacteria	GO:0006355 regulation of transcription, DNA-dependent	gg	t
yifK	CDS	predicted transporter	Integral Membrane Protein				
yifL	CDS	predicted lipoprotein	Membrane Lipoprotein			o	tt
yifN	CDS	conserved protein (pseudogene)	Cytoplasmic			gg	o
yifO	CDS	conserved protein	Cytoplasmic				
yigA	CDS	conserved protein	Cytoplasmic	COG3159;Uncharacterized protein conserved in bacteria		gg	t
yigB	CDS	predicted hydrolase	Cytoplasmic	COG1011;Predicted hydrolase (HAD superfamily)		gg	o
yigE	CDS	predicted protein	Periplasmic				
yigF	CDS	conserved inner membrane protein	Integral Membrane Protein			o	tt
yigG	CDS	predicted inner membrane protein	Integral Membrane Protein				
yigI	CDS	conserved protein	Cytoplasmic				
yigL	CDS	predicted hydrolase	Cytoplasmic				
yigM	CDS	predicted inner membrane protein	Integral Membrane Protein	COG0697;Permeases of the drug/metabolite transporter (DMT) superfamily		o	tt
yigP	CDS	conserved protein	Cytoplasmic	COG3165;Uncharacterized protein conserved in bacteria		gg	t
yigZ	CDS	predicted elongation factor	Cytoplasmic		GO:0006412 protein biosynthesis		
yihA	CDS	GTP-binding protein	Cytoplasmic	COG0218;Predicted GTPase	GO:0007049 cell cycle		
yihD	CDS	conserved protein	Cytoplasmic	COG3084;Uncharacterized protein conserved in bacteria		gg	o
yihE	CDS	predicted kinase	Cytoplasmic	COG2334;Putative homoserine kinase type II (protein kinase fold)		g	tt
yihF	CDS	conserved protein	Membrane Anchored				
yihG	CDS	predicted endonuclease	Cytoplasmic	COG0204;1-acyl-sn-glycerol-3-phosphate acyltransferase	GO:0009451 RNA modification -!- GO:0006401 RNA catabolism	o	tt
yihI	CDS	conserved protein	Cytoplasmic	COG3078;Uncharacterized protein conserved in bacteria		gg	t
yihL	CDS	predicted DNA-binding transcriptional regulator	Cytoplasmic	COG2188;Transcriptional regulators	GO:0006350 transcription	o	tt
yihM	CDS	predicted sugar phosphate isomerase	Cytoplasmic			gg	t
yihN	CDS	predicted transporter	Membrane Lipoprotein	COG0477;Permeases of the major facilitator superfamily		o	tt
yihO	CDS	predited transporter	Integral Membrane Protein				
yihP	CDS	predicted transporter	Integral Membrane Protein			o	tt
yihQ	CDS	alpha-glucosidase	Cytoplasmic	COG1501;Alpha-glucosidases, family 31 of glycosyl hydrolases		gg	t
yihR	CDS	predicted aldose-1-epimerase	Cytoplasmic			o	t
yihS	CDS	predicted glucosamine isomerase	Cytoplasmic				
yihT	CDS	predicted aldolase	Cytoplasmic	COG3684;Tagatose-1,6-bisphosphate aldolase		gg	t
yihU	CDS	predicted oxidoreductase with NAD(P)-binding Rossmann-fold domain	Cytoplasmic	COG2084;3-hydroxyisobutyrate dehydrogenase and related beta-hydroxyacid dehydrogenases		gg	t
yihV	CDS	predicted sugar kinase	Cytoplasmic	COG0524;Sugar kinases, ribokinase family			
yihW	CDS	predicted DNA-binding transcriptional regulator	Cytoplasmic	COG1349;Transcriptional regulators of sugar metabolism	GO:0006350 transcription		
yihX	CDS	predicted hydrolase	Cytoplasmic				
yiiD	CDS	predicted acetyltransferase	Cytoplasmic	COG0454;Histone acetyltransferase HPA2 and related acetyltransferases		o	tt
yiiE	CDS	predicted transcriptional regulator	Cytoplasmic				
yiiF	CDS	conserved protein	Cytoplasmic				
yiiG	CDS	conserved protein	Inner Membrane Lipoprotein			o	tt
yiiL	CDS	L-rhamnose mutarotase	Cytoplasmic	COG3254;Uncharacterized conserved protein		gg	t
yiiM	CDS	conserved protein	Cytoplasmic	COG2258;Uncharacterized protein conserved in bacteria			
yiiQ	CDS	conserved protein	Periplasmic			o	tt
yiiR	CDS	conserved inner membrane protein	Integral Membrane Protein	COG3152;Predicted membrane protein		o	tt
yiiS	CDS	conserved protein	Cytoplasmic	COG3691;Uncharacterized protein conserved in bacteria		gg	o
yiiT	CDS	stress-induced protein	Cytoplasmic	COG0589;Universal stress protein UspA and related nucleotide-binding proteins	GO:0006950 response to stress	gg	t
yiiU	CDS	conserved protein	Cytoplasmic	COG3074;Uncharacterized protein conserved in bacteria		gg	tt
yiiX	CDS	predicted peptidoglycan peptidase	Periplasmic			o	tt
yijD	CDS	conserved inner membrane protein	Integral Membrane Protein			o	tt
yijE	CDS	predicted permease	Integral Membrane Protein				
yijF	CDS	conserved protein	Periplasmic	COG3738;Uncharacterized protein conserved in bacteria		o	tt
yijO	CDS	predicted DNA-binding transcriptional regulator	Cytoplasmic	COG2207;AraC-type DNA-binding domain-containing proteins	GO:0006350 transcription	o	tt
yijP	CDS	conserved inner membrane protein	Integral Membrane Protein	COG2194;Predicted membrane-associated, metal-dependent hydrolase		o	tt
yjaA	CDS	conserved protein	Cytoplasmic			gg	t
yjaB	CDS	predicted acetyltransferase	Cytoplasmic	COG0454;Histone acetyltransferase HPA2 and related acetyltransferases		gg	o
yjaG	CDS	conserved protein	Cytoplasmic	COG3068;Uncharacterized protein conserved in bacteria		gg	tt
yjaH	CDS	conserved protein	Outer Membrane Lipoprotein			o	tt
yjbA	CDS	predicted phosphate starvation inducible protein	Integral Membrane Protein	COG3223;Predicted membrane protein		o	tt
yjbB	CDS	predicted transporter	Integral Membrane Protein	COG1283;Na+/phosphate symporter		o	tt

yjbC	CDS	23S rRNA pseudouridine synthase	Cytoplasmic	COG1187;16S rRNA uridine-516 pseudouridylate synthase and related pseudouridylate synthases		gg	t
yjbD	CDS	conserved protein	Cytoplasmic			gg	t
yjbE	CDS	predicted protein	Periplasmic			o	tt
yjbF	CDS	predicted lipoprotein	Outer Membrane Lipoprotein				
yjbG	CDS	conserved protein	Periplasmic			o	tt
yjbH	CDS	predicted porin	Membrane Lipoprotein			o	tt
yjbI	CDS	conserved protein	Cytoplasmic	COG1357;Uncharacterized low-complexity proteins		g	o
yjbJ	CDS	predicted stress response protein	Cytoplasmic	COG3237;Uncharacterized protein conserved in bacteria		gg	t
yjbL	CDS	predicted protein	Cytoplasmic			gg	o
yjbM	CDS	predicted protein	Cytoplasmic			o	tt
yjbN	CDS	tRNA-dihydrouridine synthase A	Cytoplasmic	COG0042;tRNA-dihydrouridine synthase			
yjbO	CDS	phage shock protein G	Integral Membrane Protein				
yjbQ	CDS	conserved protein	Cytoplasmic	COG0432;Uncharacterized conserved protein		gg	o
yjbR	CDS	conserved protein	Cytoplasmic	COG2315;Uncharacterized protein conserved in bacteria		gg	o
yjcB	CDS	predicted inner membrane protein	Integral Membrane Protein				
yjcC	CDS	predicted signal transduction protein (EAL domain containing protein)	Integral Membrane Protein	COG4943;Predicted signal transduction protein containing sensor and EAL domains		o	tt
yjcD	CDS	predicted permease	Integral Membrane Protein	COG2252;Permeases		o	tt
yjcE	CDS	predicted cation/proton antiporter	Integral Membrane Protein	COG0025;NhaP-type Na+/H+ and K+/H+ antiporters		o	tt
yjcF	CDS	conserved protein	Cytoplasmic	COG1357;Uncharacterized low-complexity proteins		o	o
yjcH	CDS	conserved inner membrane protein involved in acetate transport	Integral Membrane Protein	COG3162;Predicted membrane protein		o	tt
yjcO	CDS	conserved protein	Periplasmic	COG0790;FOG: TPR repeat, SEL1 subfamily		o	tt
yjcP	CDS	predicted outer membrane factor of efflux pump	Outer Membrane Lipoprotein	COG1538;Outer membrane protein		o	tt
yjcQ	CDS	predicted multidrug efflux system component	Integral Membrane Protein	COG1289;Predicted membrane protein		o	tt
yjcR	CDS	predicted membrane fusion protein of efflux pump	Membrane Anchored	COG1566;Multidrug resistance efflux pump		o	tt
yjcS	CDS	predicted alkyl sulfatase	Periplasmic	COG2015;Alkyl sulfatase and related hydrolases	GO:0006790 sulfur metabolism		
yjcZ	CDS	conserved protein	Cytoplasmic				
yjdA	CDS	conserved protein with nucleoside triphosphate hydrolase domain	Cytoplasmic	COG0699;Predicted GTPases (dynamin-related)		g	t
yjdC	CDS	predicted transcriptional regulator	Cytoplasmic	COG1309;Transcriptional regulator	GO:0006355 regulation of transcription, DNA-dependent		
yjdF	CDS	conserved inner membrane protein	Integral Membrane Protein	COG3647;Predicted membrane protein		o	tt
yjdI	CDS	conserved protein	Cytoplasmic	COG3592;Uncharacterized conserved protein		gg	o
yjdJ	CDS	predicted acyltransferase with acyl-CoA N-acyltransferase domain	Cytoplasmic	COG2388;Predicted acetyltransferase		gg	o
yjdK	CDS	predicted protein	Cytoplasmic			gg	o
yjdL	CDS	predicted transporter	Integral Membrane Protein	COG3104;Dipeptide/tripeptide permease		o	tt
yjdO	CDS	predicted protein	Integral Membrane Protein				
yjdP	CDS	conserved protein	Periplasmic				
yjeB	CDS	predicted DNA-binding transcriptional regulator	Cytoplasmic	COG1959;Predicted transcriptional regulator		o	tt
yjeE	CDS	ATPase with strong ADP affinity	Cytoplasmic	COG0802;Predicted ATPase or kinase		gg	o
yjeF	CDS	predicted carbohydrate kinase	Cytoplasmic	COG0062;Uncharacterized conserved protein -!- COG0063;Predicted sugar kinase		gg	o
yjeH	CDS	predicted transporter	Integral Membrane Protein	COG0531;Amino acid transporters		o	tt
yjeI	CDS	conserved protein	Outer Membrane Lipoprotein				
yjeJ	CDS	predicted protein	Cytoplasmic			o	t
yjeK	CDS	predicted lysine aminomutase	Cytoplasmic	COG1509;Lysine 2,3-aminomutase		gg	t
yjeM	CDS	predicted transporter	Integral Membrane Protein	COG0531;Amino acid transporters			
yjeN	CDS	predicted protein	Cytoplasmic			gg	o
yjeO	CDS	conserved inner membrane protein	Integral Membrane Protein			o	tt
yjeP	CDS	predicted mechanosensitive channel	Integral Membrane Protein	COG3264;Small-conductance mechanosensitive channel		o	tt
yjeS	CDS	predicted Fe-S electron transport protein	Cytoplasmic	COG1600;Uncharacterized Fe-S protein		gg	t
yjeT	CDS	conserved inner membrane protein	Integral Membrane Protein	COG3242;Uncharacterized protein conserved in bacteria		o	tt
yjfC	CDS	predicted synthetase/amidase	Cytoplasmic	COG0754;Glutathionylspermidine synthase		g	t
yjfF	CDS	predicted sugar transporter subunit -!- membrane component of ABC superfamily	Integral Membrane Protein	COG1172;Ribose/xylose/arabinose/galactoside ABC-type transport systems, permease components			
yjfI	CDS	conserved protein	Cytoplasmic	COG3789;Uncharacterized protein conserved in bacteria		g	t
yjfJ	CDS	predicted transcriptional regulator effector protein	Cytoplasmic	COG1842;Phage shock protein A (IM30), suppresses sigma54-dependent transcription		gg	o
yjfK	CDS	conserved protein	Cytoplasmic			gg	t
yjfL	CDS	conserved inner membrane protein	Integral Membrane Protein	COG3766;Predicted membrane protein		o	tt
yjfM	CDS	conserved protein	Cytoplasmic			o	tt
yjfN	CDS	predicted protein	Periplasmic				
yjfO	CDS	conserved protein	Outer Membrane Lipoprotein				
yjfP	CDS	predicted hydrolase	Cytoplasmic	COG1073;Hydrolases of the alpha/beta superfamily		gg	o
yjfY	CDS	predicted protein	Periplasmic			gg	o
yjfZ	CDS	predicted protein	Cytoplasmic			g	o
yjgA	CDS	conserved protein	Cytoplasmic	COG3028;Uncharacterized protein conserved in bacteria		gg	t
yjgB	CDS	predicted alcohol dehydrogenase, Zn-dependent and NAD(P)-binding	Cytoplasmic				
yjgD	CDS	conserved protein	Cytoplasmic	COG3076;Uncharacterized protein conserved in bacteria		gg	tt
yjgF	CDS	ketoacid-binding protein	Cytoplasmic	COG0251;Putative translation initiation inhibitor, yjgF family			
yjgH	CDS	predicted mRNA endoribonuclease	Cytoplasmic	COG0251;Putative translation initiation inhibitor, yjgF family	GO:0006412 protein biosynthesis	g	o
yjgI	CDS	predicted oxidoreductase with NAD(P)-binding Rossmann-fold domain	Cytoplasmic	COG1028;Dehydrogenases with different specificities (related to short-chain alcohol dehydrogenases)		gg	o
yjgJ	CDS	predicted transcriptional regulator	Cytoplasmic			o	tt
yjgK	CDS	conserved protein	Cytoplasmic				
yjgL	CDS	predicted protein	Cytoplasmic				
yjgM	CDS	predicted acetyltransferase	Cytoplasmic				
yjgN	CDS	conserved inner membrane protein	Integral Membrane Protein				
yjgP	CDS	conserved inner membrane protein	Integral Membrane Protein	COG0795;Predicted permeases		o	tt
yjgQ	CDS	conserved inner membrane protein	Integral Membrane Protein				
yjgR	CDS	predicted ATPase	Cytoplasmic	COG0433;Predicted ATPase		gg	t
yjgW	CDS	KpLE2 phage-like element; predicted protein	Cytoplasmic			o	t
yjgX	CDS	KpLE2 phage-like element; predicted protein, C-ter fragment (pseudogene)	Cytoplasmic				
yjgX	ancestral	KpLE2 phage-like element; predicted protein (pseudogene)	Membrane Anchored				
yjgX	CDS	KpLE2 phage-like element; predicted protein, middle fragment (pseudogene)	Cytoplasmic	COG2194;Predicted membrane-associated, metal-dependent hydrolase		gg	o
yjgX	CDS	KpLE2 phage-like element; predicted protein, N-ter fragment (pseudogene)	Membrane Anchored				
yjgZ	CDS	KpLE2 phage-like element; predicted protein	Cytoplasmic			g	o
yjhA	CDS	N-acetylneuraminic acid outer membrane channel protein	Periplasmic				
yjhB	CDS	KpLE2 phage-like element; predicted transporter	Integral Membrane Protein				
yjhC	CDS	KpLE2 phage-like element; predicted oxidoreductase	Cytoplasmic	COG0673;Predicted dehydrogenases and related proteins			
yjhD	CDS	KpLE2 phage-like element; predicted protein (pseudogene)	Cytoplasmic				
yjhE	CDS	KpLE2 phage-like element; predicted membrane protein (pseudogene)	Integral Membrane Protein			o	tt
yjhF	CDS	KpLE2 phage-like element; predicted transporter	Integral Membrane Protein	COG2610;H+/gluconate symporter and related permeases		o	tt
yjhG	CDS	KpLE2 phage-like element; predicted dehydratase	Cytoplasmic	COG0129;Dihydroxyacid dehydratase/phosphogluconate dehydratase		o	tt
yjhH	CDS	KpLE2 phage-like element; predicted lyase/synthase	Cytoplasmic				
yjhI	CDS	KpLE2 phage-like element; predicted DNA-binding transcriptional regulator	Cytoplasmic	COG1414;Transcriptional regulator	GO:0006350 transcription	gg	o
yjhP	CDS	KpLE2 phage-like element; predicted methyltransferase	Cytoplasmic	COG0500;SAM-dependent methyltransferases		o	t
yjhQ	CDS	KpLE2 phage-like element; predicted	Cytoplasmic	COG3153;Predicted acetyltransferase	GO:0016070 RNA metabolism	o	o
yjhR	CDS	KpLE2 phage-like element; predicted frameshift suppressor	Cytoplasmic	COG1112;Superfamily I DNA and RNA helicases and helicase subunits		gg	t
yjhS	CDS	conserved protein	Cytoplasmic	COG2801;Transposase and inactivated derivatives		o	o
yjhT	CDS	conserved protein	Periplasmic				
yjhU	CDS	KpLE2 phage-like element; predicted DNA-binding transcriptional regulator	Cytoplasmic	COG2390;Transcriptional regulator, contains sigma factor-related N-terminal domain	GO:0006355 regulation of transcription, DNA-dependent		
yjhV	CDS	KpLE2 phage-like element; predicted protein	Cytoplasmic			gg	o
yjhW	CDS	KpLE2 phage-like element; predicted transposase fragment (pseudogene)	Cytoplasmic				
yjhX	CDS	conserved protein	Cytoplasmic				
yjiA	CDS	predicted GTPase	Cytoplasmic		GO:0009236 vitamin B12		
yjiC	CDS	predicted protein	Cytoplasmic			g	o
yjiD	CDS	DNA replication/recombination/repair protein	Cytoplasmic				
yjiE	CDS	predicted DNA-binding transcriptional regulator	Cytoplasmic	COG0583;Transcriptional regulator	GO:0006350 transcription	g	o
yjiG	CDS	conserved inner membrane protein	Integral Membrane Protein	COG0700;Uncharacterized membrane protein		o	tt
yjiH	CDS	conserved inner membrane protein	Integral Membrane Protein	COG3314;Uncharacterized protein conserved in bacteria			
yjiJ	CDS	predicted inner membrane protein	Integral Membrane Protein	COG0477;Permeases of the major facilitator superfamily		o	tt
yjiK	CDS	conserved protein	Membrane Anchored	COG3204;Uncharacterized protein conserved in bacteria			
yjiL	CDS	predicted ATPase, activator of (R)-hydroxyglutaryl-CoA dehydratase	Cytoplasmic	COG1924;Activator of 2-hydroxyglutaryl-CoA dehydratase (HSP70-class ATPase domain)			
yjiM	CDS	predicted 2-hydroxyglutaryl-CoA dehydratase	Cytoplasmic				
yjiN	CDS	conserved inner membrane protein	Integral Membrane Protein	COG2733;Predicted membrane protein		o	tt
yjiO	CDS	multidrug efflux system protein	Integral Membrane Protein	COG0477;Permeases of the major facilitator superfamily		o	tt
yjiP	CDS	predicted transposase (pseudogene)	Cytoplasmic				
yjiQ	CDS	predicted transposase	Cytoplasmic			gg	o

yjiR	CDS	fused predicted DNA-binding transcriptional regulator -!- predicted aminotransferase	Cytoplasmic	COG1167;Transcriptional regulators containing a DNA-binding HTH domain and an aminotransferase domain (MocR family) and their eukaryotic orthologs	GO:0006350 transcription	o	t
yjiS	CDS	conserved protein	Cytoplasmic			gg	tt
yjiT	CDS	conserved protein	Cytoplasmic				
yjiV	CDS	conserved protein (pseudogene)	Cytoplasmic				
yjiW	CDS	conserved protein	Cytoplasmic		GO:0009432 SOS response	o	tt
yjiX	CDS	conserved protein	Cytoplasmic	COG2879;Uncharacterized small protein		gg	o
yjiY	CDS	predicted inner membrane protein	Integral Membrane Protein	COG1966;Carbon starvation protein, predicted membrane protein	GO:0042594 response to starvation		
yjiZ	CDS	predicted transporter	Integral Membrane Protein	COG0477;Permeases of the major facilitator superfamily		o	tt
yjjA	CDS	conserved protein	Periplasmic				
yjjB	CDS	conserved inner membrane protein	Integral Membrane Protein	COG3610;Uncharacterized conserved protein		o	tt
yjjG	CDS	predicted hydrolase	Cytoplasmic	COG1011;Predicted hydrolase (HAD superfamily)		o	tt
yjjI	CDS	conserved protein	Cytoplasmic			gg	t
yjjJ	CDS	predicted DNA-binding transcriptional regulator	Cytoplasmic			o	tt
yjjK	CDS	fused predicted transporter subunits -!- ATP-binding components of ABC superfamily	Cytoplasmic	COG0488;ATPase components of ABC transporters with duplicated ATPase domains		gg	t
yjjM	CDS	predicted DNA-binding transcriptional regulator	Cytoplasmic		GO:0006355 regulation of transcription, DNA-dependent		
yjjN	CDS	predicted oxidoreductase, Zn-dependent and NAD(P)-binding	Cytoplasmic	COG1063;Threonine dehydrogenase and related Zn-dependent dehydrogenases			
yjjP	CDS	predicted inner membrane protein	Integral Membrane Protein				
yjjQ	CDS	predicted DNA-binding transcriptional regulator	Cytoplasmic	COG2197;Response regulator containing a CheY-like receiver domain and an HTH DNA-binding domain		o	tt
yjjU	CDS	predicted esterase	Cytoplasmic	COG4667;Predicted esterase of the alpha-beta hydrolase superfamily	GO:0006355 regulation of transcription, DNA-dependent	gg	tt
yjjV	CDS	predicted DNase	Cytoplasmic			g	o
yjjW	CDS	predicted pyruvate formate lyase activating enzyme	Cytoplasmic	COG1180;Pyruvate-formate lyase-activating enzyme	GO:0009061 anaerobic respiration -!- GO:0006464 protein modification	g	t
yjjX	CDS	thiamin metabolism associated protein	Cytoplasmic	COG1986;Uncharacterized conserved protein			
yjjY	CDS	predicted protein	Cytoplasmic			o	t
yjjZ	CDS	predicted protein	Integral Membrane Protein				
yjtD	CDS	predicted rRNA methyltransferase	Cytoplasmic	COG0565;rRNA methylase	GO:0009451 RNA modification	g	t
ykfA	CDS	CP4-6 prophage; predicted GTP-binding protein	Cytoplasmic			o	t
ykfB	CDS	CP4-6 prophage; predicted protein	Periplasmic			o	tt
ykfC	CDS	CP4-6 prophage; conserved protein	Cytoplasmic	COG3344;Retron-type reverse transcriptase			
ykfF	CDS	CP4-6 prophage; predicted protein	Cytoplasmic				
ykfG	CDS	CP4-6 prophage; predicted DNA repair protein	Cytoplasmic	COG2003;DNA repair proteins		gg	o
ykfH	CDS	predicted protein	Cytoplasmic				
ykfI	CDS	CP4-6 prophage; toxin of the YkfI-YafW toxin-antitoxin system	Cytoplasmic			gg	tt
ykfJ	CDS	conserved protein	Cytoplasmic	COG1690;Uncharacterized conserved protein			
ykgA	CDS	predicted DNA-binding transcriptional regulator	Cytoplasmic	COG2207;AraC-type DNA-binding domain-containing proteins	GO:0006350 transcription		
ykgB	CDS	conserved inner membrane protein	Integral Membrane Protein	COG3059;Predicted membrane protein			
ykgC	CDS	predicted oxidoreductase with FAD/NAD(P)-binding domain and dimerization domain	Cytoplasmic	COG1249;Pyruvate/2-oxoglutarate dehydrogenase complex, dihydrolipoamide dehydrogenase (E3) component, and related enzymes			
ykgD	CDS	predicted DNA-binding transcriptional regulator	Cytoplasmic	COG2207;AraC-type DNA-binding domain-containing proteins	GO:0006350 transcription	o	tt
ykgE	CDS	predicted oxidoreductase	Cytoplasmic	COG0247;Fe-S oxidoreductase			
ykgF	CDS	predicted amino acid dehydrogenase with NAD(P)-binding domain and ferridoxin-like domain	Cytoplasmic	COG1139;Uncharacterized conserved protein containing a ferredoxin-like domain		o	tt
ykgG	CDS	predicted transporter	Cytoplasmic				
ykgH	CDS	predicted inner membrane protein	Integral Membrane Protein				
ykgI	CDS	predicted protein	Periplasmic				
ykgJ	CDS	predicted ferredoxin	Periplasmic	COG0727;Predicted Fe-S-cluster oxidoreductase		gg	o
ykgK	CDS	predicted regulator	Cytoplasmic	COG2771;DNA-binding HTH domain-containing proteins			
ykgL	CDS	predicted protein	Cytoplasmic				
ykgM	CDS	predicted ribosomal protein	Cytoplasmic				
ykgM	CDS	predicted ribosomal protein	Cytoplasmic	COG0254;Ribosomal protein L31			
ykgN	CDS	predicted IS protein	Cytoplasmic			o	tt
ykiA	CDS	predicted protein	Cytoplasmic			gg	o
ykiB	CDS	predicted protein	Cytoplasmic				
ylaB	CDS	conserved inner membrane protein	Integral Membrane Protein	COG4943;Predicted signal transduction protein containing sensor and EAL domains			
ylaC	CDS	predicted inner membrane protein	Integral Membrane Protein				
ylbA	CDS	conserved protein	Cytoplasmic	COG3257;Uncharacterized protein, possibly involved in glyoxylate		gg	t
ylbE	CDS	predicted protein, N-ter fragment (pseudogene)	Cytoplasmic			gg	o
ylbE	ancestral	predicted protein (pseudogene)	Cytoplasmic				
ylbE	CDS	predicted protein, C-ter fragment (pseudogene)	Cytoplasmic			o	o
ylbF	CDS	conserved protein	Cytoplasmic			o	tt
ylbG	CDS	predicted DNA-binding transcriptional regulator	Cytoplasmic				
ylbH	CDS	conserved protein, rhs-like	Cytoplasmic	COG3209;Rhs family protein		o	o
ylcE	CDS	DLP12 prophage; predicted protein	Cytoplasmic				
ylcG	CDS	DLP12 prophage; predicted protein	Cytoplasmic				
yliA	CDS	fused predicted peptide transport subunits -!- ATP-binding components of ABC superfamily	Cytoplasmic	COG1123;ATPase components of various ABC-type transport systems, contain duplicated ATPase			
yliB	CDS	predicted peptide transporter subunit -!- periplasmic-binding component of ABC superfamily	Periplasmic	COG0747;ABC-type dipeptide transport system, periplasmic component			
yliC	CDS	predicted peptide transporter subunit -!- membrane component of ABC superfamily	Integral Membrane Protein	COG0601;ABC-type dipeptide/oligopeptide/nickel transport systems, permease components		o	tt
yliD	CDS	predicted peptide transporter subunit -!- membrane component of ABC superfamily	Integral Membrane Protein	COG1173;ABC-type dipeptide/oligopeptide/nickel transport systems, permease components		o	tt
yliE	CDS	conserved inner membrane protein	Integral Membrane Protein	COG2200;FOG: EAL domain		o	tt
yliF	CDS	predicted diguanylate cyclase	Integral Membrane Protein	COG2199;FOG: GGDEF domain		o	tt
yliG	CDS	predicted SAM-dependent methyltransferase	Cytoplasmic	COG0621;2-methylthioadenine synthetase		gg	t
yliH	CDS	conserved protein	Cytoplasmic			gg	t
yliI	CDS	predicted dehydrogenase	Periplasmic	COG2133;Glucose/sorbosone dehydrogenases		o	tt
yliJ	CDS	predicted glutathione S-transferase	Cytoplasmic			gg	t
yliK	CDS	methylmalonyl-CoA mutase	Cytoplasmic	COG1884;Methylmalonyl-CoA mutase, N-terminal domain/subunit -!- COG2185;Methylmalonyl-CoA mutase, C-terminal domain/subunit (cobalamin-binding)	GO:0019395 fatty acid oxidation	gg	t
yliL	CDS	predicted protein	Cytoplasmic				
ymbA	CDS	conserved protein	Cytoplasmic	COG3009;Uncharacterized protein conserved in bacteria			
ymcA	CDS	conserved protein	Periplasmic			gg	tt
ymcB	CDS	conserved protein	Periplasmic			gg	o
ymcC	CDS	predicted outer membrane lipoprotein	Outer Membrane Lipoprotein			o	tt
ymcD	CDS	predicted protein	Membrane Anchored				
ymcE	CDS	cold shock gene	Membrane Anchored			o	tt
ymdA	CDS	predicted protein	Periplasmic			o	tt
ymdB	CDS	conserved protein	Cytoplasmic	COG2110;Predicted phosphatase homologous to the C-terminal domain of histone macroH2A1		o	o
ymdC	CDS	predicted hydrolase	Cytoplasmic	COG1502;Phosphatidylserine/phosphatidylglycerophos phate/cardiolipin synthases and related enzymes			
ymdE	CDS	predicted protein (pseudogene)	Cytoplasmic				
ymdF	CDS	conserved protein	Cytoplasmic				
ymfA	CDS	predicted inner membrane protein	Integral Membrane Protein				
ymfB	CDS	bifunctional thiamin pyrimidine pyrophosphate hydrolase -!- thiamin pyrophosphate hydrolase	Cytoplasmic	COG0494;NTP pyrophosphohydrolases including oxidative damage repair enzymes		gg	t
ymfC	CDS	23S rRNA pseudouridine synthase	Cytoplasmic	COG1187;16S rRNA uridine-516 pseudouridylate synthase and related pseudouridylate synthases	GO:0009451 RNA modification	gg	o
ymfD	CDS	e14 prophage; predicted SAM-dependent methyltransferase	Cytoplasmic	COG0500;SAM-dependent methyltransferases		o	o
ymfE	CDS	e14 prophage; predicted inner membrane protein	Integral Membrane Protein				
ymfG	CDS	e14 prophage; predicted excisionase	Cytoplasmic			o	tt
ymfH	CDS	e14 prophage; predicted protein	Integral Membrane Protein			o	o
ymfI	CDS	e14 prophage; predicted protein	Cytoplasmic				
ymfJ	CDS	e14 prophage; predicted protein	Cytoplasmic			gg	o
ymfK	CDS	e14 prophage; repressor protein phage e14	Cytoplasmic	COG1974;SOS-response transcriptional repressors (RecA-mediated autopeptidases)		o	tt
ymfL	CDS	e14 prophage; predicted DNA-binding transcriptional regulator	Cytoplasmic			gg	o
ymfM	CDS	e14 prophage; predicted protein	Cytoplasmic			g	t
ymfN	CDS	e14 prophage; predicted DNA-binding transcriptional regulator	Cytoplasmic	COG4626;Phage terminase-like protein, large subunit		o	tt
ymfO	CDS	e14 prophage; conserved protein	Cytoplasmic	COG4695;Phage-related protein		g	o
ymfP	CDS	e14 prophage; conserved protein	Cytoplasmic	COG3299;Uncharacterized homolog of phage Mu protein gp47			
ymfQ	CDS	e14 prophage; conserved protein	Cytoplasmic	COG3778;Uncharacterized protein conserved in bacteria		o	t
ymfR	CDS	e14 prophage; predicted protein	Integral Membrane Protein			o	tt
ymfS	CDS	e14 prophage; predicted protein	Cytoplasmic				
ymfT	CDS	e14 prophage; predicted DNA-binding transcriptional regulator	Cytoplasmic				
ymgA	CDS	predicted protein	Cytoplasmic			gg	o
ymgB	CDS	predicted protein	Cytoplasmic				
ymgC	CDS	predicted protein	Cytoplasmic			g	o
ymgD	CDS	predicted protein	Periplasmic				
ymgE	CDS	predicted inner membrane protein	Integral Membrane Protein	COG2261;Predicted membrane protein		o	tt
ymgF	CDS	predicted protein	Integral Membrane Protein			o	tt
ymgG	CDS	predicted protein	Membrane Anchored				
ymgH	CDS	predicted protein	Cytoplasmic				
ymjA	CDS	predicted protein	Cytoplasmic			gg	o

ymjB	CDS	predicted protein	Cytoplasmic				
ymjC	CDS	predicted oxidoreductase	Cytoplasmic				
ynaA	CDS	Rac prophage; predicted tail protein	Cytoplasmic			gg	tt
ynaE	CDS	Rac prophage; predicted DNA-binding transcriptional regulator	Cytoplasmic			o	tt
ynaI	CDS	conserved inner membrane protein	Integral Membrane Protein	COG0668;Small-conductance mechanosensitive channel		o	tt
ynaJ	CDS	predicted inner membrane protein	Integral Membrane Protein			g	tt
ynaK	CDS	Rac prophage; conserved protein	Cytoplasmic	COG1475;Predicted transcriptional regulators		gg	o
ynbA	CDS	predicted inner membrane protein	Integral Membrane Protein			o	tt
ynbB	CDS	predicted CDP-diglyceride synthase	Integral Membrane Protein	COG4589;Predicted CDP-diglyceride synthetase/phosphatidate cytidylyltransferase	GO:0015949 nucleobase, nucleoside and nucleotide interconversion	o	tt
ynbC	CDS	predicted hydrolase	Cytoplasmic	COG2267;Lysophospholipase -!- COG0500;SAM-dependent methyltransferases		g	t
ynbD	CDS	predicted phosphatase, inner membrane protein	Integral Membrane Protein	COG0671;Membrane-associated phospholipid phosphatase -!- COG2453;Predicted protein-tyrosine phosphatase		o	tt
ynbE	CDS	predicted lipoprotein	Outer Membrane Lipoprotein			o	tt
yncA	CDS	predicted acyltransferase with acyl-CoA N-acyltransferase domain	Cytoplasmic	COG1247;Sortase and related acyltransferases	GO:0042493 response to drug		
yncB	CDS	predicted oxidoreductase, Zn-dependent and NAD(P)-binding	Cytoplasmic				
yncC	CDS	predicted DNA-binding transcriptional regulator	Cytoplasmic	COG1802;Transcriptional regulators	GO:0006350 transcription -!- GO:0006355 regulation of transcription, DNA-dependent	o	tt
yncD	CDS	predicted iron outer membrane transporter	Outer Membrane B-barrel protein	COG1629;Outer membrane receptor proteins, mostly Fe transport		o	tt
yncE	CDS	conserved protein	Periplasmic	COG3391;Uncharacterized conserved protein		o	tt
yncG	CDS	predicted enzyme	Cytoplasmic	COG0625;Glutathione S-transferase		g	t
yncH	CDS	predicted protein	Cytoplasmic				
yncI	CDS	predicted protein	Cytoplasmic			o	tt
yncJ	CDS	predicted protein	Periplasmic			o	tt
yncK	CDS	predicted transposase, N-ter fragment (pseudogene)	Cytoplasmic				
yncK	ancestral	predicted transposase (pseudogene)	Cytoplasmic				
yncK	CDS	predicted transposase, C-ter fragment (pseudogene)	Cytoplasmic				
yncM	CDS	predicted protein	Cytoplasmic				
yncN	CDS	predicted protein	Cytoplasmic				
yneE	CDS	conserved inner membrane protein	Integral Membrane Protein	COG3781;Predicted membrane protein			
yneF	CDS	predicted diguanylate cyclase	Integral Membrane Protein	COG2199;FOG: GGDEF domain			
yneG	CDS	conserved protein	Cytoplasmic			gg	tt
yneH	CDS	predicted glutaminase	Cytoplasmic	COG2066;Glutaminase	GO:0009063 amino acid catabolism	o	tt
yneI	CDS	predicted aldehyde dehydrogenase	Cytoplasmic	COG1012;NAD-dependent aldehyde dehydrogenases			
yneJ	CDS	predicted DNA-binding transcriptional regulator	Cytoplasmic	COG0583;Transcriptional regulator	GO:0006350 transcription	g	t
yneK	CDS	predicted protein	Cytoplasmic			g	o
yneL	CDS	predicted transcriptional regulator	Cytoplasmic	COG2207;AraC-type DNA-binding domain-containing proteins			
ynfA	CDS	conserved inner membrane protein	Integral Membrane Protein	COG1742;Uncharacterized conserved protein		o	tt
ynfB	CDS	predicted protein	Periplasmic			o	tt
ynfC	CDS	predicted protein	Inner Membrane Lipoprotein				
ynfD	CDS	predicted protein	Periplasmic				
ynfE	CDS	oxidoreductase subunit	Periplasmic	COG0243;Anaerobic dehydrogenases, typically selenocysteine-containing		gg	tt
ynfF	CDS	oxidoreductase subunit	Periplasmic	COG0243;Anaerobic dehydrogenases, typically selenocysteine-containing			
ynfG	CDS	oxidoreductase, Fe-S subunit	Periplasmic	COG0437;Fe-S-cluster-containing hydrogenase components 1		g	t
ynfH	CDS	oxidoreductase, membrane subunit	Integral Membrane Protein	COG3302;DMSO reductase anchor subunit			
ynfK	CDS	predicted dethiobiotin synthetase	Cytoplasmic		GO:0009102 biotin biosynthesis		
ynfL	CDS	predicted DNA-binding transcriptional regulator	Cytoplasmic	COG0583;Transcriptional regulator	GO:0006355 regulation of transcription, DNA-dependent	g	o
ynfM	CDS	predicted transporter	Integral Membrane Protein	COG0477;Permeases of the major facilitator superfamily		o	tt
ynfN	CDS	Qin prophage; predicted protein	Cytoplasmic				
ynfO	CDS	Qin prophage; predicted protein	Cytoplasmic				
ynfP	CDS	predicted protein (pseudogene)	Cytoplasmic				
ynhG	CDS	conserved protein	Periplasmic	COG1376;Uncharacterized protein conserved in bacteria		o	tt
yniA	CDS	predicted phosphotransferase/kinase	Cytoplasmic	COG3001;Fructosamine-3-kinase		g	t
yniB	CDS	predicted inner membrane protein	Integral Membrane Protein			o	tt
yniC	CDS	predicted hydrolase	Cytoplasmic	COG0637;Predicted phosphatase/phosphohexomutase		gg	t
yniD	CDS	predicted protein	Membrane Anchored				
ynjA	CDS	conserved protein	Cytoplasmic	COG2128;Uncharacterized conserved protein			
ynjB	CDS	conserved protein	Periplasmic	COG4134;ABC-type uncharacterized transport system, periplasmic component			
ynjC	CDS	fused transporter subunits -!- membrane component of ABC superfamily	Integral Membrane Protein				
ynjD	CDS	predicted transporter subunit -!- ATP-binding component of ABC superfamily	Cytoplasmic	COG4136;ABC-type uncharacterized transport system, ATPase component			
ynjE	CDS	predicted thiosulfate sulfur transferase	Periplasmic		GO:0006790 sulfur metabolism		
ynjF	CDS	predicted phosphatidyl transferase, inner membrane protein	Integral Membrane Protein	COG0558;Phosphatidylglycerophosphate synthase		o	tt
ynjH	CDS	predicted protein	Periplasmic			o	tt
ynjI	CDS	predicted inner membrane protein	Integral Membrane Protein				
yoaA	CDS	conserved protein with nucleoside triphosphate hydrolase domain	Cytoplasmic	COG1199;Rad3-related DNA helicases		o	tt
yoaB	CDS	conserved protein	Cytoplasmic				
yoaC	CDS	predicted protein	Cytoplasmic				
yoaD	CDS	predicted phosphodiesterase	Membrane Anchored			o	tt
yoaE	CDS	fused predicted membrane proteins	Integral Membrane Protein	COG0861;Membrane protein TerC, possibly involved in tellurium resistance -!- COG1253;Hemolysins and related proteins containing CBS domains		o	tt
yoaF	CDS	conserved outer membrane protein	Outer Membrane Lipoprotein	COG3042;Putative hemolysin		o	tt
yoaG	CDS	predicted protein	Cytoplasmic			gg	o
yoaH	CDS	conserved protein	Cytoplasmic	COG3140;Uncharacterized protein conserved in bacteria		gg	o
yoaI	CDS	predicted protein	Cytoplasmic				
yobA	CDS	conserved protein	Periplasmic	COG2372;Uncharacterized protein, homolog of Cu resistance protein		o	tt
yobB	CDS	conserved protein	Cytoplasmic	COG0388;Predicted amidohydrolase		g	o
yobD	CDS	conserved inner membrane protein	Integral Membrane Protein	COG4811;Predicted membrane protein		o	tt
yobF	CDS	predicted protein	Cytoplasmic				
yobG	CDS	predicted protein	Periplasmic				
yobH	CDS	predicted protein	Membrane Anchored				
yodA	CDS	conserved metal-binding protein	Periplasmic	COG3443;Predicted periplasmic or secreted protein		o	tt
yodB	CDS	predicted cytochrome	Integral Membrane Protein		GO:0017004 cytochrome biogenesis		
yodC	CDS	predicted protein	Cytoplasmic			gg	t
yodD	CDS	predicted protein	Cytoplasmic				
yoeA	CDS	CP4-44 prophage; predicted disrupted hemin or colicin receptor (pseudogene)	Outer Membrane B-barrel protein	COG1629;Outer membrane receptor proteins, mostly Fe transport			
yoeB	CDS	toxin of the YoeB-YefM toxin-antitoxin system	Cytoplasmic				
yoeE	CDS	CP4-44 prophage; predicted disrupted hemin or colicin receptor	Cytoplasmic	COG1629;Outer membrane receptor proteins, mostly Fe transport		gg	o
yoeF	CDS	conserved protein	Cytoplasmic				
yohC	CDS	predicted inner membrane protein	Integral Membrane Protein				
yohD	CDS	conserved inner membrane protein	Integral Membrane Protein	COG0586;Uncharacterized membrane-associated protein		o	tt
yohF	CDS	predicted oxidoreductase with NAD(P)-binding Rossmann-fold domain	Cytoplasmic	COG1028;Dehydrogenases with different specificities (related to short-chain alcohol dehydrogenases)		g	t
yohG	CDS	predicted outer membrane protein	Cytoplasmic	COG1538;Outer membrane protein			
yohH	CDS	conserved protein	Cytoplasmic	COG1538;Outer membrane protein			
yohJ	CDS	conserved inner membrane protein	Integral Membrane Protein	COG1380;Putative effector of murein hydrolase LrgA		o	tt
yohK	CDS	predicted inner membrane protein	Integral Membrane Protein	COG1346;Putative effector of murein hydrolase		o	tt
yohL	CDS	conserved protein	Cytoplasmic	COG1937;Uncharacterized protein conserved in bacteria		gg	o
yohM	CDS	membrane protein conferring nickel and cobalt resistance	Integral Membrane Protein	COG2215;ABC-type uncharacterized transport system, permease component		o	tt
yohN	CDS	predicted protein	Periplasmic				
yohO	CDS	predicted protein	Membrane Anchored				
yojI	CDS	fused predicted multidrug transport subunits -!- membrane component and ATP-binding component of ABC superfamily	Integral Membrane Protein	COG4615;ABC-type siderophore export system, fused ATPase and permease components		g	tt
yojL	CDS	predicted thiamine biosynthesis lipoprotein	Inner Membrane Lipoprotein	COG1477;Membrane-associated lipoprotein involved in thiamine biosynthesis	GO:0009228 thiamin biosynthesis		
ypaA	CDS	predicted protein (pseudogene)	Cytoplasmic				
ypdA	CDS	predicted sensory kinase in two-component system with YpdB	Integral Membrane Protein	COG3275;Putative regulator of cell autolysis			
ypdB	CDS	predicted response regulator in two-component system withYpdA	Cytoplasmic	COG3279;Response regulator of the LytR/AlgR family	GO:0006350 transcription	gg	t
ypdC	CDS	predicted DNA-binding protein	Cytoplasmic	COG2207;AraC-type DNA-binding domain-containing proteins	GO:0006350 transcription	o	tt
ypdD	CDS	fused predicted PTS enzymes Hpr component -!- enzyme I component -!- enzyme IIA component	Cytoplasmic	COG1080;Phosphoenolpyruvate-protein kinase (PTS system EI component in bacteria) -!- COG1762;Phosphotransferase system mannitol/fructose-specific IIA domain (Ntr-type)	GO:0009401 phosphoenolpyruvate-dependent sugar phosphotransferase system	gg	tt
ypdE	CDS	predicted peptidase	Cytoplasmic	COG1363;Cellulase M and related proteins		gg	t
ypdF	CDS	predicted peptidase	Cytoplasmic	COG0006;Xaa-Pro aminopeptidase		g	tt
ypdG	CDS	predicted enzyme IIC component of PTS	Integral Membrane Protein	COG1299;Phosphotransferase system, fructose-specific IIC component	GO:0016052 carbohydrate	g	tt
ypdH	CDS	predicted enzyme IIB component of PTS	Periplasmic	COG1445;Phosphotransferase system fructose-specific component IIB	GO:0009401 phosphoenolpyruvate-dependent sugar phosphotransferase system		
ypdI	CDS	predicted lipoprotein involved in colanic acid biosynthesis	Outer Membrane Lipoprotein				

ypdJ	CDS	predicted protein	Cytoplasmic				
ypeA	CDS	predicted acyltransferase with acyl-CoA N-acyltransferase domain	Cytoplasmic			gg	t
ypeB	CDS	predicted protein	Cytoplasmic				
ypeC	CDS	conserved protein	Periplasmic			o	tt
ypfE	CDS	predicted carboxysome structural protein with predicted role in ethanol utilization	Cytoplasmic		GO:0009310 amine catabolism	gg	t
ypfG	CDS	predicted protein	Periplasmic			g	tt
ypfH	CDS	predicted hydrolase	Cytoplasmic	COG0400;Predicted esterase			
ypfI	CDS	predicted hydrolase	Cytoplasmic	COG1444;Predicted P-loop ATPase fused to an acetyltransferase		g	t
ypfJ	CDS	conserved protein	Membrane Anchored	COG2321;Predicted metalloprotease		o	tt
ypfN	CDS	predicted protein	Membrane Anchored			g	tt
yphA	CDS	predicted inner membrane protein	Integral Membrane Protein			o	tt
yphB	CDS	conserved protein	Cytoplasmic	COG2017;Galactose mutarotase and related enzymes		gg	tt
yphC	CDS	predicted oxidoreductase, Zn-dependent and NAD(P)-binding	Cytoplasmic	COG1063;Threonine dehydrogenase and related Zn-dependent dehydrogenases			
yphD	CDS	predicted sugar transporter subunit -!- membrane component of ABC superfamily	Integral Membrane Protein	COG1172;Ribose/xylose/arabinose/galactoside ABC-type transport systems, permease components		o	tt
yphE	CDS	fused predicted sugar transporter subunits -!- ATP-binding components of ABC superfamily	Cytoplasmic	COG1129;ABC-type sugar transport system, ATPase component		gg	tt
yphF	CDS	predicted sugar transporter subunit -!- periplasmic-binding component of ABC superfamily	Periplasmic	COG1879;ABC-type sugar transport system, periplasmic component		o	tt
yphG	CDS	conserved protein	Cytoplasmic				
yphH	CDS	predicted DNA-binding transcriptional regulator	Cytoplasmic		GO:0006355 regulation of transcription, DNA-dependent		
ypjA	CDS	adhesin-like autotransporter	Outer Membrane B-barrel protein	COG3468;Type V secretory pathway, adhesin AidA			
ypjB	CDS	predicted protein	Cytoplasmic			gg	o
ypjC	CDS	predicted protein	Cytoplasmic				
ypjD	CDS	predicted inner membrane protein	Integral Membrane Protein			o	tt
ypjF	CDS	CP4-57 prophage; toxin of the YpjF-YfjZ toxin-antitoxin system	Cytoplasmic			gg	tt
ypjJ	CDS	predicted protein	Cytoplasmic				
ypjK	CDS	CP4-57 prophage; predicted inner membrane protein	Integral Membrane Protein				
ypjL	CDS	CP4-57 prophage; predicted inner membrane protein	Integral Membrane Protein				
ypjM	CDS	CP4-57 prophage; predicted protein (pseudogene)	Cytoplasmic				
yqaA	CDS	conserved inner membrane protein	Integral Membrane Protein	COG1238;Predicted membrane protein		o	tt
yqaB	CDS	predicted hydrolase	Cytoplasmic	COG0637;Predicted phosphatase/phosphohexomutase		gg	t
yqaC	CDS	conserved protein (pseudogene)	Cytoplasmic	COG0366;Glycosidases			
yqaD	CDS	conserved protein	Cytoplasmic	COG0366;Glycosidases		g	o
yqaE	CDS	predicted membrane protein	Integral Membrane Protein	COG0401;Uncharacterized homolog of Blt101		o	tt
yqcA	CDS	predicted flavoprotein	Cytoplasmic	COG0716;Flavodoxins		gg	tt
yqcB	CDS	tRNA pseudouridine synthase	Cytoplasmic	COG0564;Pseudouridylate synthases, 23S RNA-specific		gg	t
yqcC	CDS	conserved protein	Cytoplasmic	COG3098;Uncharacterized protein conserved in bacteria		gg	t
yqcD	CDS	conserved protein	Cytoplasmic	COG2904;Uncharacterized protein conserved in bacteria -!- COG0780;Enzyme related to GTP cyclohydrolase I		gg	t
yqcE	CDS	predicted transporter	Integral Membrane Protein	COG0477;Permeases of the major facilitator superfamily		o	tt
yqeA	CDS	predicted amino acid kinase	Cytoplasmic	COG0549;Carbamate kinase		gg	t
yqeB	CDS	conserved protein with NAD(P)-binding Rossman fold	Cytoplasmic	COG1975;Xanthine and CO dehydrogenases maturation factor, XdhC/CoxF family		gg	t
yqeC	CDS	conserved protein	Cytoplasmic				
yqeF	CDS	predicted acyltransferase	Cytoplasmic	COG0183;Acetyl-CoA acetyltransferase			
yqeG	CDS	predicted transporter	Integral Membrane Protein	COG0814;Amino acid permeases		o	tt
yqeH	CDS	conserved protein with bipartite regulator domain	Cytoplasmic	COG2771;DNA-binding HTH domain-containing proteins			
yqeI	CDS	predicted transcriptional regulator	Cytoplasmic	COG3710;DNA-binding winged-HTH domains			
yqeJ	CDS	predicted protein	Periplasmic				
yqeK	CDS	predicted protein	Cytoplasmic			o	o
yqfA	CDS	predicted oxidoreductase, inner membrane subunit	Integral Membrane Protein	COG1272;Predicted membrane protein, hemolysin III homolog		o	tt
yqfB	CDS	conserved protein	Cytoplasmic	COG3097;Uncharacterized protein conserved in bacteria		gg	o
yqfE	CDS	conserved protein	Cytoplasmic	COG0583;Transcriptional regulator		gg	t
yqgA	CDS	predicted inner membrane protein	Integral Membrane Protein	COG1811;Uncharacterized membrane protein, possible Na+ channel or pump		g	tt
yqgB	CDS	predicted protein	Cytoplasmic			g	o
yqgC	CDS	predicted protein	Cytoplasmic			g	t
yqgD	CDS	predicted inner membrane protein	Integral Membrane Protein			o	tt
yqgE	CDS	predicted protein	Cytoplasmic			gg	o
yqgF	CDS	predicted Holliday junction resolvase	Cytoplasmic	COG0816;Predicted endonuclease involved in recombination (possible Holliday junction resolvase in Mycoplasmas and B. subtilis)		gg	t
yqhA	CDS	conserved inner membrane protein	Integral Membrane Protein	COG2862;Predicted membrane protein		o	tt
yqhC	CDS	predicted DNA-binding transcriptional regulator	Cytoplasmic	COG2207;AraC-type DNA-binding domain-containing proteins	GO:0006350 transcription		
yqhD	CDS	alcohol dehydrogenase, NAD(P)-dependent	Cytoplasmic	COG1979;Uncharacterized oxidoreductases, Fe-dependent alcohol dehydrogenase family		gg	t
yqhG	CDS	conserved protein	Periplasmic				
yqhH	CDS	predicted outer membrane lipoprotein	Outer Membrane Lipoprotein	COG4238;Murein lipoprotein		o	tt
yqiA	CDS	predicted esterase	Cytoplasmic	COG3150;Predicted esterase		o	tt
yqiB	CDS	predicted dehydrogenase	Cytoplasmic	COG3151;Uncharacterized protein conserved in bacteria		gg	t
yqiC	CDS	conserved protein	Cytoplasmic				
yqiG	CDS	predicted outer membrane usher protein	Outer Membrane B-barrel protein				
yqiH	CDS	predicted periplasmic pilin chaperone	Outer Membrane B-barrel protein		GO:0006457 protein folding		
yqiI	CDS	conserved protein	Periplasmic				
yqiJ	CDS	predicted inner membrane protein	Integral Membrane Protein			o	tt
yqiK	CDS	conserved protein	Membrane Anchored	COG2268;Uncharacterized protein conserved in bacteria		o	tt
yqjA	CDS	conserved inner membrane protein	Integral Membrane Protein	COG0586;Uncharacterized membrane-associated protein		gg	tt
yqjB	CDS	conserved protein	Membrane Anchored			o	tt
yqjC	CDS	conserved protein	Periplasmic				
yqjD	CDS	conserved protein	Membrane Anchored	COG4575;Uncharacterized conserved protein		o	o
yqjE	CDS	conserved inner membrane protein	Integral Membrane Protein	COG5393;Predicted membrane protein		o	tt
yqjF	CDS	predicted quinol oxidase subunit	Integral Membrane Protein	COG2259;Predicted membrane protein			
yqjG	CDS	predicted S-transferase	Cytoplasmic	COG0435;Predicted glutathione S-transferase		gg	t
yqjH	CDS	predicted siderophore interacting protein	Cytoplasmic	COG2375;Siderophore-interacting protein		gg	t
yqjI	CDS	predicted transcriptional regulator	Cytoplasmic	COG1695;Predicted transcriptional regulators		g	o
yqjK	CDS	conserved protein	Membrane Anchored			o	tt
yraH	CDS	predicted fimbrial-like adhesin protein	Periplasmic	COG3539;P pilus assembly protein, pilin FimA		o	tt
yraI	CDS	predicted periplasmic pilin chaperone	Periplasmic	COG3121;P pilus assembly protein, chaperone PapD	GO:0006457 protein folding	o	tt
yraJ	CDS	predicted outer membrane protein	Outer Membrane B-barrel protein	COG3188;P pilus assembly protein, porin PapC		o	tt
yraK	CDS	predicted fimbrial-like adhesin protein	Periplasmic	COG3539;P pilus assembly protein, pilin FimA		o	tt
yraL	CDS	predicted methyltransferase	Cytoplasmic	COG0313;Predicted methyltransferases		gg	t
yraM	CDS	conserved protein	Membrane Lipoprotein	COG3107;Putative lipoprotein		o	tt
yraN	CDS	conserved protein	Cytoplasmic	COG0792;Predicted endonuclease distantly related to archaeal Holliday junction resolvase		gg	t
yraO	CDS	DnaA initiator-associating factor for replication initiation	Cytoplasmic	COG0279;Phosphoheptose isomerase		gg	t
yraP	CDS	predicted protein	Outer Membrane Lipoprotein	COG2823;Predicted periplasmic or secreted lipoprotein		o	tt
yraQ	CDS	predicted permease	Integral Membrane Protein	COG0701;Predicted permeases		o	tt
yraR	CDS	predicted nucleoside-diphosphate-sugar epimerase	Cytoplasmic	COG0702;Predicted nucleoside-diphosphate-sugar epimerases			
yrbA	CDS	predicted DNA-binding transcriptional regulator	Cytoplasmic	COG5007;Predicted transcriptional regulator, BolA superfamily	GO:0006355 regulation of transcription, DNA-dependent	gg	o
yrbB	CDS	predicted protein	Cytoplasmic				
yrbC	CDS	predicted ABC-type organic solvent transporter	Periplasmic	COG2854;ABC-type transport system involved in resistance to organic solvents, auxiliary component		o	tt
yrbD	CDS	predicted ABC-type organic solvent transporter	Periplasmic	COG1463;ABC-type transport system involved in resistance to organic solvents, periplasmic component		o	tt
yrbE	CDS	predicted toluene transporter subunit-!- membrane component of ABC superfamily	Integral Membrane Protein	COG0767;ABC-type transport system involved in resistance to organic solvents, permease component		o	tt
yrbF	CDS	predicted toluene transporter subunit -!- ATP-binding component of ABC superfamily	Cytoplasmic	COG1127;ABC-type transport system involved in resistance to organic solvents, ATPase component		gg	t
yrbG	CDS	predicted calcium/sodium:proton antiporter	Integral Membrane Protein	COG0530;Ca2+/Na+ antiporter		o	tt
yrbK	CDS	conserved protein	Membrane Anchored	COG3117;Uncharacterized protein conserved in bacteria		o	tt
yrbL	CDS	predicted protein	Cytoplasmic			gg	t
yrdA	CDS	conserved protein	Cytoplasmic				
yrdB	CDS	conserved protein	Cytoplasmic			gg	o
yrdC	CDS	dsRNA-binding protein	Cytoplasmic	COG0009;Putative translation factor (SUA5)		gg	t
yrdD	CDS	predicted DNA topoisomerase	Cytoplasmic				
yrfA	CDS	predicted protein	Cytoplasmic				
yrfB	CDS	conserved membrane protein	Membrane Anchored			o	tt
yrfC	CDS	predicted fimbrial assembly protein	Membrane Anchored	COG3166;Tfp pilus assembly protein PilN		o	tt
yrfD	CDS	predicted pilus assembly protein	Cytoplasmic				
yrfF	CDS	predicted inner membrane protein	Integral Membrane Protein			o	tt
yrfG	CDS	predicted hydrolase	Cytoplasmic				
yrhA	CDS	conserved protein	Cytoplasmic				
yrhB	CDS	predicted protein	Cytoplasmic			gg	o
yrhC	CDS	predicted protein fragment (pseudogene)	Cytoplasmic				
ysaB	CDS	predicted protein	Outer Membrane Lipoprotein			o	tt
ysdC	CDS	predicted protein (pseudogene)	Cytoplasmic				

ysgA	CDS	predicted hydrolase	Cytoplasmic				
ytfA	CDS	predicted transcriptional regulator	Cytoplasmic	COG1309;Transcriptional regulator		g	o
ytfB	CDS	predicted cell envelope opacity-associated protein	Cytoplasmic	COG3061;Cell envelope opacity-associated protein A			
ytfE	CDS	predicted regulator of cell morphogenesis and cell wall metabolism	Cytoplasmic	COG2846;Regulator of cell morphogenesis and NO signaling		gg	t
ytfF	CDS	predicted inner membrane protein	Integral Membrane Protein	COG0697;Permeases of the drug/metabolite transporter (DMT) superfamily		o	tt
ytfG	CDS	NAD(P)H:quinone oxidoreductase	Cytoplasmic	COG0702;Predicted nucleoside-diphosphate-sugar epimerases		gg	o
ytfH	CDS	predicted transcriptional regulator	Cytoplasmic		GO:0006355 regulation of transcription, DNA-dependent		
ytfI	CDS	predicted protein	Cytoplasmic				
ytfJ	CDS	predicted transcriptional regulator	Periplasmic	COG3054;Predicted transcriptional regulator		o	tt
ytfK	CDS	conserved protein	Cytoplasmic				
ytfL	CDS	predicted inner membrane protein	Integral Membrane Protein	COG1253;Hemolysins and related proteins containing CBS domains		o	tt
ytfM	CDS	predicted outer membrane protein and surface antigen	Outer Membrane B-barrel protein	COG0729;Outer membrane protein		o	tt
ytfN	CDS	conserved protein	Membrane Anchored	COG2911;Uncharacterized protein conserved in bacteria		o	tt
ytfP	CDS	conserved protein	Cytoplasmic	COG2105;Uncharacterized conserved protein		o	tt
ytfQ	CDS	predicted sugar transporter subunit -!- periplasmic-binding component of ABC superfamily	Periplasmic	COG1879;ABC-type sugar transport system, periplasmic component		o	tt
ytfR	CDS	predicted sugar transporter subunit -! ATP-binding component of ABC superfamily	Cytoplasmic				
ytfT	CDS	predicted sugar transporter subunit -!- membrane component of ABC superfamily	Integral Membrane Protein	COG1172;Ribose/xylose/arabinose/galactoside ABC-type transport systems, permease components			
ytjA	CDS	predicted protein	Integral Membrane Protein				
ytjB	CDS	conserved protein	Membrane Anchored	COG3726;Uncharacterized membrane protein affecting hemolysin expression		o	tt
ytjC	CDS	phosphoglyceromutase 2, co-factor independent	Membrane Anchored	COG0406;Fructose-2,6-bisphosphatase	GO:0006096 glycolysis -!- GO:0006094 gluconeogenesis	gg	t
yzcX	CDS	predicted protein	Cytoplasmic				
yzfA	CDS	predicted protein	Cytoplasmic			gg	t
yzgL	CDS	predicted protein	Integral Membrane Protein			o	tt
zapA	CDS	protein that localizes to the cytokinetic ring	Cytoplasmic	COG3027;Uncharacterized protein conserved in bacteria		gg	tt
zipA	CDS	cell division protein involved in Z ring assembly	Periplasmic	COG3115;Cell division protein		o	tt
zitB	CDS	zinc efflux system	Integral Membrane Protein	COG1230;Co/Zn/Cd efflux system component		o	tt
zntA	CDS	zinc, cobalt and lead efflux system	Integral Membrane Protein	COG2217;Cation transport ATPase	GO:0015986 ATP synthesis coupled proton transport -!- GO:0006805 xenobiotic metabolism	o	tt
zntR	CDS	DNA-binding transcriptional activator	Cytoplasmic	COG0789;Predicted transcriptional regulators	GO:0006350 transcription -!- GO:0006805 xenobiotic metabolism	gg	o
znuA	CDS	zinc transporter subunit -!- periplasmic-binding component of ABC superfamily	Periplasmic				
znuB	CDS	zinc transporter subunit -!- membrane component of ABC superfamily	Integral Membrane Protein	COG1108;ABC-type Mn2+/Zn2+ transport systems, permease components		o	tt
znuC	CDS	zinc transporter subunit -!- ATP-binding component of ABC superfamily	Cytoplasmic	COG1121;ABC-type Mn/Zn transport systems, ATPase component		gg	tt
zraP	CDS	Zn-binding periplasmic protein	Periplasmic	COG3678;P pilus assembly/Cpx signaling pathway, periplasmic inhibitor/zinc-resistance associated protein			
zraR	CDS	fused DNA-binding response regulator in two-component regulatory system with ZraS	Cytoplasmic	COG2204;Response regulator containing CheY-like receiver, AAA-type ATPase, and DNA-binding domains	GO:0006113 fermentation -!- GO:0006350 transcription	o	t
zraS	CDS	sensory histidine kinase in two-component regulatory system with ZraR	Integral Membrane Protein	COG0642;Signal transduction histidine kinase	GO:0006113 fermentation -!- GO:0006464 protein modification	o	tt
zupT	CDS	predicted dioxygenase	Cytoplasmic	COG3384;Uncharacterized conserved protein		g	tt
zur	CDS	DNA-binding transcriptional activator, Zn(II)-binding	Cytoplasmic		GO:0006350 transcription		
zwf	CDS	glucose-6-phosphate dehydrogenase	Cytoplasmic	COG0364;Glucose-6-phosphate 1-dehydrogenase	GO:0016052 carbohydrate catabolism -!- GO:0009051 pentose-phosphate shunt, oxidative branch	gg	t

Table S2. Toxicity of essential genes. Toxicity severity was assessed by survivor frequency values. We tested the essential genes causing both severe growth inhibition and no GFP production in Table S1. Results are shown in order of toxicity severity.

Gene	Feature	Product	Location	Function	Process	Survivor frequency ×10^{-6}
dnaX	CDS	DNA polymerase III/DNA elongation factor III, tau and gamma subunits	Cytoplasmic	COG2812;DNA polymerase III, gamma/tau subunits	GO:0006261 DNA dependent DNA replication	0
ftsK	CDS	DNA-binding membrane protein required for chromosome resolution and partitioning	Integral Membrane Protein	COG1674;DNA segregation ATPase FtsK/SpoIIIE and related proteins	GO:0009432 SOS response	0
mrdA	CDS	transpeptidase involved in peptidoglycan synthesis (penicillin-binding protein 2)	Membrane Anchored	COG0768;Cell division protein FtsI/penicillin-binding protein 2	GO:0009252 peptidoglycan biosynthesis -!- GO:0042493 response to drug	0
mrdB	CDS	cell wall shape-determining protein	Integral Membrane Protein	COG0772;Bacterial cell division membrane protein		0
mukB	CDS	fused chromosome partitioning proteins	Cytoplasmic	COG3096;Uncharacterized protein involved in chromosome partitioning		0
rpsD	CDS	30S ribosomal subunit protein S4	Cytoplasmic	COG0522;Ribosomal protein S4 and related proteins	GO:0006412 protein biosynthesis	0
lexA	CDS	DNA-binding transcriptional repressor	Cytoplasmic	COG1974;SOS-response transcriptional repressors (RecA-mediated autopeptidases)	GO:0006281 DNA repair -!- GO:0006350 transcription -!- GO:0009432 SOS response	0.59
pheT	CDS	phenylalanine tRNA synthetase, beta subunit	Cytoplasmic	COG0073;EMAP domain -!- COG0072;Phenylalanyl-tRNA synthetase beta subunit	GO:0006418 amino acid activation	1.4
prfA	CDS	peptide chain release factor RF-1	Cytoplasmic	COG0216;Protein chain release factor A	GO:0006412 protein biosynthesis	1.77
lepB	CDS	leader peptidase (signal peptidase I)	Integral Membrane Protein	COG0681;Signal peptidase I		1.8
polA	CDS	fused DNA polymerase I 5'->3' exonuclease -!- 3'->5' polymerase -!- 3'->5' exonuclease	Cytoplasmic	COG0258;5'-3' exonuclease (including N-terminal domain of PolI) -!- COG0749;DNA polymerase I - 3'-5' exonuclease and polymerase domains	GO:0006261 DNA dependent DNA replication	3.98
leuS	CDS	leucyl-tRNA synthetase	Cytoplasmic	COG0495;Leucyl-tRNA synthetase	GO:0006418 amino acid activation	5.57
ftsI	CDS	transpeptidase involved in septal peptidoglycan synthesis (penicillin-binding protein 3)	Membrane Anchored	COG0768;Cell division protein FtsI/penicillin-binding protein 2	GO:0009252 peptidoglycan biosynthesis -!- GO:0042493 response to drug	8.13
yaeL	CDS	zinc metallopeptidase	Integral Membrane Protein	COG0750;Predicted membrane-associated Zn-dependent proteases 1		8.26
secA	CDS	preprotein translocase subunit, ATPase that targets protein precursors to the SecYE core	Cytoplasmic	COG0653;Preprotein translocase subunit SecA (ATPase, RNA helicase)		8.84
parC	CDS	DNA topoisomerase IV, subunit A	Cytoplasmic	COG0188;Type IIA topoisomerase (DNA gyrase/topo II, topoisomerase IV), A subunit	GO:0006261 DNA dependent DNA replication	11.16
ftsQ	CDS	membrane anchored protein involved in growth of wall at septum	Membrane Anchored	COG1589;Cell division septal protein		11.51
rplP	CDS	50S ribosomal subunit protein L16	Cytoplasmic	COG0197;Ribosomal protein L16/L10E	GO:0006412 protein biosynthesis	16.54
dnaC	CDS	DNA biosynthesis protein	Cytoplasmic	COG1484;DNA replication protein	GO:0006261 DNA dependent DNA replication	19.64
mviN	CDS	predicted inner membrane protein	Integral Membrane Protein	COG0728;Uncharacterized membrane protein, putative virulence factor		20.33
lolE	CDS	outer membrane-specific lipoprotein transporter subunit -!- membrane component of ABC superfamily	Integral Membrane Protein	COG4591;ABC-type transport system, involved in lipoprotein release, permease component		20.34
yejM	CDS	predicted hydrolase, inner membrane	Integral Membrane Protein	COG3083;Predicted hydrolase of alkaline phosphatase superfamily		21.01
tyrS	CDS	tyrosyl-tRNA synthetase	Cytoplasmic	COG0162;Tyrosyl-tRNA synthetase	GO:0006418 amino acid activation	22.51
dnaA	CDS	chromosomal replication initiator protein DnaA, DNA-binding transcriptional dual regulator	Cytoplasmic	COG0593;ATPase involved in DNA replication initiation	GO:0006261 DNA dependent DNA replication	23.89
rplD	CDS	50S ribosomal subunit protein L4	Cytoplasmic	COG0088;Ribosomal protein L4	GO:0006412 protein biosynthesis -!- GO:0006355 regulation of transcription, DNA-dependent -!- GO:0009386 translational attenuation	24.27
rpoD	CDS	RNA polymerase, sigma 70 (sigma D) factor	Cytoplasmic	COG0568;DNA-directed RNA polymerase, sigma subunit (sigma70/sigma32)	GO:0006350 transcription	26.09
ftsH	CDS	protease, ATP-dependent zinc-metallo	Integral Membrane Protein	COG0465;ATP-dependent Zn proteases		27.62
secY	CDS	preprotein translocase membrane subunit	Integral Membrane Protein	COG0201;Preprotein translocase subunit SecY		28.88
hemH	CDS	ferrochelatase	Cytoplasmic	COG0276;Protoheme ferro-lyase (ferrochelatase)		30.85
rpoH	CDS	RNA polymerase, sigma 32 (sigma H) factor	Cytoplasmic	COG0568;DNA-directed RNA polymerase, sigma subunit (sigma70/sigma32)	GO:0006350 transcription -!- GO:0009266 response to temperature	34.94

lgt	CDS	phosphatidylglycerol-prolipoprotein diacylglyceryl transferase	Integral Membrane Protein	COG0682;Prolipoprotein diacylglyceryltransferase	GO:0008654 phospholipid biosynthesis -!- GO:0006464 protein modification	39.68
ftsL	CDS	membrane bound cell division protein at septum containing leucine zipper motif	Membrane Anchored	COG3116;Cell division protein		40.4
rplS	CDS	50S ribosomal subunit protein L19	Cytoplasmic	COG0335;Ribosomal protein L19	GO:0006412 protein biosynthesis	40.62
ribF	CDS	bifunctional riboflavin kinase -!- FAD synthetase	Cytoplasmic	COG0196;FAD synthase		43.72
rpsB	CDS	30S ribosomal subunit protein S2	Cytoplasmic	COG0052;Ribosomal protein S2	GO:0006412 protein biosynthesis	51.92
rpsC	CDS	30S ribosomal subunit protein S3	Cytoplasmic	COG0092;Ribosomal protein S3	GO:0006412 protein biosynthesis	55.12
ftsX	CDS	predicted transporter subunit -!- membrane component of ABC superfamily	Integral Membrane Protein	COG2177;Cell division protein		56.57
yhbN	CDS	predicted transporter subunit -!- periplasmic-binding component of ABC superfamily	Periplasmic	COG1934;Uncharacterized protein conserved in bacteria		58.4
ygjE	CDS	predicted tartrate:succinate antiporter	Integral Membrane Protein	COG0471;Di- and tricarboxylate transporters		64.97
ileS	CDS	isoleucyl-tRNA synthetase	Cytoplasmic	COG0060;Isoleucyl-tRNA synthetase	GO:0006418 amino acid activation	67
rplB	CDS	50S ribosomal subunit protein L2	Cytoplasmic	COG0090;Ribosomal protein L2	GO:0006412 protein biosynthesis	71.05
rpsA	CDS	30S ribosomal subunit protein S1	Cytoplasmic	COG0539;Ribosomal protein S1	GO:0006412 protein biosynthesis	75.1
lpxB	CDS	tetraacyldisaccharide-1-P synthase	Cytoplasmic	COG0763;Lipid A disaccharide synthetase	GO:0009245 lipid A biosynthesis	86.56
pgsA	CDS	phosphatidylglycerophosphate synthetase	Integral Membrane Protein	COG0558;Phosphatidylglycerophosphate synthase	GO:0008654 phospholipid biosynthesis	87.01
tilS	CDS	tRNA(Ile)-lysidine synthetase	Cytoplasmic	COG0037;Predicted ATPase of the PP-loop superfamily implicated in cell cycle control		89.4
msbA	CDS	fused lipid transporter subunits -!- membrane component and ATP-binding component of ABC	Integral Membrane Protein	COG1132;ABC-type multidrug transport system, ATPase and permease components		94.48
ftsW	CDS	integral membrane protein involved in stabilising FstZ ring during cell division	Integral Membrane Protein	COG0772;Bacterial cell division membrane protein		114.92
rpsS	CDS	30S ribosomal subunit protein S19	Cytoplasmic	COG0185;Ribosomal protein S19	GO:0006412 protein biosynthesis	118.51
degS	CDS	serine endoprotease, periplasmic	Membrane Anchored	COG0265;Trypsin-like serine proteases, typically periplasmic, contain C-terminal PDZ domain	GO:0006508 proteolysis and peptidolysis	120.19
infB	CDS	fused protein chain initiation factor 2, IF2	Cytoplasmic	COG0532;Translation initiation factor 2 (IF-2; GTPase)	GO:0006412 protein biosynthesis	130.69
mreC	CDS	cell wall structural complex MreBCD transmembrane component MreC	Membrane Anchored	COG1792;Cell shape-determining protein	GO:0009252 peptidoglycan biosynthesis	135
trmU	CDS	tRNA (5-methylaminomethyl-2-thiouridylate)-methyltransferase	Cytoplasmic		GO:0009451 RNA modification	158.46
yfcC	CDS	predicted inner membrane protein	Integral Membrane Protein	COG1288;Predicted membrane protein		159
dnaB	CDS	replicative DNA helicase	Cytoplasmic	COG0305;Replicative DNA helicase	GO:0006261 DNA dependent DNA replication	161.14
lpxC	CDS	UDP-3-O-acyl N-acetylglucosamine deacetylase	Cytoplasmic	COG0774;UDP-3-O-acyl-N-acetylglucosamine deacetylase	GO:0009245 lipid A biosynthesis	176.47
zipA	CDS	cell division protein involved in Z ring assembly	Periplasmic	COG3115;Cell division protein		183.23
yjgP	CDS	conserved inner membrane protein	Integral Membrane Protein	COG0795;Predicted permeases		191.73
yrbK	CDS	conserved protein	Membrane Anchored	COG3117;Uncharacterized protein conserved in bacteria		191.78
lspA	CDS	prolipoprotein signal peptidase (signal peptidase II)	Integral Membrane Protein	COG0597;Lipoprotein signal peptidase		219
murG	CDS	N-acetylglucosaminyl transferase	Membrane Associated	COG0707;UDP-N-acetylglucosamine:LPS N-acetylglucosamine transferase	GO:0009252 peptidoglycan biosynthesis	219.69
yrfF	CDS	predicted inner membrane protein	Integral Membrane Protein			237.18
proS	CDS	prolyl-tRNA synthetase	Cytoplasmic	COG0442;Prolyl-tRNA synthetase	GO:0006418 amino acid activation	266.67
lolA	CDS	chaperone for lipoproteins	Periplasmic	COG2834;Outer membrane lipoprotein-sorting protein		271.54
mreD	CDS	cell wall structural complex MreBCD transmembrane component MreD	Integral Membrane Protein	COG2891;Cell shape-determining protein	GO:0009252 peptidoglycan biosynthesis	282.95
plsB	CDS	glycerol-3-phosphate O-acyltransferase	Cytoplasmic		GO:0008654 phospholipid biosynthesis	290.23
dxs	CDS	1-deoxyxylulose-5-phosphate synthase, thiamine-requiring, FAD-requiring	Cytoplasmic	COG1154;Deoxyxylulose-5-phosphate synthase	GO:0008615 pyridoxine biosynthesis -!- GO:0009228 thiamin biosynthesis	291.14

ssb	CDS	Single-stranded DNA-binding protein	Cytoplasmic	COG0629;Single-stranded DNA-binding protein	GO:0006310 DNA recombination -!- GO:0009432 SOS response	291.14
frr	CDS	ribosome recycling factor	Cytoplasmic	COG0233;Ribosome recycling factor	GO:0006412 protein biosynthesis -!- GO:0009386 translational attenuation	342.39
mraY	CDS	phospho-N-acetylmuramoyl-pentapeptide transferase	Integral Membrane Protein	COG0472;UDP-N-acetylmuramyl pentapeptide phosphotransferase/UDP-N-acetylglucosamine-1-phosp hate transferase	GO:0009252 peptidoglycan biosynthesis	345.24
rho	CDS	transcription termination factor	Cytoplasmic	COG1158;Transcription termination factor	GO:0006350 transcription	355.88
accB	CDS	acetyl CoA carboxylase, BCCP subunit	Cytoplasmic	COG0511;Biotin carboxyl carrier protein	GO:0042966 biotin carboxyl carrier protein biosynthesis	361.49
argS	CDS	arginyl-tRNA synthetase	Cytoplasmic	COG0018;Arginyl-tRNA synthetase	GO:0006418 amino acid activation	393.73
ftsE	CDS	predicted transporter subunit -!- ATP-binding component of ABC	Cytoplasmic	COG2884;Predicted ATPase involved in cell division		396.37
secD	CDS	SecYEG protein translocase auxillary subunit	Integral Membrane Protein	COG0342;Preprotein translocase subunit SecD		406.78
yidC	CDS	cytoplasmic insertase into membrane protein, Sec system	Integral Membrane Protein	COG0706;Preprotein translocase subunit YidC		465.62
grpE	CDS	heat shock protein	Cytoplasmic	COG0576;Molecular chaperone GrpE (heat shock protein)	GO:0006457 protein folding	471.07
psd	CDS	phosphatidylserine decarboxylase	Cytoplasmic	COG0688;Phosphatidylserine decarboxylase	GO:0008654 phospholipid biosynthesis	552.24
ftsY	CDS	fused Signal Recognition Particle (SRP) receptor	Cytoplasmic	COG0552;Signal recognition particle GTPase		700.89
groL	CDS	Cpn60 chaperonin GroEL, large subunit of GroESL	Cytoplasmic	COG0459;Chaperonin GroEL (HSP60 family)	GO:0006457 protein folding	715.35
lnt	CDS	apolipoprotein N-acyltransferase	Integral Membrane Protein	COG0815;Apolipoprotein N-acyltransferase	GO:0042158 lipoprotein biosynthesis	783.51
rplQ	CDS	50S ribosomal subunit protein L17	Cytoplasmic	COG0203;Ribosomal protein L17	GO:0006412 protein biosynthesis	828.27
secE	CDS	preprotein translocase membrane subunit	Integral Membrane Protein	COG0690;Preprotein translocase subunit SecE		852.43
hemA	CDS	glutamyl tRNA reductase	Cytoplasmic	COG0373;Glutamyl-tRNA reductase	GO:0042493 response to drug	863.95
rpsM	CDS	30S ribosomal subunit protein S13	Cytoplasmic	COG0099;Ribosomal protein S13	GO:0006412 protein biosynthesis	906.63
accC	CDS	acetyl-CoA carboxylase, biotin carboxylase subunit	Cytoplasmic	COG0439;Biotin carboxylase		977.62
plsC	CDS	1-acyl-sn-glycerol-3-phosphate acyltransferase	Membrane Anchored	COG0204;1-acyl-sn-glycerol-3-phosphate acyltransferase	GO:0008654 phospholipid biosynthesis	983.05
fmt	CDS	10-formyltetrahydrofolate:L-methionyl-tRNA(fMet) N-formyltransferase	Cytoplasmic	COG0223;Methionyl-tRNA formyltransferase	GO:0009451 RNA modification	1044.25
ubiA	CDS	p-hydroxybenzoate octaprenyltransferase	Integral Membrane Protein	COG0382;4-hydroxybenzoate polyprenyltransferase and related prenyltransferases	GO:0009060 aerobic respiration	1087.98
ftsB	CDS	cell division protein	Membrane Anchored	COG2919;Septum formation initiator		1234.03
nusG	CDS	transcription termination factor	Cytoplasmic	COG0250;Transcription antiterminator	GO:0006350 transcription	1331.23
secF	CDS	SecYEG protein translocase auxillary subunit	Integral Membrane Protein	COG0341;Preprotein translocase subunit SecF		1355.12
rlpB	CDS	minor lipoprotein	Membrane Lipoprotein	COG2980;Rare lipoprotein B	GO:0042158 lipoprotein biosynthesis	1561.64
rpoC	CDS	RNA polymerase, beta prime subunit	Cytoplasmic	COG0086;DNA-directed RNA polymerase, beta' subunit/160 kD subunit	GO:0006350 transcription	1804.79
rpsG	CDS	30S ribosomal subunit protein S7	Cytoplasmic	COG0049;Ribosomal protein S7	GO:0006412 protein biosynthesis	2018.29
glyS	CDS	glycine tRNA synthetase, beta subunit	Cytoplasmic	COG0751;Glycyl-tRNA synthetase, beta subunit	GO:0006418 amino acid activation	2035.76
rpsH	CDS	30S ribosomal subunit protein S8	Cytoplasmic	COG0096;Ribosomal protein S8	GO:0006412 protein biosynthesis -!- GO:0009386 translational attenuation	3325.3
rplN	CDS	50S ribosomal subunit protein L14	Cytoplasmic	COG0093;Ribosomal protein L14	GO:0006412 protein biosynthesis	4195.98
dnaE	CDS	DNA polymerase III alpha subunit	Cytoplasmic	COG0587;DNA polymerase III, alpha subunit	GO:0006261 DNA dependent DNA replication	5206.75
holB	CDS	DNA polymerase III, delta prime subunit	Cytoplasmic	COG0470;ATPase involved in DNA replication	GO:0006261 DNA dependent DNA replication	5900.2
rpsR	CDS	30S ribosomal subunit protein S18	Cytoplasmic	COG0238;Ribosomal protein S18	GO:0006412 protein biosynthesis	7792.75
rne	CDS	fused ribonucleaseE endoribonuclease -!- scaffold for formation of degradosome	Cytoplasmic	COG1530;Ribonucleases G and E	GO:0006401 RNA catabolism -!- GO:0006401 RNA catabolism	9038.83
hemG	CDS	protoporphyrin oxidase, flavoprotein	Cytoplasmic	COG4635;Flavodoxin		9679.35

kdtA	CDS	3-deoxy-D-manno-octulosonic-acid transferase (KDO transferase)	Cytoplasmic	COG1519;3-deoxy-D-manno-octulosonic-acid transferase	GO:0009245 lipid A biosynthesis -!- GO:0009244 lipopolysaccharide core region biosynthesis -!- GO:0009248 K antigen biosynthesis	10533.18
rplJ	CDS	50S ribosomal subunit protein L10	Cytoplasmic	COG0244;Ribosomal protein L10	GO:0006412 protein biosynthesis	13807.38
mreB	CDS	cell wall structural complex MreBCD, actin-like component MreB	Cytoplasmic	COG1077;Actin-like ATPase involved in cell morphogenesis	GO:0042493 response to drug	18005.26
ftsA	CDS	ATP-binding cell division protein involved in recruitment of FtsK to Z	Cytoplasmic	COG0849;Actin-like ATPase involved in cell division		26525.2
kdsC	CDS	3-deoxy-D-manno-octulosonate 8-phosphate phosphatase	Periplasmic	COG1778;Low specificity phosphatase (HAD superfamily)		
lpxH	CDS	UDP-2,3-diacylglucosamine pyrophosphatase	Cytoplasmic	COG2908;Uncharacterized protein conserved in bacteria	GO:0009245 lipid A biosynthesis	
spoT	CDS	bifunctional (p)ppGpp synthetase II -!- guanosine-3',5'-bis pyrophosphate 3'-pyrophosphohydrolase	Cytoplasmic	COG0317;Guanosine polyphosphate pyrophosphohydrolases/synthetases	GO:0015949 nucleobase, nucleoside and nucleotide interconversion -!- GO:0042594 response to starvation	
ftsN	CDS	essential cell division protein	Membrane Anchored	COG3087;Cell division protein		
murA	CDS	UDP-N-acetylglucosamine 1-carboxyvinyltransferase	Cytoplasmic	COG0766;UDP-N-acetylglucosamine enolpyruvyl transferase	GO:0009252 peptidoglycan biosynthesis	

Table S3. Toxicity of aminoacyl-tRNA synthetase genes. Genes that exhibit the toxic-overexpression phenotype are highlighted. *tyrS* is highlighted in green. Essential genes are indicated as “e” in the "Essentiality" column.

Gene	Feature	Essentiality	Product	Location	Function	GFP fluorescence	IPTG growth	Survivor frequency ×10^{-6}
pheT	CDS	e	phenylalanine tRNA synthetase, beta subunit	Cytoplasmic	COG0073;EMAP domain -!- COG0072;Phenylalanyl-tRNA synthetase beta subunit	o	tt	1.4
leuS	CDS	e	leucyl-tRNA synthetase	Cytoplasmic	COG0495;Leucyl-tRNA synthetase	o	tt	5.6
tyrS	CDS	e	tyrosyl-tRNA synthetase	Cytoplasmic	COG0162;Tyrosyl-tRNA	o	tt	22.5
ileS	CDS	e	isoleucyl-tRNA synthetase	Cytoplasmic	COG0060;Isoleucyl-tRNA	o	tt	67
tilS	CDS	e	tRNA(Ile)-lysidine synthetase	Cytoplasmic	COG0037;Predicted ATPase of the PP-loop superfamily implicated in cell cycle control	o	tt	89.4
proS	CDS	e	prolyl-tRNA synthetase	Cytoplasmic	COG0442;Prolyl-tRNA synthetase	o	tt	266.7
argS	CDS	e	arginyl-tRNA synthetase	Cytoplasmic	COG0018;Arginyl-tRNA synthetase	o	tt	393.7
glyS	CDS	e	glycine tRNA synthetase, beta subunit	Cytoplasmic	COG0751;Glycyl-tRNA synthetase, beta subunit	o	tt	2035.8
poxA	CDS		predicted lysyl-tRNA synthetase	Cytoplasmic	COG2269;Truncated, possibly inactive, lysyl-tRNA synthetase (class II)	o	tt	
alaS	CDS	e	alanyl-tRNA synthetase	Cytoplasmic	COG0013;Alanyl-tRNA synthetase	gg	tt	
cysS	CDS	e	cysteinyl-tRNA synthetase	Cytoplasmic	COG0215;Cysteinyl-tRNA	gg	tt	
glnS	CDS	e	glutamyl-tRNA synthetase	Cytoplasmic	COG0008;Glutamyl- and glutaminyl-tRNA synthetases	gg	tt	
gltX	CDS	e	glutamyl-tRNA synthetase	Cytoplasmic	COG0008;Glutamyl- and glutaminyl-tRNA synthetases	gg	tt	
serS	CDS	e	seryl-tRNA synthetase, also charges selenocysteinyl-tRNA with	Cytoplasmic	COG0172;Seryl-tRNA synthetase	gg	tt	
trpS	CDS	e	tryptophanyl-tRNA synthetase	Cytoplasmic	COG0180;Tryptophanyl-tRNA synthetase	gg	tt	
valS	CDS	e	valyl-tRNA synthetase	Cytoplasmic	COG0525;Valyl-tRNA synthetase	gg	tt	
asnS	CDS	e	asparaginyl tRNA synthetase	Cytoplasmic	COG0017;Aspartyl/asparaginyl-tRNA synthetases	gg	t	
aspS	CDS	e	aspartyl-tRNA synthetase	Cytoplasmic	COG0173;Aspartyl-tRNA	gg	t	
thrS	CDS	e	threonyl-tRNA synthetase	Cytoplasmic	COG0441;Threonyl-tRNA	gg	t	
lysS	CDS		lysine tRNA synthetase, constitutive	Cytoplasmic	COG1190;Lysyl-tRNA synthetase (class II)	gg	t	
lysU	CDS		lysine tRNA synthetase, inducible	Cytoplasmic	COG1190;Lysyl-tRNA synthetase (class II)	gg	t	
glyQ	CDS	e	glycine tRNA synthetase, alpha subunit	Cytoplasmic	COG0752;Glycyl-tRNA synthetase, alpha subunit	g	t	
metG	CDS	e	methionyl-tRNA synthetase	Cytoplasmic	COG0143;Methionyl-tRNA synthetase -!- COG0073;EMAP	g	t	
hisS	CDS	e	histidyl tRNA synthetase	Cytoplasmic	COG0124;Histidyl-tRNA	gg	o	
pheS	CDS	e	phenylalanine tRNA synthetase, alpha subunit	Cytoplasmic	COG0016;Phenylalanyl-tRNA synthetase alpha subunit			
yadB	CDS		glutamyl-Q tRNA(Asp) synthetase	Cytoplasmic	COG0008;Glutamyl- and glutaminyl-tRNA synthetases			

Table S4. Genomic mutations found in IPTG-resistant mutants. Mutations found in P14R and P30R genomes are shown.

position	P0S	P14R	position	P0S	P30R
			112795	GTTTTTTTTT	GTGGTTTTTTTTTTTTT (upstream)
1737060	AAC	AAAACAC (frame shift) *chbC*	1737060	AAC	AAAACAC,AACCAC (frame shift, insertion) *chbC*
